# UniClo: scarless hierarchical DNA assembly without sequence constraint

**DOI:** 10.1093/nar/gkaf548

**Published:** 2025-06-23

**Authors:** Carol N Flores-Fernández, Da Lin, Katherine Robins, Chris A O’Callaghan

**Affiliations:** Centre for Human Genetics, Nuffield Department of Medicine, University of Oxford, Oxford OX3 7BN, United Kingdom; Centre for Human Genetics, Nuffield Department of Medicine, University of Oxford, Oxford OX3 7BN, United Kingdom; Centre for Human Genetics, Nuffield Department of Medicine, University of Oxford, Oxford OX3 7BN, United Kingdom; Centre for Human Genetics, Nuffield Department of Medicine, University of Oxford, Oxford OX3 7BN, United Kingdom

## Abstract

Type IIS restriction enzyme-mediated DNA assembly is efficient but has sequence constraints and can result in unwanted sequence scars. To overcome these drawbacks, we developed UniClo, a type IIS restriction enzyme-mediated method for universal and flexible DNA assembly. This is achieved through a combination of vector engineering, DNA methylation using recombinant methylases, and steric blockade using CRISPR–dCas9 technology to regulate this methylation. Type IIS restriction enzyme sites within fragments to be assembled are methylated using recombinant methylases, while the fragment-flanking outer sites are protected from methylation by a recombinant dCas9–sgRNA complex. During the subsequent assembly reaction, only the protected flanking sites are cut as only they are unmethylated. Fragments are correctly assembled, despite containing internal sites for the single type IIS restriction enzyme used for the one-pot assembly. The assembled plasmid can be used as a donor plasmid in a subsequent assembly round with the same type IIS restriction enzyme and the assembly vector engineering ensures removal of potential scars by a trimming process. This simplifies assembly design and only three vectors are required for any multi-round assembly. These vectors all use the same pair of overhangs. UniClo provides a simple scarless approach for hierarchical assembly of any sequence and has wide potential application.

## Graphical Abstract

**Graphical Abstract:**
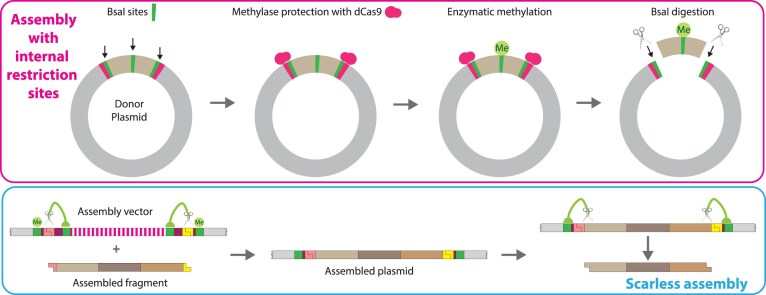

## Introduction

Long lengths of DNA cannot be synthesized directly and must be assembled from shorter fragments. Therefore, DNA assembly methods are required to generate long constructs for diverse applications across fields including synthetic biology, biotechnology, agriculture, medicine, and bioremediation [1–4]. Considerable research effort has led to the development of DNA assembly techniques that can generate long constructs from the assembly of multiple fragments in a simple one-pot reaction [4–7]. In this context, the development of type IIS restriction-based assembly methods, such as Golden Gate and MoClo, have been pivotal [6–8]. These techniques use type IIS restriction endonucleases which cut DNA outside their recognition sequence and can be used to release DNA fragments from donor plasmids. As the generated overhangs can be of any nucleotide sequence, multiple consecutively compatible fragments can be assembled in the correct order without reconstituting the original restriction site [1, 2, 6–8]. For multiple rounds in a hierarchical assembly, multiple assembly vectors and more than one type IIS restriction enzyme have been required. In MoClo, assembly vectors have one set of two “inner” and one set or two “outer” type IIS restriction enzyme sites surrounding the insert site [8]. The two inner sites are both recognized by the same type IIS enzyme and the two outer sites are both recognized by another type IIS enzyme. During the assembly reaction, the inner sites cut outwards to remove a stuffer insert containing these sites. After the assembly reaction, the outer sites can be used to cut inwards to release the newly assembled DNA fragment for use in a further round of assembly. A key limitation is that the fragments to be assembled and the plasmid into which the assembled fragments will be inserted must not contain internal sites for the type IIS restriction enzymes being used in the assembly process. Any such internal sites would need to be mutated (“domesticated”) before the assembly and critically this will result in deviation from the original intended sequence. A further limitation is that over multiple rounds of assembly, short “scars” of unwanted sequence are incorporated into the assembled DNA; these scars represent the overhangs required for interaction with the assembly vectors in each round of the assembly [6–11].

**Figure 1.**
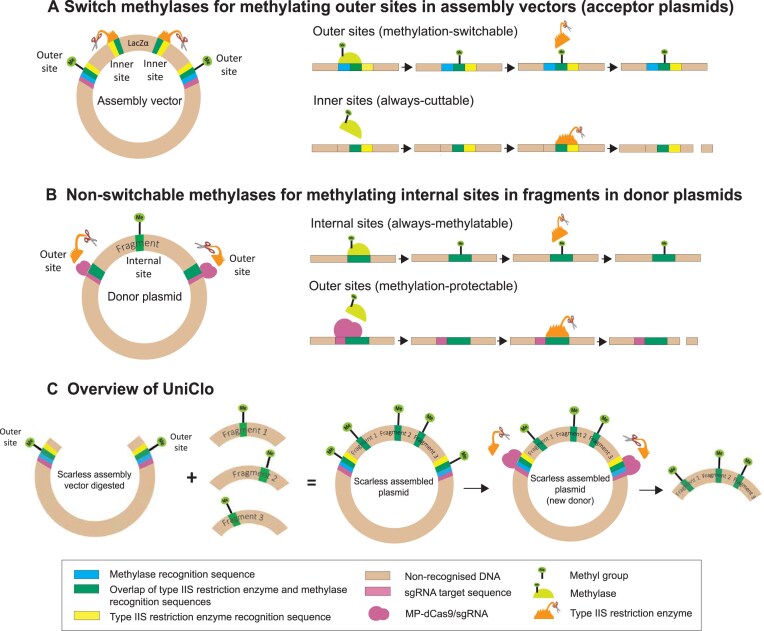
Methylase and type IIS restriction enzyme sites and engineering for DNA assembly. (**A**) A switch methylase is a methylase whose recognition site overlaps partially, but not completely, with a type IIS restriction enzyme recognition site. The design of an original MetClo assembly vector includes two outer and two inner sites for the same type IIS restriction enzyme. The outer sites are “methylation-switchable,” because they are recognized by a switch methylase which can methylate them and so inhibit cutting of these sites by the type IIS restriction enzyme. In contrast, the inner sites are “always-cuttable” as they are not recognized by the switch methylase and so are never methylated by it and are always cut by the restriction enzyme. UniClo vectors also include an sgRNA target sequences which as shown. (**B**) A nonswitchable methylase is a methylase whose recognition site overlaps exactly with the type IIS restriction enzyme recognition site. Therefore, in the presence of the methylase all such restriction enzyme recognition sites will be methylated and so protected from cutting by the restriction enzyme. We term these sites “always-methylatable” and they can occur within any DNA such as in fragments to be assembled. We hypothesized that access of a nonswitchable methylase to an always-methylatable site could be sterically blocked using a CRISPR–dCas9 complex. We term this steric blockade approach “methylation-protection,” and we term the sites that can be protected from methylation “methylation-protectable” sites. Sites can be rendered methylation-protectable by generating an RNA-guided CRISPR–dCas9 complex that binds specifically to the restriction enzyme recognition site and the neighboring DNA. We term the recombinant methylation-protection RNA-guided CRISPR–dCas9 complex MP-dCas9–sgRNA A plasmid acting as a donor plasmid in a DNA assembly contains a fragment flanked by outer restriction enzyme sites which are methylation-protectable and within the fragment there may be one or more internal sites which will be always-methylatable. These sites are always-methylatable because they do not have targets sites for the MP-dCas9–sgRNA complex. The methylation-protection approach could be used to protect the outer sites from methylation while a nonswitchable methylase is used to methylate all the internal sites. (**C**) Before a UniClo assembly reaction the fragments to be assembled are methylated at any internal recognition site for the restriction enzyme used in the assembly. This methylation is carried out in the presence of the MP-dCas9–sgRNA which prevents methylation of the outer flanking restriction enzyme sites. These internal restriction enzyme sites are not cut during the assembly reaction because they are methylated. However, the outer methylation-protectable are cut during the assembly reaction releasing the intact fragments to be assembled. In parallel, during a UniClo assembly reaction, the assembly vector is cut from the outer sites which are not methylated. A scarless assembled plasmid is obtained which can be used as a new donor plasmid using the methylation-protection approach again.

DNA methylation plays a key role in prokaryotic restriction-modification systems and has been deployed in molecular and synthetic biology [9, 12–18]. Methylation of restriction enzyme recognition sites can inhibit the activity of the restriction enzyme at the methylated site. We have previously developed MetClo [10], which is a modification of MoClo [8], in which instead of using two different type IIS restriction enzymes (one for the two inner sites and one for the two outer sites), only one enzyme is used, but with two different types of sites for that one enzyme (Supplementary Figs S1 and S2). This is achieved by engineering the two outer type IIS recognition sites to make them targets for a site-specific methylase which can methylate and inhibit these outer sites but cannot methylate the two inner sites (Fig. 1A, and Supplementary Figs S1 and S2). This allows the outer restriction sites flanking the insert region in the assembly vector (acceptor plasmid) to be methylated before the assembly and so inactive during the assembly. After the assembly, these outer sites can be demethylated (by propagation of the plasmid in a standard *Escherichia coli* strain), so that they can then be cut in the next round of a hierarchical assembly to release the assembled fragment for use in that round of the assembly. As methylation switches off the restriction enzyme site, we termed this process methylation switching.

We have previously generated panels of recombinant methylases and defined different types of site-specific methylases functionally, based on the extent of overlap, if any, between the methylase recognition site and the restriction enzyme recognition [19]. We hypothesized that DNA methylases could be used to overcome the need to avoid “internal” type IIS restriction enzyme sites in the DNA fragments to be assembled. Before the assembly reaction, a methylase could be used to methylate and so block any restriction enzyme sites within the fragments to be assembled or within the donor plasmid in which the fragment is cloned. However, this would also result in methylation and blocking of the outer type IIS restriction enzyme sites in the donor plasmid that flank the DNA fragment and these sites must be cuttable for the fragment to be released and so participate in the assembly reaction. Therefore, these outer sites must be protected from methylation (Fig. 1B).

Steric blockade can be used to inhibit the access of an enzyme to its site of action. Various DNA-binding species could be used to block access of a methylase to its target DNA [20, 21]. The *Streptococcus pyogenes* CRISPR–Cas9 type II system uses a single guide RNA (sgRNA) to conduct a Cas9 nuclease to a sequence-specific DNA location. The sgRNA is a chimeric molecule composed of a variable 20-nucleotide target-specific spacer and constant RNA elements formed by CRISPR RNA (crRNA) and *trans*-activated RNA (tracrRNA). The spacer is complementary to the target DNA sequence and can be modified according to the desired target sequence. The crRNA and the tracrRNA are responsible for forming the Cas9–sgRNA complex. A protospacer-adjacent motif (PAM) located on the noncomplementary strand of the target DNA is required for its recognition and cleavage by the Cas9 nuclease and is NGG for the *S. pyogenes* Cas9. The introduction of two mutations (D10A and H840A) into the *S. pyogenes* Cas9 abolishes its nuclease activity, but this nuclease-deficient Cas9 (dCas9) can still be guided by the sgRNA to the target DNA where it binds the DNA without cutting it [22–28]. This dCas9–sgRNA complex can be used to prevent access of methylases to DNA [21, 29, 30] and has been used to target methylase and demethylase activity to specific sequences in eukaryotic cells [31–34].

We deployed a recombinant CRISPR–dCas9 molecule to specifically bind to, and so sterically block methylation, at the flanking type IIS outer sites in donor plasmids from which DNA fragments will be cut during a DNA assembly [21, 29, 30]. This allows the assembly of DNA fragments which contain internal sites for the type IIS restriction enzyme used during the assembly. We also designed a set of only three assembly vectors that result in the removal of any scars that would otherwise arise during hierarchical DNA assembly, so achieving a fully scarless assembly technique (Fig. 1C). Overall, we report a type IIS enzyme-based DNA assembly approach that can assemble DNA fragments which have internal sites for the type IIS restriction endonuclease used during the assembly and that does not result in unwanted DNA scars in the assembled DNA.

## Materials and methods

### Bacterial strains, plasmids, enzymes, and chemicals

Unless otherwise stated bacterial strains, restriction enzymes, and other molecular biology reagents were purchased from New England Biolabs (NEB, Hitchin, UK), while chemicals were from Sigma–Aldrich, Merck KGaA (Darmstadt, Germany). Plasmids were constructed using standard restriction enzyme/ligation-based cloning techniques or directly synthesized and DNA fragments were generated by DNA synthesis (GenScript) or PCR with Q5 polymerase (NEB). The plasmids containing the DNA fragments for the hierarchical scarless assembly were derived from the human MHC class I polypeptide-related sequence A (MICA) modified gene and were obtained by DNA synthesis (GenScript). The “donor plasmids” are plasmids containing a DNA fragment to be assembled, the “assembly vectors” are acceptor plasmids into which the assembled DNA will be annealed during the assembly, and the “assembled plasmids” are the end result of an assembly in which the assembled DNA has been inserted into an assembly vector during the assembly. For the DNA assembly and methylation-protection assays, the donor plasmids and assembly vectors were based on the ampicillin-resistant MoClo vector pICH47732 (Addgene #48000) [8]. In the donor plasmids, the ampicillin-resistant gene was replaced by kanamycin. For the hierarchical scarless DNA assembly of fragments with internal type IIS restriction sites, the plasmids were constructed based on the chloramphenicol-resistant pMXLC (Addgene #114219) and kanamycin-resistant pMXBK (Addgene #114256) MetClo vectors [10]. The recombinant methylases were produced and purified as we have described previously [19], and a detailed protocol is provided for reference in the Supplementary Information. All other chemicals, plasmids, and services are commercially available, and their sources are listed in Supplementary Table S1. Details of all plasmids used in the assemblies and plasmids used for recombinant methylase production are provided in Supplementary Table S2. Details of the recombinant methylases are provided in Supplementary Table S3.

### DNA assembly using recombinant switch methylase

DNA assembly was assayed using the BsaI-associated switch methylase M.Osp807II for the methylation of the assembly vector POC1355. The fragments 1, 2, 3, and 4 were in the donor plasmids POC1343, POC1344, POC1345, and POC1346, respectively. All reactions were carried out *in vitro* using our recombinant methylase. The methylation of the assembly vector was carried out by mixing 2 μl of 10× methylase buffer (0.5 M Tris–HCl, 0.1 M EDTA, and 50 mM dithiothreitol, pH 7.5), 1 μl of 3200 μM S-adenosylmethionine (SAM), 2000 ng of plasmid, and 200 ng of M.Osp807II in a 20 μl reaction. The mixture was incubated at 37°C for 1 h and heat-inactivated at 80°C for 20 min. The reaction was purified using the QIAquick PCR Purification Kit (Qiagen, Manchester, UK). The assembly reaction was performed by mixing 60 fmol of the methylated POC1355 assembly vector, 60 fmol of each donor plasmid, 2 μl of T4 DNA Ligase buffer, 2.5 μl (1000 U) of T4 DNA Ligase, and 0.25 μl (5 U) of BsaI-HF^®^v2 in a 20 μl of final volume. The assembly was run in a thermocycler (MJ Research–PTC-225 Peltier Thermal Cycler PCR 96 well Tetrad 4 block) incubating the reactions at 37°C for 15 min followed by 45 cycles of 37°C for 2 min and 16°C for 5 min. The reactions were then incubated at 37°C for 20 min and 80°C for 5 min, then held at 4°C. Subsequently, 2 μl of 10× CutSmart buffer and 1 μl (20 U) of BsaI-HF^®^v2 were added, the mixture was incubated at 37°C for 3 h and heat-inactivated at 80°C for 20 min. The assembled reactions (5 μl) were transformed into NEB^®^ 10-beta Competent *E. coli* (80 μl). The transformed cells were plated in LB agar containing 100 μg/ml ampicillin, 100 μM isopropyl β-d-1-thiogalactopyranoside (IPTG), and 50 μg/ml 5-bromo-4-chloro-3-indolyl-β-d-galacto-pyranoside (X-Gal) (Thermo Fisher Scientific, Loughborough, UK), and incubated at 37°C overnight. Transformation efficiency was calculated as described in the Supplementary Methods section of the Supplementary Information. The success rate of the assembly was calculated as the percentage of transformants (white colonies) containing the correctly assembled fragment. The white colonies were analyzed by extracting their plasmids using the QIAprep^®^ Spin Miniprep Kit followed by restriction digestion using BsaI. The restriction digestion products were analyzed by 1% agarose gel electrophoresis to confirm the correct assembly. In addition, these plasmids were sequenced (Source BioScience Genomics) using the primers: Forward CO9566: 5′-TGGTGTAAACAAATTGACGC-3′ and Reverse CO9567: 5′- ACGCCCTTTTAAATATCCG-3′.

### Targeted methylation-protection of type IIS restriction sites using dCas9–sgRNA

The transcribed sgRNA was synthesized using the GeneArt^™^ Precision gRNA Synthesis Kit (Thermo Fisher Scientific). The primers used for the polymerase chain reaction (PCR) construction of the target gRNA DNA template were Forward CO9568360F: 5′-TAATACGACTCACTATAGGTGCAGTACCTCTCACGAC-3′ and Reverse CO9569360R: 5′-TTCTAGCTCTAAAACAGTCGTGAGAGGTACTGCAC-3′. The targeted methylation-protection of the BsaI sites in plasmids was carried out *in vitro* in three steps using a dCas9:sgRNA:target DNA ratio of 10:10:1 (8110 fmol: 8110 fmol: 811 fmol). The amount in ng of sgRNA and target DNA for the reactions based on the fmol required was calculated using the NEBioCalculator (https://nebiocalculator.neb.com) depending on their length. In this three-step process, first, the dCas9 was incubated with the sgRNA; therefore, 3 μl of 10× NEBuffer^™^ r3.1, 313 ng of the sgRNA, and 0.5 μl of EnGen^®^ Spy dCas9 (SNAP-tag^®^) were mixed in a 20 μl volume and incubated at 25°C for 10 min. Second, the target DNA that was to undergo site-selective methylation-protection was added. The plasmids POC1423, POC1427, POC1428, and POC1429 were used as target DNA. These plasmids are all around 5 kb and for each plasmid 811 fmol was ∼2500 ng of DNA. After the addition of the target DNA, the mixture was incubated at 37°C for 15 min. Third, a nonswitchable methylase was added. The three recombinant BsaI-associated nonswitchable methylases used were M2.Eco31I_2, M2.Eco31I and M2.BsaI. In this step, 1 μl of 3200 μM SAM and 500 ng of each methylase were added in a 30 μl final volume. Controls using POC1423 without either dCas9, sgRNA or the methylase and with only the methylase were also tested. The reaction was incubated at 37°C for 15 min, heat-inactivated at 80°C for 20 min, and purified using the QIAquick PCR Purification Kit. Details of the methylation-protection reactions are described in the Supplementary Information (Supplementary Table S4). Restriction digestion was performed to confirm the correct site-selective methylation-protection, using BsaI and BamHI for POC1423 and BsaI for POC1426, POC1427, POC1429, and POC1429.

### DNA assembly using methylases and methylation-protection

DNA assembly using methylation-protection was undertaken using the recombinant BsaI-associated switch methylase M.Osp807II for the methylation of the assembly vector POC1430. The fragments 1, 2, 3, and 4 containing one internal BsaI site each were in the donor plasmids POC1426, POC1427, POC1428, and POC1429, respectively. A dCas9–sgRNA molecule and the recombinant BsaI-associated nonswitchable methylases M2.Eco31I, M2.Eco31I_2, and M2.BsaI were used for the site-selective methylation-protection of the donor plasmids. All the reactions were carried out *in vitro* using recombinant methylases.

Methylation of the assembly vector using recombinant M.Osp807II and targeted methylation-protection of the outer BsaI sites in the donor plasmids were carried out as described above, keeping the dCas9: sgRNA: target DNA ratio of 10:10:1 (8110 fmol: 8110 fmol: 811 fmol) for the methylation-protection. As each of the four donor plasmids was −5 kb and 811 fmol total of target DNA were required, 625 ng of each donor plasmid (−203 fmol each) were used with 500 ng of any of the nonswitchable methylases. The assembly reaction, transformation, and analysis of the assembled plasmids were undertaken as described above. Restriction digestion of the assembled plasmids with DraIII and BsaI was performed to confirm the correct assembly. In addition, the assembled plasmids were sequenced using the primers Forward CO9566: 5′-TGGTGTAAACAAATTGACGC-3′, Forward CO9574: 5′-AGAAATAATGAAACTACGTC-3′, and Reverse CO9567: 5′-ACGCCCTTTTAAATATCCG-3′.

### Hierarchical scarless DNA assembly using a three-vector set, methylases, and methylation-protection

The hierarchical scarless assembly of 11 fragments (1_1 to 1_11) containing multiple internal BsaI sites was undertaken using a three-vector set constructed based on the MoClo vector pICH47732 [10]. The scheme to achieve the final construct of 10.8 kb is flexible and, in this case, involved two rounds of assembly. In the first round, the fragments were assembled in four groups (1_1–1_4, 1_5–1_7, 1_8–1_9, and 1_10–1_11) from donor plasmids with chloramphenicol resistance, and the resulting assembled fragments were designated 2_1, 2_2, 2_3, and 2_4. These assembled fragments were inserted into assembly vectors with kanamycin resistance. In the second round, these four fragments were assembled to construct the final assembled fragment 3_1 in an assembly vector with chloramphenicol resistance.

Methylation of the assembly vectors using recombinant M.Osp807II and targeted methylation-protection of the outer BsaI sites in the donor plasmids were undertaken as described above. The methylation reactions were purified to remove the methylases using the Genomic DNA Clean & Concentrator^™^ Kit-25 (Zymo Research, California, USA). In the first round of assembly, fragments 1_1–1_4 in their corresponding donor plasmids were assembled in POC1518 (VL), 1_5–1_7, and 1_8–1_9 in POC1519 (VM) and 1_10–1_11 in POC1520 (VR). The assembly reactions were performed using 60 fmol of the methylated assembly vectors and 60 fmol of each donor plasmid. The resulting assembled fragments 2_1, 2_2, 2_3, and 2_4 in their corresponding assembled plasmids were used as new donor plasmids for the second round of the assembly. These fragments were assembled in POC1525 (VM) to obtain the final assembled fragment 3_1 in the plasmid POC1550. The second round assembly reaction was performed using 30 fmol of the methylated assembly vector and 30 fmol of each donor plasmid.

Assembled plasmids from the first round of assembly (<10 kb) were transformed into NEB^®^ 10-beta Competent *E. coli* and plated in LB agar containing 30 μg/ml kanamycin, 100 μM IPTG, and 50 μg/ml X-Gal, and incubated at 37°C overnight. The assembled plasmid from the second round of assembly (>10 kb) was transformed in NEB^®^ 10-beta Electrocompetent *E. coli* as follows. The assembly reaction was drop dialysed in 50 ml of UltraPure^™^ DNase/RNase-Free Distilled Water (Thermo Fisher Scientific) using a 0.05 μm membrane filter (MCE 0.05U WH PL 25MM 100PK, Merck KGaA, Darmstadt, Germany) for 1 h. Then, 5 μl of the dialysed sample was transformed into 25 μl of the electrocompetent *E. coli* cells using electroporation conditions of 0.9 kV, 100 Ω, and 25 μF. The transformed cells were plated in LB agar containing 25 μg/ml chloramphenicol, 100 μM IPTG, and 50 μg/ml X-Gal and incubated at 37°C overnight. Transformation efficiency was calculated as described in the Supplementary Methods in the Supplementary Information, and the success rate of the assembly was determined as the percentage of transformants (white colonies) containing the correctly assembled fragment. The white colonies were analyzed by extracting their plasmids using the Monarch^®^ Spin Plasmid Miniprep Kit (NEB) followed by restriction digestion using DraIII and NotI to confirm the correct assembly.

## Results

### DNA assembly *in vitro* using a recombinant switch methylase

We have previously described MetClo, a DNA assembly system built on the Golden Gate/MoClo approach but requiring only one type IIS restriction enzyme because the outer type IIS sites can be deactivated by methylation (Supplementary Figs S1 and S2) [10]. MetClo required the growth of plasmids in special bacterial strains which had been engineered to express particular methylases. The capacity of recombinant switch methylases to inhibit type IIS restriction enzyme activity at appropriately engineered sites [19] was used to develop a simple efficient *in vitro* methylation approach. The assembly vector (acceptor plasmid) POC1355 containing two pairs of BsaI sites was methylated *in vitro* at the methylation-switchable (outer) BsaI sites (Supplementary Fig. S3) using recombinant M.Osp807II, which is a BsaI-associated switch methylase (Supplementary Table S3). The outer BsaI sites are designed to partially overlap with the recognition sequence of the methylase allowing methylation and so inhibition of the restriction enzyme at these “methylation-switchable” sites (Fig. 1A). In contrast, the inner BsaI sites are not recognized by the methylase, and so are never methylated and are “always-cuttable” by the restriction enzyme (Fig. 1A). The donor plasmids POC1343, POC1344, POC1345, and POC1346 (Supplementary Fig. S4) contain fragments of 35, 290, 293, and 294 bp, respectively, that do not include “internal” BsaI sites. These fragments are each flanked by “outer” BsaI sites which are unmethylated and so cuttable by BsaI (Supplementary Fig. S5). Cutting at these BsaI sites releases the fragments, each with different four-base overhangs at each end. During the assembly, these overhangs anneal with complementary overhangs on the neighboring fragments. The outer overhangs of the outer fragments anneal with complementary overhangs in the assembly vector generated by BsaI digestion from the inner always-cuttable BsaI sites of the assembly vector. After the assembly reaction, the assembled plasmid POC1358 was obtained, which contains the four fragments correctly assembled into a 912 bp fragment flanked by the two outer BsaI sites of the assembly vector (Supplementary Fig. S6 and Supplementary Table S5).

Plasmid DNA extracted from white colonies was digested with BsaI resulting in a band of 912 bp (assembled fragment) and a band of 4357 bp (vector backbone), consistent with the correct assembly of the four DNA fragments (Supplementary Fig. S7). The restriction digestion assays demonstrated a success rate of 100% for this assembly since all plasmids analyzed from white colonies contained the correctly assembled fragment (Supplementary Table S6). These results were confirmed by sequencing. This assembled plasmid is suitable for use as a fragment donor plasmid in a further round of assembly using BsaI. Methylation can be removed by transformation and propagation of the plasmid in a standard *E. coli* strain that does not express the M.Osp807II methylase, and the assembled fragment can then be cut out by the action of BsaI on the unmethylated outer BsaI sites as shown in Supplementary Fig. S7.

### Methylation-protection *in vitro* using recombinant nonswitchable methylases and sgRNA-guided dCas9

**Figure 2.**
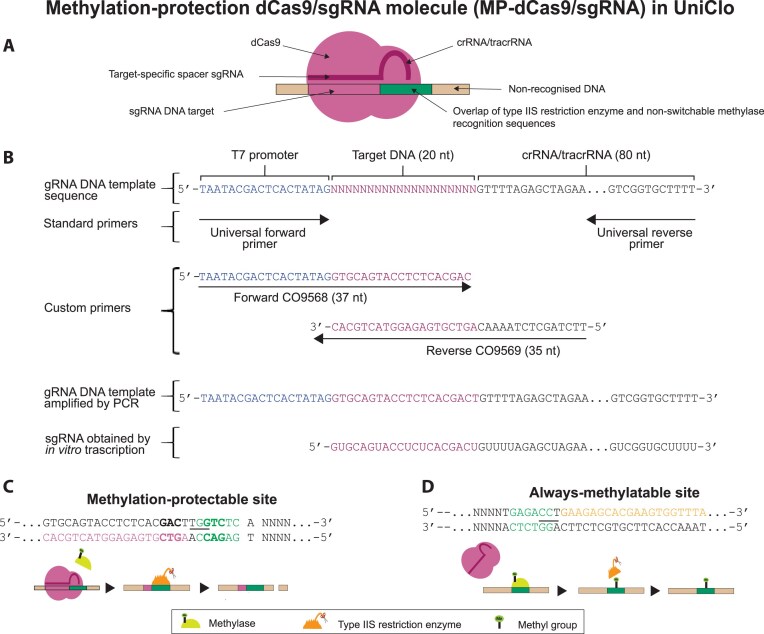
Methylation-protection. (**A**) The MP-dCas9–sgRNA is composed of a deactivated Cas9 nuclease (dCas9) bound to an sgRNA, which conducts the dCas9 to its a target DNA. (**B**) The sgRNA includes a variable target-specific spacer (20 nt) which is complementary to the target DNA and a crRNA–tracrRNA constant region. An sgRNA DNA template is first assembled by PCR amplification using standard and custom primers as illustrated. The sgRNA is then generated by *in vitro* transcription of the amplified DNA template. (**C**) “Methylation-protectable” sites are protected from methylation by the MP-dCas9–sgRNA and so can subsequently be digested by the type IIS restriction enzyme. The MP-dCas9–sgRNA allows the site-selective methylation-protection of a type IIS restriction site that has been suitable engineered with the target DNA sequence for the MP-dCas9–sgRNA and so is a methylation-protectable site. The MP-dCas9–sgRNA target sequence (purple) is engineered to be in close proximity to the nonswitchable methylase site, which overlaps completely with the BsaI site (GGTCTC, shown in green). The target sequence extends from the BsaI site outwards (that is away from where a fragment might be inserted. The PAM sequence (NGG, shown underlined) is positioned within the BsaI site (which is also the nonswitchable methylase site). The target sequence also includes part of recognition site for the switch methylase M.Osp807II (GACNNNGTC, shown in bold) which allows the methylation-protectable BsaI site to also be used as a methylation-switchable BsaI site in a DNA assembly vector. (**D**) “Always-methylatable” sites are not targets for MP-dCas9–sgRNA and so are not protected from methylation and will always be methylated and therefore not digested by the restriction enzyme. The “always-methylatable” site illustrated has a BsaI site (GGTCTC, green) that overlaps completely with a site for the nonswitchable methylase and so is always methylated in the presence of the methylase. The nucleotides (shown in orange) in the equivalent position to the target sequence of the methylation-protectable site are not recognized by the sgRNA around and the nucleotides (underlined) in the equivalent position to the PAM sequence do not form constitute an effective PAM sequence.

The recognition site for the site-specific methylase M2.Eco31I fully overlaps and exactly matches the BsaI recognition site. Therefore, we classify it functionally as a nonswitchable methylase (Fig. 1B and Supplementary Table S3), because in the presence of the methylase, all BsaI sites will be methylated [18]. The plasmid POC1423 (Supplementary Fig. S8) was constructed to have two BsaI sites and one BamHI site interposed between the two BsaI sites. One of the BsaI sites is a standard BsaI site with no additional features and so should always be methylated in the presence of the M2.Eco31I methylase. We term such a site an “always-methylatable site” (Fig. 1B). The other BsaI site was engineered to include a target sequence for an sgRNA in close proximity to the site and a PAM sequence (TGG) within the site on the opposite strand (Fig. 2). The sequence requirement for a PAM sequence for Cas9 from *S. pyogenes* is NGG. An appropriately guided dCas9–sgRNA complex will bind to this target sequence and so potentially sterically block access of the methylase to the BsaI site (Fig. 2 and Supplementary Fig. S8B). We term such a site a “methylation-protectable site” (Figs 1B and 2B) because it is designed to be protected from methylation.

The plasmid target DNA sequence (20 nt) was 3′-CACGTCATGGAGAGTGCTGA-5′ and, therefore, the DNA template sequence required for transcription of the sgRNA was 5′-GTGCAGTACCTCTCACGACT-3′ (Fig. 2B and C). The plasmid target DNA sequence also overlaps with a recognition site for the switch methylase M.Osp807II (GACNNNGTC). This allows the methylation-protectable site to be used as a methylation-switchable site in a DNA assembly vector. An sgRNA of 100 bases with these features was designed and *in vitro* transcribed (Fig. 2 and Supplementary Fig. S9). This sgRNA was used together with recombinant dCas9 to bind to and protect the engineered methylation-protectable BsaI site from methylation by recombinant M2.Eco31I methylase. We term this recombinant methylation-protection dCas9–sgRNA molecule MP-dCas9–sgRNA.

In the absence of methylation or methylation-protection, digestion of the plasmid POC1423 with BamHI and BsaI resulted in three bands of 217, 369, and 4377 as expected (Supplementary Fig. S10). However, when the MP-dCas9–sgRNA and the methylase were both present during an *in vitro* methylase reaction, subsequent digestion with both BsaI and BamHI only resulted in two bands of 217 and 4746 bp indicating that the MP-dCas9–sgRNA had successfully prevented methylation of the methylation-protectable site (Supplementary Fig. S10). In controls where dCas9, sgRNA, or dCas9–sgRNA were absent during the methylase reaction, the methylation-protectable site was methylated. Digestion with both BsaI and BamHI resulted only in linearized plasmid due to restriction at the single BamHI site (Supplementary Fig. S10). In controls where the methylase was absent, none of the BsaI sites were methylated and digestion with both BsaI and BamHI resulted in three bands of 217, 369, and 4377 (Supplementary Fig. S10). These results demonstrate that in the presence of the methylase, the “control” standard always-methylatable site was methylated and so was not cut by BsaI subsequently. However, in the presence of the methylase and MP-dCas9–sgRNA, the engineered methylation-protectable site was protected from methylation and so was cut by BsaI subsequently. Therefore, it is possible to use a recombinant sgRNA-guided dCas9 approach to protect a type IIS restriction site from methylation by a recombinant site-specific methylase.

Methylation-protection was tested further on four plasmids designed to be donor plasmids for DNA assembly. These plasmids, POC1426, POC1427, POC1428, and POC1429 contained DNA fragments of lengths 1022 bp, 846 bp, 850 bp, and 889 bp, respectively. These fragments were flanked on either side by methylation-protectable outer BsaI sites, and each fragment included a standard always-methylatable internal BsaI site which could be methylated by the nonswitchable methylases M2.Eco31I or M2.BsaI (Supplementary Fig. S11). When the MP-dCas9–sgRNA and the methylase were both present during an *in vitro* methylase reaction, subsequent digestion with BsaI resulted in two bands corresponding to the plasmid backbones and the entire fragments released. In the absence of a methylase or in the presence of methylation-protection, digestion by BsaI resulted in three bands due to digestion at the methylation-protectable outer sites and at the internal sites of the fragments (Supplementary Fig. S12).

### Assembly of DNA fragments with internal type IIS restriction sites using recombinant methylases and *in vitro* methylation-protection

The application of methylation-protection was tested in the assembly of DNA fragments which contain internal BsaI sites. The assembly vector POC1430 (Supplementary Fig. S13A) was created such that the two outer flanking BsaI sites are both methylation-switchable by the recombinant switch methylase M.Osp807II and also methylation-protectable by the recombinant MP-dCas9–sgRNA (Supplementary Fig. S13 and Supplementary Table S2). These outer BsaI sites were rendered methylation-protectable by the inclusion of the sgRNA target sequence adjacent to a PAM sequence as outlined above (Fig. 2 and Supplementary Fig. S13B). MP-dCas9–sgRNA is designed to block methylation by either of the nonswitchable methylases M2.Eco31I or M2.BsaI. Prior to the assembly reaction, the recombinant switch methylase M.Osp807II was used to selectively methylate and so inactivate the outer BsaI sites in the assembly vector.

The donor plasmids used for testing this assembly were POC1426, POC1427, POC1428, and POC1429 which are described above and are identical to each other apart from the fragments inserted between their “outer” BsaI sites. The flanking outer BsaI sites are designed to be bound by the MP-dCas9–sgRNA and so can protected from methylation. Each of the fragments contains an internal BsaI site which can be methylated and so blocked from subsequent digestion by BsaI (Supplementary Fig. S14). Prior to the assembly, these donor plasmids containing the fragments to be assembled were methylated by the recombinant nonswitchable methylase M2.Eco31I (Supplementary Table S3) in the presence of the recombinant MP-dCas9–sgRNA. M2.Eco31I can methylate and so inactivate the internal BsaI sites within the fragments, but in the presence of the recombinant MP-dCas9–sgRNA, the outer flanking BsaI sites will be protected from methylation (Supplementary Fig. S12).

An assembly reaction was then undertaken using BsaI with these four donor plasmids and the assembly vector POC1430. During the assembly reaction, the assembly vector POC1430 should only be cut at the two inner always-cuttable BsaI sites within its LacZalpha-containing stuffer insert. Similarly, the donor plasmids should only be cut at their methylation-protectable outer BsaI sites. The ends of each fragment had sequences that when cut by BsaI during the assembly reaction would result in the overhangs necessary for correct assembly. The outermost overhangs of the outer fragments in the assembly were compatible with overhangs in the assembly vector. The other overhangs were compatible with the overhangs of the neighboring fragments in the assembly (Supplementary Fig. S15).

At the end of the assembly reaction, the assembled fragment (3607 bp, Supplementary Table S5) flanked by the methylated outer BsaI sites of the vector should include the four internal (always-methylatable) BsaI sites (Supplementary Fig. S15A). Since the methylation-switchable (outer) sites of the assembly vector are also methylation-protectable, the assembled plasmid (POC1431) could be used as a donor plasmid to release the assembled fragment in a subsequent round of assembly by using the methylation-protection approach with a recombinant nonswitchable methylase and the MP-sgRNA–dCas9 (Supplementary Fig. S15B and C).

The resulting assembled plasmid POC1431 should have six BsaI sites and three DraIII sites (Supplementary Fig. S16). Restriction digestion of the assembled plasmid obtained from white colonies showed six bands of 129, 247, 736, 854, 1641, and 4391 bp for BsaI; and three bands of 35, 3641, and 4322 bp for DraIII consistent with correct assembly of the fragments (Supplementary Fig. S17). Restriction digestion demonstrated a success rate of 100% for this assembly since all plasmids analyzed from white colonies contained the correctly assembled fragments (Supplementary Table S6). Sequencing confirmed the correct assembly and the presence of the internal BsaI sites (Supplementary File S1). Independent assemblies were also conducted using the recombinant nonswitchable methylases M2.Eco31I_2 and M2.BsaI (Supplementary Figs S18 and S19).

### Design and construction of assembly vectors for hierarchical scarless type IIS enzyme-based assembly

**Figure 3.**
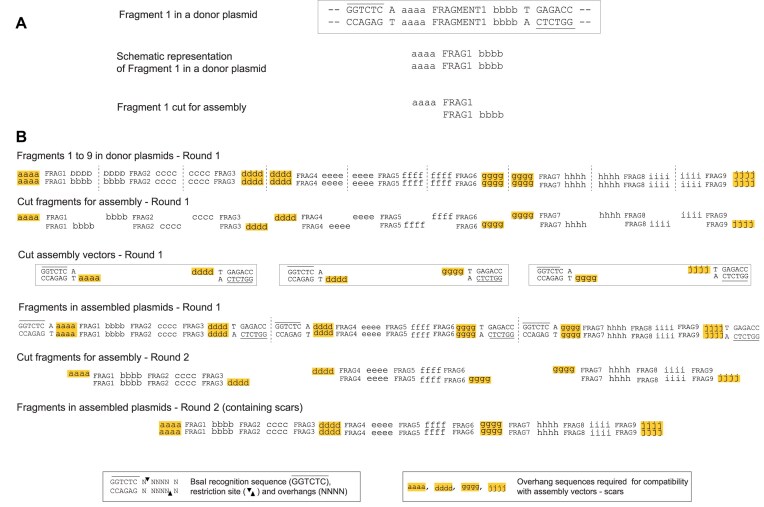
Scar formation in a hierarchical assembly with Golden Gate/MoClo-based DNA assembly methods using type IIS restriction enzymes. (**A**) Schematic representation of a DNA fragment (Fragment 1) in a donor plasmid for an assembly reaction using BsaI, where the four letters aaaa and bbbb represent the overhangs of Fragment 1. (**B**) Schematic representation of two rounds of an assembly of nine fragments using BsaI. In the first round, the fragments are assembled in groups of three. The overhangs between fragments (bbbb, cccc, eeee, ffff, hhhh, and iiii) can be freely chosen from within the sequence to be assembled. Thus, no scars are formed within the assembled fragments. However, the overhangs at the end of the assembled fragments (aaaa, dddd, gggg, and jjjj) must match the overhangs from the assembly vectors. In the second round, these assembled fragments, each containing three original fragments, are assembled to produce the final assembled fragment. Here and in any subsequent rounds, since the overhangs at each end of the assembled fragments are derived from the assembly vectors, they become incorporated into the assembled DNA as short unwanted scars. In this round the scars will be dddd and gggg within the assembled fragment and aaaa and jjjj at the ends of the assembled fragment. It is necessary to have multiple assembly vectors because the sets of terminal overhangs in each assembly in any one round must differ from each other because they will determine the order of assembly in the next round.

In a Golden Gate/MoClo-based DNA assembly method using type IIS restriction enzymes, the cut DNA fragments to be assembled and the cut assembly vector into which the assembled DNA will be inserted must all have appropriate “sticky end” overhangs to allow correctly ordered assembly of the fragments into the assembled plasmid. In a one-round assembly, such as that of plasmid POC1431 above, the overhangs between the fragments to be assembled can be determined by the sequence of the DNA being assembled. These overhangs between fragments must not match the overhangs of the assembly vector. The two terminal overhangs at the ends of the sequence being assembled must differ from each other and each must be a reverse complement of one of the overhangs of the cut assembly vector. This arrangement is illustrated schematically in the first round of the assembly in Fig. 3.

The two terminal overhang sequences must be compatible with the assembly vector, but are otherwise unwanted as they are not an intrinsic part of the original sequence that is to be assembled. The assembled fragment can be used in the next round of a hierarchical assembly with other fragments that have been assembled in a similar manner. In this situation, the overhangs between each of these fragments will be formed by sequences which were chosen in the first round for compatibility with the first round assembly vectors (Fig. 3). These potentially unwanted sequence elements form short scars between the fragments being assembled in the second round. Similarly, in each subsequent round, further scars will be formed at the junctions between fragments produced from assemblies in previous rounds. These assembly designs also require multiple assembly vectors with different combinations of overhang sequences because the sets of terminal overhangs in each assembly in any one round must differ from each other because they will determine the order of assembly in the next round (Fig. 3).

**Figure 4.**
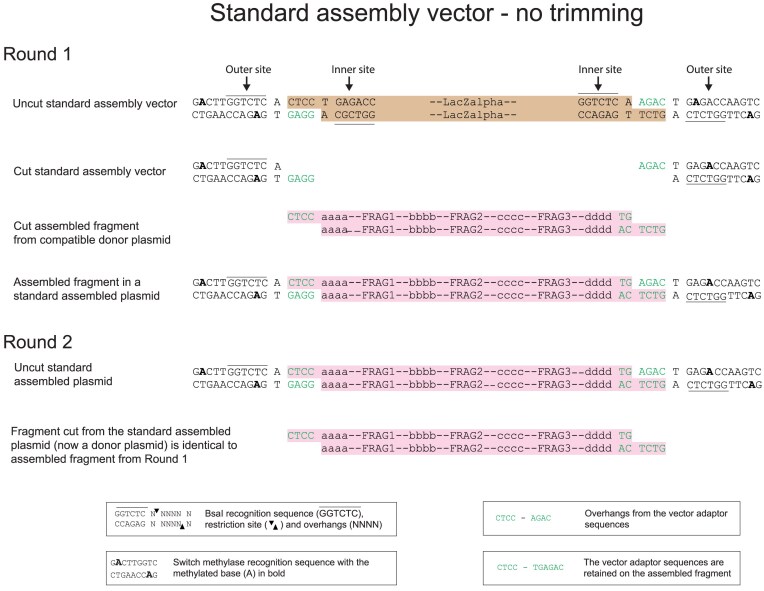
DNA assembly using a standard nontrimming assembly vector. The inner and outer BsaI sites are symmetrically distributed around the overhangs. In the first round of assembly, the outer site is methylated and inactive. During the first round of the assembly cutting from the inner site removes the stuffer fragment (brown) which contains LacZalpha and is replaced with the assembled fragment (pink). In a second round, the outer sites are not methylated and so are active. The assembled fragment (pink) that is released from the assembled plasmid, is identical to the fragment that was assembled in the first round. The overhangs at each end of this assembled fragment become incorporated as short scars between the assembled fragments in the second round (see also Fig. 3).

During a DNA assembly reaction, the assembly vector is cut by a type IIS enzyme which recognizes two inner sites within a stuffer insert and cuts out the insert at a site remote to the enzyme recognition site and flanking the insert (Fig. 4, and Supplementary Figs S1 and S2). After the assembly, the assembled fragment can be released from the assembled plasmid in a subsequent assembly round by the action of a type IIS restriction enzyme on the outer sites of the assembly vector. Therefore, a straightforward way to eliminate the unwanted scars is to move the outer type IIS restriction sites nearer to the ends of the assembled DNA fragment. The consequence of this is that when the fragment is released in the next round of the assembly, the scar remains in the assembled plasmid backbone rather than in the released fragment. As a result, the newly created overhangs are formed within the sequence required, rather than as scars outside this sequence.

**Figure 5.**
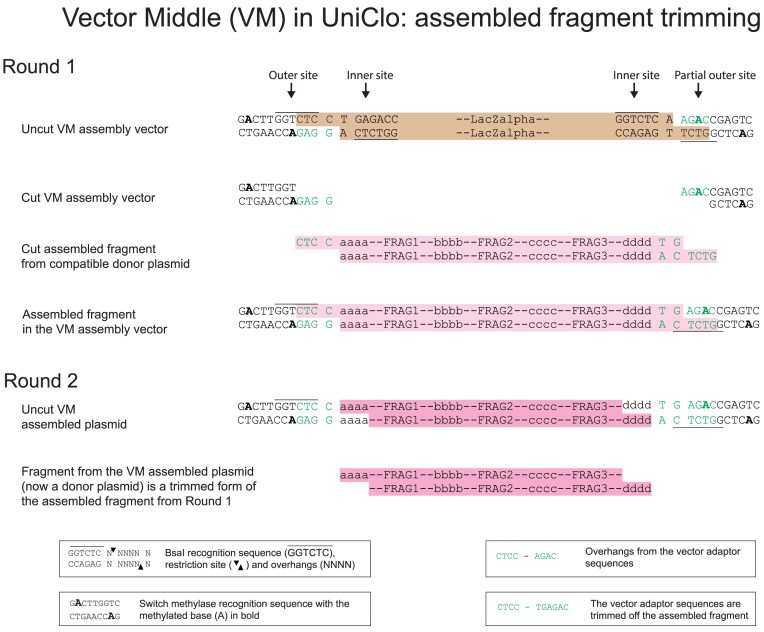
DNA assembly using a scarless VM assembly vector that trims vector adaptor sequences at each end of an assembled fragment. The outer BsaI sites are nearer to the overhangs that form during the assembly than the inner sites. The inner sites remain in the same position in relation to the overhang as the inner sites in the standard vector shown in Fig. 4. The outer BsaI sites are themselves involved in the overhangs that form during the assembly and so are incomplete until the assembly occurs. The stuffer fragment (brown) which contains LacZalpha is removed during the assembly and replaced with the assembled fragment (pink). When the assembled fragment is assembled into the assembly vector, the outer sites are reconstituted in the assembled plasmid. In a subsequent second round of assembly, the fragment (dark pink) that is released from the assembled plasmid is not the same as the fragment that was assembled in the first round. Moreover, the vector adaptor sequences and their overhangs that were used in the first round have been trimmed off. Therefore, there are no scars formed between the fragments assembled in the second round.

We designed assembly vectors in which the site of BsaI cutting from the inner site is closer to the outer than to the inner BsaI recognition site (Fig. 5). To achieve this, the inner site directs cutting outwards into a “vector adaptor sequence” resulting in “vector adaptor overhangs.” The outer site cuts inwards and because it is nearer to the vector adaptor sequence, it cuts beyond this vector adaptor sequence producing an overhang that is derived from the insert and not from the vector adaptor sequence. The outer ends of the terminal fragments in an assembly reaction are designed to have overhangs that match the assembly vector overhangs and to reconstitute the vector adaptor sequence in the assembly vector. This allows the assembly vector to be used in a further round of assembly in the same way as in the previous round, so that the vector adaptor sequence is removed by cutting from the outer BsaI site. The requirement to reconstitute the vector adaptor sequence for BsaI-based assembly, required a 4 bp vector adaptor sequence CTCC at the left end of the fragment and a 6 bp vector adaptor sequence TG AGAC at the right end of the fragment, resulting in the vector overhangs CTCC and AGAC respectively (considering the notional upper or forward strand). This is illustrated in Fig. 5. An important consequence of this assembly vector design is that the same assembly vector can be used in each assembly in any one round because the terminal overhangs which determine the order of assembly of the fragments in the next round are all derived from the required sequence and not from the assembly vector. This significantly simplifies assembly design.

Previously, with a standard arrangement of type IIS restriction sites in which the inner and outer sites of the assembly vector are arranged symmetrically about the overhang sequences, the fragment that is assembled during the first round of an assembly is exactly the same as the fragment that is released for use in a subsequent round of the assembly (Fig. 4). However, this is not the case with the new assembly vector design which includes vector adaptor sequences and has the outer BsaI sites moved nearer to the site of the overhang produced by cutting from the inner BsaI sites. In this situation, the vector overhangs are trimmed off the assembled fragment and so the fragment released for use in the next round is shorter than the fragment that was assembled during the first round (Fig. 5). Therefore, we refer to this as a “trimming” design. This process of trimming takes place at each round of the assembly, so that no unwanted scars are incorporated into the final sequence assembled.

To achieve this trimming, the outer BsaI recognition sites in the assembly vector are positioned such that they overlap the vector overhangs used during the assembly into the vector (Fig. 5). It is important that these outer sites are methylated before the assembly and so blocked from restriction cutting during the assembly. This is achieved using the recombinant switch methylase M.Osp807II which methylates two adenines that are present in the vector, one adenine on each strand. If methylation was not present at this site, the assembled fragment would be cut out of the assembled plasmid during the assembly reaction and the assembly would fail.

In principle, all hierarchical assemblies could be undertaken with a single assembly vector of the same design, but available in two forms, each having a different antibiotic resistance marker. The two assembly vectors could be used in alternating order for sequential rounds of assembly. DNA fragments to be assembled can be constructed such that the outermost fragments of any assembly have sticky end overlaps that correspond to those of the assembly vector. We constructed this vector in two forms, differing only in their antibiotic resistance and term these vector middle (VM) type assembly vectors (Fig. 5). Using VM, vector sequence-derived overhangs are required at each end of the assembled fragment in each round of a multi-round assembly. The use of VM alone would lead to inflexible design constraints.

**Figure 6.**
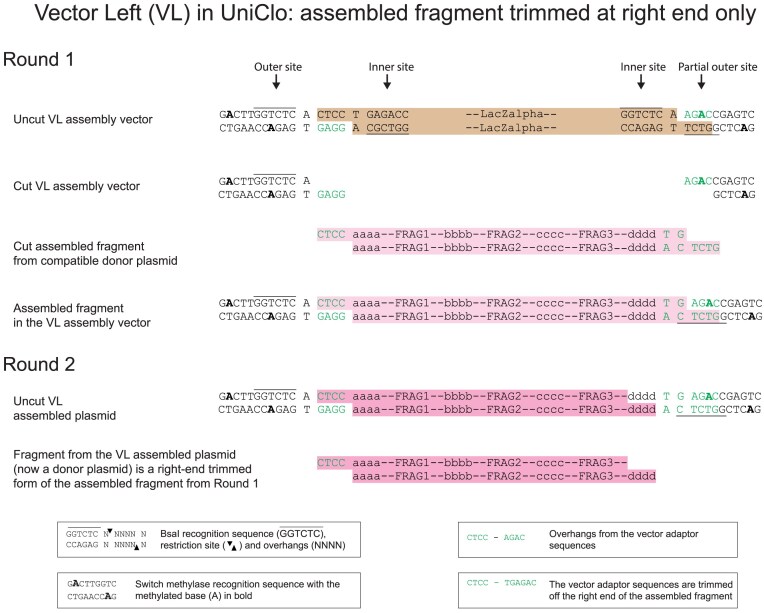
DNA assembly using a scarless VL assembly vector. The design of this vector is a hybrid of the standard vector and VM. At the left end, the inner and outer BsaI sites are symmetrically placed around the overhang. At the right end, the inner and outer sites are asymmetrically distributed with the outer site nearer than the inner site to the overhang. As a consequence, the assembled fragment (pink) from the first round of assembly differs from the fragment released during the second round (dark pink) in that the vector adaptor sequence from the right end has been trimmed whereas the left end has been retained.

**Figure 7.**
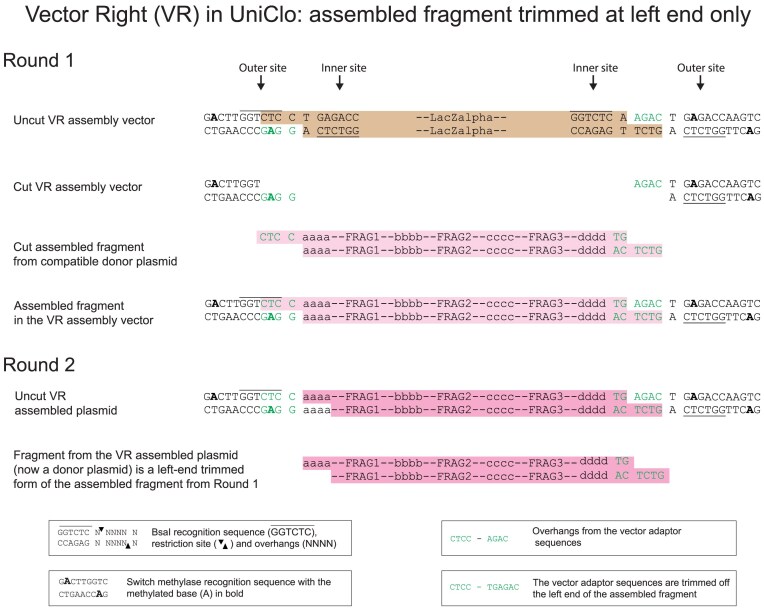
DNA assembly using a scarless VR assembly vector. The design of this vector is a hybrid of the standard vector and VM. At the right end, the inner and outer BsaI sites are symmetrically placed around the overhang. At the left end, the inner and outer sites are asymmetrically distributed with the outer site nearer than the inner site to the overhang. As a consequence, the assembled fragment (pink) from the first round of assembly differs from the fragment released during the second round (dark pink) in that the vector adaptor sequence from the left end has been trimmed whereas the right end has been retained.

A better approach is to use a combination of the standard and fragment trimming arrangements described above. This can be achieved by also using assembly vectors that have the standard distance between the outer BsaI site and the assembled DNA fragment at one end of the fragment, and have a shorter distance between the BsaI site and the assembled DNA fragment at the other end of the fragment (Figs 6 and 7). There are two possibilities for such assembly vectors: one we term vector left (VL) has the standard site at the left and the trimming site at the right (Fig. 6), the other we term vector right (VR) has the trimming site at the left end and the standard site at the right end (Fig. 7). Each was constructed in two forms, which differ only in their antibiotic resistance.

The detailed design and operation of these three new types of assembly vectors are illustrated in Figs 4–7 and Supplementary Fig. S20. For all vector types (standard, VM, VL, and VR), the vector adaptor sequences required in the fragments to be assembled are always CTCC at the left end of the assembled fragment and TGAGAC at the right end of the assembled fragment, and these generate the 4 bp overhangs CTCC and AGAC, respectively, which are compatible with the assembly overhangs. If the same DNA fragments are assembled into each of the different assembly vector types (standard, VM, VL, and VR), then in the next round of the assembly, the fragments released from each of these plasmids will differ in their termini. For the assembly of fragments that will form the left or the right end of the next round of assembly, VL or VR types can be used as the assembly vectors. For the assembly of fragments that will be internal fragments in the next round of assembly or for the final assembled fragment, the VM type assembly vectors can be used. By assembling a fragment into different assembly vectors, the same assembled fragment can be used flexibly in complex variants of assemblies, without any change having to be made in the fragment itself.

**Figure 8.**
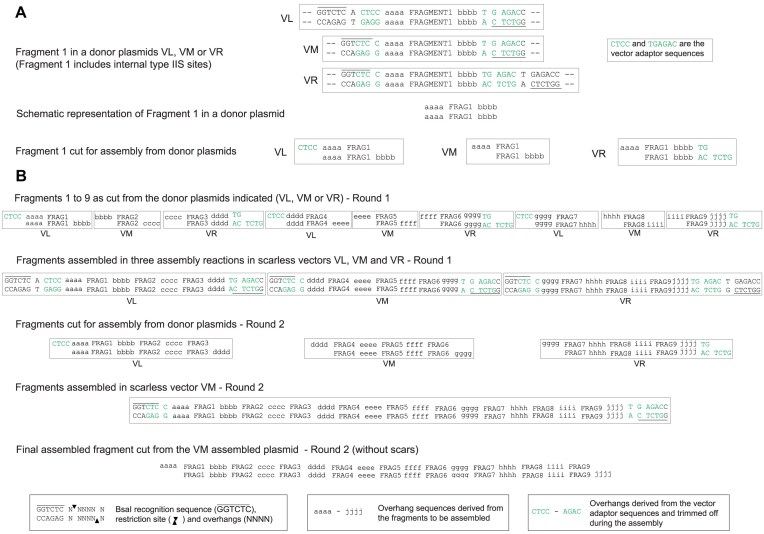
General scheme for scarless hierarchical DNA assembly of fragments with internal type IIS restriction sites using UniClo. (**A**) Illustration of how a fragment, Fragment 1, would be cut from a donor plasmid of VL, VM, or VR type. The fragments have the vector adaptors sequences CCTT and TGAGAC required for assembly using the scarless three-vector set. The four letters aaaa and bbbb represent overhangs that could be generated from within the sequence of Fragment 1. CTCC and AGAC are the overhangs generated from the vector adaptor sequences and are the required overhangs for the end of an assembled fragment to be compatible with the overhangs in the assembly vector (whether VL, VM, or VR type). (**B**) Schematic representation of two rounds of assembly of nine fragments using BsaI. In the first round, the fragments are assembled in groups (three in this example, but more can be used) in assembly vectors VL, VM, and VR. Using this scarless three-vector set, the overhangs of the vector adaptor sequences are trimmed off as appropriate according to the vector type used. In the second round, these assembled fragments are further assembled into the final assembled fragment. The vector adaptor sequences from the first round of assembly are trimmed off at both ends generating a fully scarless assembled fragment. The vector adaptor sequences are removed in each round of the assembly. This assembly design can be used for fragments with internal BsaI sites as these will be methylated by a recombinant nonswitchable methylases, while the outer BsaI sites are protected from methylation by recombinant MP-dCas9–sgRNA.

The use of methylation-protection to assemble DNA fragments which have internal sites for the restriction enzyme used in the assembly can be combined with the set of trimming vectors to offer DNA assembly without sequence constraint and without scar formation. We term this universal assembly approach “UniClo,” and Fig. 8 provides a schematic overview of a scarless hierarchical UniClo assembly of nine hypothetical fragments. Each round assembles fragments using a VL plasmid at the left end of the assembly, a VR plasmid at the right end of the assembly and VM plasmids in between these.

### Scarless hierarchical DNA assembly of fragments with internal type IIS restriction sites using methylases and methylation-protection

Scarless hierarchical assembly using UniClo was demonstrated experimentally by the assembly of 11 DNA fragments of around −1 kb each over two rounds of assembly (Supplementary Fig. S21). In total, the 11 fragments contained 39 internal BsaI sites with multiple BsaI sites in each fragment. The donor plasmids (POC1535–POC1545) containing these fragments were created by DNA synthesis with the fragments flanked by unmethylated outer methylation-protectable BsaI sites and containing multiple always-methylatable internal BsaI sites.

Before the first round of the assembly, the recombinant switch methylase M.Osp807II was used to methylate and so inactivate the outer BsaI sites in the kanamycin-resistant assembly vectors POC1518 (VL), POC1519 (VM), and POC1520 (VR). For the chloramphenicol-resistant donor plasmids, the recombinant nonswitchable methylase M2.BsaI (Supplementary Table S3) was used to methylate and so inactivate the internal BsaI sites in the fragments to be assembled. This methylation was performed in the presence of recombinant MP-dCas9–sgRNA, which protects the flanking outer BsaI sites from methylation, so these sites remain active.

In the first round of assembly, the 11 fragments were assembled in four groups (Supplementary Table S7). The fragments 1_1 to 1_4 were assembled in the VL assembly vector POC1518 to make the assembled plasmid POC1546, 1_5 to 1_7 in the VM assembly vector POC1519 to make POC1547, 1_8 and1_I9 in the VM assembly vector POC1519 to make POC1548, and 1_10 and 1_11 in the VR assembly vector POC1520 to make POC1549. During these assembly reactions, the assembly vectors were cut from their inner always-cuttable BsaI sites releasing the stuffer insert and generating overhangs CTCC and AGAC. The fragments in the donor plasmids were cut from their unmethylated outer methylation-protectable BsaI sites.

The assembled plasmids POC1546, POC1547, POC1548, and POC1549 contained the assembled fragments (2_1, 3.5 kb; 2_2, 3.5 kb; 3_3, 1.5 kb; and 4_4, 2.3 kb, respectively) flanked by the outer methylation-switchable BsaI sites of the assembly vectors. The assembled plasmids each should have two NotI sites, one beyond each of the outer BsaI sites (Supplementary Fig. S22). NotI digestion of all the assembled plasmids showed a band of 6.3 kb corresponding to the plasmid backbone and bands of 3.5, 3.5, 1.5, and 2.3 kb corresponding to the assembled fragments 2_1, 2_2, 2_3, and 2_4, respectively (Supplementary Fig. S23). All the internal BsaI sites remained within the fragments. The restriction digestion using NotI demonstrated a success rate of 100% for these assemblies since all plasmids analyzed from white colonies contained the correctly assembled fragment (Supplementary Table S6). As the outer methylation-switchable sites of the assembly vectors are also methylation-protectable, the assembled plasmids from the first round of assembly were used as new donor plasmids in a subsequent second round of assembly (Supplementary Table S7).

The second round of the assembly was performed in a similar manner. Prior to the assembly, recombinant switch methylase M.Osp807II was used to methylate and inactive the outer BsaI sites in the chloramphenicol-resistant assembly vector POC1525 (VM). For the new kanamycin-resistant donor plasmids (POC1546, POC1547, POC1548, and POC1549), recombinant M2.BsaI methylase was used to methylate and so inactivate the internal BsaI sites in the fragments to be assembled, with recombinant MP-dCas9–sgRNA present to protect the flanking outer BsaI sites from methylation.

The four previously assembled fragments were assembled in the VM assembly vector to make the plasmid POC1550 which contained the final assembled fragment 3_1. During the assembly reaction, the assembly vector was cut from its inner always-cuttable BsaI sites generating overhangs CTCC and AGAC. The new donor plasmids were cut from their unmethylated outer BsaI sites which are both methylation-protectable and methylation-switchable. The fragments released from the donor plasmids had scarless trimmed overhangs at their left or right or both sides depending on whether they were being cut from a VL or VR or VM, respectively.

The final assembled plasmid POC1550 should have two DraIII and two NotI sites, with one of each beyond each of the outer BsaI sites (Supplementary Fig. S24). Restriction digestion of the assembled plasmid with either DraIII or NotI showed a band of 6.3 kb corresponding to the plasmid backbone and a band of 10.8 kb corresponding to the assembled fragment 3_1 (Supplementary Fig. S25). In this assembly, a success rate of 83% was obtained since 5 of the 6 plasmids tested from white colonies contained the correctly assembled fragment (Supplementary Table S6). Long-range (Nanopore) sequencing confirmed the presence of the correctly assembled fragment 3_1, including the presence of the 39 internal BsaI sites (Supplementary File S2). This assembled plasmid could be used as a donor plasmid in a further round if necessary. The success rate of the assemblies was over 80% in all cases (Supplementary Table S6).

**Figure 9.**
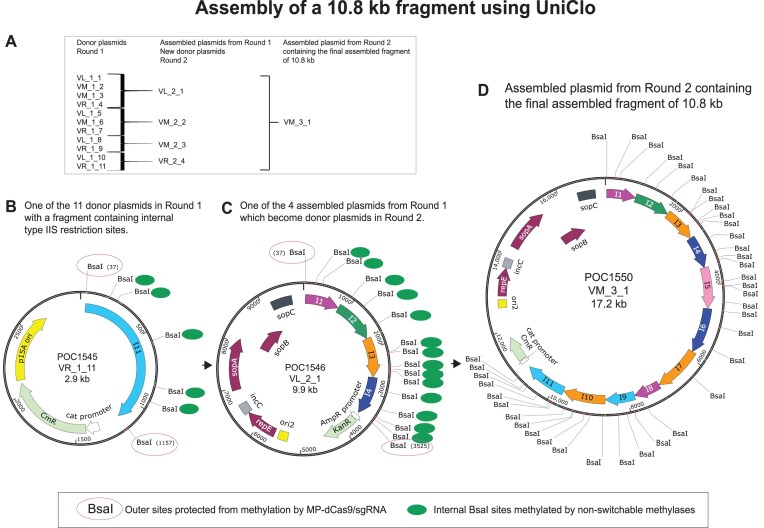
Scarless hierarchical assembly of a 10.8 kb DNA fragment containing 39 internal BsaI sites using UniClo. (**A**) Scheme of the two-round assembly. In the first round, 11 fragments, each in a donor plasmid, were assembled in groups of 4, 3, 2, and 2 resulting in four assembled plasmids. In the second round, these four assembled plasmids were used as donor plasmids for the assembly of the final assembled fragment of 10.8 kb. (**B**) An example of one of the 11 donor plasmids from the first round. POC1545 containing the fragment 1_11 flanked by the outer methylation-protectable BsaI sites (purple circles) and including multiple internal always-methylatable BsaI sites (green filled circles). The fragment 1_11 is in a VR plasmid because it forms the right end in the first round of assembly. (**C**) An example of one of the four assembled plasmids from the first round which are also used as donor plasmids in the second round. POC1456 contains the assembled fragment 2_1 without any unwanted scars between the four fragments assembled in the first round. The assembled fragment 2_1 is flanked by outer BsaI sites which are methylation-protectable, allowing it to be cut from the plasmid in the second round of the assembly. The fragment 2_1 was assembled in a VL vector because it forms the left end of the next round of the assembly. (**D**) The assembled plasmid POC1550 from the second round of the assembly containing the assembled fragment 3_1 (10.8 kb) without scars. The assembled fragment 3_1 was assembled in a VM vector because it is the final assembled fragment and cutting by BsaI will trim the vector adaptor sequences off both ends generating the desired assembled fragment fully free of scars. MP-dCas9–sgRNA: methylation-protection dCas9–sgRNA molecule. I1–I11 represent fragments 1 to 11.

A completely scarless hierarchical assembly of a 10.8 kb fragment containing multiple internal type IIS sites was achieved (Fig. 9). This was possible because the construction of the scarless assembly vectors (VL, VM, and VR) is such that cutting from the outer BsaI restriction sites results in trimming of the released fragment at one or both ends depending on which assembly vector is used. The use of these trimming assembly vectors requires only two universal vector adaptor sequences CCTT and TGAGAC, which generate the overhangs CTCC and AGAC at the left and right ends of the assembled fragment, respectively. When the overhangs of the assembled fragment anneal with the assembly vector, the vector adaptor sequences regenerate the trimmed outer BsaI sites in the VL, VR, or VM assembly vectors such that cutting from this outer BsaI site in the next round of assembly removes from the excised fragment the vector adaptor sequences which would otherwise form unwanted scars. In each round of an assembly, the vector adaptor sequences are trimmed off the assembled fragment and remain attached to the assembly vectors backbone rather than to the assembled fragment.

## Discussion

### Overview/synopsis

DNA assembly has a key role in synthetic and molecular biology in the generation of customized long DNA constructs for applications in fields including biotechnology, agriculture, medicine, and environmental remediation [6, 35]. Desirable features of a DNA assembly technique include simplicity, universality with respect to sequence, flexibility, and efficiency in the generation of long DNA constructs without scars [36, 37]. We have developed UniClo, a universal DNA assembly approach that builds on MetClo which is a variant of the type IIS restriction enzyme-based MoClo/Golden Gate method. We have previously studied the properties of recombinant switch and nonswitchable methylases [19], and UniClo exploits these in combination with a recombinant CRISPR–dCas9 molecule to permit scarless hierarchical modular assembly of DNA fragments with internal sites for the type IIS enzyme used in the assembly (Fig. 1). The methylases used were BsaI-associated methylases, and the assembly vectors and donor plasmids were designed to contain appropriately engineered methylase recognition sequences. Recombinant M.Osp807II switch methylase was used to methylate and so inactivate the methylation-switchable outer BsaI sites in the assembly vectors. These outer sites can be demethylated by transformation and propagation of the plasmid in a standard laboratory *E. coli* strain which does not express the methylase; demethylated plasmid can then be used as a donor in a subsequent round of assembly using BsaI. The assembly of DNA fragments containing internal BsaI sites was possible by methylating these sites with recombinant nonswitchable methylases (M2.Eco31I or M2.BsaI) in the presence of a recombinant CRISPR–dCas9 molecule appropriately RNA-guided to sterically block access of the methylase to the outer BsaI sites in donor plasmids. Engineering of the distance between the inner and outer BsaI sites in the design of the assembly vectors allows trimming of the assembled DNA fragments in each round to remove unwanted scars.

### Engineering of the outer flanking type IIS restriction enzyme site

UniClo relies upon recombinant methylases, a recombinant sgRNA-guided CRISPR–dCas9 molecule and engineering of the outer flanking BsaI sites to create the desired properties for universal DNA assembly. The set of recombinant methylases has been described previously [19]. The use of CRISPR–dCas9 molecules to sterically block access to DNA is well-established [21, 29, 30], and we used recombinant dCas9 and a suitably designed guide RNA to target blockade to the outer flanking BsaI site. UniClo is based on MoClo, with engineering of the outer insert-flanking type IIS restriction enzyme recognition site to achieve three aims. The first modification of this outer site provides an overlapping recognition site for a switch methylase and this forms the basis of MetClo [10]. The engineered outer site can be methylated and so inactivated, which means that only one type IIS enzyme is required, even across multiple rounds of hierarchical assemblies. In UniClo, we have now built further on the MoClo design by further engineering the outer sites in two further ways. The second modification of the outer sites allows the assembly of fragments containing internal BsaI sites—this is achieved by altering the outer BsaI sites to include a target sequence and PAM sequence to guide a CRISPR–dCas9 molecule to bind to the sites and so prevent them from being methylated by the nonswitchable methylase M2.BsaI, which can still methylate and so block the internal BsaI sites. The third modification of the outer sites allows the removal of unwanted vector adaptor overhang scars—this is achieved by moving the outer sites nearer to the overhang sequence used in the assembly, so that in any subsequent assembly round cutting by BsaI from these outer sites trims the unwanted vector adaptor overhangs off the previously assembled fragment that is then being released for assembly in that round. We have exemplified UniClo using BsaI, but the same principles could be applied to develop this approach for use with other type IIS restriction enzymes where suitable methylases are identifiable.

### Methylation-protection using CRISPR–dCas9

In standard MoClo, DNA fragments cannot be assembled if they contain internal sites for one of the type IIS restriction enzymes used during the assembly because the fragments will be cut at these sites. The bacterial restriction-modification system uses methylation of restriction enzyme sites to inhibit cutting at these sites by the relevant restriction enzyme. Therefore, methylation of restriction enzyme recognition sites within DNA fragments to be assembled could prevent digestion of these fragments during the assembly. However, the outer restriction enzyme sites flanking the fragments to be assembled must not be inhibited because these sites must be cut during the assembly to release the fragment. Access to DNA by proteins, such as restriction enzymes or transcription factors, can be sterically blocked by DNA-binding molecules [21, 29, 30]. The use of a CRISPR–dCas9 molecule guided with high specificity by a long sgRNA is an established method of blocking access of enzymes to specific DNA sequences [21]. We demonstrate that this approach can be used to block and so protect BsaI sites from methylation by recombinant site-specific methylases, thus allowing DNA assembly of fragments containing internal BsaI sites. This technique offers great flexibility as the DNA target sequence and sgRNA sequence can be modified as needed [26] to block the actions of different methylases on their associated restriction enzyme sites. The dCas9–sgRNA target sequence in any assembly vectors or donor plasmids should include the methylase recognition sequences, and the PAM sequence should be within the recognition sequence of the type IIS restriction enzyme (Fig. 2). Thus, the technique could be expanded for use with other type IIS restriction enzymes used in DNA assembly such as BpiI and LguI.

### Scarless assembly using trimming

In a standard multi-round MoClo assembly, overhangs at the ends of each assembled fragment in each round must be designed for compatibility with particular assembly vectors and these overhangs become incorporated as short scars between the assembled fragments in the next round (Fig. 3). Considerable numbers of vectors can be required for multi-round assemblies [8, 11]. Refinements have included the design of vector overhang scars to be made up of sequences, such as start and stop codons [3]. Type IIS enzymes used in MoClo assemblies cut DNA at a defined distance from the enzyme recognition site. In an standard assembly vector, the vector overhang is produced by a type IIS enzyme which recognizes the inner recognition site, for BsaI the vector overhang will be 11 bp from this site (Fig. 4). The position of the overhang produced when an assembled fragment is cut out for the next round of assembly is determined by the outer type IIS recognition site. Therefore, by moving an outer BsaI site nearer than 11 bp to the vector overhang used during the first round of the assembly, cutting from the outer site will result in trimming of that overhang sequence off the assembled fragment, leaving it attached to the plasmid backbone. This can be done at either end of the assembled fragment, and a set of three assembly vectors was generated that resulted in trimming at either the left or right end of the fragment or at both ends (Figs 5–7). The trimming assembly vector VL trims only the right end and so can be used to assemble a fragment that will be at the left end in the next round of an assembly. Reciprocally, the trimming assembly vector VR trims only the left end and so can be used to assemble a fragment that will be at the right end in the next round of an assembly. The trimming assembly vector VM trims at both ends and so can be used to assemble “middle” fragments that will not be at either end in the next round of assembly. Only these 3 vectors are needed for any assembly.

The three vectors all use the same overhangs of CTCC at the left end and AGAC at the right end, so that any assembled fragment with these overhangs could be assembled into each of the three assembly vectors and cut out with one or more ends trimmed to remove these overhangs if required. The design of the trimming allows reconstitution of the BsaI sites and it is such that left-end trimming results in the removal of just the 4 bp overhang CTCC and right-end trimming results in the removal of 6 bp, TGAGAC, which includes the overhang AGAC. Therefore, in the design of a scarless assembly, the sequences CTCC at the left end and TGAGAC at the right end serve as vector adaptor sequences that are added to the ends of the sequence that is wanted and are subsequently removed by this trimming process when no longer required. In an analogous manner, Chen *et al.* [16] used ligated adaptors to control the distance between the recognition site for the type IIS enzyme MspJI and the position of the resulting overhang within the fragment, so permitting the generation of cohesive ends that do not contain unwanted scar material.

### Exemplification of scarless hierarchical assembly of DNA with internal type IIS sites using UniClo

UniClo allows hierarchical scarless DNA assemblies to be designed with great flexibility because the overhangs are the same with each of the three vectors (Figs 5–8). This also allows easy reuse of fragments in new assemblies. We demonstrate the scarless hierarchical assembly of a 10.8 kb fragment by UniClo using our recombinant methylases, methylation-protection approach and the set of three trimming vectors (VL, VM, and VR) (Fig. 9). In the first round of assembly, four plasmids containing assembled fragments from 1.5 to 3.5 kb with multiple internal BsaI sites were assembled. The outer BsaI sites flanking these the fragments were protected from methylation by the MP-dCas9–sgRNA and the internal BsaI sites were methylated and so inactivated by the nonswitchable methylase M2.BsaI. The assembled plasmids from the first round were used as new donor plasmids in the second round to assemble the final fragment of 10.8 kb in a VM plasmid without any unwanted scars. UniClo makes possible the scarless assembly of fragments containing multiple internal type IIS restriction sites.

### Other DNA assembly techniques and the use of methylases in DNA assembly

A number of DNA assembly techniques have been described in recent years, each with its own advantages and drawbacks as summarized in Supplementary Table S8 [3, 4, 8–10, 15–17, 36, 38, 39]. Most of them use type IIS or type IIP restriction enzymes or both. PlasmidMaker, uses *Pyrococcus furiosus* Argonaute (*Pf*Ago)-based artificial restriction enzymes (AREs) for the assembly. These argonaute-based AREs are guided by custom-designed ssDNA molecules towards specific DNA sequences, where digestion generates the compatible overhangs required for the assembly [38]. The reported DNA assembly techniques have permitted important achievements in this field, such as the long DNA fragments assembly (up to 218 kb) [8, 10, 16, 17, 36], the assembly of multiple linear or circular fragments in a one-pot reaction [9, 10, 36], and the scarless assembly of expression units due to the use of start and stop codons as junctions between transcriptional units [3]. Among the drawbacks of these techniques are the multiple assembly vectors and adaptors required for a hierarchical assembly [3, 8–10] and the great number of primers used for the assembly of PCR-amplified DNA [4, 36, 38, 39]. A key drawback is unwanted scar formation as a result of the joining adaptors used [8, 9, 15–17, 35] and the unfeasibility of the assembly of fragments containing internal restriction sites without domestication. Some techniques have avoided domestication by using oligos, panels of multiple argonaute-based AREs or methylation [9, 16, 17, 38].

Methylases have been used previously in DNA assembly [9, 14–18, 35]. In pairwise selection assembly (PSA), a methylase was used to methylate internal restriction sites in the fragments to be assembled, and oligonucleotides to block methylation at the restriction sites required for the assembly [17]. MASTER ligation uses a modification-dependent endonuclease which only cuts methylated sequences. Methylation is introduced in the franking sites of the fragments before the assembly. During the assembly, the endonuclease only cuts these methylated flanking sites, leaving the unmethylated internal sites uncut [16]. In 2ab assembly, only two fragments can be assembled. One donor plasmid is grown in a bacterial strain expressing a methylase that blocks BamHI sites and the other donor plasmid is grown in a strain expressing a methylase that blocks BglII sites [15]. Further applications of 2ab assembly using more type IIP enzymes were developed by Matsumura *et al.* [14, 35]. The TNT cloning system uses a methylase to inhibit cutting by type IIS restriction enzymes and oligonucleotides to block internal enzyme recognition sites in the fragments to be assembled. However, these oligonucleotides have to be custom-designed according to the sequence for each fragment [9]. Overall, these assembly techniques using methylases have potential drawbacks and complexities and no simple approach to the hierarchical scarless assembly of fragments containing internal restriction sites has been developed [4, 37, 39–43].

To the best of our knowledge, UniClo is the first simple universally applicable method for hierarchical scarless assembly of long DNA fragments containing internal sites for the type IIS enzyme restriction enzyme used in the assembly. These major advantages arise from the combined use of recombinant methylases, CRISPR–dCas9 technology, a scarless three-vector set and only two joining adaptors. A potential limitation is the need for recombinant methylases which are not currently available commercially, but are relatively easy to produce without specialist equipment [19].

### Final conclusions

In conclusion, our work provides a framework for hierarchical modular and scarless assembly using recombinant switch and nonswitchable methylases in association with a recombinant CRISPR–dCas9 molecule. The *in vitro* reactions using the recombinant enzymes allow precisely controlled targeted methylation and methylation-protection as required in assembly vectors and donor plasmids. In addition, the dCas9–gsRNA system developed for the targeted methylation-protection of the fragments’ flanking sites is based on steric hindrance and does not require additional enzymes or chemicals. UniClo allows hierarchical assembly without scar formation using a set of only three assembly vectors and is simple and flexible in its application. This DNA assembly technique could be applied with suitable methylases compatible with other type IIS restriction enzymes such as BpiI and LguI by designing sgRNAs and assembly vectors that include their recognition sequences. Our approach allows the assembly of a broad range of engineered long DNA constructs for diverse applications.

## Supplementary Material

gkaf548_Supplemental_Files

## Data Availability

All the plasmids from this study have been deposited with Addgene.

## Notes

Present address: Triple Helix Biotechnology Ltd., Moneta Building (B280), Babraham Research Campus, Babraham, CB22 3AT, United Kingdom

Present address: Complete Regulatory, 19-20 King Edward Street, Macclesfield, SK10 1AQ, United Kingdom
