# Supplementary material for: UniClo: scarless hierarchical DNA assembly without sequence constraint
Source: Nucleic Acids Res. 2025 Jun 23;53(12):gkaf548. doi: 10.1093/nar/gkaf548 (PMC12205989; doi:10.1093/nar/gkaf548)
Supplement: gkaf548_Supplemental_Files [file gkaf548_supplemental_files.zip › Supplementary File S1 POC1431 sequences from five colonies.docx]

# POC1431 sequences from five white colonies using the methylases M.Osp807II and M2.Eco31I

## Colony 1

Forward CO9566: TGGTGTAAACAAATTGACGC

NNNNNNNNNNNNNGGACGTTTTTATGNACTGGGGTGGATGCAGTGGGCCCCACTCTGTGGTGCAGTACCTCTCACGACTTGGTCTCACGTTGTGGGGCTTAGCTTCATATTTTCAAACTGAAATATTCTCTTCCTTAACCTCCACATAAATCCAAGTTTATAATTTTTATTATTTTAAAATTTTATTTATTTTTCTGTTTTGGGGACAGGGTCTCCTCCTGTCACTCAGGCTGGAGTGCAATGGCACAATCATAGCTCACTGCAGCCTGGAACTCCTGGGCTTAAGCAATCTTCCTGCCTTCGATTCCCAAAGAGCTGGGATTATAGTCATGAACCACTGCAATCCACCCAAATCCAAGTTTACACTAAAAGATAAAATTCCAACATTGTAGGGGATTGGTCAGGTGGTGGGAATAATTATAAAGATAAAGTTATAGGAAATAGACACAAACCTTCTTGGAAGGTGGAAAGTTTTGCAAAAGCCTCAGGATAGGGTTATAGCTGAAAGCAGCCTAATCCCCTTACCTTGAGTTAATAGCTTCGAGTAAGTACAAAGACATGTAAGAGAGTTTATCTAAAGAGCATGTTTACCTTTGATCATTTGTAGGACTGCTCTCTCTGGGGGACTGCGACCAGATTAATTACCCACAGGTGTGTTGACTCAAAGCCTTTGTCATTAAATCTGTGCTGAATAAAGGCCCACAGGGCCAGATAGTCAGGGCACGCAGCTGCCACAACCCTTTCTGTGAGTGGCCTGGCCCTCTGGTGCACTCTTTCACTGAATATCGGTGTCTGAGTACATTATTCATCCATCGTGCAGCCTGGGTCTGCCGGTCAGACCCTGGCACAACATTTAAGAGGAAATGAAAGTCACAAAGTTATCCCAGTCTCTGGAGTCACTGTCAAAACTTTGGTGAGGAATCTTCCAGGTTTTCCCCTACTTCAAATATATATTAATATTATGTAAGTGATATTAGTGGCATTTTCGCCCAGGCTGGA

Forward CO9574: AGAAATAATGAAACTACGTC

NNNNNNNTCNNNNGGNGCGGNGGCTCACGCCTGTAATCCCAGCACTTTGAGAGGCCAAGGCAGGCAGATCACGAGGTCAGGAGATCGAGACCATCCTGGCTAACACAGTGAAACCCCGTCTCTACTAAAAATACAAAAAATTAGTCGGGCGTGGTGGCAGGAGCCTGTAGTCCCAGCTACTCCAGAGGCTGAGGCAGCAGAATGCCCTGAACCCGGGAGGCGGAGCTTGCAGTGAGCCAAGATCGCGCCACTGCACTCCAGCCTGGGCGACAGAGCAAGACTCCGTCTCAAAAAAAAAAAACAAAAAACAAAAAAACTCTCCTTTACTTTTTCTCTCCCCTTTTCTTCCTATCTCTTCCCTCATTTCTTCAACACGTCCCCCCATCCTTCCCTCTTTTCTCCATTCTCTGCATTTGATCCCCGGTATATTCCAGCCTCCAGGCCAACAAACTTCTCCGCGTCCGCCGGGAGCAGGTCAGGGAAGGGACGCGAGGCGGCGCTGTCACCGCATTCTGAGCGCCGCAGCTCCCTGGGCCCCTTGTATCATTTCAGTGAAGGTCACTCCAGTCTTTCATGGAGGCCAAACTAAGGGTGTAAATTAGGATCCTCACTGAAGTGGCGGGACCCTAAGAGGCTTTTTCCTGGCCCCTTAGTTGTGGGTTTTCCTGCGGGCGGCGCAGCCGGTTTCCATCAGAACCGCCCAGAGGCGGACGCTGCCTTCCTGGGGTGACGGAGCAGCAGGAAGCGTTTTCGGATCCTGGAATACGTGGGCGGCCCGTGGGAGGGGCTGAGGCGCAGTTTCCTACTCACCCGGATCCGAATCCTCCGCGGTGCTGTTTCAAGAGAGCCGGATTCCAGATCGCGCTCCAGCCCGGACTCGGAATTCCTGCCCTGCGGGTCTGCATTTTCATAACGGGCAGGTGTGAGTGCCCTGCAGCTGGAGACCAGAAGCCTGAAGGCAGCTCGGCCCTCCCCAGCCCACAGCGCCGTTATTCCGTTTCTATATCAGTAAACACATTTCATTTTCCGTAGACCAGGGCGGGGTGACGGGTGATCCCAGTCCTCGCAGTGAATTCNGGGCAGCAAAATTCAAAACACATGCGGCCAAGGCCGGGCACGGNGNTTCACGCCTGTATTCCAGCACTTTGGGAGGTCGAGGTGGNCGATCACCTGNNGTCGGNAGTNCANANNAACCTGACCACCA

Reverse CO9567: ACGCCCTTTTAAATATCCG

NNNNNNNNNTTCTCTTAGGTTTACCCGCCAATATATCCTGTCAAACACTGATAGTTTAAACCACTTCGTGGTGCAGTACCTCTCACGACTTGGTCTCACCTGACACAGGTCCCAAGCCCTTACCTCCACGGTGTCTACCCTCCCTCCAGGACTTCCTCCGTGTGCCAGCTCCAGCAAAGGATCTGATTCAGCTCGCCCCCAAAAAAGACTTTTAATAGTTCAATAACAATAATGAATATGCAAGGTTTGTTCTAAGGCATTTAGAAATGGTTTCAGGGAGTCATGAAGCCAGTCCTCTCTTGGGCTAGGGGAGGCCGAGATGGTCTTGAGCTCCAGGGGACTTGTTTCTTAGTGCCCAGGCCTGGGCGCCCCTCCCCCACCAAGCCTCCCAGGTCTTCTGTCCAAAGCCCTCCCCCTCCACCCCACCTCCAACCCCGTCTGCTCTACCCCATCAACTACGTTTTCTCCCTCAGCACTTGCCTTATACCCCATGCACTCACGAGCACAGAGGCGACTTCCCTCTCTCAGACTTTAGGCGCCACTGCAGGGTCCGGAAAAGAAAGAGAAACGGCCCAGCGCGGTCGCTTACATAACCCAGGGCGGGGCTCCGCTCCGCCCCCGAAAGTTTTTGCGACGGAGTTTTCCCTCTTGTTGCCCAGGCTGAAGTGCAATGGCGCGATCTCGGCTCACCGCAACCTCCGCCTCCAAGGTTTAAGCGATTCTCCTGCCTCAGCCTCCCGACTAGCTGGGATTACAGGCATTCACCACCAAGCCCGTCTAATTTTATATTTTTAGTAGAGACGGGATTTCCCCATGTTGGTCAGGTTGGTCTCGAGCTCCCGACCTCAGGTGATCGCCCGCCTCGACCTCCCAAAGTGCTGGGATTACAGGCGTGAACCACCGTGCCCGGCCTTGGCCGCATGTGTTTTGAATTTTGCTGCCCGGAATTCACTGCGAGGACTGGGATCACCCGTCACCCCGCCCTGGTCTACGGAAAATGAAATGTGTTTACTGATATAGAAACGGAATAACGGCGCTGTGGGCTGGGGAGGGCCGAGCTGCCTTCAGGCTTCTGGTCTCCAGCTGCAGGGCACTCACACCTGCCCGTTATGAAAATGCAGACCCGCAGGGCAGGAATTCCGAGTCCGGGCTGGAGCGCGATCTGGAATCCGGCTCTCTTGAAACAGCACCGNGGAGGATTCGGATCCGGNTGANTAGGAAACTGCGCCTCAGCCCCTCCCACGGGCCGCCCACGTATTCCAGGATCCGAAAACGCTTCCTGCTGCTCCGTNACCCCAGGAAGGCAGCGTCCGCCTCTGGGNGGTTCTGATGAAAACCGGCTGNNCNCCCNNAGGAAAACCCACAACTAAGGGGCCAGAAAAAGCCTCTTAGGGTCCNNCACTTCAGGAGGATCCTAATTNACACCTTNNNTTGGNCCCCTNNAAANNNNGNANGGCCTTNNNNNNAANNAAAAANNGGGNCNNNNNNNANNNNGGGGNNNNANAANNNNNGNANNNNNCCGCNNNNNNNNCNNNNNNNNNNCNNNTNNNCCNNNGNNNNNNNNNAAAAATTTNNNNNNCNNNNNTNNGGNNNNNCC

Reverse Complement Reverse CO9567: ACGCCCTTTTAAATATCCG

CGGTGCTGTTTCAAGAGAGCCGGATTCCAGATCGCGCTCCAGCCCGGACTCGGAATTCCTGCCCTGCGGGTCTGCATTTTCATAACGGGCAGGTGTGAGTGCCCTGCAGCTGGAGACCAGAAGCCTGAAGGCAGCTCGGCCCTCCCCAGCCCACAGCGCCGTTATTCCGTTTCTATATCAGTAAACACATTTCATTTTCCGTAGACCAGGGCGGGGTGACGGGTGATCCCAGTCCTCGCAGTGAATTCCGGGCAGCAAAATTCAAAACACATGCGGCCAAGGCCGGGCACGGTGGTTCACGCCTGTAATCCCAGCACTTTGGGAGGTCGAGGCGGGCGATCACCTGAGGTCGGGAGCTCGAGACCAACCTGACCAACATGGGGAAATCCCGTCTCTACTAAAAATATAAAATTAGACGGGCTTGGTGGTGAATGCCTGTAATCCCAGCTAGTCGGGAGGCTGAGGCAGGAGAATCGCTTAAACCTTGGAGGCGGAGGTTGCGGTGAGCCGAGATCGCGCCATTGCACTTCAGCCTGGGCAACAAGAGGGAAAACTCCGTCGCAAAAACTTTCGGGGGCGGAGCGGAGCCCCGCCCTGGGTTATGTAAGCGACCGCGCTGGGCCGTTTCTCTTTCTTTTCCGGACCCTGCAGTGGCGCCTAAAGTCTGAGAGAGGGAAGTCGCCTCTGTGCTCGTGAGTGCATGGGGTATAAGGCAAGTGCTGAGGGAGAAAACGTAGTTGATGGGGTAGAGCAGACGGGGTTGGAGGTGGGGTGGAGGGGGAGGGCTTTGGACAGAAGACCTGGGAGGCTTGGTGGGGGAGGGGCGCCCAGGCCTGGGCACTAAGAAACAAGTCCCCTGGAGCTCAAGACCATCTCGGCCTCCCCTAGCCCAAGAGAGGACTGGCTTCATGACTCCCTGAAACCATTTCTAAATGCCTTAGAACAAACCTTGCATATTCATTATTGTTATTGAACTATTAAAAGTCTTTTTTGGGGGCGAGCTGAATCAGATCCTTTGCTGGAGCTGGCACACGGAGGAAGTCCTGGAGGGAGGGTAGACACCGTGGAGGTAAGGGCTTGGGACCTGTGTCAGGTGAGACCAAGTCGTGAGAGGTACTGCACCACGAAGTGGTTTAAACTATCAGTGTTTGACAGGATATATTGGCGGGTAAACCTAAGAGAA

**Assembled sequence**

Insert 1

ACTGGGGTGGATGCAGTGGGCCCCACTCTGTGGTGCAGTACCTCTCACGACTTGGTCTCACGTTGTGGGGCTTAGCTTCATATTTTCAAACTGAAATATTCTCTTCCTTAACCTCCACATAAATCCAAGTTTATAATTTTTATTATTTTAAAATTTTATTTATTTTTCTGTTTTGGGGACAGGGTCTCCTCCTGTCACTCAGGCTGGAGTGCAATGGCACAATCATAGCTCACTGCAGCCTGGAACTCCTGGGCTTAAGCAATCTTCCTGCCTTCGATTCCCAAAGAGCTGGGATTATAGTCATGAACCACTGCAATCCACCCAAATCCAAGTTTACACTAAAAGATAAAATTCCAACATTGTAGGGGATTGGTCAGGTGGTGGGAATAATTATAAAGATAAAGTTATAGGAAATAGACACAAACCTTCTTGGAAGGTGGAAAGTTTTGCAAAAGCCTCAGGATAGGGTTATAGCTGAAAGCAGCCTAATCCCCTTACCTTGAGTTAATAGCTTCGAGTAAGTACAAAGACATGTAAGAGAGTTTATCTAAAGAGCATGTTTACCTTTGATCATTTGTAGGACTGCTCTCTCTGGGGGACTGCGACCAGATTAATTACCCACAGGTGTGTTGACTCAAAGCCTTTGTCATTAAATCTGTGCTGAATAAAGGCCCACAGGGCCAGATAGTCAGGGCACGCAGCTGCCACAACCCTTTCTGTGAGTGGCCTGGCCCTCTGGTGCACTCTTTCACTGAATATCGGTGTCTGAGTACATTATTCATCCATCGTGCAGCCTGGGTCTGCCGGTCAGACCCTGGCACAACATTTAAGAGGAAATGAAAGTCACAAAGTTATCCCAGTCTCTGGAGTCACTGTCAAAACTTTGGTGAGGAATCTTCCAGGTTTTCCCCTACTTCAAATATATATTAATATTATGTAAGTGATATTAGTGGCATTTTCGCCCAGGCTGGA

Inserts 2, 3 and 4

GGCTCACGCCTGTAATCCCAGCACTTTGAGAGGCCAAGGCAGGCAGATCACGAGGTCAGGAGATCGAGACCATCCTGGCTAACACAGTGAAACCCCGTCTCTACTAAAAATACAAAAAATTAGTCGGGCGTGGTGGCAGGAGCCTGTAGTCCCAGCTACTCCAGAGGCTGAGGCAGCAGAATGCCCTGAACCCGGGAGGCGGAGCTTGCAGTGAGCCAAGATCGCGCCACTGCACTCCAGCCTGGGCGACAGAGCAAGACTCCGTCTCAAAAAAAAAAAACAAAAAACAAAAAAACTCTCCTTTACTTTTTCTCTCCCCTTTTCTTCCTATCTCTTCCCTCATTTCTTCAACACGTCCCCCCATCCTTCCCTCTTTTCTCCATTCTCTGCATTTGATCCCCGGTATATTCCAGCCTCCAGGCCAACAAACTTCTCCGCGTCCGCCGGGAGCAGGTCAGGGAAGGGACGCGAGGCGGCGCTGTCACCGCATTCTGAGCGCCGCAGCTCCCTGGGCCCCTTGTATCATTTCAGTGAAGGTCACTCCAGTCTTTCATGGAGGCCAAACTAAGGGTGTAAATTAGGATCCTCACTGAAGTGGCGGGACCCTAAGAGGCTTTTTCCTGGCCCCTTAGTTGTGGGTTTTCCTGCGGGCGGCGCAGCCGGTTTCCATCAGAACCGCCCAGAGGCGGACGCTGCCTTCCTGGGGTGACGGAGCAGCAGGAAGCGTTTTCGGATCCTGGAATACGTGGGCGGCCCGTGGGAGGGGCTGAGGCGCAGTTTCCTACTCACCCGGATCCGAATCCTCCGCGGTGCTGTTTCAAGAGAGCCGGATTCCAGATCGCGCTCCAGCCCGGACTCGGAATTCCTGCCCTGCGGGTCTGCATTTTCATAACGGGCAGGTGTGAGTGCCCTGCAGCTGGAGACCAGAAGCCTGAAGGCAGCTCGGCCCTCCCCAGCCCACAGCGCCGTTATTCCGTTTCTATATCAGTAAACACATTTCATTTTCCGTAGACCAGGGCGGGGTGACGGGTGATCCCAGTCCTCGCAGTGAATTCCGGGCAGCAAAATTCAAAACACATGCGGCCAAGGCCGGGCACGGTGGTTCACGCCTGTAATCCCAGCACTTTGGGAGGTCGAGGCGGGCGATCACCTGAGGTCGGGAGCTCGAGACCAACCTGACCAACATGGGGAAATCCCGTCTCTACTAAAAATATAAAATTAGACGGGCTTGGTGGTGAATGCCTGTAATCCCAGCTAGTCGGGAGGCTGAGGCAGGAGAATCGCTTAAACCTTGGAGGCGGAGGTTGCGGTGAGCCGAGATCGCGCCATTGCACTTCAGCCTGGGCAACAAGAGGGAAAACTCCGTCGCAAAAACTTTCGGGGGCGGAGCGGAGCCCCGCCCTGGGTTATGTAAGCGACCGCGCTGGGCCGTTTCTCTTTCTTTTCCGGACCCTGCAGTGGCGCCTAAAGTCTGAGAGAGGGAAGTCGCCTCTGTGCTCGTGAGTGCATGGGGTATAAGGCAAGTGCTGAGGGAGAAAACGTAGTTGATGGGGTAGAGCAGACGGGGTTGGAGGTGGGGTGGAGGGGGAGGGCTTTGGACAGAAGACCTGGGAGGCTTGGTGGGGGAGGGGCGCCCAGGCCTGGGCACTAAGAAACAAGTCCCCTGGAGCTCAAGACCATCTCGGCCTCCCCTAGCCCAAGAGAGGACTGGCTTCATGACTCCCTGAAACCATTTCTAAATGCCTTAGAACAAACCTTGCATATTCATTATTGTTATTGAACTATTAAAAGTCTTTTTTGGGGGCGAGCTGAATCAGATCCTTTGCTGGAGCTGGCACACGGAGGAAGTCCTGGAGGGAGGGTAGACACCGTGGAGGTAAGGGCTTGGGACCTGTGTCAGGTGAGACCAAGTCGTGAGAGGTACTGCACCACGAAGTGGTTTAAACTATCAGTGTTTGACAGGATATATTGGCGGGTAAACCTAAGAGAA

## Colony 2

Forward CO9566: TGGTGTAAACAAATTGACGC

NNNNNNNNNNNNCGGACGTTTTTATGTACTGGGGTGGATGCAGTGGGCCCCACTCTGTGGTGCAGTACCTCTCACGACTTGGTCTCACGTTGTGGGGCTTAGCTTCATATTTTCAAACTGAAATATTCTCTTCCTTAACCTCCACATAAATCCAAGTTTATAATTTTTATTATTTTAAAATTTTATTTATTTTTCTGTTTTGGGGACAGGGTCTCCTCCTGTCACTCAGGCTGGAGTGCAATGGCACAATCATAGCTCACTGCAGCCTGGAACTCCTGGGCTTAAGCAATCTTCCTGCCTTCGATTCCCAAAGAGCTGGGATTATAGTCATGAACCACTGCAATCCACCCAAATCCAAGTTTACACTAAAAGATAAAATTCCAACATTGTAGGGGATTGGTCAGGTGGTGGGAATAATTATAAAGATAAAGTTATAGGAAATAGACACAAACCTTCTTGGAAGGTGGAAAGTTTTGCAAAAGCCTCAGGATAGGGTTATAGCTGAAAGCAGCCTAATCCCCTTACCTTGAGTTAATAGCTTCGAGTAAGTACAAAGACATGTAAGAGAGTTTATCTAAAGAGCATGTTTACCTTTGATCATTTGTAGGACTGCTCTCTCTGGGGGACTGCGACCAGATTAATTACCCACAGGTGTGTTGACTCAAAGCCTTTGTCATTAAATCTGTGCTGAATAAAGGCCCACAGGGCCAGATAGTCAGGGCACGCAGCTGCCACAACCCTTTCTGTGAGTGGCCTGGCCCTCTGGTGCACTCTTTCACTGAATATCGGTGTCTGAGTACATTATTCATCCATCGTGCAGCCTGGGTCTGCCGGTCAGACCCTGGCACAACATTTAAGAGGAAATGAAAGTCACAAAGTTATCCCAGTCTCTGGAGTCACTGTCAAAACTTTGGTGAGGAATCTTCCAGGTTTTCCCCTACTTCAAATATATATTAATATTATGTAAGTGATATTAGTGGCATTTT

Forward CO9574: AGAAATAATGAAACTACGTC

NNNNNNNTCGGNNNNGNGCGGNGGCTCACGCCTGTAATCCCAGCACTTTGAGAGGCCAAGGCAGGCAGATCACGAGGTCAGGAGATCGAGACCATCCTGGCTAACACAGTGAAACCCCGTCTCTACTAAAAATACAAAAAATTAGTCGGGCGTGGTGGCAGGAGCCTGTAGTCCCAGCTACTCCAGAGGCTGAGGCAGCAGAATGCCCTGAACCCGGGAGGCGGAGCTTGCAGTGAGCCAAGATCGCGCCACTGCACTCCAGCCTGGGCGACAGAGCAAGACTCCGTCTCAAAAAAAAAAAACAAAAAACAAAAAAACTCTCCTTTACTTTTTCTCTCCCCTTTTCTTCCTATCTCTTCCCTCATTTCTTCAACACGTCCCCCCATCCTTCCCTCTTTTCTCCATTCTCTGCATTTGATCCCCGGTATATTCCAGCCTCCAGGCCAACAAACTTCTCCGCGTCCGCCGGGAGCAGGTCAGGGAAGGGACGCGAGGCGGCGCTGTCACCGCATTCTGAGCGCCGCAGCTCCCTGGGCCCCTTGTATCATTTCAGTGAAGGTCACTCCAGTCTTTCATGGAGGCCAAACTAAGGGTGTAAATTAGGATCCTCACTGAAGTGGCGGGACCCTAAGAGGCTTTTTCCTGGCCCCTTAGTTGTGGGTTTTCCTGCGGGCGGCGCAGCCGGTTTCCATCAGAACCGCCCAGAGGCGGACGCTGCCTTCCTGGGGTGACGGAGCAGCAGGAAGCGTTTTCGGATCCTGGAATACGTGGGCGGCCCGTGGGAGGGGCTGAGGCGCAGTTTCCTACTCACCCGGATCCGAATCCTCCGCGGTGCTGTTTCAAGAGAGCCGGATTCCAGATCGCGCTCCAGCCCGGACTCGGAATTCCTGCCCTGCGGGTCTGCATTTTCATAACGGGCAGGTGTGAGTGCCCTGCAGCTGGAGACCAGAAGCCTGAAGGCAGCTCGGCCCTCCCCAGCCCACAGCGCCGTTATTCCGTTTCTATATCAGTAAACACATTTCATTTTCCGTAGACCAGGGCGGGGTGACGGGTGATCCCAGTCCTCGCANNGAATTCCGGGCAGCAAAATTCAAAACACATGCGGCCAAGGCCGGGCACGGTGGTTCACGCCTGTAATCCCAGCACTTTGGGAGGTCGAGGCGGGCGATNNCCTGAGGTCGGGAGCTCGAGACAANCCTGACCACATGGGGAAATCCNNNTCTACTAAAAATATAAANTAGACGGNNNTGGGGGGGAAGGCNGGAATTCCAGTNANCCGGNNNNNGAGNNN

Reverse CO9567: ACGCCCTTTTAAATATCCG

NNNNNNNNNTCTCTTAGGTTTACCCGCCAATATATCCTGTCAAACACTGATAGTTTAAACCACTTCGTGGTGCAGTACCTCTCACGACTTGGTCTCACCTGACACAGGTCCCAAGCCCTTACCTCCACGGTGTCTACCCTCCCTCCAGGACTTCCTCCGTGTGCCAGCTCCAGCAAAGGATCTGATTCAGCTCGCCCCCAAAAAAGACTTTTAATAGTTCAATAACAATAATGAATATGCAAGGTTTGTTCTAAGGCATTTAGAAATGGTTTCAGGGAGTCATGAAGCCAGTCCTCTCTTGGGCTAGGGGAGGCCGAGATGGTCTTGAGCTCCAGGGGACTTGTTTCTTAGTGCCCAGGCCTGGGCGCCCCTCCCCCACCAAGCCTCCCAGGTCTTCTGTCCAAAGCCCTCCCCCTCCACCCCACCTCCAACCCCGTCTGCTCTACCCCATCAACTACGTTTTCTCCCTCAGCACTTGCCTTATACCCCATGCACTCACGAGCACAGAGGCGACTTCCCTCTCTCAGACTTTAGGCGCCACTGCAGGGTCCGGAAAAGAAAGAGAAACGGCCCAGCGCGGTCGCTTACATAACCCAGGGCGGGGCTCCGCTCCGCCCCCGAAAGTTTTTGCGACGGAGTTTTCCCTCTTGTTGCCCAGGCTGAAGTGCAATGGCGCGATCTCGGCTCACCGCAACCTCCGCCTCCAAGGTTTAAGCGATTCTCCTGCCTCAGCCTCCCGACTAGCTGGGATTACAGGCATTCACCACCAAGCCCGTCTAATTTTATATTTTTAGTAGAGACGGGATTTCCCCATGTTGGTCAGGTTGGTCTCGAGCTCCCGACCTCAGGTGATCGCCCGCCTCGACCTCCCAAAGTGCTGGGATTACAGGCGTGAACCACCGTGCCCGGCCTTGGCCGCATGTGTTTTGAATTTTGCTGCCCGGAATTCACTGCGAGGACTGGGATCACCCGTCACCCCGCCCTGGTCTACGGAAAATGAAATGTGTTTACTGATATAGAAACGGAATAACGGCGCTGTGGGCTGGGGAGGGCCGAGCTGCCTTCAGGCTTCTGGTCTCCAGCTGCAGGGCACTCACACCTGCCCGTTATGAAAATGCAGACCCGCAGGGCAGGAATTCCGAGTCCGGGCTGGAGCGCGATCTGGAATCCGGCTCTCTTGAAACAGCACCGCGGAGGATTCGGATCCGGGTGAGTAGGAAACTGCGCCTCAGCCCNTCCNGGGNCGCCACGTATTCCAGGATCCGAAAACGCTTCCTGCTGCTCGTCCCCCAGAAAGCAACGTCCGCCTCTGGNNGGTCTGATGGAANNCGNCTGNGCNNCCGCAGGAAANCCACAACTAANGGCCAGGAAANNNCCNTTAAGGNCCGCCNTTNNGGNAGATCTAATTTACCCCTTAGTTGGCCTCCTGAAAGATGGAAGGACCTTCCNNAAATGATCAAGGGNCCNAGGANNNGGGNCTTCAANNNNGGGNNNNNCCCCCCNNNNNNNNNNNNNNNNNCCNGGAANNNNAAATTTNNNNNGNNNNTGAANTNNNNGNAA

Reverse Complement Reverse CO9567: ACGCCCTTTTAAATATCCG

GGGCTGAGGCGCAGTTTCCTACTCACCCGGATCCGAATCCTCCGCGGTGCTGTTTCAAGAGAGCCGGATTCCAGATCGCGCTCCAGCCCGGACTCGGAATTCCTGCCCTGCGGGTCTGCATTTTCATAACGGGCAGGTGTGAGTGCCCTGCAGCTGGAGACCAGAAGCCTGAAGGCAGCTCGGCCCTCCCCAGCCCACAGCGCCGTTATTCCGTTTCTATATCAGTAAACACATTTCATTTTCCGTAGACCAGGGCGGGGTGACGGGTGATCCCAGTCCTCGCAGTGAATTCCGGGCAGCAAAATTCAAAACACATGCGGCCAAGGCCGGGCACGGTGGTTCACGCCTGTAATCCCAGCACTTTGGGAGGTCGAGGCGGGCGATCACCTGAGGTCGGGAGCTCGAGACCAACCTGACCAACATGGGGAAATCCCGTCTCTACTAAAAATATAAAATTAGACGGGCTTGGTGGTGAATGCCTGTAATCCCAGCTAGTCGGGAGGCTGAGGCAGGAGAATCGCTTAAACCTTGGAGGCGGAGGTTGCGGTGAGCCGAGATCGCGCCATTGCACTTCAGCCTGGGCAACAAGAGGGAAAACTCCGTCGCAAAAACTTTCGGGGGCGGAGCGGAGCCCCGCCCTGGGTTATGTAAGCGACCGCGCTGGGCCGTTTCTCTTTCTTTTCCGGACCCTGCAGTGGCGCCTAAAGTCTGAGAGAGGGAAGTCGCCTCTGTGCTCGTGAGTGCATGGGGTATAAGGCAAGTGCTGAGGGAGAAAACGTAGTTGATGGGGTAGAGCAGACGGGGTTGGAGGTGGGGTGGAGGGGGAGGGCTTTGGACAGAAGACCTGGGAGGCTTGGTGGGGGAGGGGCGCCCAGGCCTGGGCACTAAGAAACAAGTCCCCTGGAGCTCAAGACCATCTCGGCCTCCCCTAGCCCAAGAGAGGACTGGCTTCATGACTCCCTGAAACCATTTCTAAATGCCTTAGAACAAACCTTGCATATTCATTATTGTTATTGAACTATTAAAAGTCTTTTTTGGGGGCGAGCTGAATCAGATCCTTTGCTGGAGCTGGCACACGGAGGAAGTCCTGGAGGGAGGGTAGACACCGTGGAGGTAAGGGCTTGGGACCTGTGTCAGGTGAGACCAAGTCGTGAGAGGTACTGCACCACGAAGTGGTTTAAACTATCAGTGTTTGACAGGATATATTGGCGGGTAAACCTAAGAGA

**Assembled sequence**

Insert 1

CGGACGTTTTTATGTACTGGGGTGGATGCAGTGGGCCCCACTCTGTGGTGCAGTACCTCTCACGACTTGGTCTCACGTTGTGGGGCTTAGCTTCATATTTTCAAACTGAAATATTCTCTTCCTTAACCTCCACATAAATCCAAGTTTATAATTTTTATTATTTTAAAATTTTATTTATTTTTCTGTTTTGGGGACAGGGTCTCCTCCTGTCACTCAGGCTGGAGTGCAATGGCACAATCATAGCTCACTGCAGCCTGGAACTCCTGGGCTTAAGCAATCTTCCTGCCTTCGATTCCCAAAGAGCTGGGATTATAGTCATGAACCACTGCAATCCACCCAAATCCAAGTTTACACTAAAAGATAAAATTCCAACATTGTAGGGGATTGGTCAGGTGGTGGGAATAATTATAAAGATAAAGTTATAGGAAATAGACACAAACCTTCTTGGAAGGTGGAAAGTTTTGCAAAAGCCTCAGGATAGGGTTATAGCTGAAAGCAGCCTAATCCCCTTACCTTGAGTTAATAGCTTCGAGTAAGTACAAAGACATGTAAGAGAGTTTATCTAAAGAGCATGTTTACCTTTGATCATTTGTAGGACTGCTCTCTCTGGGGGACTGCGACCAGATTAATTACCCACAGGTGTGTTGACTCAAAGCCTTTGTCATTAAATCTGTGCTGAATAAAGGCCCACAGGGCCAGATAGTCAGGGCACGCAGCTGCCACAACCCTTTCTGTGAGTGGCCTGGCCCTCTGGTGCACTCTTTCACTGAATATCGGTGTCTGAGTACATTATTCATCCATCGTGCAGCCTGGGTCTGCCGGTCAGACCCTGGCACAACATTTAAGAGGAAATGAAAGTCACAAAGTTATCCCAGTCTCTGGAGTCACTGTCAAAACTTTGGTGAGGAATCTTCCAGGTTTTCCCCTACTTCAAATATATATTAATATTATGTAAGTGATATTAGTGGCATTTT

Inserts 2, 3 and 4

GGCTCACGCCTGTAATCCCAGCACTTTGAGAGGCCAAGGCAGGCAGATCACGAGGTCAGGAGATCGAGACCATCCTGGCTAACACAGTGAAACCCCGTCTCTACTAAAAATACAAAAAATTAGTCGGGCGTGGTGGCAGGAGCCTGTAGTCCCAGCTACTCCAGAGGCTGAGGCAGCAGAATGCCCTGAACCCGGGAGGCGGAGCTTGCAGTGAGCCAAGATCGCGCCACTGCACTCCAGCCTGGGCGACAGAGCAAGACTCCGTCTCAAAAAAAAAAAACAAAAAACAAAAAAACTCTCCTTTACTTTTTCTCTCCCCTTTTCTTCCTATCTCTTCCCTCATTTCTTCAACACGTCCCCCCATCCTTCCCTCTTTTCTCCATTCTCTGCATTTGATCCCCGGTATATTCCAGCCTCCAGGCCAACAAACTTCTCCGCGTCCGCCGGGAGCAGGTCAGGGAAGGGACGCGAGGCGGCGCTGTCACCGCATTCTGAGCGCCGCAGCTCCCTGGGCCCCTTGTATCATTTCAGTGAAGGTCACTCCAGTCTTTCATGGAGGCCAAACTAAGGGTGTAAATTAGGATCCTCACTGAAGTGGCGGGACCCTAAGAGGCTTTTTCCTGGCCCCTTAGTTGTGGGTTTTCCTGCGGGCGGCGCAGCCGGTTTCCATCAGAACCGCCCAGAGGCGGACGCTGCCTTCCTGGGGTGACGGAGCAGCAGGAAGCGTTTTCGGATCCTGGAATACGTGGGCGGCCCGTGGGAGGGGCTGAGGCGCAGTTTCCTACTCACCCGGATCCGAATCCTCCGCGGTGCTGTTTCAAGAGAGCCGGATTCCAGATCGCGCTCCAGCCCGGACTCGGAATTCCTGCCCTGCGGGTCTGCATTTTCATAACGGGCAGGTGTGAGTGCCCTGCAGCTGGAGACCAGAAGCCTGAAGGCAGCTCGGCCCTCCCCAGCCCACAGCGCCGTTATTCCGTTTCTATATCAGTAAACACATTTCATTTTCCGTAGACCAGGGCGGGGTGACGGGTGATCCCAGTCCTCGCAGTGAATTCCGGGCAGCAAAATTCAAAACACATGCGGCCAAGGCCGGGCACGGTGGTTCACGCCTGTAATCCCAGCACTTTGGGAGGTCGAGGCGGGCGATCACCTGAGGTCGGGAGCTCGAGACCAACCTGACCAACATGGGGAAATCCCGTCTCTACTAAAAATATAAAATTAGACGGGCTTGGTGGTGAATGCCTGTAATCCCAGCTAGTCGGGAGGCTGAGGCAGGAGAATCGCTTAAACCTTGGAGGCGGAGGTTGCGGTGAGCCGAGATCGCGCCATTGCACTTCAGCCTGGGCAACAAGAGGGAAAACTCCGTCGCAAAAACTTTCGGGGGCGGAGCGGAGCCCCGCCCTGGGTTATGTAAGCGACCGCGCTGGGCCGTTTCTCTTTCTTTTCCGGACCCTGCAGTGGCGCCTAAAGTCTGAGAGAGGGAAGTCGCCTCTGTGCTCGTGAGTGCATGGGGTATAAGGCAAGTGCTGAGGGAGAAAACGTAGTTGATGGGGTAGAGCAGACGGGGTTGGAGGTGGGGTGGAGGGGGAGGGCTTTGGACAGAAGACCTGGGAGGCTTGGTGGGGGAGGGGCGCCCAGGCCTGGGCACTAAGAAACAAGTCCCCTGGAGCTCAAGACCATCTCGGCCTCCCCTAGCCCAAGAGAGGACTGGCTTCATGACTCCCTGAAACCATTTCTAAATGCCTTAGAACAAACCTTGCATATTCATTATTGTTATTGAACTATTAAAAGTCTTTTTTGGGGGCGAGCTGAATCAGATCCTTTGCTGGAGCTGGCACACGGAGGAAGTCCTGGAGGGAGGGTAGACACCGTGGAGGTAAGGGCTTGGGACCTGTGTCAGGTGAGACCAAGTCGTGAGAGGTACTGCACCACGAAGTGGTTTAAACTATCAGTGTTTGACAGGATATATTGGCGGGTAAACCTAAGAGA

## Colony 3

Forward CO9566: TGGTGTAAACAAATTGACGC

NNNNNNNNNNNNNGGACGTTTTTATGTACTGGGGTGGATGCAGTGGGCCCCACTCTGTGGTGCAGTACCTCTCACGACTTGGTCTCACGTTGTGGGGCTTAGCTTCATATTTTCAAACTGAAATATTCTCTTCCTTAACCTCCACATAAATCCAAGTTTATAATTTTTATTATTTTAAAATTTTATTTATTTTTCTGTTTTGGGGACAGGGTCTCCTCCTGTCACTCAGGCTGGAGTGCAATGGCACAATCATAGCTCACTGCAGCCTGGAACTCCTGGGCTTAAGCAATCTTCCTGCCTTCGATTCCCAAAGAGCTGGGATTATAGTCATGAACCACTGCAATCCACCCAAATCCAAGTTTACACTAAAAGATAAAATTCCAACATTGTAGGGGATTGGTCAGGTGGTGGGAATAATTATAAAGATAAAGTTATAGGAAATAGACACAAACCTTCTTGGAAGGTGGAAAGTTTTGCAAAAGCCTCAGGATAGGGTTATAGCTGAAAGCAGCCTAATCCCCTTACCTTGAGTTAATAGCTTCGAGTAAGTACAAAGACATGTAAGAGAGTTTATCTAAAGAGCATGTTTACCTTTGATCATTTGTAGGACTGCTCTCTCTGGGGGACTGCGACCAGATTAATTACCCACAGGTGTGTTGACTCAAAGCCTTTGTCATTAAATCTGTGCTGAATAAAGGCCCACAGGGCCAGATAGTCAGGGCACGCAGCTGCCACAACCCTTTCTGTGAGTGGCCTGGCCCTCTGGTGCACTCTTTCACTGAATATCGGTGTCTGAGTACATTATTCATCCATCGTGCAGCCTGGGTCTGCCGGTCAGACCCTGGCACAACATTTAAGAGGAAATGAAAGTCACAAAGTTATCCCAGTCTCTGGAGTCACTGTCAAAACTTTGGTGAGGAATCTTCCAGGTTTTCCCCTACTTCAAATATATATTAATATTATGTAAGTGATATTAGTGGCATTTTCGCCCAGG

Forward CO9574: AGAAATAATGAAACTACGTC

TNNNNNTCNNNNGGNGCGGTGGCTCACGCCTGTAATCCCAGCACTTTGAGAGGCCAAGGCAGGCAGATCACGAGGTCAGGAGATCGAGACCATCCTGGCTAACACAGTGAAACCCCGTCTCTACTAAAAATACAAAAAATTAGTCGGGCGTGGTGGCAGGAGCCTGTAGTCCCAGCTACTCCAGAGGCTGAGGCAGCAGAATGCCCTGAACCCGGGAGGCGGAGCTTGCAGTGAGCCAAGATCGCGCCACTGCACTCCAGCCTGGGCGACAGAGCAAGACTCCGTCTCAAAAAAAAAAAACAAAAAACAAAAAAACTCTCCTTTACTTTTTCTCTCCCCTTTTCTTCCTATCTCTTCCCTCATTTCTTCAACACGTCCCCCCATCCTTCCCTCTTTTCTCCATTCTCTGCATTTGATCCCCGGTATATTCCAGCCTCCAGGCCAACAAACTTCTCCGCGTCCGCCGGGAGCAGGTCAGGGAAGGGACGCGAGGCGGCGCTGTCACCGCATTCTGAGCGCCGCAGCTCCCTGGGCCCCTTGTATCATTTCAGTGAAGGTCACTCCAGTCTTTCATGGAGGCCAAACTAAGGGTGTAAATTAGGATCCTCACTGAAGTGGCGGGACCCTAAGAGGCTTTTTCCTGGCCCCTTAGTTGTGGGTTTTCCTGCGGGCGGCGCAGCCGGTTTCCATCAGAACCGCCCAGAGGCGGACGCTGCCTTCCTGGGGTGACGGAGCAGCAGGAAGCGTTTTCGGATCCTGGAATACGTGGGCGGCCCGTGGGAGGGGCTGAGGCGCAGTTTCCTACTCACCCGGATCCGAATCCTCCGCGGTGCTGTTTCAAGAGAGCCGGATTCCAGATCGCGCTCCAGCCCGGACTCGGAATTCCTGCCCTGCGGGTCTGCATTTTCATAACGGGCAGGTGTGAGTGCCCTGCAGCTGGAGACCAGAAGCCTGAAGGCAGCTCGGCCCTCCCCAGCCCACAGCGCCGTTATTCCGTTTCTATATCAGTAAACACATTTCATTTTCCGTANACCAGGGCGGGGTGACGGGTGATCCCAGTCCTCGCAGTGAATTCCGGGCAGCAAAATTCAAAACANATGCGGCCAAGGCCGGGCACGTGGGTTCACGCCTGAAATCCCAGCANTTTGGGNGGTCGANGCGNNNNNNNTCTGANGTGAGANNNNNAAACCNACCTGACNACAGGGNAAAC

Reverse CO9567: ACGCCCTTTTAAATATCCG

NNNNNNNNNCTCTTAGGTTTACCCGCCNATATATCCTGTCAAACACTGATAGTTTAAACCACTTCGTGGTGCAGTACCTCTCACGACTTGGTCTCACCTGACACAGGTCCCAAGCCCTTACCTCCACGGTGTCTACCCTCCCTCCAGGACTTCCTCCGTGTGCCAGCTCCAGCAAAGGATCTGATTCAGCTCGCCCCCAAAAAAGACTTTTAATAGTTCAATAACAATAATGAATATGCAAGGTTTGTTCTAAGGCATTTAGAAATGGTTTCAGGGAGTCATGAAGCCAGTCCTCTCTTGGGCTAGGGGAGGCCGAGATGGTCTTGAGCTCCAGGGGACTTGTTTCTTAGTGCCCAGGCCTGGGCGCCCCTCCCCCACCAAGCCTCCCAGGTCTTCTGTCCAAAGCCCTCCCCCTCCACCCCACCTCCAACCCCGTCTGCTCTACCCCATCAACTACGTTTTCTCCCTCAGCACTTGCCTTATACCCCATGCACTCACGAGCACAGAGGCGACTTCCCTCTCTCAGACTTTAGGCGCCACTGCAGGGTCCGGAAAAGAAAGAGAAACGGCCCAGCGCGGTCGCTTACATAACCCAGGGCGGGGCTCCGCTCCGCCCCCGAAAGTTTTTGCGACGGAGTTTTCCCTCTTGTTGCCCAGGCTGAAGTGCAATGGCGCGATCTCGGCTCACCGCAACCTCCGCCTCCAAGGTTTAAGCGATTCTCCTGCCTCAGCCTCCCGACTAGCTGGGATTACAGGCATTCACCACCAAGCCCGTCTAATTTTATATTTTTAGTAGAGACGGGATTTCCCCATGTTGGTCAGGTTGGTCTCGAGCTCCCGACCTCAGGTGATCGCCCGCCTCGACCTCCCAAAGTGCTGGGATTACAGGCGTGAACCACCGTGCCCGGCCTTGGCCGCATGTGTTTTGAATTTTGCTGCCCGGAATTCACTGCGAGGACTGGGATCACCCGTCACCCCGCCCTGGTCTACGGAAAATGAAATGTGTTTACTGATATAGAAACGGAATAACGGCGCTGTGGGCTGGGGAGGGCCGAGCTGCCTTCAGGCTTCTGGTCTCCAGCTGCAGGGCACTCACACCTGCCCGTTATGAAAATGCAGACCCGCAGGGCAGGAATTCCGAGTCCGGGCTGGAGCGCGATCTGGAATCCGGCTCTCTTGAAACAGCACCGNGGAGGATTCGGATCCGGGTGAGTAGGAAACTGCGCCTCAGCCCCTCCACGGGNCGCCCACGTATTCCAGGATCCGAAAACGCTTCCTGCTGCTCCGTNACCCCAGAAAGGAACGTCCGCCNCTGGNNGGTTCTGAAGGAAACCGGCTGNNCCGCCNNCAGNAAANCCCNNACTAAGGGGNCNNGAAAAAGCTNNTTAGGGNCCNNCCNTTNNNTGNNGNCNNAATTTAACCCCTAANTTGGCCTCCTGAGAGAACNGCGGNCCTNNCCG

Reverse Complement Reverse CO9567: ACGCCCTTTTAAATATCCG

CCCGTGGAGGGGCTGAGGCGCAGTTTCCTACTCACCCGGATCCGAATCCTCCNCGGTGCTGTTTCAAGAGAGCCGGATTCCAGATCGCGCTCCAGCCCGGACTCGGAATTCCTGCCCTGCGGGTCTGCATTTTCATAACGGGCAGGTGTGAGTGCCCTGCAGCTGGAGACCAGAAGCCTGAAGGCAGCTCGGCCCTCCCCAGCCCACAGCGCCGTTATTCCGTTTCTATATCAGTAAACACATTTCATTTTCCGTAGACCAGGGCGGGGTGACGGGTGATCCCAGTCCTCGCAGTGAATTCCGGGCAGCAAAATTCAAAACACATGCGGCCAAGGCCGGGCACGGTGGTTCACGCCTGTAATCCCAGCACTTTGGGAGGTCGAGGCGGGCGATCACCTGAGGTCGGGAGCTCGAGACCAACCTGACCAACATGGGGAAATCCCGTCTCTACTAAAAATATAAAATTAGACGGGCTTGGTGGTGAATGCCTGTAATCCCAGCTAGTCGGGAGGCTGAGGCAGGAGAATCGCTTAAACCTTGGAGGCGGAGGTTGCGGTGAGCCGAGATCGCGCCATTGCACTTCAGCCTGGGCAACAAGAGGGAAAACTCCGTCGCAAAAACTTTCGGGGGCGGAGCGGAGCCCCGCCCTGGGTTATGTAAGCGACCGCGCTGGGCCGTTTCTCTTTCTTTTCCGGACCCTGCAGTGGCGCCTAAAGTCTGAGAGAGGGAAGTCGCCTCTGTGCTCGTGAGTGCATGGGGTATAAGGCAAGTGCTGAGGGAGAAAACGTAGTTGATGGGGTAGAGCAGACGGGGTTGGAGGTGGGGTGGAGGGGGAGGGCTTTGGACAGAAGACCTGGGAGGCTTGGTGGGGGAGGGGCGCCCAGGCCTGGGCACTAAGAAACAAGTCCCCTGGAGCTCAAGACCATCTCGGCCTCCCCTAGCCCAAGAGAGGACTGGCTTCATGACTCCCTGAAACCATTTCTAAATGCCTTAGAACAAACCTTGCATATTCATTATTGTTATTGAACTATTAAAAGTCTTTTTTGGGGGCGAGCTGAATCAGATCCTTTGCTGGAGCTGGCACACGGAGGAAGTCCTGGAGGGAGGGTAGACACCGTGGAGGTAAGGGCTTGGGACCTGTGTCAGGTGAGACCAAGTCGTGAGAGGTACTGCACCACGAAGTGGTTTAAACTATCAGTGTTTGACAGGATATAT

**Assembled sequence**

Insert 1

GGACGTTTTTATGTACTGGGGTGGATGCAGTGGGCCCCACTCTGTGGTGCAGTACCTCTCACGACTTGGTCTCACGTTGTGGGGCTTAGCTTCATATTTTCAAACTGAAATATTCTCTTCCTTAACCTCCACATAAATCCAAGTTTATAATTTTTATTATTTTAAAATTTTATTTATTTTTCTGTTTTGGGGACAGGGTCTCCTCCTGTCACTCAGGCTGGAGTGCAATGGCACAATCATAGCTCACTGCAGCCTGGAACTCCTGGGCTTAAGCAATCTTCCTGCCTTCGATTCCCAAAGAGCTGGGATTATAGTCATGAACCACTGCAATCCACCCAAATCCAAGTTTACACTAAAAGATAAAATTCCAACATTGTAGGGGATTGGTCAGGTGGTGGGAATAATTATAAAGATAAAGTTATAGGAAATAGACACAAACCTTCTTGGAAGGTGGAAAGTTTTGCAAAAGCCTCAGGATAGGGTTATAGCTGAAAGCAGCCTAATCCCCTTACCTTGAGTTAATAGCTTCGAGTAAGTACAAAGACATGTAAGAGAGTTTATCTAAAGAGCATGTTTACCTTTGATCATTTGTAGGACTGCTCTCTCTGGGGGACTGCGACCAGATTAATTACCCACAGGTGTGTTGACTCAAAGCCTTTGTCATTAAATCTGTGCTGAATAAAGGCCCACAGGGCCAGATAGTCAGGGCACGCAGCTGCCACAACCCTTTCTGTGAGTGGCCTGGCCCTCTGGTGCACTCTTTCACTGAATATCGGTGTCTGAGTACATTATTCATCCATCGTGCAGCCTGGGTCTGCCGGTCAGACCCTGGCACAACATTTAAGAGGAAATGAAAGTCACAAAGTTATCCCAGTCTCTGGAGTCACTGTCAAAACTTTGGTGAGGAATCTTCCAGGTTTTCCCCTACTTCAAATATATATTAATATTATGTAAGTGATATTAGTGGCATTTTCGCCCAGG

Inserts 2, 3, 4

GCGGTGGCTCACGCCTGTAATCCCAGCACTTTGAGAGGCCAAGGCAGGCAGATCACGAGGTCAGGAGATCGAGACCATCCTGGCTAACACAGTGAAACCCCGTCTCTACTAAAAATACAAAAAATTAGTCGGGCGTGGTGGCAGGAGCCTGTAGTCCCAGCTACTCCAGAGGCTGAGGCAGCAGAATGCCCTGAACCCGGGAGGCGGAGCTTGCAGTGAGCCAAGATCGCGCCACTGCACTCCAGCCTGGGCGACAGAGCAAGACTCCGTCTCAAAAAAAAAAAACAAAAAACAAAAAAACTCTCCTTTACTTTTTCTCTCCCCTTTTCTTCCTATCTCTTCCCTCATTTCTTCAACACGTCCCCCCATCCTTCCCTCTTTTCTCCATTCTCTGCATTTGATCCCCGGTATATTCCAGCCTCCAGGCCAACAAACTTCTCCGCGTCCGCCGGGAGCAGGTCAGGGAAGGGACGCGAGGCGGCGCTGTCACCGCATTCTGAGCGCCGCAGCTCCCTGGGCCCCTTGTATCATTTCAGTGAAGGTCACTCCAGTCTTTCATGGAGGCCAAACTAAGGGTGTAAATTAGGATCCTCACTGAAGTGGCGGGACCCTAAGAGGCTTTTTCCTGGCCCCTTAGTTGTGGGTTTTCCTGCGGGCGGCGCAGCCGGTTTCCATCAGAACCGCCCAGAGGCGGACGCTGCCTTCCTGGGGTGACGGAGCAGCAGGAAGCGTTTTCGGATCCTGGAATACGTGGGCGGCCCGTGGGAGGGGCTGAGGCGCAGTTTCCTACTCACCCGGATCCGAATCCTCCGCGGTGCTGTTTCAAGAGAGCCGGATTCCAGATCGCGCTCCAGCCCGGACTCGGAATTCCTGCCCTGCGGGTCTGCATTTTCATAACGGGCAGGTGTGAGTGCCCTGCAGCTGGAGACCAGAAGCCTGAAGGCAGCTCGGCCCTCCCCAGCCCACAGCGCCGTTATTCCGTTTCTATATCAGTAAACACATTTCATTTTCCGTAGACCAGGGCGGGGTGACGGGTGATCCCAGTCCTCGCAGTGAATTCCGGGCAGCAAAATTCAAAACACATGCGGCCAAGGCCGGGCACGGTGGTTCACGCCTGTAATCCCAGCACTTTGGGAGGTCGAGGCGGGCGATCACCTGAGGTCGGGAGCTCGAGACCAACCTGACCAACATGGGGAAATCCCGTCTCTACTAAAAATATAAAATTAGACGGGCTTGGTGGTGAATGCCTGTAATCCCAGCTAGTCGGGAGGCTGAGGCAGGAGAATCGCTTAAACCTTGGAGGCGGAGGTTGCGGTGAGCCGAGATCGCGCCATTGCACTTCAGCCTGGGCAACAAGAGGGAAAACTCCGTCGCAAAAACTTTCGGGGGCGGAGCGGAGCCCCGCCCTGGGTTATGTAAGCGACCGCGCTGGGCCGTTTCTCTTTCTTTTCCGGACCCTGCAGTGGCGCCTAAAGTCTGAGAGAGGGAAGTCGCCTCTGTGCTCGTGAGTGCATGGGGTATAAGGCAAGTGCTGAGGGAGAAAACGTAGTTGATGGGGTAGAGCAGACGGGGTTGGAGGTGGGGTGGAGGGGGAGGGCTTTGGACAGAAGACCTGGGAGGCTTGGTGGGGGAGGGGCGCCCAGGCCTGGGCACTAAGAAACAAGTCCCCTGGAGCTCAAGACCATCTCGGCCTCCCCTAGCCCAAGAGAGGACTGGCTTCATGACTCCCTGAAACCATTTCTAAATGCCTTAGAACAAACCTTGCATATTCATTATTGTTATTGAACTATTAAAAGTCTTTTTTGGGGGCGAGCTGAATCAGATCCTTTGCTGGAGCTGGCACACGGAGGAAGTCCTGGAGGGAGGGTAGACACCGTGGAGGTAAGGGCTTGGGACCTGTGTCAGGTGAGACCAAGTCGTGAGAGGTACTGCACCACGAAGTGGTTTAAACTATCAGTGTTTGACAGGATATAT

## Colony 4

Forward CO9566: TGGTGTAAACAAATTGACGC

NNNNNNNNNNGCGGACGTTTTTATGNACTGGGGTGGATGCAGTGGGCCCCACTCTGTGGTGCAGTACCTCTCACGACTTGGTCTCACGTTGTGGGGCTTAGCTTCATATTTTCAAACTGAAATATTCTCTTCCTTAACCTCCACATAAATCCAAGTTTATAATTTTTATTATTTTAAAATTTTATTTATTTTTCTGTTTTGGGGACAGGGTCTCCTCCTGTCACTCAGGCTGGAGTGCAATGGCACAATCATAGCTCACTGCAGCCTGGAACTCCTGGGCTTAAGCAATCTTCCTGCCTTCGATTCCCAAAGAGCTGGGATTATAGTCATGAACCACTGCAATCCACCCAAATCCAAGTTTACACTAAAAGATAAAATTCCAACATTGTAGGGGATTGGTCAGGTGGTGGGAATAATTATAAAGATAAAGTTATAGGAAATAGACACAAACCTTCTTGGAAGGTGGAAAGTTTTGCAAAAGCCTCAGGATAGGGTTATAGCTGAAAGCAGCCTAATCCCCTTACCTTGAGTTAATAGCTTCGAGTAAGTACAAAGACATGTAAGAGAGTTTATCTAAAGAGCATGTTTACCTTTGATCATTTGTAGGACTGCTCTCTCTGGGGGACTGCGACCAGATTAATTACCCACAGGTGTGTTGACTCAAAGCCTTTGTCATTAAATCTGTGCTGAATAAAGGCCCACAGGGCCAGATAGTCAGGGCACGCAGCTGCCACAACCCTTTCTGTGAGTGGCCTGGCCCTCTGGTGCACTCTTTCACTGAATATCGGTGTCTGAGTACATTATTCATCCATCGTGCAGCCTGGGTCTGCCGGTCAGACCCTGGCACAACATTTAAGAGGAAATGAAAGTCACAAAGTTATCCCAGTCTCTGGAGTCACTGTCAAAACTTTGGTGAGGAATCTTCCAGGTTTTCCCCTACTTCAAATATATATTAATATTATGTAAGTGATATTAGTGGCATTTTCGCCC

Forward CO9574: AGAAATAATGAAACTACGTC

NNNNNNNTCNNNNNNGNGCGGNGGCTCACGCCTGTAATCCCAGCACTTTGAGAGGCCAAGGCAGGCAGATCACGAGGTCAGGAGATCGAGACCATCCTGGCTAACACAGTGAAACCCCGTCTCTACTAAAAATACAAAAAATTAGTCGGGCGTGGTGGCAGGAGCCTGTAGTCCCAGCTACTCCAGAGGCTGAGGCAGCAGAATGCCCTGAACCCGGGAGGCGGAGCTTGCAGTGAGCCAAGATCGCGCCACTGCACTCCAGCCTGGGCGACAGAGCAAGACTCCGTCTCAAAAAAAAAAAACAAAAAACAAAAAAACTCTCCTTTACTTTTTCTCTCCCCTTTTCTTCCTATCTCTTCCCTCATTTCTTCAACACGTCCCCCCATCCTTCCCTCTTTTCTCCATTCTCTGCATTTGATCCCCGGTATATTCCAGCCTCCAGGCCAACAAACTTCTCCGCGTCCGCCGGGAGCAGGTCAGGGAAGGGACGCGAGGCGGCGCTGTCACCGCATTCTGAGCGCCGCAGCTCCCTGGGCCCCTTGTATCATTTCAGTGAAGGTCACTCCAGTCTTTCATGGAGGCCAAACTAAGGGTGTAAATTAGGATCCTCACTGAAGTGGCGGGACCCTAAGAGGCTTTTTCCTGGCCCCTTAGTTGTGGGTTTTCCTGCGGGCGGCGCAGCCGGTTTCCATCAGAACCGCCCAGAGGCGGACGCTGCCTTCCTGGGGTGACGGAGCAGCAGGAAGCGTTTTCGGATCCTGGAATACGTGGGCGGCCCGTGGGAGGTGCTGAGGCGCAGTTTCCTACTCACCCGGATCCGAATCCTCCGCGGTGCTGTTTCAAGAGAGCCGGATTCCAGATCGCGCTCCAGCCCGGACTCGGAATTCCTGCCCTGCGGGTCTGCATTTTCATAACGGGCAGGTGTGAGTGCCCTGCAGCTGGAGACCAGAAGCCTGAAGGCAGCTCGGCCCTCCCCAGCCCACAGCGCCGTTATTCCGTTTCTATATCAGTAAACACATTTCATTTTCCGGAGACCAGGGCGGGGTGACGGNTGATCCAGTCCTCGCAGTGAATTCCGNNAGCAAAATTCAAAACACNTGCGNCTNNAGCCNGTCAACANNNGTCCNTCNGTAATCCNNNNNTTTNGGAAGNNNNGCGTGTGANNN

Reverse CO9567: ACGCCCTTTTAAATATCCG

NNNNNNNNNCTCTTAGGTTTACCCGCCAATATATCCTGTCAAACACTGATAGTTTAAACCACTTCGTGGTGCAGTACCTCTCACGACTTGGTCTCACCTGACACAGGTCCCAAGCCCTTACCTCCACGGTGTCTACCCTCCCTCCAGGACTTCCTCCGTGTGCCAGCTCCAGCAAAGGATCTGATTCAGCTCGCCCCCAAAAAAGACTTTTAATAGTTCAATAACAATAATGAATATGCAAGGTTTGTTCTAAGGCATTTAGAAATGGTTTCAGGGAGTCATGAAGCCAGTCCTCTCTTGGGCTAGGGGAGGCCGAGATGGTCTTGAGCTCCAGGGGACTTGTTTCTTAGTGCCCAGGCCTGGGCGCCCCTCCCCCACCAAGCCTCCCAGGTCTTCTGTCCAAAGCCCTCCCCCTCCACCCCACCTCCAACCCCGTCTGCTCTACCCCATCAACTACGTTTTCTCCCTCAGCACTTGCCTTATACCCCATGCACTCACGAGCACAGAGGCGACTTCCCTCTCTCAGACTTTAGGCGCCACTGCAGGGTCCGGAAAAGAAAGAGAAACGGCCCAGCGCGGTCGCTTACATAACCCAGGGCGGGGCTCCGCTCCGCCCCCGAAAGTTTTTGCGACGGAGTTTTCCCTCTTGTTGCCCAGGCTGAAGTGCAATGGCGCGATCTCGGCTCACCGCAACCTCCGCCTCCAAGGTTTAAGCGATTCTCCTGCCTCAGCCTCCCGACTAGCTGGGATTACAGGCATTCACCACCAAGCCCGTCTAATTTTATATTTTTAGTAGAGACGGGATTTCCCCATGTTGGTCAGGTTGGTCTCGAGCTCCCGACCTCAGGTGATCGCCCGCCTCGACCTCCCAAAGTGCTGGGATTACAGGCGTGAACCACCGTGCCCGGCCTTGGCCGCATGTGTTTTGAATTTTGCTGCCCGGAATTCACTGCGAGGACTGGGATCACCCGTCACCCCGCCCTGGTCTACGGAAAATGAAATGTGTTTACTGATATAGAAACGGAATAACGGCGCTGTGGGCTGGGGAGGGCCGAGCTGCCTTCAGGCTTCTGGTCTCCAGCTGCAGGGCACTCACACCTGCCCGTTATGAAAATGCAGACCCGCAGGGCAGGAATTCCGAGTCCGGGCTGGAGCGCGATCTGGAATCCGGCTCTCTTGAAACAGCACCGCGGAGGATTCGGATCCGGGTGAGTAGGAAACTGCGCCTCAGCCCCTCCACGGGGCGCCCACGTATTCCAGGATCCGAAAACGCTTCCTGCTGCTCCGTNACCCCAGAAAGGCANNGTCCGCCTCTGGNNNGTTCTGATGGAAACCGGCTGNNCCNCCCGCAGGAAAACCCACACTAAGGGGCCAGGAAAAAGCCTCTTAGGGTCCNNCCNNTTCAGGNAGGATCCTAATTTAACCCCTTAATTTGGCCTNCTGAAAGAATGGNAGGNNNCNNNNNNNNAAAGAAANNNNGNNNNNCCNGNNNNNTGGGGNNNTTNAAAANNGNGGNNNNNNCCCCCNNNCCGNNNCTTNCNNNNNNNNNCCCCNNNNNNNNNGGAAAAANNTTNNNNNGNNNGNAGGNNNNNANNNNNCCGNNNNNANANTNNNNNAAANNNNNAAAANAANNNTNNANNNNNGT

Reverse Complement Reverse CO9567: ACGCCCTTTTAAATATCCG

ACGGAGCAGCAGGAAGCGTTTTCGGATCCTGGAATACGTGGGCGCCCCGTGGAGGGGCTGAGGCGCAGTTTCCTACTCACCCGGATCCGAATCCTCCGCGGTGCTGTTTCAAGAGAGCCGGATTCCAGATCGCGCTCCAGCCCGGACTCGGAATTCCTGCCCTGCGGGTCTGCATTTTCATAACGGGCAGGTGTGAGTGCCCTGCAGCTGGAGACCAGAAGCCTGAAGGCAGCTCGGCCCTCCCCAGCCCACAGCGCCGTTATTCCGTTTCTATATCAGTAAACACATTTCATTTTCCGTAGACCAGGGCGGGGTGACGGGTGATCCCAGTCCTCGCAGTGAATTCCGGGCAGCAAAATTCAAAACACATGCGGCCAAGGCCGGGCACGGTGGTTCACGCCTGTAATCCCAGCACTTTGGGAGGTCGAGGCGGGCGATCACCTGAGGTCGGGAGCTCGAGACCAACCTGACCAACATGGGGAAATCCCGTCTCTACTAAAAATATAAAATTAGACGGGCTTGGTGGTGAATGCCTGTAATCCCAGCTAGTCGGGAGGCTGAGGCAGGAGAATCGCTTAAACCTTGGAGGCGGAGGTTGCGGTGAGCCGAGATCGCGCCATTGCACTTCAGCCTGGGCAACAAGAGGGAAAACTCCGTCGCAAAAACTTTCGGGGGCGGAGCGGAGCCCCGCCCTGGGTTATGTAAGCGACCGCGCTGGGCCGTTTCTCTTTCTTTTCCGGACCCTGCAGTGGCGCCTAAAGTCTGAGAGAGGGAAGTCGCCTCTGTGCTCGTGAGTGCATGGGGTATAAGGCAAGTGCTGAGGGAGAAAACGTAGTTGATGGGGTAGAGCAGACGGGGTTGGAGGTGGGGTGGAGGGGGAGGGCTTTGGACAGAAGACCTGGGAGGCTTGGTGGGGGAGGGGCGCCCAGGCCTGGGCACTAAGAAACAAGTCCCCTGGAGCTCAAGACCATCTCGGCCTCCCCTAGCCCAAGAGAGGACTGGCTTCATGACTCCCTGAAACCATTTCTAAATGCCTTAGAACAAACCTTGCATATTCATTATTGTTATTGAACTATTAAAAGTCTTTTTTGGGGGCGAGCTGAATCAGATCCTTTGCTGGAGCTGGCACACGGAGGAAGTCCTGGAGGGAGGGTAGACACCGTGGAGGTAAGGGCTTGGGACCTGTGTCAGGTGAGACCAAGTCGTGAGAGGTACTGCACCACGAAGTGGTTTAAACTATCAGTGTTTGACAGGATATATTGGCGGGTAAACCTAAGAG

**Assembled sequence**

Insert 1

ACTGGGGTGGATGCAGTGGGCCCCACTCTGTGGTGCAGTACCTCTCACGACTTGGTCTCACGTTGTGGGGCTTAGCTTCATATTTTCAAACTGAAATATTCTCTTCCTTAACCTCCACATAAATCCAAGTTTATAATTTTTATTATTTTAAAATTTTATTTATTTTTCTGTTTTGGGGACAGGGTCTCCTCCTGTCACTCAGGCTGGAGTGCAATGGCACAATCATAGCTCACTGCAGCCTGGAACTCCTGGGCTTAAGCAATCTTCCTGCCTTCGATTCCCAAAGAGCTGGGATTATAGTCATGAACCACTGCAATCCACCCAAATCCAAGTTTACACTAAAAGATAAAATTCCAACATTGTAGGGGATTGGTCAGGTGGTGGGAATAATTATAAAGATAAAGTTATAGGAAATAGACACAAACCTTCTTGGAAGGTGGAAAGTTTTGCAAAAGCCTCAGGATAGGGTTATAGCTGAAAGCAGCCTAATCCCCTTACCTTGAGTTAATAGCTTCGAGTAAGTACAAAGACATGTAAGAGAGTTTATCTAAAGAGCATGTTTACCTTTGATCATTTGTAGGACTGCTCTCTCTGGGGGACTGCGACCAGATTAATTACCCACAGGTGTGTTGACTCAAAGCCTTTGTCATTAAATCTGTGCTGAATAAAGGCCCACAGGGCCAGATAGTCAGGGCACGCAGCTGCCACAACCCTTTCTGTGAGTGGCCTGGCCCTCTGGTGCACTCTTTCACTGAATATCGGTGTCTGAGTACATTATTCATCCATCGTGCAGCCTGGGTCTGCCGGTCAGACCCTGGCACAACATTTAAGAGGAAATGAAAGTCACAAAGTTATCCCAGTCTCTGGAGTCACTGTCAAAACTTTGGTGAGGAATCTTCCAGGTTTTCCCCTACTTCAAATATATATTAATATTATGTAAGTGATATTAGTGGCATTTTCGCCC

Inserts 2, 3 and 4

GCGGNGGCTCACGCCTGTAATCCCAGCACTTTGAGAGGCCAAGGCAGGCAGATCACGAGGTCAGGAGATCGAGACCATCCTGGCTAACACAGTGAAACCCCGTCTCTACTAAAAATACAAAAAATTAGTCGGGCGTGGTGGCAGGAGCCTGTAGTCCCAGCTACTCCAGAGGCTGAGGCAGCAGAATGCCCTGAACCCGGGAGGCGGAGCTTGCAGTGAGCCAAGATCGCGCCACTGCACTCCAGCCTGGGCGACAGAGCAAGACTCCGTCTCAAAAAAAAAAAACAAAAAACAAAAAAACTCTCCTTTACTTTTTCTCTCCCCTTTTCTTCCTATCTCTTCCCTCATTTCTTCAACACGTCCCCCCATCCTTCCCTCTTTTCTCCATTCTCTGCATTTGATCCCCGGTATATTCCAGCCTCCAGGCCAACAAACTTCTCCGCGTCCGCCGGGAGCAGGTCAGGGAAGGGACGCGAGGCGGCGCTGTCACCGCATTCTGAGCGCCGCAGCTCCCTGGGCCCCTTGTATCATTTCAGTGAAGGTCACTCCAGTCTTTCATGGAGGCCAAACTAAGGGTGTAAATTAGGATCCTCACTGAAGTGGCGGGACCCTAAGAGGCTTTTTCCTGGCCCCTTAGTTGTGGGTTTTCCTGCGGGCGGCGCAGCCGGTTTCCATCAGAACCGCCCAGAGGCGGACGCTGCCTTCCTGGGGTGACGGAGCAGCAGGAAGCGTTTTCGGATCCTGGAATACGTGGGCGCCCCGTGGAGGGGCTGAGGCGCAGTTTCCTACTCACCCGGATCCGAATCCTCCGCGGTGCTGTTTCAAGAGAGCCGGATTCCAGATCGCGCTCCAGCCCGGACTCGGAATTCCTGCCCTGCGGGTCTGCATTTTCATAACGGGCAGGTGTGAGTGCCCTGCAGCTGGAGACCAGAAGCCTGAAGGCAGCTCGGCCCTCCCCAGCCCACAGCGCCGTTATTCCGTTTCTATATCAGTAAACACATTTCATTTTCCGTAGACCAGGGCGGGGTGACGGGTGATCCCAGTCCTCGCAGTGAATTCCGGGCAGCAAAATTCAAAACACATGCGGCCAAGGCCGGGCACGGTGGTTCACGCCTGTAATCCCAGCACTTTGGGAGGTCGAGGCGGGCGATCACCTGAGGTCGGGAGCTCGAGACCAACCTGACCAACATGGGGAAATCCCGTCTCTACTAAAAATATAAAATTAGACGGGCTTGGTGGTGAATGCCTGTAATCCCAGCTAGTCGGGAGGCTGAGGCAGGAGAATCGCTTAAACCTTGGAGGCGGAGGTTGCGGTGAGCCGAGATCGCGCCATTGCACTTCAGCCTGGGCAACAAGAGGGAAAACTCCGTCGCAAAAACTTTCGGGGGCGGAGCGGAGCCCCGCCCTGGGTTATGTAAGCGACCGCGCTGGGCCGTTTCTCTTTCTTTTCCGGACCCTGCAGTGGCGCCTAAAGTCTGAGAGAGGGAAGTCGCCTCTGTGCTCGTGAGTGCATGGGGTATAAGGCAAGTGCTGAGGGAGAAAACGTAGTTGATGGGGTAGAGCAGACGGGGTTGGAGGTGGGGTGGAGGGGGAGGGCTTTGGACAGAAGACCTGGGAGGCTTGGTGGGGGAGGGGCGCCCAGGCCTGGGCACTAAGAAACAAGTCCCCTGGAGCTCAAGACCATCTCGGCCTCCCCTAGCCCAAGAGAGGACTGGCTTCATGACTCCCTGAAACCATTTCTAAATGCCTTAGAACAAACCTTGCATATTCATTATTGTTATTGAACTATTAAAAGTCTTTTTTGGGGGCGAGCTGAATCAGATCCTTTGCTGGAGCTGGCACACGGAGGAAGTCCTGGAGGGAGGGTAGACACCGTGGAGGTAAGGGCTTGGGACCTGTGTCAGGTGAGACCAAGTCGTGAGAGGTACTGCACCACGAAGTGGTTTAAACTATCAGTGTTTGACAGGATATATTGGCGGGTAAACCTAAGAG

## Colony 5

Forward CO9566: TGGTGTAAACAAATTGACGC

NNNNNNNNNNNGCGGACGTTTTTATGNACTGGGGTGGATGCAGTGGGCCCCACTCTGTGGTGCAGTACCTCTCACGACTTGGTCTCACGTTGTGGGGCTTAGCTTCATATTTTCAAACTGAAATATTCTCTTCCTTAACCTCCACATAAATCCAAGTTTATAATTTTTATTATTTTAAAATTTTATTTATTTTTCTGTTTTGGGGACAGGGTCTCCTCCTGTCACTCAGGCTGGAGTGCAATGGCACAATCATAGCTCACTGCAGCCTGGAACTCCTGGGCTTAAGCAATCTTCCTGCCTTCGATTCCCAAAGAGCTGGGATTATAGTCATGAACCACTGCAATCCACCCAAATCCAAGTTTACACTAAAAGATAAAATTCCAACATTGTAGGGGATTGGTCAGGTGGTGGGAATAATTATAAAGATAAAGTTATAGGAAATAGACACAAACCTTCTTGGAAGGTGGAAAGTTTTGCAAAAGCCTCAGGATAGGGTTATAGCTGAAAGCAGCCTAATCCCCTTACCTTGAGTTAATAGCTTCGAGTAAGTACAAAGACATGTAAGAGAGTTTATCTAAAGAGCATGTTTACCTTTGATCATTTGTAGGACTGCTCTCTCTGGGGGACTGCGACCAGATTAATTACCCACAGGTGTGTTGACTCAAAGCCTTTGTCATTAAATCTGTGCTGAATAAAGGCCCACAGGGCCAGATAGTCAGGGCACGCAGCTGCCACAACCCTTTCTGTGAGTGGCCTGGCCCTCTGGTGCACTCTTTCACTGAATATCGGTGTCTGAGTACATTATTCATCCATCGTGCAGCCTGGGTCTGCCGGTCAGACCCTGGCACAACATTTAAGAGGAAATGAAAGTCACAAAGTTATCCCAGTCTCTGGAGTCACTGTCAAAACTTTGGTGAGGAATCTTCCAGGTTTTCCCCTACTTCAAATATATATTAATATTATGTAAGTGATATTAGTGGCATTTTCGCC

Forward CO9574: AGAAATAATGAAACTACGTC

NNNNNNNNNNNNNNNGNGCGGTGGCTCACGCCTGTAATCCCAGCACTTTGAGAGGCCAAGGCAGGCAGATCACGAGGTCAGGAGATCGAGACCATCCTGGCTAACACAGTGAAACCCCGTCTCTACTAAAAATACAAAAAATTAGTCGGGCGTGGTGGCAGGAGCCTGTAGTCCCAGCTACTCCAGAGGCTGAGGCAGCAGAATGCCCTGAACCCGGGAGGCGGAGCTTGCAGTGAGCCAAGATCGCGCCACTGCACTCCAGCCTGGGCGACAGAGCAAGACTCCGTCTCAAAAAAAAAAAACAAAAAACAAAAAAACTCTCCTTTACTTTTTCTCTCCCCTTTTCTTCCTATCTCTTCCCTCATTTCTTCAACACGTCCCCCCATCCTTCCCTCTTTTCTCCATTCTCTGCATTTGATCCCCGGTATATTCCAGCCTCCAGGCCAACAAACTTCTCCGCGTCCGCCGGGAGCAGGTCAGGGAAGGGACGCGAGGCGGCGCTGTCACCGCATTCTGAGCGCCGCAGCTCCCTGGGCCCCTTGTATCATTTCAGTGAAGGTCACTCCAGTCTTTCATGGAGGCCAAACTAAGGGTGTAAATTAGGATCCTCACTGAAGTGGCGGGACCCTAAGAGGCTTTTTCCTGGCCCCTTAGTTGTGGGTTTTCCTGCGGGCGGCGCAGCCGGTTTCCATCAGAACCGCCCAGAGGCGGACGCTGCCTTCCTGGGGTGACGGAGCAGCAGGAAGCGTTTTCGGATCCTGGAATACGTGGGCGGCCCGTGTGAGGGGCTGAGGCGCAGTTTCCTACTCACCCGGATCCGAATCCTCCGCGGTGCTGTTTCAAGAGAGCCGGATTCCAGATCGCGCTCCAGCCCGGACTCGGAATTCCTGCCCTGCGGGTCTGCATTTTCATAACGTGCNNNNNTNANGGCCTGCAGCTGTAGACCATAAGTCTGAATNCAGTCNTTCCTCCCAGNCCACAGNGACGTNNNTCCGTNNNTANNTCA

Reverse CO9567: ACGCCCTTTTAAATATCCG

NNNNNNNNNNNNTCTTAGGTTTACCCGCCNATATATCCTGTCAAACACTGATAGTTTAAACCACTTCGTGGTGCAGTACCTCTCACGACTTGGTCTCACCTGACACAGGTCCCAAGCCCTTACCTCCACGGTGTCTACCCTCCCTCCAGGACTTCCTCCGTGTGCCAGCTCCAGCAAAGGATCTGATTCAGCTCGCCCCCAAAAAAGACTTTTAATAGTTCAATAACAATAATGAATATGCAAGGTTTGTTCTAAGGCATTTAGAAATGGTTTCAGGGAGTCATGAAGCCAGTCCTCTCTTGGGCTAGGGGAGGCCGAGATGGTCTTGAGCTCCAGGGGACTTGTTTCTTAGTGCCCAGGCCTGGGCGCCCCTCCCCCACCAAGCCTCCCAGGTCTTCTGTCCAAAGCCCTCCCCCTCCACCCCACCTCCAACCCCGTCTGCTCTACCCCATCAACTACGTTTTCTCCCTCAGCACTTGCCTTATACCCCATGCACTCACGAGCACAGAGGCGACTTCCCTCTCTCAGACTTTAGGCGCCACTGCAGGGTCCGGAAAAGAAAGAGAAACGGCCCAGCGCGGTCGCTTACATAACCCAGGGCGGGGCTCCGCTCCGCCCCCGAAAGTTTTTGCGACGGAGTTTTCCCTCTTGTTGCCCAGGCTGAAGTGCAATGGCGCGATCTCGGCTCACCGCAACCTCCGCCTCCAAGGTTTAAGCGATTCTCCTGCCTCAGCCTCCCGACTAGCTGGGATTACAGGCATTCACCACCAAGCCCGTCTAATTTTATATTTTTAGTAGAGACGGGATTTCCCCATGTTGGTCAGGTTGGTCTCGAGCTCCCGACCTCAGGTGATCGCCCGCCTCGACCTCCCAAAGTGCTGGGATTACAGGCGTGAACCACCGTGCCCGGCCTTGGCCGCATGTGTTTTGAATTTTGCTGCCCGGAATTCACTGCGAGGACTGGGATCACCCGTCACCCCGCCCTGGTCTACGGAAAATGAAATGTGTTTACTGATATAGAAACGGAATAACGGCGCTGTGGGCTGGGGAGGGCCGAGCTGCCTTCAGGCTTCTGGTCTCCAGCTGCAGGGCACTCACACCTGCCCGTTATGAAAATGCAGACCCGCAGGGCAGGAATTCCGAGTCCGGGCTGGAGCGCGATCTGGAATCCGGCTCTCTTGAAACAGCACCGCGGAGGATTCGGATCCGGGTGAGTAGGAAACTGNNCCTCAGCCCCTCCCACGGGCCGCCNACGTATTCCAGGATCCGAAAACGCTTCCTGCTGCTCCGTNNCCCAGGAAGGCANGTCCGCCTCTGGNNGNTCTGATGGAAACCGCNTGNGCCGCCCGCAGAAAACCCNAACTAAGGGCCAGAAAAAGCTCTTAGGTNCCGCACTTCNNNNGGACCTAATTACCCNTTAGTTGGCCTCCTGAAAAANTGGAGGACCTTCCTGAANNANANNNGGNCCNGGNNNNNGGNNNNNNTAAANNNNGGGGAANNGNNCCCNNNCNGCNTNNC

Reverse Complement Reverse CO9567: ACGCCCTTTTAAATATCCG

CAGTTTCCTACTCACCCGGATCCGAATCCTCCGCGGTGCTGTTTCAAGAGAGCCGGATTCCAGATCGCGCTCCAGCCCGGACTCGGAATTCCTGCCCTGCGGGTCTGCATTTTCATAACGGGCAGGTGTGAGTGCCCTGCAGCTGGAGACCAGAAGCCTGAAGGCAGCTCGGCCCTCCCCAGCCCACAGCGCCGTTATTCCGTTTCTATATCAGTAAACACATTTCATTTTCCGTAGACCAGGGCGGGGTGACGGGTGATCCCAGTCCTCGCAGTGAATTCCGGGCAGCAAAATTCAAAACACATGCGGCCAAGGCCGGGCACGGTGGTTCACGCCTGTAATCCCAGCACTTTGGGAGGTCGAGGCGGGCGATCACCTGAGGTCGGGAGCTCGAGACCAACCTGACCAACATGGGGAAATCCCGTCTCTACTAAAAATATAAAATTAGACGGGCTTGGTGGTGAATGCCTGTAATCCCAGCTAGTCGGGAGGCTGAGGCAGGAGAATCGCTTAAACCTTGGAGGCGGAGGTTGCGGTGAGCCGAGATCGCGCCATTGCACTTCAGCCTGGGCAACAAGAGGGAAAACTCCGTCGCAAAAACTTTCGGGGGCGGAGCGGAGCCCCGCCCTGGGTTATGTAAGCGACCGCGCTGGGCCGTTTCTCTTTCTTTTCCGGACCCTGCAGTGGCGCCTAAAGTCTGAGAGAGGGAAGTCGCCTCTGTGCTCGTGAGTGCATGGGGTATAAGGCAAGTGCTGAGGGAGAAAACGTAGTTGATGGGGTAGAGCAGACGGGGTTGGAGGTGGGGTGGAGGGGGAGGGCTTTGGACAGAAGACCTGGGAGGCTTGGTGGGGGAGGGGCGCCCAGGCCTGGGCACTAAGAAACAAGTCCCCTGGAGCTCAAGACCATCTCGGCCTCCCCTAGCCCAAGAGAGGACTGGCTTCATGACTCCCTGAAACCATTTCTAAATGCCTTAGAACAAACCTTGCATATTCATTATTGTTATTGAACTATTAAAAGTCTTTTTTGGGGGCGAGCTGAATCAGATCCTTTGCTGGAGCTGGCACACGGAGGAAGTCCTGGAGGGAGGGTAGACACCGTGGAGGTAAGGGCTTGGGACCTGTGTCAGGTGAGACCAAGTCGTGAGAGGTACTGCACCACGAAGTGGTTTAAACTATCAGTGTTTGACAGGATATAT

**Assembled sequence**

Insert 1

ACTGGGGTGGATGCAGTGGGCCCCACTCTGTGGTGCAGTACCTCTCACGACTTGGTCTCACGTTGTGGGGCTTAGCTTCATATTTTCAAACTGAAATATTCTCTTCCTTAACCTCCACATAAATCCAAGTTTATAATTTTTATTATTTTAAAATTTTATTTATTTTTCTGTTTTGGGGACAGGGTCTCCTCCTGTCACTCAGGCTGGAGTGCAATGGCACAATCATAGCTCACTGCAGCCTGGAACTCCTGGGCTTAAGCAATCTTCCTGCCTTCGATTCCCAAAGAGCTGGGATTATAGTCATGAACCACTGCAATCCACCCAAATCCAAGTTTACACTAAAAGATAAAATTCCAACATTGTAGGGGATTGGTCAGGTGGTGGGAATAATTATAAAGATAAAGTTATAGGAAATAGACACAAACCTTCTTGGAAGGTGGAAAGTTTTGCAAAAGCCTCAGGATAGGGTTATAGCTGAAAGCAGCCTAATCCCCTTACCTTGAGTTAATAGCTTCGAGTAAGTACAAAGACATGTAAGAGAGTTTATCTAAAGAGCATGTTTACCTTTGATCATTTGTAGGACTGCTCTCTCTGGGGGACTGCGACCAGATTAATTACCCACAGGTGTGTTGACTCAAAGCCTTTGTCATTAAATCTGTGCTGAATAAAGGCCCACAGGGCCAGATAGTCAGGGCACGCAGCTGCCACAACCCTTTCTGTGAGTGGCCTGGCCCTCTGGTGCACTCTTTCACTGAATATCGGTGTCTGAGTACATTATTCATCCATCGTGCAGCCTGGGTCTGCCGGTCAGACCCTGGCACAACATTTAAGAGGAAATGAAAGTCACAAAGTTATCCCAGTCTCTGGAGTCACTGTCAAAACTTTGGTGAGGAATCTTCCAGGTTTTCCCCTACTTCAAATATATATTAATATTATGTAAGTGATATTAGTGGCATTTTCGCC

Inserts 2, 3 and 4

NNNNNNNNNNNNNNNGNGCGGTGGCTCACGCCTGTAATCCCAGCACTTTGAGAGGCCAAGGCAGGCAGATCACGAGGTCAGGAGATCGAGACCATCCTGGCTAACACAGTGAAACCCCGTCTCTACTAAAAATACAAAAAATTAGTCGGGCGTGGTGGCAGGAGCCTGTAGTCCCAGCTACTCCAGAGGCTGAGGCAGCAGAATGCCCTGAACCCGGGAGGCGGAGCTTGCAGTGAGCCAAGATCGCGCCACTGCACTCCAGCCTGGGCGACAGAGCAAGACTCCGTCTCAAAAAAAAAAAACAAAAAACAAAAAAACTCTCCTTTACTTTTTCTCTCCCCTTTTCTTCCTATCTCTTCCCTCATTTCTTCAACACGTCCCCCCATCCTTCCCTCTTTTCTCCATTCTCTGCATTTGATCCCCGGTATATTCCAGCCTCCAGGCCAACAAACTTCTCCGCGTCCGCCGGGAGCAGGTCAGGGAAGGGACGCGAGGCGGCGCTGTCACCGCATTCTGAGCGCCGCAGCTCCCTGGGCCCCTTGTATCATTTCAGTGAAGGTCACTCCAGTCTTTCATGGAGGCCAAACTAAGGGTGTAAATTAGGATCCTCACTGAAGTGGCGGGACCCTAAGAGGCTTTTTCCTGGCCCCTTAGTTGTGGGTTTTCCTGCGGGCGGCGCAGCCGGTTTCCATCAGAACCGCCCAGAGGCGGACGCTGCCTTCCTGGGGTGACGGAGCAGCAGGAAGCGTTTTCGGATCCTGGAATACGTGGGCGGCCCGTGTGAGGGGCTGAGGCGCAGTTTCCTACTCACCCGGATCCGAATCCTCCGCGGTGCTGTTTCAAGAGAGCCGGATTCCAGATCGCGCTCCAGCCCGGACTCGGAATTCCTGCCCTGCGGGTCTGCATTTTCATAACGGGCAGGTGTGAGTGCCCTGCAGCTGGAGACCAGAAGCCTGAAGGCAGCTCGGCCCTCCCCAGCCCACAGCGCCGTTATTCCGTTTCTATATCAGTAAACACATTTCATTTTCCGTAGACCAGGGCGGGGTGACGGGTGATCCCAGTCCTCGCAGTGAATTCCGGGCAGCAAAATTCAAAACACATGCGGCCAAGGCCGGGCACGGTGGTTCACGCCTGTAATCCCAGCACTTTGGGAGGTCGAGGCGGGCGATCACCTGAGGTCGGGAGCTCGAGACCAACCTGACCAACATGGGGAAATCCCGTCTCTACTAAAAATATAAAATTAGACGGGCTTGGTGGTGAATGCCTGTAATCCCAGCTAGTCGGGAGGCTGAGGCAGGAGAATCGCTTAAACCTTGGAGGCGGAGGTTGCGGTGAGCCGAGATCGCGCCATTGCACTTCAGCCTGGGCAACAAGAGGGAAAACTCCGTCGCAAAAACTTTCGGGGGCGGAGCGGAGCCCCGCCCTGGGTTATGTAAGCGACCGCGCTGGGCCGTTTCTCTTTCTTTTCCGGACCCTGCAGTGGCGCCTAAAGTCTGAGAGAGGGAAGTCGCCTCTGTGCTCGTGAGTGCATGGGGTATAAGGCAAGTGCTGAGGGAGAAAACGTAGTTGATGGGGTAGAGCAGACGGGGTTGGAGGTGGGGTGGAGGGGGAGGGCTTTGGACAGAAGACCTGGGAGGCTTGGTGGGGGAGGGGCGCCCAGGCCTGGGCACTAAGAAACAAGTCCCCTGGAGCTCAAGACCATCTCGGCCTCCCCTAGCCCAAGAGAGGACTGGCTTCATGACTCCCTGAAACCATTTCTAAATGCCTTAGAACAAACCTTGCATATTCATTATTGTTATTGAACTATTAAAAGTCTTTTTTGGGGGCGAGCTGAATCAGATCCTTTGCTGGAGCTGGCACACGGAGGAAGTCCTGGAGGGAGGGTAGACACCGTGGAGGTAAGGGCTTGGGACCTGTGTCAGGTGAGACCAAGTCGTGAGAGGTACTGCACCACGAAGTGGTTTAAACTATCAGTGTTTGACAGGATATAT

# POC1431 sequences from five white colonies using the methylases M.Osp807II and M2.Eco31I_2

## Colony 1

Forward CO9566: TGGTGTAAACAAATTGACGC

NNNNNNNNNTTNNGGACGTTTTTATGTACTGGGGTGGATGCAGTGGGCCCCACTCTGTGGTGCAGTACCTCTCACGACTTGGTCTCACGTTGTGGGGCTTAGCTTCATATTTTCAAACTGAAATATTCTCTTCCTTAACCTCCACATAAATCCAAGTTTATAATTTTTATTATTTTAAAATTTTATTTATTTTTCTGTTTTGGGGACAGGGTCTCCTCCTGTCACTCAGGCTGGAGTGCAATGGCACAATCATAGCTCACTGCAGCCTGGAACTCCTGGGCTTAAGCAATCTTCCTGCCTTCGATTCCCAAAGAGCTGGGATTATAGTCATGAACCACTGCAATCCACCCAAATCCAAGTTTACACTAAAAGATAAAATTCCAACATTGTAGGGGATTGGTCAGGTGGTGGGAATAATTATAAAGATAAAGTTATAGGAAATAGACACAAACCTTCTTGGAAGGTGGAAAGTTTTGCAAAAGCCTCAGGATAGGGTTATAGCTGAAAGCAGCCTAATCCCCTTACCTTGAGTTAATAGCTTCGAGTAAGTACAAAGACATGTAAGAGAGTTTATCTAAAGAGCATGTTTACCTTTGATCATTTGTAGGACTGCTCTCTCTGGGGGACTGCGACCAGATTAATTACCCACAGGTGTGTTGACTCAAAGCCTTTGTCATTAAATCTGTGCTGAATAAAGGCCCACAGGGCCAGATAGTCAGGGCACGCAGCTGCCACAACCCTTTCTGTGAGTGGCCTGGCCCTCTGGTGCACTCTTTCACTGAATATCGGTGTCTGAGTACATTATTCATCCATCGTGCAGCCTGGGTCTGCCGGTCAGACCCTGGCACAACATTTAAGAGGAAATGAAAGTCACAAAGTTATCCCAGTCTCTGGAGTCACTGTCAAAACTTTGGTGAGGAATCTTCCAGGTTTTCCCCTACTTCAAATATATATTAATATTATGTAAGTGATATTAGTGGCATTTTCGCCC

Forward CO9574: AGAAATAATGAAACTACGTC

NNNNNNNCNGNNNNNNGCGGNGGCTCACGCCTGTAATCCCAGCACTTTGAGAGGCCAAGGCAGGCAGATCACGAGGTCAGGAGATCGAGACCATCCTGGCTAACACAGTGAAACCCCGTCTCTACTAAAAATACAAAAAATTAGTCGGGCGTGGTGGCAGGAGCCTGTAGTCCCAGCTACTCCAGAGGCTGAGGCAGCAGAATGCCCTGAACCCGGGAGGCGGAGCTTGCAGTGAGCCAAGATCGCGCCACTGCACTCCAGCCTGGGCGACAGAGCAAGACTCCGTCTCAAAAAAAAAAAACAAAAAACAAAAAAACTCTCCTTTACTTTTTCTCTCCCCTTTTCTTCCTATCTCTTCCCTCATTTCTTCAACACGTCCCCCCATCCTTCCCTCTTTTCTCCATTCTCTGCATTTGATCCCCGGTATATTCCAGCCTCCAGGCCAACAAACTTCTCCGCGTCCGCCGGGAGCAGGTCAGGGAAGGGACGCGAGGCGGCGCTGTCACCGCATTCTGAGCGCCGCAGCTCCCTGGGCCCCTTGTATCATTTCAGTGAAGGTCACTCCAGTCTTTCATGGAGGCCAAACTAAGGGTGTAAATTAGGATCCTCACTGAAGTGGCGGGACCCTAAGAGGCTTTTTCCTGGCCCCTTAGTTGTGGGTTTTCCTGCGGGCGGCGCAGCCGGTTTCCATCAGAACCGCCCAGAGGCGGACGCTGCCTTCCTGGGGTGACGGAGCAGCAGGAAGCGTTTTCGGATCCTGGAATACGTGGGCGGCCCGTGGGAGGNGCTGAGGCGCAGNTTCCTACTCACCCGGATCCGAATCCTCCGCGGNGCTGTTTCAAGAGAGNNGGATTCCAGATCGCGCTCCAGCCCGGACTCNNAATTCTTGNCCTGNGGGGCTGNATTTT

Reverse CO9567: ACGCCCTTTTAAATATCCG

NNNNNNNTTTCTCTTANNGNTTACCCGCCNATATATCCTGTCAAACACTGATAGTTTAAACCACTTCGTGGTGCAGTACCTCTCACGACTTGGTCTCACCTGACACAGGTCCCAAGCCCTTACCTCCACGGTGTCTACCCTCCCTCCAGGACTTCCTCCGTGTGCCAGCTCCAGCAAAGGATCTGATTCAGCTCGCCCCCAAAAAAGACTTTTAATAGTTCAATAACAATAATGAATATGCAAGGTTTGTTCTAAGGCATTTAGAAATGGTTTCAGGGAGTCATGAAGCCAGTCCTCTCTTGGGCTAGGGGAGGCCGAGATGGTCTTGAGCTCCAGGGGACTTGTTTCTTAGTGCCCAGGCCTGGGCGCCCCTCCCCCACCAAGCCTCCCAGGTCTTCTGTCCAAAGCCCTCCCCCTCCACCCCACCTCCAACCCCGTCTGCTCTACCCCATCAACTACGTTTTCTCCCTCAGCACTTGCCTTATACCCCATGCACTCACGAGCACAGAGGCGACTTCCCTCTCTCAGACTTTAGGCGCCACTGCAGGGTCCGGAAAAGAAAGAGAAACGGCCCAGCGCGGTCGCTTACATAACCCAGGGCGGGGCTCCGCTCCGCCCCCGAAAGTTTTTGCGACGGAGTTTTCCCTCTTGTTGCCCAGGCTGAAGTGCAATGGCGCGATCTCGGCTCACCGCAACCTCCGCCTCCAAGGTTTAAGCGATTCTCCTGCCTCAGCCTCCCGACTAGCTGGGATTACAGGCATTCACCACCAAGCCCGTCTAATTTTATATTTTTAGTAGAGACGGGATTTCCTCATGTTGGTCAGGTTGGTCTCGAGCTCCCGACCTCAGGTGATCGCCCGCCTCGACCTCCCAAAGTGCTGGGATTACAGGCGTGAACCACCGTGCCCGGCCTTGGCCGCATGTGTTTTGAATTTTGCTGCCCGAAATTCACTGNTAGGACTGGGATCACCCGTCACCCCGCCCTGCNCTACGGAAAATGAAATGTGTTTACTGATCTAGAAAACGGATTACCGGCNCTGTGGGCTTGTGTAGNTCNGAGCTGCNNTCAGNCTTCTGNTTNNTAGCTNTTGCAACTCCCCNTTGCCNNNNGGANAANNNNGACCCNNNGGNNTGAANTCCNA

Reverse Complement Reverse CO9567: ACGCCCTTTTAAATATCCG

CAGTGAATTTCGGGCAGCAAAATTCAAAACACATGCGGCCAAGGCCGGGCACGGTGGTTCACGCCTGTAATCCCAGCACTTTGGGAGGTCGAGGCGGGCGATCACCTGAGGTCGGGAGCTCGAGACCAACCTGACCAACATGAGGAAATCCCGTCTCTACTAAAAATATAAAATTAGACGGGCTTGGTGGTGAATGCCTGTAATCCCAGCTAGTCGGGAGGCTGAGGCAGGAGAATCGCTTAAACCTTGGAGGCGGAGGTTGCGGTGAGCCGAGATCGCGCCATTGCACTTCAGCCTGGGCAACAAGAGGGAAAACTCCGTCGCAAAAACTTTCGGGGGCGGAGCGGAGCCCCGCCCTGGGTTATGTAAGCGACCGCGCTGGGCCGTTTCTCTTTCTTTTCCGGACCCTGCAGTGGCGCCTAAAGTCTGAGAGAGGGAAGTCGCCTCTGTGCTCGTGAGTGCATGGGGTATAAGGCAAGTGCTGAGGGAGAAAACGTAGTTGATGGGGTAGAGCAGACGGGGTTGGAGGTGGGGTGGAGGGGGAGGGCTTTGGACAGAAGACCTGGGAGGCTTGGTGGGGGAGGGGCGCCCAGGCCTGGGCACTAAGAAACAAGTCCCCTGGAGCTCAAGACCATCTCGGCCTCCCCTAGCCCAAGAGAGGACTGGCTTCATGACTCCCTGAAACCATTTCTAAATGCCTTAGAACAAACCTTGCATATTCATTATTGTTATTGAACTATTAAAAGTCTTTTTTGGGGGCGAGCTGAATCAGATCCTTTGCTGGAGCTGGCACACGGAGGAAGTCCTGGAGGGAGGGTAGACACCGTGGAGGTAAGGGCTTGGGACCTGTGTCAGGTGAGACCAAGTCGTGAGAGGTACTGCACCACGAAGTGGTTTAAACTATCAGTGTTTGACAGGATATAT

**Assembled sequence**

Insert 1

GGACGTTTTTATGTACTGGGGTGGATGCAGTGGGCCCCACTCTGTGGTGCAGTACCTCTCACGACTTGGTCTCACGTTGTGGGGCTTAGCTTCATATTTTCAAACTGAAATATTCTCTTCCTTAACCTCCACATAAATCCAAGTTTATAATTTTTATTATTTTAAAATTTTATTTATTTTTCTGTTTTGGGGACAGGGTCTCCTCCTGTCACTCAGGCTGGAGTGCAATGGCACAATCATAGCTCACTGCAGCCTGGAACTCCTGGGCTTAAGCAATCTTCCTGCCTTCGATTCCCAAAGAGCTGGGATTATAGTCATGAACCACTGCAATCCACCCAAATCCAAGTTTACACTAAAAGATAAAATTCCAACATTGTAGGGGATTGGTCAGGTGGTGGGAATAATTATAAAGATAAAGTTATAGGAAATAGACACAAACCTTCTTGGAAGGTGGAAAGTTTTGCAAAAGCCTCAGGATAGGGTTATAGCTGAAAGCAGCCTAATCCCCTTACCTTGAGTTAATAGCTTCGAGTAAGTACAAAGACATGTAAGAGAGTTTATCTAAAGAGCATGTTTACCTTTGATCATTTGTAGGACTGCTCTCTCTGGGGGACTGCGACCAGATTAATTACCCACAGGTGTGTTGACTCAAAGCCTTTGTCATTAAATCTGTGCTGAATAAAGGCCCACAGGGCCAGATAGTCAGGGCACGCAGCTGCCACAACCCTTTCTGTGAGTGGCCTGGCCCTCTGGTGCACTCTTTCACTGAATATCGGTGTCTGAGTACATTATTCATCCATCGTGCAGCCTGGGTCTGCCGGTCAGACCCTGGCACAACATTTAAGAGGAAATGAAAGTCACAAAGTTATCCCAGTCTCTGGAGTCACTGTCAAAACTTTGGTGAGGAATCTTCCAGGTTTTCCCCTACTTCAAATATATATTAATATTATGTAAGTGATATTAGTGGCATTTTCGCCC

Inserts 2 and 3

GGCTCACGCCTGTAATCCCAGCACTTTGAGAGGCCAAGGCAGGCAGATCACGAGGTCAGGAGATCGAGACCATCCTGGCTAACACAGTGAAACCCCGTCTCTACTAAAAATACAAAAAATTAGTCGGGCGTGGTGGCAGGAGCCTGTAGTCCCAGCTACTCCAGAGGCTGAGGCAGCAGAATGCCCTGAACCCGGGAGGCGGAGCTTGCAGTGAGCCAAGATCGCGCCACTGCACTCCAGCCTGGGCGACAGAGCAAGACTCCGTCTCAAAAAAAAAAAACAAAAAACAAAAAAACTCTCCTTTACTTTTTCTCTCCCCTTTTCTTCCTATCTCTTCCCTCATTTCTTCAACACGTCCCCCCATCCTTCCCTCTTTTCTCCATTCTCTGCATTTGATCCCCGGTATATTCCAGCCTCCAGGCCAACAAACTTCTCCGCGTCCGCCGGGAGCAGGTCAGGGAAGGGACGCGAGGCGGCGCTGTCACCGCATTCTGAGCGCCGCAGCTCCCTGGGCCCCTTGTATCATTTCAGTGAAGGTCACTCCAGTCTTTCATGGAGGCCAAACTAAGGGTGTAAATTAGGATCCTCACTGAAGTGGCGGGACCCTAAGAGGCTTTTTCCTGGCCCCTTAGTTGTGGGTTTTCCTGCGGGCGGCGCAGCCGGTTTCCATCAGAACCGCCCAGAGGCGGACGCTGCCTTCCTGGGGTGACGGAGCAGCAGGAAGCGTTTTCGGATCCTGGAATACGTGGGCGGCCCGTGGGAGGNGCTGAGGCGCAGNTTCCTACTCACCCGGATCCGAATCCTCCGCGGNGCTGTTTCAAGAGAGNNGGATTCCAGATCGCGCTCCAGCCCGGACTCNNAATTCTTGNCCTGNGGGGCTGNATTTT

Insert 4

CAGTGAATTTCGGGCAGCAAAATTCAAAACACATGCGGCCAAGGCCGGGCACGGTGGTTCACGCCTGTAATCCCAGCACTTTGGGAGGTCGAGGCGGGCGATCACCTGAGGTCGGGAGCTCGAGACCAACCTGACCAACATGAGGAAATCCCGTCTCTACTAAAAATATAAAATTAGACGGGCTTGGTGGTGAATGCCTGTAATCCCAGCTAGTCGGGAGGCTGAGGCAGGAGAATCGCTTAAACCTTGGAGGCGGAGGTTGCGGTGAGCCGAGATCGCGCCATTGCACTTCAGCCTGGGCAACAAGAGGGAAAACTCCGTCGCAAAAACTTTCGGGGGCGGAGCGGAGCCCCGCCCTGGGTTATGTAAGCGACCGCGCTGGGCCGTTTCTCTTTCTTTTCCGGACCCTGCAGTGGCGCCTAAAGTCTGAGAGAGGGAAGTCGCCTCTGTGCTCGTGAGTGCATGGGGTATAAGGCAAGTGCTGAGGGAGAAAACGTAGTTGATGGGGTAGAGCAGACGGGGTTGGAGGTGGGGTGGAGGGGGAGGGCTTTGGACAGAAGACCTGGGAGGCTTGGTGGGGGAGGGGCGCCCAGGCCTGGGCACTAAGAAACAAGTCCCCTGGAGCTCAAGACCATCTCGGCCTCCCCTAGCCCAAGAGAGGACTGGCTTCATGACTCCCTGAAACCATTTCTAAATGCCTTAGAACAAACCTTGCATATTCATTATTGTTATTGAACTATTAAAAGTCTTTTTTGGGGGCGAGCTGAATCAGATCCTTTGCTGGAGCTGGCACACGGAGGAAGTCCTGGAGGGAGGGTAGACACCGTGGAGGTAAGGGCTTGGGACCTGTGTCAGGTGAGACCAAGTCGTGAGAGGTACTGCACCACGAAGTGGTTTAAACTATCAGTGTTTGACAGGATATAT

## Colony 2

Forward CO9566: TGGTGTAAACAAATTGACGC

NNNNNNNNNTNNNGGACGTTTTTATGNACTGGGGTGGATGCAGTGGGCCCCACTCTGTGGTGCAGTACCTCTCACGACTTGGTCTCACGTTGTGGGGCTTAGCTTCATATTTTCAAACTGAAATATTCTCTTCCTTAACCTCCACATAAATCCAAGTTTATAATTTTTATTATTTTAAAATTTTATTTATTTTTCTGTTTTGGGGACAGGGTCTCCTCCTGTCACTCAGGCTGGAGTGCAATGGCACAATCATAGCTCACTGCAGCCTGGAACTCCTGGGCTTAAGCAATCTTCCTGCCTTCGATTCCCAAAGAGCTGGGATTATAGTCATGAACCACTGCAATCCACCCAAATCCAAGTTTACACTAAAAGATAAAATTCCAACATTGTAGGGGATTGGTCAGGTGGTGGGAATAATTATAAAGATAAAGTTATAGGAAATAGACACAAACCTTCTTGGAAGGTGGAAAGTTTTGCAAAAGCCTCAGGATAGGGTTATAGCTGAAAGCAGCCTAATCCCCTTACCTTGAGTTAATAGCTTCGAGTAAGTACAAAGACATGTAAGAGAGTTTATCTAAAGAGCATGTTTACCTTTGATCATTTGTAGGACTGCTCTCTCTGGGGGACTGCGACCAGATTAATTACCCACAGGTGTGTTGACTCAAAGCCTTTGTCATTAAATCTGTGCTGAATAAAGGCCCACAGGGCCAGATAGTCAGGGCACGCAGCTGCCACAACCCTTTCTGTGAGTGGCCTGGCCCTCTGGTGCACTCTTTCACTGAATATCGGTGTCTGAGTACATTATTCATCCATCGTGCAGCCTGGGTCTGCCGGTCAGACCCTGGCACAACATTTAAGAGGAAATGAAAGTCACAAAGTTATCCCAGTCTCTGGAGTCACTGTCAAAACTTTGGTGAGGAATCTTCCAGGTTTTCCCCTACTTCAAATATATATTAATATTATGTAAGTGATATTAGTGGCATTTTCGCCC

Forward CO9574: AGAAATAATGAAACTACGTC

NNNNNNNNNCGNNNNGGNGCGGNGGCTCACGCCTGTAATCCCAGCACTTTGAGAGGCCAAGGCAGGCAGATCACGAGGTCAGGAGATCGAGACCATCCTGGCTAACACAGTGAAACCCCGTCTCTACTAAAAATACAAAAAATTAGTCGGGCGTGGTGGCAGGAGCCTGTAGTCCCAGCTACTCCAGAGGCTGAGGCAGCAGAATGCCCTGAACCCGGGAGGCGGAGCTTGCAGTGAGCCAAGATCGCGCCACTGCACTCCAGCCTGGGCGACAGAGCAAGACTCCGTCTCAAAAAAAAAAAACAAAAAACAAAAAAACTCTCCTTTACTTTTTCTCTCCCCTTTTCTTCCTATCTCTTCCCTCATTTCTTCAACACGTCCCCCCATCCTTCCCTCTTTTCTCCATTCTCTGCATTTGATCCCCGGTATATTCCAGCCTCCAGGCCAACAAACTTCTCCGCGTCCGCCGGGAGCAGGTCAGGGAAGGGACGCGAGGCGGCGCTGTCACCGCATTCTGAGCGCCGCAGCTCCCTGGGCCCCTTGTATCATTTCAGTGAAGGTCACTCCAGTCTTTCATGGAGGCCAAACTAAGGGTGTAAATTAGGATCCTCACTGAAGTGGCGGGACCCTAAGAGGCTTTTTCCTGGCCCCTTAGTTGTGGGTTTTCCTGCGGGCGGCGCAGCCGGTTTCCATCAGAACCGCCCAGAGGCGGACGCTGCCTTCCTGGGGTGACGGAGCAGCAGGAAGCGTTTTCGGATCCTGGAATACGTGGGCGGCCCGTGCGAGGGGCTGAGGCGCAGTTTCCTACTCACCCGGATCCGAATCCTCCGCGGTGCTGTTTCAAGAGAGCCGGATTCCAGATCGCGCTCCAGCCCGGACTCGGAATTCCTGCCCTGCGGGTCTGCATTTTCATAACGGGCAGGTGTGAGTGCCCTGCAGCTGCAGACCAGAAGCCTGAAGGCAGCTCGGCCCTCCCCAGCCCACAGCGCCGTTATTCCGTTTCTATATCAGTAAACACATTTCATTTTCCGTANACCAGGGCGGNGTGACGGGTGATCCCAGTCCTCGCAGTGAATCCGGNCNGCAANNTCAAANNTCATGNNGCCNAAGNCGGCACAGNNGTTCCC

Reverse CO9567: ACGCCCTTTTAAATATCCG

NNNNNNNNNCTCTTAGGNTTACCCGCCAATATATCCTGTCAAACACTGATAGTTTAAACCACTTCGTGGTGCAGTACCTCTCACGACTTGGTCTCACCTGACACAGGTCCCAAGCCCTTACCTCCACGGTGTCTACCCTCCCTCCAGGACTTCCTCCGTGTGCCAGCTCCAGCAAAGGATCTGATTCAGCTCGCCCCCAAAAAAGACTTTTAATAGTTCAATAACAATAATGAATATGCAAGGTTTGTTCTAAGGCATTTAGAAATGGTTTCAGGGAGTCATGAAGCCAGTCCTCTCTTGGGCTAGGGGAGGCCGAGATGGTCTTGAGCTCCAGGGGACTTGTTTCTTAGTGCCCAGGCCTGGGCGCCCCTCCCCCACCAAGCCTCCCAGGTCTTCTGTCCAAAGCCCTCCCCCTCCACCCCACCTCCAACCCCGTCTGCTCTACCCCATCAACTACGTTTTCTCCCTCAGCACTTGCCTTATACCCCATGCACTCACGAGCACAGAGGCGACTTCCCTCTCTCAGACTTTAGGCGCCACTGCAGGGTCCGGAAAAGAAAGAGAAACGGCCCAGCGCGGTCGCTTACATAACCCAGGGCGGGGCTCCGCTCCGCCCCCGAAAGTTTTTGCGACGGAGTTTTCCCTCTTGTTGCCCAGGCTGAAGTGCAATGGCGCGATCTCGGCTCACCGCAACCTCCGCCTCCAAGGTTTAAGCGATTCTCCTGCCTCAGCCTCCCGACTAGCTGGGATTACAGGCATTCACCACCAAGCCCGTCTAATTTTATATTTTTAGTAGAGACGGGATTTCCCCATGTTGGTCAGGTTGGTCTCGAGCTCCCGACCTCAGGTGATCGCCCGCCTCGACCTCCCAAAGTGCTGGGATTACAGGCGTGAACCACCGTGCCCGGCCTTGGCCGCATGTGTTTTGAATTTTGCTGCCCGGAATTCACTGCGAGGACTGGGATCACCCGTCACCCCGCCCTGGTCTACGGAAAATGAAATGTGTTTACTGATATAGAAACGGAATAACGGCGCTGTGGGCTGGGGAGGGCCGAGCTGCCTTCAGGCTTCTGGTCTCCAGCTGCAGGGCACTCACACCTGCCCGTTATGAAAATGCAGACCCGCAGGGCAGGAATTCCGAGTCCGGGCTGGAGCGCGATCTGGAANCCGGCTCTCTTGAAANNNCACCGCGGAGGATTCGGATCCGGNNGANNAAGAAACTGCGCCTNAGCCCNTCCCACGGGCCGCCN

Reverse Complement Reverse CO9567: ACGCCCTTTTAAATATCCG

TTCCAGATCGCGCTCCAGCCCGGACTCGGAATTCCTGCCCTGCGGGTCTGCATTTTCATAACGGGCAGGTGTGAGTGCCCTGCAGCTGGAGACCAGAAGCCTGAAGGCAGCTCGGCCCTCCCCAGCCCACAGCGCCGTTATTCCGTTTCTATATCAGTAAACACATTTCATTTTCCGTAGACCAGGGCGGGGTGACGGGTGATCCCAGTCCTCGCAGTGAATTCCGGGCAGCAAAATTCAAAACACATGCGGCCAAGGCCGGGCACGGTGGTTCACGCCTGTAATCCCAGCACTTTGGGAGGTCGAGGCGGGCGATCACCTGAGGTCGGGAGCTCGAGACCAACCTGACCAACATGGGGAAATCCCGTCTCTACTAAAAATATAAAATTAGACGGGCTTGGTGGTGAATGCCTGTAATCCCAGCTAGTCGGGAGGCTGAGGCAGGAGAATCGCTTAAACCTTGGAGGCGGAGGTTGCGGTGAGCCGAGATCGCGCCATTGCACTTCAGCCTGGGCAACAAGAGGGAAAACTCCGTCGCAAAAACTTTCGGGGGCGGAGCGGAGCCCCGCCCTGGGTTATGTAAGCGACCGCGCTGGGCCGTTTCTCTTTCTTTTCCGGACCCTGCAGTGGCGCCTAAAGTCTGAGAGAGGGAAGTCGCCTCTGTGCTCGTGAGTGCATGGGGTATAAGGCAAGTGCTGAGGGAGAAAACGTAGTTGATGGGGTAGAGCAGACGGGGTTGGAGGTGGGGTGGAGGGGGAGGGCTTTGGACAGAAGACCTGGGAGGCTTGGTGGGGGAGGGGCGCCCAGGCCTGGGCACTAAGAAACAAGTCCCCTGGAGCTCAAGACCATCTCGGCCTCCCCTAGCCCAAGAGAGGACTGGCTTCATGACTCCCTGAAACCATTTCTAAATGCCTTAGAACAAACCTTGCATATTCATTATTGTTATTGAACTATTAAAAGTCTTTTTTGGGGGCGAGCTGAATCAGATCCTTTGCTGGAGCTGGCACACGGAGGAAGTCCTGGAGGGAGGGTAGACACCGTGGAGGTAAGGGCTTGGGACCTGTGTCAGGTGAGACCAAGTCGTGAGAGGTACTGCACCACGAAGTGGTTTAAACTATCAGTGTTTGACAGGATATATTGGCGGGTAA

**Assembled sequence**

Insert 1

ACTGGGGTGGATGCAGTGGGCCCCACTCTGTGGTGCAGTACCTCTCACGACTTGGTCTCACGTTGTGGGGCTTAGCTTCATATTTTCAAACTGAAATATTCTCTTCCTTAACCTCCACATAAATCCAAGTTTATAATTTTTATTATTTTAAAATTTTATTTATTTTTCTGTTTTGGGGACAGGGTCTCCTCCTGTCACTCAGGCTGGAGTGCAATGGCACAATCATAGCTCACTGCAGCCTGGAACTCCTGGGCTTAAGCAATCTTCCTGCCTTCGATTCCCAAAGAGCTGGGATTATAGTCATGAACCACTGCAATCCACCCAAATCCAAGTTTACACTAAAAGATAAAATTCCAACATTGTAGGGGATTGGTCAGGTGGTGGGAATAATTATAAAGATAAAGTTATAGGAAATAGACACAAACCTTCTTGGAAGGTGGAAAGTTTTGCAAAAGCCTCAGGATAGGGTTATAGCTGAAAGCAGCCTAATCCCCTTACCTTGAGTTAATAGCTTCGAGTAAGTACAAAGACATGTAAGAGAGTTTATCTAAAGAGCATGTTTACCTTTGATCATTTGTAGGACTGCTCTCTCTGGGGGACTGCGACCAGATTAATTACCCACAGGTGTGTTGACTCAAAGCCTTTGTCATTAAATCTGTGCTGAATAAAGGCCCACAGGGCCAGATAGTCAGGGCACGCAGCTGCCACAACCCTTTCTGTGAGTGGCCTGGCCCTCTGGTGCACTCTTTCACTGAATATCGGTGTCTGAGTACATTATTCATCCATCGTGCAGCCTGGGTCTGCCGGTCAGACCCTGGCACAACATTTAAGAGGAAATGAAAGTCACAAAGTTATCCCAGTCTCTGGAGTCACTGTCAAAACTTTGGTGAGGAATCTTCCAGGTTTTCCCCTACTTCAAATATATATTAATATTATGTAAGTGATATTAGTGGCATTTTCGCCC

Inserts 2, 3 and 4

GCGGNGGCTCACGCCTGTAATCCCAGCACTTTGAGAGGCCAAGGCAGGCAGATCACGAGGTCAGGAGATCGAGACCATCCTGGCTAACACAGTGAAACCCCGTCTCTACTAAAAATACAAAAAATTAGTCGGGCGTGGTGGCAGGAGCCTGTAGTCCCAGCTACTCCAGAGGCTGAGGCAGCAGAATGCCCTGAACCCGGGAGGCGGAGCTTGCAGTGAGCCAAGATCGCGCCACTGCACTCCAGCCTGGGCGACAGAGCAAGACTCCGTCTCAAAAAAAAAAAACAAAAAACAAAAAAACTCTCCTTTACTTTTTCTCTCCCCTTTTCTTCCTATCTCTTCCCTCATTTCTTCAACACGTCCCCCCATCCTTCCCTCTTTTCTCCATTCTCTGCATTTGATCCCCGGTATATTCCAGCCTCCAGGCCAACAAACTTCTCCGCGTCCGCCGGGAGCAGGTCAGGGAAGGGACGCGAGGCGGCGCTGTCACCGCATTCTGAGCGCCGCAGCTCCCTGGGCCCCTTGTATCATTTCAGTGAAGGTCACTCCAGTCTTTCATGGAGGCCAAACTAAGGGTGTAAATTAGGATCCTCACTGAAGTGGCGGGACCCTAAGAGGCTTTTTCCTGGCCCCTTAGTTGTGGGTTTTCCTGCGGGCGGCGCAGCCGGTTTCCATCAGAACCGCCCAGAGGCGGACGCTGCCTTCCTGGGGTGACGGAGCAGCAGGAAGCGTTTTCGGATCCTGGAATACGTGGGCGGCCCGTGCGAGGGGCTGAGGCGCAGTTTCCTACTCACCCGGATCCGAATCCTCCGCGGTGCTGTTTCAAGAGAGCCGGATTCCAGATCGCGCTCCAGCCCGGACTCGGAATTCCTGCCCTGCGGGTCTGCATTTTCATAACGGGCAGGTGTGAGTGCCCTGCAGCTGGAGACCAGAAGCCTGAAGGCAGCTCGGCCCTCCCCAGCCCACAGCGCCGTTATTCCGTTTCTATATCAGTAAACACATTTCATTTTCCGTAGACCAGGGCGGGGTGACGGGTGATCCCAGTCCTCGCAGTGAATTCCGGGCAGCAAAATTCAAAACACATGCGGCCAAGGCCGGGCACGGTGGTTCACGCCTGTAATCCCAGCACTTTGGGAGGTCGAGGCGGGCGATCACCTGAGGTCGGGAGCTCGAGACCAACCTGACCAACATGGGGAAATCCCGTCTCTACTAAAAATATAAAATTAGACGGGCTTGGTGGTGAATGCCTGTAATCCCAGCTAGTCGGGAGGCTGAGGCAGGAGAATCGCTTAAACCTTGGAGGCGGAGGTTGCGGTGAGCCGAGATCGCGCCATTGCACTTCAGCCTGGGCAACAAGAGGGAAAACTCCGTCGCAAAAACTTTCGGGGGCGGAGCGGAGCCCCGCCCTGGGTTATGTAAGCGACCGCGCTGGGCCGTTTCTCTTTCTTTTCCGGACCCTGCAGTGGCGCCTAAAGTCTGAGAGAGGGAAGTCGCCTCTGTGCTCGTGAGTGCATGGGGTATAAGGCAAGTGCTGAGGGAGAAAACGTAGTTGATGGGGTAGAGCAGACGGGGTTGGAGGTGGGGTGGAGGGGGAGGGCTTTGGACAGAAGACCTGGGAGGCTTGGTGGGGGAGGGGCGCCCAGGCCTGGGCACTAAGAAACAAGTCCCCTGGAGCTCAAGACCATCTCGGCCTCCCCTAGCCCAAGAGAGGACTGGCTTCATGACTCCCTGAAACCATTTCTAAATGCCTTAGAACAAACCTTGCATATTCATTATTGTTATTGAACTATTAAAAGTCTTTTTTGGGGGCGAGCTGAATCAGATCCTTTGCTGGAGCTGGCACACGGAGGAAGTCCTGGAGGGAGGGTAGACACCGTGGAGGTAAGGGCTTGGGACCTGTGTCAGGTGAGACCAAGTCGTGAGAGGTACTGCACCACGAAGTGGTTTAAACTATCAGTGTTTGACAGGATATATTGGCGGGTAA

## Colony 3

Forward CO9566: TGGTGTAAACAAATTGACGC

NNNNNNNNNTNNGGACGTTTTTATGTACTGGGGTGGATGCAGTGGGCCCCACTCTGTGGTGCAGTACCTCTCACGACTTGGTCTCACGTTGTGGGGCTTAGCTTCATATTTTCAAACTGAAATATTCTCTTCCTTAACCTCCACATAAATCCAAGTTTATAATTTTTATTATTTTAAAATTTTATTTATTTTTCTGTTTTGGGGACAGGGTCTCCTCCTGTCACTCAGGCTGGAGTGCAATGGCACAATCATAGCTCACTGCAGCCTGGAACTCCTGGGCTTAAGCAATCTTCCTGCCTTCGATTCCCAAAGAGCTGGGATTATAGTCATGAACCACTGCAATCCACCCAAATCCAAGTTTACACTAAAAGATAAAATTCCAACATTGTAGGGGATTGGTCAGGTGGTGGGAATAATTATAAAGATAAAGTTATAGGAAATAGACACAAACCTTCTTGGAAGGTGGAAAGTTTTGCAAAAGCCTCAGGATAGGGTTATAGCTGAAAGCAGCCTAATCCCCTTACCTTGAGTTAATAGCTTCGAGTAAGTACAAAGACATGTAAGAGAGTTTATCTAAAGAGCATGTTTACCTTTGATCATTTGTAGGACTGCTCTCTCTGGGGGACTGCGACCAGATTAATTACCCACAGGTGTGTTGACTCAAAGCCTTTGTCATTAAATCTGTGCTGAATAAAGGCCCACAGGGCCAGATAGTCAGGGCACGCAGCTGCCACAACCCTTTCTGTGAGTGGCCTGGCCCTCTGGTGCACTCTTTCACTGAATATCGGTGTCTGAGTACATTATTCATCCATCGTGCAGCCTGGGTCTGCCGGTCAGACCCTGGCACAACATTTAAGAGGAAATGAAAGTCACAAAGTTATCCCAGTCTCTGGAGTCACTGTCAAAACTTTGGTGAGGAATCTTCCAGGTTTTCCCCTACTTCAAATATATATTAATATTATGTAAGTGATATTAGTGGCATTTTCGCCCAGGCTGGAATGCAGTGGCATGATCTCGGCTCACTCTAACCTCTACCTCCCAGATTCAAGCGATTCTCCTGCCTCAGCCTCCCAAACAGTTGGAACTACCGGAACCCANACCANNCCNGGCTAATTTTTGCTTTNCNNNNGANACAGGGATTTNCCATGTTGGCCANNCTGATCTTNAACTTCTG

Forward CO9574: AGAAATAATGAAACTACGTC

NNNNNNNTCNNNGNGNGCGGNGGCTCACGCCTGTAATCCCAGCACTTTGAGAGGCCAAGGCAGGCAGATCACGAGGTCAGGAGATCGAGACCATCCTGGCTAACACAGTGAAACCCCGTCTCTACTAAAAATACAAAAAATTAGTCGGGCGTGGTGGCAGGAGCCTGTAGTCCCAGCTACTCCAGAGGCTGAGGCAGCAGAATGCCCTGAACCCGGGAGGCGGAGCTTGCAGTGAGCCAAGATCGCGCCACTGCACTCCAGCCTGGGCGACAGAGCAAGACTCCGTCTCAAAAAAAAAAAACAAAAAACAAAAAAACTCTCCTTTACTTTTTCTCTCCCCTTTTCTTCCTATCTCTTCCCTCATTTCTTCAACACGTCCCCCCATCCTTCCCTCTTTTCTCCATTCTCTGCATTTGATCCCCGGTATATTCCAGCCTCCAGGCCAACAAACTTCTCCGCGTCCGCCGGGAGCAGGTCAGGGAAGGGACGCGAGGCGGCGCTGTCACCGCATTCTGAGCGCCGCAGCTCCCTGGGCCCCTTGTATCATTTCAGTGAAGGTCACTCCAGTCTTTCATGGAGGCCAAACTAAGGGTGTAAATTAGGATCCTCACTGAAGTGGCGGGACCCTAAGAGGCTTTTTCCTGGCCCCTTAGTTGTGGGTTTTCCTGCGGGCGGCGCAGCCGGTTTCCATCAGAACCGCCCAGAGGCGGACGCTGCCTTCCTGGGGTGACGGAGCAGCAGGAAGCGTTTTCGGATCCTGGAATACGTGGGCGGCCCGTGGGAGGGGCTGAGGCGCAGTTTCCTACTCACCCGGATCCGAATCCTCCGCGATGCTGTTTCAAGAGAGCCGGATTCCAGATCGCGCTCCAGCCCGGACTCGGAATTCCTGCCCTGCGNGTCTGCATTTTCATAACGGGCAGGTGTGAGTGCCCTGCAGCTGGAGACCANAAGCCTGAAGGCAGCTCGGGCCTCCCCAGCCCACAGCGCCGTTATCCCGTTTCTATATCAGTNAACCCATGTCATTTTCCGTAGACCAGGNNGGGNNGAACGCTGATCCANNCCTCACNNNCGAATTCNNNGCAGTCANACNCTAANNNNA

Reverse CO9567: ACGCCCTTTTAAATATCCG

NNNNNNNTTTCTCTTAGGNTTACCCGCCNATATATCCTGTCAAACACTGATAGTTTAAACCACTTCGTGGTGCAGTACCTCTCACGACTTGGTCTCACCTGACACAGGTCCCAAGCCCTTACCTCCACGGTGTCTACCCTCCCTCCAGGACTTCCTCCGTGTGCCAGCTCCAGCAAAGGATCTGATTCAGCTCGCCCCCAAAAAAGACTTTTAATAGTTCAATAACAATAATGAATATGCAAGGTTTGTTCTAAGGCATTTAGAAATGGTTTCAGGGAGTCATGAAGCCAGTCCTCTCTTGGGCTAGGGGAGGCCGAGATGGTCTTGAGCTCCAGGGGACTTGTTTCTTAGTGCCCAGGCCTGGGCGCCCCTCCCCCACCAAGCCTCCCAGGTCTTCTGTCCAAAGCCCTCCCCCTCCACCCCACCTCCAACCCCGTCTGCTCTACCCCATCAACTACGTTTTCTCCCTCAGCACTTGCCTTATACCCCATGCACTCACGAGCACAGAGGCGACTTCCCTCTCTCAGACTTTAGGCGCCACTGCAGGGTCCGGAAAAGAAAGAGAAACGGCCCAGCGCGGTCGCTTACATAACCCAGGGCGGGGCTCCGCTCCGCCCCCGAAAGTTTTTGCGACGGAGTTTTCCCTCTTGTTGCCCAGGCTGAAGTGCAATGGCGCGATCTCGGCTCACCGCAACCTCCGCCTCCAAGGTTTAAGCGATTCTCCTGCCTCAGCCTCCCGACTAGCTGGGATTACAGGCATTCACCACCAAGCCCGTCTAATTTTATATTTTTAGTAGAGACGGGATTTCCCCATGTTGGTCAGGTTGGTCTCGAGCTCCCGACCTCAGGTGATCGCCCGCCTCGACCTCCCAAAGTGCTGGGATTACAGGCGTGAACCACCGTGCCCGGCCTTGGCCGCATGTGTTTTGAATTTTGCTGCCCGGAATTCACTGCGAGGACTGGGATCACCCGTCACCCCGCCCTGGTCTACGGAAAATGAAATGTGTTTACTGATATAGAAACGGAATAACGGCGCTGTGGGCTGGGGAGGGCCGAGCTGCCTTCAGGCTTCTGGTCTCCAGCTGCAGGGCACTCACACCTGCCCGTTATGAAAATGCAGACCCGCAGGGCAGGAATTCCGAGTCCGGGCTGGAGCGCGATCTGGAATCCGGCTCTCTTGAAACAGCACCGGCGAAGGATTCGGANCCGGGNGAATAAGAAACTGCGCCTCAGCCCCTCCCACGGGCCGCCNACGTATTCCAGGATCCGAAAANNNTTCCTGCTGNTCCGTCACCCCAGGAANGGAANGTNCCGCCTCTGGGCGGTTCTGATGAAAACCGGNNNCCCNGCCNNNAGGAAAACCCCNAAC

Reverse Complement Reverse CO9567: ACGCCCTTTTAAATATCCG

TCCGAATCCTTCGCCGGTGCTGTTTCAAGAGAGCCGGATTCCAGATCGCGCTCCAGCCCGGACTCGGAATTCCTGCCCTGCGGGTCTGCATTTTCATAACGGGCAGGTGTGAGTGCCCTGCAGCTGGAGACCAGAAGCCTGAAGGCAGCTCGGCCCTCCCCAGCCCACAGCGCCGTTATTCCGTTTCTATATCAGTAAACACATTTCATTTTCCGTAGACCAGGGCGGGGTGACGGGTGATCCCAGTCCTCGCAGTGAATTCCGGGCAGCAAAATTCAAAACACATGCGGCCAAGGCCGGGCACGGTGGTTCACGCCTGTAATCCCAGCACTTTGGGAGGTCGAGGCGGGCGATCACCTGAGGTCGGGAGCTCGAGACCAACCTGACCAACATGGGGAAATCCCGTCTCTACTAAAAATATAAAATTAGACGGGCTTGGTGGTGAATGCCTGTAATCCCAGCTAGTCGGGAGGCTGAGGCAGGAGAATCGCTTAAACCTTGGAGGCGGAGGTTGCGGTGAGCCGAGATCGCGCCATTGCACTTCAGCCTGGGCAACAAGAGGGAAAACTCCGTCGCAAAAACTTTCGGGGGCGGAGCGGAGCCCCGCCCTGGGTTATGTAAGCGACCGCGCTGGGCCGTTTCTCTTTCTTTTCCGGACCCTGCAGTGGCGCCTAAAGTCTGAGAGAGGGAAGTCGCCTCTGTGCTCGTGAGTGCATGGGGTATAAGGCAAGTGCTGAGGGAGAAAACGTAGTTGATGGGGTAGAGCAGACGGGGTTGGAGGTGGGGTGGAGGGGGAGGGCTTTGGACAGAAGACCTGGGAGGCTTGGTGGGGGAGGGGCGCCCAGGCCTGGGCACTAAGAAACAAGTCCCCTGGAGCTCAAGACCATCTCGGCCTCCCCTAGCCCAAGAGAGGACTGGCTTCATGACTCCCTGAAACCATTTCTAAATGCCTTAGAACAAACCTTGCATATTCATTATTGTTATTGAACTATTAAAAGTCTTTTTTGGGGGCGAGCTGAATCAGATCCTTTGCTGGAGCTGGCACACGGAGGAAGTCCTGGAGGGAGGGTAGACACCGTGGAGGTAAGGGCTTGGGACCTGTGTCAGGTGAGACCAAGTCGTGAGAGGTACTGCACCACGAAGTGGTTTAAACTATCAGTGTTTGACAGGATATATNGGCGGGTAA

**Assembled sequence**

Insert 1

GGACGTTTTTATGTACTGGGGTGGATGCAGTGGGCCCCACTCTGTGGTGCAGTACCTCTCACGACTTGGTCTCACGTTGTGGGGCTTAGCTTCATATTTTCAAACTGAAATATTCTCTTCCTTAACCTCCACATAAATCCAAGTTTATAATTTTTATTATTTTAAAATTTTATTTATTTTTCTGTTTTGGGGACAGGGTCTCCTCCTGTCACTCAGGCTGGAGTGCAATGGCACAATCATAGCTCACTGCAGCCTGGAACTCCTGGGCTTAAGCAATCTTCCTGCCTTCGATTCCCAAAGAGCTGGGATTATAGTCATGAACCACTGCAATCCACCCAAATCCAAGTTTACACTAAAAGATAAAATTCCAACATTGTAGGGGATTGGTCAGGTGGTGGGAATAATTATAAAGATAAAGTTATAGGAAATAGACACAAACCTTCTTGGAAGGTGGAAAGTTTTGCAAAAGCCTCAGGATAGGGTTATAGCTGAAAGCAGCCTAATCCCCTTACCTTGAGTTAATAGCTTCGAGTAAGTACAAAGACATGTAAGAGAGTTTATCTAAAGAGCATGTTTACCTTTGATCATTTGTAGGACTGCTCTCTCTGGGGGACTGCGACCAGATTAATTACCCACAGGTGTGTTGACTCAAAGCCTTTGTCATTAAATCTGTGCTGAATAAAGGCCCACAGGGCCAGATAGTCAGGGCACGCAGCTGCCACAACCCTTTCTGTGAGTGGCCTGGCCCTCTGGTGCACTCTTTCACTGAATATCGGTGTCTGAGTACATTATTCATCCATCGTGCAGCCTGGGTCTGCCGGTCAGACCCTGGCACAACATTTAAGAGGAAATGAAAGTCACAAAGTTATCCCAGTCTCTGGAGTCACTGTCAAAACTTTGGTGAGGAATCTTCCAGGTTTTCCCCTACTTCAAATATATATTAATATTATGTAAGTGATATTAGTGGCATTTTCGCCCAGGCTGGAATGCAGTGGCATGATCTCGGCTCACTCTAACCTCTACCTCCCAGATTCAAGCGATTCTCCTGCCTCAGCCTCCCAAACAGTTGGAACTACCGGAACCCA

Inserts 2, 3 and 4

GGCTCACGCCTGTAATCCCAGCACTTTGAGAGGCCAAGGCAGGCAGATCACGAGGTCAGGAGATCGAGACCATCCTGGCTAACACAGTGAAACCCCGTCTCTACTAAAAATACAAAAAATTAGTCGGGCGTGGTGGCAGGAGCCTGTAGTCCCAGCTACTCCAGAGGCTGAGGCAGCAGAATGCCCTGAACCCGGGAGGCGGAGCTTGCAGTGAGCCAAGATCGCGCCACTGCACTCCAGCCTGGGCGACAGAGCAAGACTCCGTCTCAAAAAAAAAAAACAAAAAACAAAAAAACTCTCCTTTACTTTTTCTCTCCCCTTTTCTTCCTATCTCTTCCCTCATTTCTTCAACACGTCCCCCCATCCTTCCCTCTTTTCTCCATTCTCTGCATTTGATCCCCGGTATATTCCAGCCTCCAGGCCAACAAACTTCTCCGCGTCCGCCGGGAGCAGGTCAGGGAAGGGACGCGAGGCGGCGCTGTCACCGCATTCTGAGCGCCGCAGCTCCCTGGGCCCCTTGTATCATTTCAGTGAAGGTCACTCCAGTCTTTCATGGAGGCCAAACTAAGGGTGTAAATTAGGATCCTCACTGAAGTGGCGGGACCCTAAGAGGCTTTTTCCTGGCCCCTTAGTTGTGGGTTTTCCTGCGGGCGGCGCAGCCGGTTTCCATCAGAACCGCCCAGAGGCGGACGCTGCCTTCCTGGGGTGACGGAGCAGCAGGAAGCGTTTTCGGATCCTGGAATACGTGGGCGGCCCGTGGGAGGGGCTGAGGCGCAGTTTCCTACTCACCCGGATCCGAATCCTCCGCGATGCTGTTTCAAGAGAGCCGGATTCCAGATCGCGCTCCAGCCCGGACTCGGAATTCCTGCCCTGCGGGTCTGCATTTTCATAACGGGCAGGTGTGAGTGCCCTGCAGCTGGAGACCAGAAGCCTGAAGGCAGCTCGGCCCTCCCCAGCCCACAGCGCCGTTATTCCGTTTCTATATCAGTAAACACATTTCATTTTCCGTAGACCAGGGCGGGGTGACGGGTGATCCCAGTCCTCGCAGTGAATTCCGGGCAGCAAAATTCAAAACACATGCGGCCAAGGCCGGGCACGGTGGTTCACGCCTGTAATCCCAGCACTTTGGGAGGTCGAGGCGGGCGATCACCTGAGGTCGGGAGCTCGAGACCAACCTGACCAACATGGGGAAATCCCGTCTCTACTAAAAATATAAAATTAGACGGGCTTGGTGGTGAATGCCTGTAATCCCAGCTAGTCGGGAGGCTGAGGCAGGAGAATCGCTTAAACCTTGGAGGCGGAGGTTGCGGTGAGCCGAGATCGCGCCATTGCACTTCAGCCTGGGCAACAAGAGGGAAAACTCCGTCGCAAAAACTTTCGGGGGCGGAGCGGAGCCCCGCCCTGGGTTATGTAAGCGACCGCGCTGGGCCGTTTCTCTTTCTTTTCCGGACCCTGCAGTGGCGCCTAAAGTCTGAGAGAGGGAAGTCGCCTCTGTGCTCGTGAGTGCATGGGGTATAAGGCAAGTGCTGAGGGAGAAAACGTAGTTGATGGGGTAGAGCAGACGGGGTTGGAGGTGGGGTGGAGGGGGAGGGCTTTGGACAGAAGACCTGGGAGGCTTGGTGGGGGAGGGGCGCCCAGGCCTGGGCACTAAGAAACAAGTCCCCTGGAGCTCAAGACCATCTCGGCCTCCCCTAGCCCAAGAGAGGACTGGCTTCATGACTCCCTGAAACCATTTCTAAATGCCTTAGAACAAACCTTGCATATTCATTATTGTTATTGAACTATTAAAAGTCTTTTTTGGGGGCGAGCTGAATCAGATCCTTTGCTGGAGCTGGCACACGGAGGAAGTCCTGGAGGGAGGGTAGACACCGTGGAGGTAAGGGCTTGGGACCTGTGTCAGGTGAGACCAAGTCGTGAGAGGTACTGCACCACGAAGTGGTTTAAACTATCAGTGTTTGACAGGATATATNGGCGGGTAA

## Colony 4

Forward CO9566: TGGTGTAAACAAATTGACGC

NNNNNNNNTNNNGGACGTTTTTATGNACTGGGGTGGATGCAGTGGGCCCCACTCTGTGGTGCAGTACCTCTCACGACTTGGTCTCACGTTGTGGGGCTTAGCTTCATATTTTCAAACTGAAATATTCTCTTCCTTAACCTCCACATAAATCCAAGTTTATAATTTTTATTATTTTAAAATTTTATTTATTTTTCTGTTTTGGGGACAGGGTCTCCTCCTGTCACTCAGGCTGGAGTGCAATGGCACAATCATAGCTCACTGCAGCCTGGAACTCCTGGGCTTAAGCAATCTTCCTGCCTTCGATTCCCAAAGAGCTGGGATTATAGTCATGAACCACTGCAATCCACCCAAATCCAAGTTTACACTAAAAGATAAAATTCCAACATTGTAGGGGATTGGTCAGGTGGTGGGAATAATTATAAAGATAAAGTTATAGGAAATAGACACAAACCTTCTTGGAAGGTGGAAAGTTTTGCAAAAGCCTCAGGATAGGGTTATAGCTGAAAGCAGCCTAATCCCCTTACCTTGAGTTAATAGCTTCGAGTAAGTACAAAGACATGTAAGAGAGTTTATCTAAAGAGCATGTTTACCTTTGATCATTTGTAGGACTGCTCTCTCTGGGGGACTGCGACCAGATTAATTACCCACAGGTGTGTTGACTCAAAGCCTTTGTCATTAAATCTGTGCTGAATAAAGGCCCACAGGGCCAGATAGTCAGGGCACGCAGCTGCCACAACCCTTTCTGTGAGTGGCCTGGCCCTCTGGTGCACTCTTTCACTGAATATCGGTGTCTGAGTACATTATTCATCCATCGTGCAGCCTGGGTCTGCCGGTCAGACCCTGGCACAACATTTAAGAGGAAATGAAAGTCACAAAGTTATCCCAGTCTCTGGAGTCACTGTCAAAACTTTGGTGAGGAATCTTCCAGNTTTTCCCCTACTTCACATATATATTAATATTATGTAAGTGATATTA

Forward CO9574: AGAAATAATGAAACTACGTC

NNNNNNNNNNNNNNNNNNNNNGNNNNNNNGGNTGTGGANGGNNNANNGTGANNCCTCTGTGNTGAAGTACCNNNANNNANTTCGTCTCTCGTTGTGCTGATTAATTCNTATTTNCANCTGAAACATTCTCTCCCTTATCCCCTCCATAAATCCTAGNTGATAANTTTTATTATAATAAAATTTTTTTTATNTNTCTGNGNTANGGNCAGAGTCTCCTCCTGNGNCNCTGAATGTGGAGNGNTGGNNCAAACATATATAACTGNNNNNTGNANNTCTTGNGCTNNTGNACTCTCCTGNCTTNNANNNNAAAAAGTTGTNTTTATTAANNTGAACCTTTGCNATTANCCCANATCCGAGGGCACNANAANANAAAAAACTCCTACANTTTTTGNGATTAGTCNTCNGGNGGAAATAATTATAANCATAANCTATGATANGCACNNNCAANTANTNTTGCTGCGAACTACTTTTNNACCANANGCTCTAGGGANNAATAANGAGACGACNNCCCCCTCCCTCACTTTAGTTGACTAAGTACCNNNAANNNCATNCTAANACTTGTNNNAGNGTGTATGAATAACAANNCC

Reverse CO9567: ACGCCCTTTTAAATATCCG

NNNNNCTNNTCTCTTAGGTTTACCCGCCAATATATCCTGTCAAACACTGATAGTTTAAACCACTTCGTGGTGCAGTACCTCTCACGACTTGGTCTCACCTGACACAGGTCCCAAGCCCTTACCTCCACGGTGTCTACCCTCCCTCCAGGACTTCCTCCGTGTGCCAGCTCCAGCAAAGGATCTGATTCAGCTCGCCCCCAAAAAAGACTTTTAATAGTTCAATAACAATAATGAATATGCAAGGTTTGTTCTAAGGCATTTAGAAATGGTTTCAGGGAGTCATGAAGCCAGTCCTCTCTTGGGCTAGGGGAGGCCGAGATGGTCTTGAGCTCCAGGGGACTTGTTTCTTAGTGCCCAGGCCTGGGCGCCCCTCCCCCACCAAGCCTCCCAGGTCTTCTGTCCAAAGCCCTCCCCCTCCACCCCACCTCCAACCCCGTCTGCTCTACCCCATCAACTACGTTTTCTCCCTCAGCACTTGCCTTATACCCCATGCACTCACGAGCACAGAGGCGACTTCCCTCTCTCAGACTTTAGGCGCCACTGCAGGGTCCGGAAAAGAAAGAGAAACGGCCCAGCGCGGTCGCTTACATAACCCAGGGCGGGGCTCCGCTCCGCCCCCGAAAGTTTTTGCGACGGAGTTTTCCCTCTTGTTGCCCAGGCTGAAGTGCAATGGCGCGATCTCGGCTCACCGCAACCTCCGCCTCCAAGGTTTAAGCGATTCTCCTGCCTCAGCCTCCCGACTAGCTGGGATTACAGGCATTCACCACCAAGCCCGTCTAATTTTATATTTTTAGTAGAGACGGGATTTCCCCATGTTGGTCAGGTTGGTCTCGAGCTCCCGACCTCAGGTGATCGCCCGCCTCGACCTCCCAAAGTGCTGGGATTACAGGCGTGAACCACCGTGCCCGGCCTTGGCCGCATGTGTTTTGAATTTTGCTGCCCGGAATTCACTGCGAGGACTGGGATCACCCGTCACCCCGCCCTGGTCTACGGAAAATGAAATGTGTTTACTGATATAGAAACGGAATAACGGCGCTGTGGGCTGGGGAGGGCCGAGCTGCCTTCAGGCTTCTGGTCTCCAGCTGCAGGGCACTCACACCTGCCCGTTATGAAAATGCAGACCCGCAGGGCAGGAATTCCGAGTCGGGGCTGGAGCGCGATCTGGAATCCGGCTCTCTTGAAACAGCACCGCGGGAGGATTCGGATCCGGGTGANAAGGAAACTGCGCCTNNCCCCTCCCACGGGCCGCCCCNNNNTTCAAGAATCCAAAACGNNTTCCTGNTGGTCCGNNNNNCCNNNAAANGCAANNNCCNNNTNNTGGNNNNNT

Reverse Complement Reverse CO9567: ACGCCCTTTTAAATATCCG

TCACCCGGATCCGAATCCTCCCGCGGTGCTGTTTCAAGAGAGCCGGATTCCAGATCGCGCTCCAGCCCCGACTCGGAATTCCTGCCCTGCGGGTCTGCATTTTCATAACGGGCAGGTGTGAGTGCCCTGCAGCTGGAGACCAGAAGCCTGAAGGCAGCTCGGCCCTCCCCAGCCCACAGCGCCGTTATTCCGTTTCTATATCAGTAAACACATTTCATTTTCCGTAGACCAGGGCGGGGTGACGGGTGATCCCAGTCCTCGCAGTGAATTCCGGGCAGCAAAATTCAAAACACATGCGGCCAAGGCCGGGCACGGTGGTTCACGCCTGTAATCCCAGCACTTTGGGAGGTCGAGGCGGGCGATCACCTGAGGTCGGGAGCTCGAGACCAACCTGACCAACATGGGGAAATCCCGTCTCTACTAAAAATATAAAATTAGACGGGCTTGGTGGTGAATGCCTGTAATCCCAGCTAGTCGGGAGGCTGAGGCAGGAGAATCGCTTAAACCTTGGAGGCGGAGGTTGCGGTGAGCCGAGATCGCGCCATTGCACTTCAGCCTGGGCAACAAGAGGGAAAACTCCGTCGCAAAAACTTTCGGGGGCGGAGCGGAGCCCCGCCCTGGGTTATGTAAGCGACCGCGCTGGGCCGTTTCTCTTTCTTTTCCGGACCCTGCAGTGGCGCCTAAAGTCTGAGAGAGGGAAGTCGCCTCTGTGCTCGTGAGTGCATGGGGTATAAGGCAAGTGCTGAGGGAGAAAACGTAGTTGATGGGGTAGAGCAGACGGGGTTGGAGGTGGGGTGGAGGGGGAGGGCTTTGGACAGAAGACCTGGGAGGCTTGGTGGGGGAGGGGCGCCCAGGCCTGGGCACTAAGAAACAAGTCCCCTGGAGCTCAAGACCATCTCGGCCTCCCCTAGCCCAAGAGAGGACTGGCTTCATGACTCCCTGAAACCATTTCTAAATGCCTTAGAACAAACCTTGCATATTCATTATTGTTATTGAACTATTAAAAGTCTTTTTTGGGGGCGAGCTGAATCAGATCCTTTGCTGGAGCTGGCACACGGAGGAAGTCCTGGAGGGAGGGTAGACACCGTGGAGGTAAGGGCTTGGGACCTGTGTCAGGTGAGACCAAGTCGTGAGAGGTACTGCACCACGAAGTGGTTTAAACTATCAGTGTTTGACAGGATATATTGGCGGGTAAACCTAAGAGA

**Assembled sequence**

Insert 1

GGACGTTTTTATGNACTGGGGTGGATGCAGTGGGCCCCACTCTGTGGTGCAGTACCTCTCACGACTTGGTCTCACGTTGTGGGGCTTAGCTTCATATTTTCAAACTGAAATATTCTCTTCCTTAACCTCCACATAAATCCAAGTTTATAATTTTTATTATTTTAAAATTTTATTTATTTTTCTGTTTTGGGGACAGGGTCTCCTCCTGTCACTCAGGCTGGAGTGCAATGGCACAATCATAGCTCACTGCAGCCTGGAACTCCTGGGCTTAAGCAATCTTCCTGCCTTCGATTCCCAAAGAGCTGGGATTATAGTCATGAACCACTGCAATCCACCCAAATCCAAGTTTACACTAAAAGATAAAATTCCAACATTGTAGGGGATTGGTCAGGTGGTGGGAATAATTATAAAGATAAAGTTATAGGAAATAGACACAAACCTTCTTGGAAGGTGGAAAGTTTTGCAAAAGCCTCAGGATAGGGTTATAGCTGAAAGCAGCCTAATCCCCTTACCTTGAGTTAATAGCTTCGAGTAAGTACAAAGACATGTAAGAGAGTTTATCTAAAGAGCATGTTTACCTTTGATCATTTGTAGGACTGCTCTCTCTGGGGGACTGCGACCAGATTAATTACCCACAGGTGTGTTGACTCAAAGCCTTTGTCATTAAATCTGTGCTGAATAAAGGCCCACAGGGCCAGATAGTCAGGGCACGCAGCTGCCACAACCCTTTCTGTGAGTGGCCTGGCCCTCTGGTGCACTCTTTCACTGAATATCGGTGTCTGAGTACATTATTCATCCATCGTGCAGCCTGGGTCTGCCGGTCAGACCCTGGCACAACATTTAAGAGGAAATGAAAGTCACAAAGTTATCCCAGTCTCTGGAGTCACTGTCAAAACTTTGGTGAGGAATCTTCCAGNTTTTCCCCTACTTCACATATATATTAATATTATGTAAGTGATATTA

Insert 2

Bad quality sequence

Inserts 3 and 4

TCACCCGGATCCGAATCCTCCCGCGGTGCTGTTTCAAGAGAGCCGGATTCCAGATCGCGCTCCAGCCCCGACTCGGAATTCCTGCCCTGCGGGTCTGCATTTTCATAACGGGCAGGTGTGAGTGCCCTGCAGCTGGAGACCAGAAGCCTGAAGGCAGCTCGGCCCTCCCCAGCCCACAGCGCCGTTATTCCGTTTCTATATCAGTAAACACATTTCATTTTCCGTAGACCAGGGCGGGGTGACGGGTGATCCCAGTCCTCGCAGTGAATTCCGGGCAGCAAAATTCAAAACACATGCGGCCAAGGCCGGGCACGGTGGTTCACGCCTGTAATCCCAGCACTTTGGGAGGTCGAGGCGGGCGATCACCTGAGGTCGGGAGCTCGAGACCAACCTGACCAACATGGGGAAATCCCGTCTCTACTAAAAATATAAAATTAGACGGGCTTGGTGGTGAATGCCTGTAATCCCAGCTAGTCGGGAGGCTGAGGCAGGAGAATCGCTTAAACCTTGGAGGCGGAGGTTGCGGTGAGCCGAGATCGCGCCATTGCACTTCAGCCTGGGCAACAAGAGGGAAAACTCCGTCGCAAAAACTTTCGGGGGCGGAGCGGAGCCCCGCCCTGGGTTATGTAAGCGACCGCGCTGGGCCGTTTCTCTTTCTTTTCCGGACCCTGCAGTGGCGCCTAAAGTCTGAGAGAGGGAAGTCGCCTCTGTGCTCGTGAGTGCATGGGGTATAAGGCAAGTGCTGAGGGAGAAAACGTAGTTGATGGGGTAGAGCAGACGGGGTTGGAGGTGGGGTGGAGGGGGAGGGCTTTGGACAGAAGACCTGGGAGGCTTGGTGGGGGAGGGGCGCCCAGGCCTGGGCACTAAGAAACAAGTCCCCTGGAGCTCAAGACCATCTCGGCCTCCCCTAGCCCAAGAGAGGACTGGCTTCATGACTCCCTGAAACCATTTCTAAATGCCTTAGAACAAACCTTGCATATTCATTATTGTTATTGAACTATTAAAAGTCTTTTTTGGGGGCGAGCTGAATCAGATCCTTTGCTGGAGCTGGCACACGGAGGAAGTCCTGGAGGGAGGGTAGACACCGTGGAGGTAAGGGCTTGGGACCTGTGTCAGGTGAGACCAAGTCGTGAGAGGTACTGCACCACGAAGTGGTTTAAACTATCAGTGTTTGACAGGATATATTGGCGGGTAAACCTAAGAGA

## Colony 5

Forward CO9566: TGGTGTAAACAAATTGACGC

NNNNNNNNNTTGNGGACGTTTTTATGNACTGGGGTGGATGCAGTGGGCCCCACTCTGTGGTGCAGTACCTCTCACGACTTGGTCTCACGTTGTGGGGCTTAGCTTCATATTTTCAAACTGAAATATTCTCTTCCTTAACCTCCACATAAATCCAAGTTTATAATTTTTATTATTTTAAAATTTTATTTATTTTTCTGTTTTGGGGACAGGGTCTCCTCCTGTCACTCAGGCTGGAGTGCAATGGCACAATCATAGCTCACTGCAGCCTGGAACTCCTGGGCTTAAGCAATCTTCCTGCCTTCGATTCCCAAAGAGCTGGGATTATAGTCATGAACCACTGCAATCCACCCAAATCCAAGTTTACACTAAAAGATAAAATTCCAACATTGTAGGGGATTGGTCAGGTGGTGGGAATAATTATAAAGATAAAGTTATAGGAAATAGACACAAACCTTCTTGGAAGGTGGAAAGTTTTGCAAAAGCCTCAGGATAGGGTTATAGCTGAAAGCAGCCTAATCCCCTTACCTTGAGTTAATAGCTTCGAGTAAGTACAAAGACATGTAAGAGAGTTTATCTAAAGAGCATGTTTACCTTTGATCATTTGTAGGACTGCTCTCTCTGGGGGACTGCGACCAGATTAATTACCCACAGGTGTGTTGACTCAAAGCCTTTGTCATTAAATCTGTGCTGAATAAAGGCCCACAGGGCCAGATAGTCAGGGCACGCAGCTGCCACAACCCTTTCTGTGAGTGGCCTGGCCCTCTGGTGCACTCTTTCACTGAATATCGGTGTCTGAGTACATTATTCATCCATCGTGCAGCCTGGGTCTGCCGGTCAGACCCTGGCACAACATTTAAGAGGAAATGAAAGTCACAAAGTTATCCCAGTCTCTGGAGTCACTGTCAAAACTTTGGTGAGGAATCTTCCAGGTTTTCCCCTACTTCAAATATATATTAATATTATGTAAGTGATATTAGTGGCATTTTCGCCC

Forward CO9574: AGAAATAATGAAACTACGTC

NNNNNNCNNNNNGGNGCGGNGGCTCACGCCTGTAATCCCAGCACTTTGAGAGGCCAAGGCAGGCAGATCACGAGGTCAGGAGATCGAGACCATCCTGGCTAACACAGTGAAACCCCGTCTCTACTAAAAATACAAAAAATTAGTCGGGCGTGGTGGCAGGAGCCTGTAGTCCCAGCTACTCCAGAGGCTGAGGCAGCAGAATGCCCTGAACCCGGGAGGCGGAGCTTGCAGTGAGCCAAGATCGCGCCACTGCACTCCAGCCTGGGCGACAGAGCAAGACTCCGTCTCAAAAAAAAAAAACAAAAAACAAAAAAACTCTCCTTTACTTTTTCTCTCCCCTTTTCTTCCTATCTCTTCCCTCATTTCTTCAACACGTCCCCCCATCCTTCCCTCTTTTCTCCATTCTCTGCATTTGATCCCCGGTATATTCCAGCCTCCAGGCCAACAAACTTCTCCGCGTCCGCCGGGAGCAGGTCAGGGAAGGGACGCGAGGCGGCGCTGTCACCGCATTCTGAGCGCCGCAGCTCCCTGGGCCCCTTGTATCATTTCAGTGAAGGTCACTCCAGTCTTTCATGGAGGCCAAACTAAGGGTGTAAATTAGGATCCTCACTGAAGTGGCGGGACCCTAAGAGGCTTTTTCCTGGCCCCTTAGTTGTGGGTTTTCCTGCGGGCGGCGCAGCCGGTTTCCATCAGAACCGCCCAGAGGCGGACGCTGCCTTCCTGGGGTGACGGAGCAGCAGGAAGCGTTTTCGGATCCTGGAATACGTGGGCGGCCCGTGGNAGGNTCTGNAGTGCAGTTTCCTACTCACCCGGATCCGAATCCTCCNNGNNGCTGTTTCAAGAGAGNGG

Reverse CO9567: ACGCCCTTTTAAATATCCG

NNNNNNNNTTCTCTTAGGTTTACCCGCCAATATATCCTGTCAAACACTGATAGTTTAAACCACTTCGTGGTGCAGTACCTCTCACGACTTGGTCTCACCTGACACAGGTCCCAAGCCCTTACCTCCACGGTGTCTACCCTCCCTCCAGGACTTCCTCCGTGTGCCAGCTCCAGCAAAGGATCTGATTCAGCTCGCCCCCAAAAAAGACTTTTAATAGTTCAATAACAATAATGAATATGCAAGGTTTGTTCTAAGGCATTTAGAAATGGTTTCAGGGAGTCATGAAGCCAGTCCTCTCTTGGGCTAGGGGAGGCCGAGATGGTCTTGAGCTCCAGGGGACTTGTTTCTTAGTGCCCAGGCCTGGGCGCCCCTCCCCCACCAAGCCTCCCAGGTCTTCTGTCCAAAGCCCTCCCCCTCCACCCCACCTCCAACCCCGTCTGCTCTACCCCATCAACTACGTTTTCTCCCTCAGCACTTGCCTTATACCCCATGCACTCACGAGCACAGAGGCGACTTCCCTCTCTCAGACTTTAGGCGCCACTGCAGGGTCCGGAAAAGAAAGAGAAACGGCCCAGCGCGGTCGCTTACATAACCCAGGGCGGGGCTCCGCTCCGCCCCCGAAAGTTTTTGCGACGGAGTTTTCCCTCTTGTTGCCCAGGCTGAAGTGCAATGGCGCGATCTCGGCTCACCGCAACCTCCGCCTCCAAGGTTTAAGCGATTCTCCTGCCTCAGCCTCCCGACTAGCTGGGATTACAGGCATTCACCACCAAGCCCGTCTAATTTTATATTTTTAGTAGAGACGGGATTTCCCCATGTTGGTCAGGTTGGTCTCGAGCTCCCGACCTCAGGTGATCGCCCGCCTCGACCTCCCAAAGTGCTGGGATTACAGGCGTGAACCACCGTGCCCGGCCTTGGCCGCATGTGTTTTGAATTTTGCTGCCCGGAATTCACTGCGAGGACTGGGATCACCCGTCACCCCGCCCTGGTCTACGGAAAATGAAATGTGTTTACTGATATAGAAACGGAATAACGGCGCTGTGGGCTGGGGAGGGCCGAGCTGCCTTCAGGCTTCTGGTCTCCAGCTGCAGGGCACTCACACCTGCCCGTTATGAAAATGCAGACCCGCAGGGCAGGAATTCCGAGTCCGGGCTGGAGCGCGATCTGGAATCCGGCTCTCTTGAAACAGNACCGCGGAGGATTCGGATCCGGNNGAGTANNAAACTGNNCCTCAGCCCCTCCCACGGGCCGCCCACGNNTTCCAGGATCCGAAAACGCTTCCTGCTGCTCCGTCACCCCAGGAAGGCAACGTCCGCCTCTGGCCGGTTCTGATGGAAACCGGCTGNNCCNCCNGCAGAAAACCCCNNA

Reverse Complement Reverse CO9567: ACGCCCTTTTAAATATCCG

CTGTTTCAAGAGAGCCGGATTCCAGATCGCGCTCCAGCCCGGACTCGGAATTCCTGCCCTGCGGGTCTGCATTTTCATAACGGGCAGGTGTGAGTGCCCTGCAGCTGGAGACCAGAAGCCTGAAGGCAGCTCGGCCCTCCCCAGCCCACAGCGCCGTTATTCCGTTTCTATATCAGTAAACACATTTCATTTTCCGTAGACCAGGGCGGGGTGACGGGTGATCCCAGTCCTCGCAGTGAATTCCGGGCAGCAAAATTCAAAACACATGCGGCCAAGGCCGGGCACGGTGGTTCACGCCTGTAATCCCAGCACTTTGGGAGGTCGAGGCGGGCGATCACCTGAGGTCGGGAGCTCGAGACCAACCTGACCAACATGGGGAAATCCCGTCTCTACTAAAAATATAAAATTAGACGGGCTTGGTGGTGAATGCCTGTAATCCCAGCTAGTCGGGAGGCTGAGGCAGGAGAATCGCTTAAACCTTGGAGGCGGAGGTTGCGGTGAGCCGAGATCGCGCCATTGCACTTCAGCCTGGGCAACAAGAGGGAAAACTCCGTCGCAAAAACTTTCGGGGGCGGAGCGGAGCCCCGCCCTGGGTTATGTAAGCGACCGCGCTGGGCCGTTTCTCTTTCTTTTCCGGACCCTGCAGTGGCGCCTAAAGTCTGAGAGAGGGAAGTCGCCTCTGTGCTCGTGAGTGCATGGGGTATAAGGCAAGTGCTGAGGGAGAAAACGTAGTTGATGGGGTAGAGCAGACGGGGTTGGAGGTGGGGTGGAGGGGGAGGGCTTTGGACAGAAGACCTGGGAGGCTTGGTGGGGGAGGGGCGCCCAGGCCTGGGCACTAAGAAACAAGTCCCCTGGAGCTCAAGACCATCTCGGCCTCCCCTAGCCCAAGAGAGGACTGGCTTCATGACTCCCTGAAACCATTTCTAAATGCCTTAGAACAAACCTTGCATATTCATTATTGTTATTGAACTATTAAAAGTCTTTTTTGGGGGCGAGCTGAATCAGATCCTTTGCTGGAGCTGGCACACGGAGGAAGTCCTGGAGGGAGGGTAGACACCGTGGAGGTAAGGGCTTGGGACCTGTGTCAGGTGAGACCAAGTCGTGAGAGGTACTGCACCACGAAGTGGTTTAAACTATCAGTGTTTGACAGGATATATTGGCGGGTAAACCTAAGAGAA

**Assembled sequence**

Insert 1

ACTGGGGTGGATGCAGTGGGCCCCACTCTGTGGTGCAGTACCTCTCACGACTTGGTCTCACGTTGTGGGGCTTAGCTTCATATTTTCAAACTGAAATATTCTCTTCCTTAACCTCCACATAAATCCAAGTTTATAATTTTTATTATTTTAAAATTTTATTTATTTTTCTGTTTTGGGGACAGGGTCTCCTCCTGTCACTCAGGCTGGAGTGCAATGGCACAATCATAGCTCACTGCAGCCTGGAACTCCTGGGCTTAAGCAATCTTCCTGCCTTCGATTCCCAAAGAGCTGGGATTATAGTCATGAACCACTGCAATCCACCCAAATCCAAGTTTACACTAAAAGATAAAATTCCAACATTGTAGGGGATTGGTCAGGTGGTGGGAATAATTATAAAGATAAAGTTATAGGAAATAGACACAAACCTTCTTGGAAGGTGGAAAGTTTTGCAAAAGCCTCAGGATAGGGTTATAGCTGAAAGCAGCCTAATCCCCTTACCTTGAGTTAATAGCTTCGAGTAAGTACAAAGACATGTAAGAGAGTTTATCTAAAGAGCATGTTTACCTTTGATCATTTGTAGGACTGCTCTCTCTGGGGGACTGCGACCAGATTAATTACCCACAGGTGTGTTGACTCAAAGCCTTTGTCATTAAATCTGTGCTGAATAAAGGCCCACAGGGCCAGATAGTCAGGGCACGCAGCTGCCACAACCCTTTCTGTGAGTGGCCTGGCCCTCTGGTGCACTCTTTCACTGAATATCGGTGTCTGAGTACATTATTCATCCATCGTGCAGCCTGGGTCTGCCGGTCAGACCCTGGCACAACATTTAAGAGGAAATGAAAGTCACAAAGTTATCCCAGTCTCTGGAGTCACTGTCAAAACTTTGGTGAGGAATCTTCCAGGTTTTCCCCTACTTCAAATATATATTAATATTATGTAAGTGATATTAGTGGCATTTTCGCCC

Inserts 2 and 3

GGCTCACGCCTGTAATCCCAGCACTTTGAGAGGCCAAGGCAGGCAGATCACGAGGTCAGGAGATCGAGACCATCCTGGCTAACACAGTGAAACCCCGTCTCTACTAAAAATACAAAAAATTAGTCGGGCGTGGTGGCAGGAGCCTGTAGTCCCAGCTACTCCAGAGGCTGAGGCAGCAGAATGCCCTGAACCCGGGAGGCGGAGCTTGCAGTGAGCCAAGATCGCGCCACTGCACTCCAGCCTGGGCGACAGAGCAAGACTCCGTCTCAAAAAAAAAAAACAAAAAACAAAAAAACTCTCCTTTACTTTTTCTCTCCCCTTTTCTTCCTATCTCTTCCCTCATTTCTTCAACACGTCCCCCCATCCTTCCCTCTTTTCTCCATTCTCTGCATTTGATCCCCGGTATATTCCAGCCTCCAGGCCAACAAACTTCTCCGCGTCCGCCGGGAGCAGGTCAGGGAAGGGACGCGAGGCGGCGCTGTCACCGCATTCTGAGCGCCGCAGCTCCCTGGGCCCCTTGTATCATTTCAGTGAAGGTCACTCCAGTCTTTCATGGAGGCCAAACTAAGGGTGTAAATTAGGATCCTCACTGAAGTGGCGGGACCCTAAGAGGCTTTTTCCTGGCCCCTTAGTTGTGGGTTTTCCTGCGGGCGGCGCAGCCGGTTTCCATCAGAACCGCCCAGAGGCGGACGCTGCCTTCCTGGGGTGACGGAGCAGCAGGAAGCGTTTTCGGATCCTGGAATACGTGGGCGGCCCGTGG

Inserts 3 and 4

CTGTTTCAAGAGAGCCGGATTCCAGATCGCGCTCCAGCCCGGACTCGGAATTCCTGCCCTGCGGGTCTGCATTTTCATAACGGGCAGGTGTGAGTGCCCTGCAGCTGGAGACCAGAAGCCTGAAGGCAGCTCGGCCCTCCCCAGCCCACAGCGCCGTTATTCCGTTTCTATATCAGTAAACACATTTCATTTTCCGTAGACCAGGGCGGGGTGACGGGTGATCCCAGTCCTCGCAGTGAATTCCGGGCAGCAAAATTCAAAACACATGCGGCCAAGGCCGGGCACGGTGGTTCACGCCTGTAATCCCAGCACTTTGGGAGGTCGAGGCGGGCGATCACCTGAGGTCGGGAGCTCGAGACCAACCTGACCAACATGGGGAAATCCCGTCTCTACTAAAAATATAAAATTAGACGGGCTTGGTGGTGAATGCCTGTAATCCCAGCTAGTCGGGAGGCTGAGGCAGGAGAATCGCTTAAACCTTGGAGGCGGAGGTTGCGGTGAGCCGAGATCGCGCCATTGCACTTCAGCCTGGGCAACAAGAGGGAAAACTCCGTCGCAAAAACTTTCGGGGGCGGAGCGGAGCCCCGCCCTGGGTTATGTAAGCGACCGCGCTGGGCCGTTTCTCTTTCTTTTCCGGACCCTGCAGTGGCGCCTAAAGTCTGAGAGAGGGAAGTCGCCTCTGTGCTCGTGAGTGCATGGGGTATAAGGCAAGTGCTGAGGGAGAAAACGTAGTTGATGGGGTAGAGCAGACGGGGTTGGAGGTGGGGTGGAGGGGGAGGGCTTTGGACAGAAGACCTGGGAGGCTTGGTGGGGGAGGGGCGCCCAGGCCTGGGCACTAAGAAACAAGTCCCCTGGAGCTCAAGACCATCTCGGCCTCCCCTAGCCCAAGAGAGGACTGGCTTCATGACTCCCTGAAACCATTTCTAAATGCCTTAGAACAAACCTTGCATATTCATTATTGTTATTGAACTATTAAAAGTCTTTTTTGGGGGCGAGCTGAATCAGATCCTTTGCTGGAGCTGGCACACGGAGGAAGTCCTGGAGGGAGGGTAGACACCGTGGAGGTAAGGGCTTGGGACCTGTGTCAGGTGAGACCAAGTCGTGAGAGGTACTGCACCACGAAGTGGTTTAAACTATCAGTGTTTGACAGGATATATTGGCGGGTAAACCTAAGAGAA

# POC1431 sequences from five white colonies using the methylases M.Osp807II and M2.BsaI

## Colony 1

Forward CO9566: TGGTGTAAACAAATTGACGC

NNNNNNNNNNNNNGACGTTTTTATGNACTGGGGTGGATGCAGTGGGCCCCACTCTGTGGTGCAGTACCTCTCACGACTTGGTCTCACGTTGTGGGGCTTAGCTTCATATTTTCAAACTGAAATATTCTCTTCCTTAACCTCCACATAAATCCAAGTTTATAATTTTTATTATTTTAAAATTTTATTTATTTTTCTGTTTTGGGGACAGGGTCTCCTCCTGTCACTCAGGCTGGAGTGCAATGGCACAATCATAGCTCACTGCAGCCTGGAACTCCTGGGCTTAAGCAATCTTCCTGCCTTCGATTCCCAAAGAGCTGGGATTATAGTCATGAACCACTGCAATCCACCCAAATCCAAGTTTACACTAAAAGATAAAATTCCAACATTGTAGGGGATTGGTCAGGTGGTGGGAATAATTATAAAGATAAAGTTATAGGAAATAGACACAAACCTTCTTGGAAGGTGGAAAGTTTTGCAAAAGCCTCAGGATAGGGTTATAGCTGAAAGCAGCCTAATCCCCTTACCTTGAGTTAATAGCTTCGAGTAAGTACAAAGACATGTAAGAGAGTTTATCTAAAGAGCATGTTTACCTTTGATCATTTGTAGGACTGCTCTCTCTGGGGGACTGCGACCAGATTAATTACCCACAGGTGTGTTGACTCAAAGCCTTTGTCATTAAATCTGTGCTGAATAAAGGCCCACAGGGCCAGATAGTCAGGGCACGCAGCTGCCACAACCCTTTCTGTGAGTGACCTGCNCCTCTGGTGCACTCTTTCACTGAATATCGGTGTCTGAGTACATTATTCATCCATCGTGCAGCCTGTGTCTGCCGGTCAGACCCTGGCACAACATTTAAGAGGAANTGANGGTCNNAAAGTTATCCCAGTCTCTGTAGTCACTGTCAAAACTTTGGTGANGAATCTTCCAGGTTTTCCCCTACTTTCNNNANNNNTTAATATNNNGGNNN

Forward CO9574: AGAAATAATGAAACTACGTC

NNNNNNNNNNNNNNNGNGCGGTGGCTCACGCCTGTAATCCCAGCACTTTGAGAGGCCAAGGCAGGCAGATCACGAGGTCAGGAGATCGAGACCATCCTGGCTAACACAGTGAAACCCCGTCTCTACTAAAAATACAAAAAATTAGTCGGGCGTGGTGGCAGGAGCCTGTAGTCCCAGCTACTCCAGAGGCTGAGGCAGCAGAATGCCCTGAACCCGGGAGGCGGAGCTTGCAGTGAGCCAAGATCGCGCCACTGCACTCCAGCCTGGGCGACAGAGCAAGACTCCGTCTCAAAAAAAAAAAACAAAAAACAAAAAAACTCTCCTTTACTTTTTCTCTCCCCTTTTCTTCCTATCTCTTCCCTCATTTCTTCAACACGTCCCCCCATCCTTCCCTCTTTTCTCCATTCTCTGCATTTGATCCCCGGTATATTCCAGCCTCCAGGCCAACAAACTTCTCCGCGTCCGCCGGGAGCAGGTCAGGGAAGGGACGCGAGGCGGCGCTGTCACCGCATTCTGAGCGCCGCAGCTCCCTGGGCCCCTTGTATCATTTCAGTGAAGGTCACTCCAGTCTTTCATGGAGGCCAAACTAAGGNNGTAAATTAGGATCCTCACTGAAGTGGCGGGACCCTATGAGGCTTTTTCCTGGCCCCTTAGATGNNNCATTTCCTGNGGTGNNANNNTCCGTTTTCCATNNAATCCATCCGAGTNGANCNCNNNCTCCNTGNGTGANNTAGTANTATCATCCNNTNNCCCTNNNTNNTATACCTGNNNNNCCNNNNNNCCCGNNNCNT

Reverse CO9567: ACGCCCTTTTAAATATCCG

NNNNNNNNNNNNCTTAGGTTTACCCGCCAATATATCCTGTCAAACACTGATAGTTTAAACCACTTCGTGGTGCAGTACCTCTCACGACTTGGTCTCACCTGACACAGGTCCCAAGCCCTTACCTCCACGGTGTCTACCCTCCCTCCAGGACTTCCTCCGTGTGCCAGCTCCAGCAAAGGATCTGATTCAGCTCGCCCCCAAAAAAGACTTTTAATAGTTCAATAACAATAATGAATATGCAAGGTTTGTTCTAAGGCATTTAGAAATGGTTTCAGGGAGTCATGAAGCCAGTCCTCTCTTGGGCTAGGGGAGGCCGAGATGGTCTTGAGCTCCAGGGGACTTGTTTCTTAGTGCCCAGGCCTGGGCGCCCCTCCCCCACCAAGCCTCCCAGGTCTTCTGTCCAAAGCCCTCCCCCTCCACCCCACCTCCAACCCCGTCTGCTCTACCCCATCAACTACGTTTTCTCCCTCAGCACTTGCCTTATACCCCATGCACTCACGAGCACAGAGGCGACTTCCCTCTCTCAGACTTTAGGCGCCACTGCAGGGTCCGGAAAAGAAAGAGAAACGGCCCAGCGCGGTCGCTTACATAACCCAGGGCGGGGCTCCGCTCCGCCCCCGAAAGTTTTTGCGACGGAGTTTTCCCTCTTGTTGCCCAGGCTGAAGTGCAATGGCGCGATCTCGGCTCACCGCAACCTCCGCCTCCAAGGTTTAAGCGATTCTCCTGCCTCAGCCTCCCGACTAGCTGGGATTACAGGCATTCACCACCAAGCCCGTCTAATTTTATATTTTTAGNAGAGACGGGATTTCCCCATGTTGGTCAGGTTGGTCTCGAGCTCCCGACCTCAGGTGATCGCCCGCCTCGACCTCCCAAAGTGCTGGGATTACAGGCGTGAACCACCTNGCCGGTCTTGGCCGCATGTGTTTTGANTATGCTGTCCCGAATTNACTGTGANNATTGGTATCTCTCGTNNCCCCGCCTGNTCTNCNGAAAACGAANGTNNNATGGAATAGNNNNGAATAACGNNNCTGTNNNNTGN

Reverse Complement Reverse CO9567: ACGCCCTTTTAAATATCCG

CTAAAAATATAAAATTAGACGGGCTTGGTGGTGAATGCCTGTAATCCCAGCTAGTCGGGAGGCTGAGGCAGGAGAATCGCTTAAACCTTGGAGGCGGAGGTTGCGGTGAGCCGAGATCGCGCCATTGCACTTCAGCCTGGGCAACAAGAGGGAAAACTCCGTCGCAAAAACTTTCGGGGGCGGAGCGGAGCCCCGCCCTGGGTTATGTAAGCGACCGCGCTGGGCCGTTTCTCTTTCTTTTCCGGACCCTGCAGTGGCGCCTAAAGTCTGAGAGAGGGAAGTCGCCTCTGTGCTCGTGAGTGCATGGGGTATAAGGCAAGTGCTGAGGGAGAAAACGTAGTTGATGGGGTAGAGCAGACGGGGTTGGAGGTGGGGTGGAGGGGGAGGGCTTTGGACAGAAGACCTGGGAGGCTTGGTGGGGGAGGGGCGCCCAGGCCTGGGCACTAAGAAACAAGTCCCCTGGAGCTCAAGACCATCTCGGCCTCCCCTAGCCCAAGAGAGGACTGGCTTCATGACTCCCTGAAACCATTTCTAAATGCCTTAGAACAAACCTTGCATATTCATTATTGTTATTGAACTATTAAAAGTCTTTTTTGGGGGCGAGCTGAATCAGATCCTTTGCTGGAGCTGGCACACGGAGGAAGTCCTGGAGGGAGGGTAGACACCGTGGAGGTAAGGGCTTGGGACCTGTGTCAGGTGAGACCAAGTCGTGAGAGGTACTGCACCACGAAGTGGTTTAAACTATCAGTGTTTGACAGGATATATTGGCGGGTAAACCTAAG

**Assembled sequence**

Insert 1

GACGTTTTTATGNACTGGGGTGGATGCAGTGGGCCCCACTCTGTGGTGCAGTACCTCTCACGACTTGGTCTCACGTTGTGGGGCTTAGCTTCATATTTTCAAACTGAAATATTCTCTTCCTTAACCTCCACATAAATCCAAGTTTATAATTTTTATTATTTTAAAATTTTATTTATTTTTCTGTTTTGGGGACAGGGTCTCCTCCTGTCACTCAGGCTGGAGTGCAATGGCACAATCATAGCTCACTGCAGCCTGGAACTCCTGGGCTTAAGCAATCTTCCTGCCTTCGATTCCCAAAGAGCTGGGATTATAGTCATGAACCACTGCAATCCACCCAAATCCAAGTTTACACTAAAAGATAAAATTCCAACATTGTAGGGGATTGGTCAGGTGGTGGGAATAATTATAAAGATAAAGTTATAGGAAATAGACACAAACCTTCTTGGAAGGTGGAAAGTTTTGCAAAAGCCTCAGGATAGGGTTATAGCTGAAAGCAGCCTAATCCCCTTACCTTGAGTTAATAGCTTCGAGTAAGTACAAAGACATGTAAGAGAGTTTATCTAAAGAGCATGTTTACCTTTGATCATTTGTAGGACTGCTCTCTCTGGGGGACTGCGACCAGATTAATTACCCACAGGTGTGTTGACTCAAAGCCTTTGTCATTAAATCTGTGCTGAATAAAGGCCCACAGGGCCAGATAGTCAGGGCACGCAGCTGCCACAACCCTTTCTGTGAGTGACCTGCNCCTCTGGTGCACTCTTTCACTGAATATCGGTGTCTGAGTACATTATTCATCCATCGTGCAGCCTGTGTCTGCCGGTCAGACCCTGGCACAACATTTAAGAGGAA

Inserts 2 and 3

GCGGTGGCTCACGCCTGTAATCCCAGCACTTTGAGAGGCCAAGGCAGGCAGATCACGAGGTCAGGAGATCGAGACCATCCTGGCTAACACAGTGAAACCCCGTCTCTACTAAAAATACAAAAAATTAGTCGGGCGTGGTGGCAGGAGCCTGTAGTCCCAGCTACTCCAGAGGCTGAGGCAGCAGAATGCCCTGAACCCGGGAGGCGGAGCTTGCAGTGAGCCAAGATCGCGCCACTGCACTCCAGCCTGGGCGACAGAGCAAGACTCCGTCTCAAAAAAAAAAAACAAAAAACAAAAAAACTCTCCTTTACTTTTTCTCTCCCCTTTTCTTCCTATCTCTTCCCTCATTTCTTCAACACGTCCCCCCATCCTTCCCTCTTTTCTCCATTCTCTGCATTTGATCCCCGGTATATTCCAGCCTCCAGGCCAACAAACTTCTCCGCGTCCGCCGGGAGCAGGTCAGGGAAGGGACGCGAGGCGGCGCTGTCACCGCATTCTGAGCGCCGCAGCTCCCTGGGCCCCTTGTATCATTTCAGTGAAGGTCACTCCAGTCTTTCATGGAGGCCAAACTAAGGNNGTAAATTAGGATCCTCACTGAAGTGGCGGGACCCTATGAGGCTTTTTCCTGGCCCCTTAGATG

Insert 4

CTAAAAATATAAAATTAGACGGGCTTGGTGGTGAATGCCTGTAATCCCAGCTAGTCGGGAGGCTGAGGCAGGAGAATCGCTTAAACCTTGGAGGCGGAGGTTGCGGTGAGCCGAGATCGCGCCATTGCACTTCAGCCTGGGCAACAAGAGGGAAAACTCCGTCGCAAAAACTTTCGGGGGCGGAGCGGAGCCCCGCCCTGGGTTATGTAAGCGACCGCGCTGGGCCGTTTCTCTTTCTTTTCCGGACCCTGCAGTGGCGCCTAAAGTCTGAGAGAGGGAAGTCGCCTCTGTGCTCGTGAGTGCATGGGGTATAAGGCAAGTGCTGAGGGAGAAAACGTAGTTGATGGGGTAGAGCAGACGGGGTTGGAGGTGGGGTGGAGGGGGAGGGCTTTGGACAGAAGACCTGGGAGGCTTGGTGGGGGAGGGGCGCCCAGGCCTGGGCACTAAGAAACAAGTCCCCTGGAGCTCAAGACCATCTCGGCCTCCCCTAGCCCAAGAGAGGACTGGCTTCATGACTCCCTGAAACCATTTCTAAATGCCTTAGAACAAACCTTGCATATTCATTATTGTTATTGAACTATTAAAAGTCTTTTTTGGGGGCGAGCTGAATCAGATCCTTTGCTGGAGCTGGCACACGGAGGAAGTCCTGGAGGGAGGGTAGACACCGTGGAGGTAAGGGCTTGGGACCTGTGTCAGGTGAGACCAAGTCGTGAGAGGTACTGCACCACGAAGTGGTTTAAACTATCAGTGTTTGACAGGATATATTGGCGGGTAAACCTAAG

## Colony 2

Forward CO9566: TGGTGTAAACAAATTGACGC

NNNNNNNNNNNNGGACGTTTTTATGNACTGGGGTGGATGCAGTGGGCCCCACTCTGTGGTGCAGTACCTCTCACGACTTGGTCTCACGTTGTGGGGCTTAGCTTCATATTTTCAAACTGAAATATTCTCTTCCTTAACCTCCACATAAATCCAAGTTTATAATTTTTATTATTTTAAAATTTTATTTATTTTTCTGTTTTGGGGACAGGGTCTCCTCCTGTCACTCAGGCTGGAGTGCAATGGCACAATCATAGCTCACTGCAGCCTGGAACTCCTGGGCTTAAGCAATCTTCCTGCCTTCGATTCCCAAAGAGCTGGGATTATAGTCATGAACCACTGCAATCCACCCAAATCCAAGTTTACACTAAAAGATAAAATTCCAACATTGTAGGGGATTGGTCAGGTGGTGGGAATAATTATAAAGATAAAGTTATAGGAAATAGACACAAACCTTCTTGGAAGGTGGAAAGTTTTGCAAAAGCCTCAGGATAGGGTTATAGCTGAAAGCAGCCTAATCCCCTTACCTTGAGTTAATAGCTTCGAGTAAGTACAAAGACATGTAAGAGAGTTTATCTAAAGAGCATGTTTACCTTTGATCATTTGTAGGACTGCTCTCTCTGGGGGACTGCGACCAGATTAATTACCCACAGGTGTGTTGACTCAAAGCCTTTGTCATTAAATCTGTGCTGAATAAAGGCCCACAGGGCCAGATAGTCAGGGCACGCAGCTGCCACAACCCTTTCTGTGAGTGGCCTGGCCCTCTGGTGCACTCTTTCACTGAATATCGGTGTCTGAGTACATTATTCATCCATCGTGCAGCCTGGGTCTGCCGGTCAGACCCTGGCACAACATTTAAGAGGAAATGAAAGTCACAAAGTTATCCCAGTCTCTGGAGTCACTGTCAAAACTTTGGTGAGGAATCTTCCAGGTTTTCCCCTACTTCAAATATATATTAATATTATGTAAGTGATATTAGTGGCATTT

Forward CO9574: AGAAATAATGAAACTACGTC

NNNNNNTCGGNNNNGNGCGGTGGCTCACGCCTGTAATCCCAGCACTTTGAGAGGCCAAGGCAGGCAGATCACGAGGTCAGGAGATCGAGACCATCCTGGCTAACACAGTGAAACCCCGTCTCTACTAAAAATACAAAAAATTAGTCGGGCGTGGTGGCAGGAGCCTGTAGTCCCAGCTACTCCAGAGGCTGAGGCAGCAGAATGCCCTGAACCCGGGAGGCGGAGCTTGCAGTGAGCCAAGATCGCGCCACTGCACTCCAGCCTGGGCGACAGAGCAAGACTCCGTCTCAAAAAAAAAAAACAAAAAACAAAAAAACTCTCCTTTACTTTTTCTCTCCCCTTTTCTTCCTATCTCTTCCCTCATTTCTTCAACACGTCCCCCCATCCTTCCCTCTTTTCTCCATTCTCTGCATTTGATCCCCGGTATATTCCAGCCTCCAGGCCAACAAACTTCTCCGCGTCCGCCGGGAGCAGGTCAGGGAAGGGACGCGAGGCGGCGCTGTCACCGCATTCTGAGCGCCGCAGCTCCCTGGGCCCCTTGTATCATTTCAGTGAAGGTCACTCCAGTCTTTCATGGAGGCCAAACTAAGGGTGTAAATTAGGATCCTCACTGAAGTGGCGGGACCCTAAGAGGCTTTTTCCTGGCCCCTTAGTTGTGGGTTTTCCTGCGGGCGGCGCAGCCGGTTTCCATCAGAACCGCCCAGAGGCGGACGCTGCCTTCCTGGGGTGACGGAGCAGCAGGAAGCGTTTTCGGATCCTGGAATACGTGGGCGGCCCGTGGGAGGGGCTGAGGCGCAGTTTCCTACTCACCCGGATCCGAATCCTCCGCGGTGCTGTTTCAAGAGAGCCGGATTCCAGATCGCGCTCCAGCCCGGACTCGGAATTCCTGCCCTGCGGGTCTGCATTTTCATAACGGGCAGGTGTGAGTGCCCTGCAGCTGGAGACCAGAAGCCTGAAGGCAGCTCGGCCCTCCCCAGCCCACAGCGCCGTTATTCCGTTTCTATATCAGTAAACACATTTCATTTTCCGTANACCAGGGCGGGGTGACGGGTGATCCCAGTTCTCGCNGTNANTNCGCGCANNAAANNTCNAANNNNCTGTGGNCAAGGNCGNTNACGGGGNTTNACGCCTGT

Reverse CO9567: ACGCCCTTTTAAATATCCG

NNNNNNNNNTTTTCTCTTAGGTTTACCCGCCAATATATCCTGTCAAACACTGATAGTTTAAACCACTTCGTGGTGCAGTACCTCTCACGACTTGGTCTCACCTGACACAGGTCCCAAGCCCTTACCTCCACGGTGTCTACCCTCCCTCCAGGACTTCCTCCGTGTGCCAGCTCCAGCAAAGGATCTGATTCAGCTCGCCCCCAAAAAAGACTTTTAATAGTTCAATAACAATAATGAATATGCAAGGTTTGTTCTAAGGCATTTAGAAATGGTTTCAGGGAGTCATGAAGCCAGTCCTCTCTTGGGCTAGGGGAGGCCGAGATGGTCTTGAGCTCCAGGGGACTTGTTTCTTAGTGCCCAGGCCTGGGCGCCCCTCCCCCACCAAGCCTCCCAGGTCTTCTGTCCAAAGCCCTCCCCCTCCACCCCACCTCCAACCCCGTCTGCTCTACCCCATCAACTACGTTTTCTCCCTCAGCACTTGCCTTATACCCCATGCACTCACGAGCACAGAGGCGACTTCCCTCTCTCAGACTTTAGGCGCCACTGCAGGGTCCGGAAAAGAAAGAGAAACGGCCCAGCGCGGTCGCTTACATAACCCAGGGCGGGGCTCCGCTCCGCCCCCGAAAGTTTTTGCGACGGAGTTTTCCCTCTTGTTGCCCAGGCTGAAGTGCAATGGCGCGATCTCGGCTCACCGCAACCTCCGCCTCCAAGGTTTAAGCGATTCTCCTGCCTCAGCCTCCCGACTAGCTGGGATTACAGGCATTCACCACCAAGCCCGTCTAATTTTATATTTTTAGTAGAGACGGGATTTCCCCATGTTGGTCAGGTTGGTCTCGAGCTCCCGACCTCAGGTGATCGCCCGCCTCGACCTCCCAAAGTGCTGGGATTACAGGCGTGAACCACCGTGCCCGGCCTTGGCCGCATGTGTTTTGAATTTTGCTGCCCGGAATTCACTGCGAGGACTGGGATCACCCGTCACCCCGCCCTGGTCTACGGAAAATGAAATGTGTTTACTGATATAGAAACGGAATAACGGCGCTGTGGGCTGGGGAGGGCCGAGCTGCCTTCAGGCTTCTGGTCTCCAGCTGCAGGGCACTCACACCTGCCCGTTATGAAAATGCAGACCCGCAGGGCAGGAAATCCGAGTCCGGGCTGAAGCGCGATCTGGAATCCGGCTNNNTTGAAACAGCACCNNGGAGGATTCGATCCGGTGNGTAAGAAATGCGCCNCAGCNCNNCCACGGNCGCCA

Reverse Complement Reverse CO9567: ACGCCCTTTTAAATATCCG

AGCCGGATTCCAGATCGCGCTTCAGCCCGGACTCGGATTTCCTGCCCTGCGGGTCTGCATTTTCATAACGGGCAGGTGTGAGTGCCCTGCAGCTGGAGACCAGAAGCCTGAAGGCAGCTCGGCCCTCCCCAGCCCACAGCGCCGTTATTCCGTTTCTATATCAGTAAACACATTTCATTTTCCGTAGACCAGGGCGGGGTGACGGGTGATCCCAGTCCTCGCAGTGAATTCCGGGCAGCAAAATTCAAAACACATGCGGCCAAGGCCGGGCACGGTGGTTCACGCCTGTAATCCCAGCACTTTGGGAGGTCGAGGCGGGCGATCACCTGAGGTCGGGAGCTCGAGACCAACCTGACCAACATGGGGAAATCCCGTCTCTACTAAAAATATAAAATTAGACGGGCTTGGTGGTGAATGCCTGTAATCCCAGCTAGTCGGGAGGCTGAGGCAGGAGAATCGCTTAAACCTTGGAGGCGGAGGTTGCGGTGAGCCGAGATCGCGCCATTGCACTTCAGCCTGGGCAACAAGAGGGAAAACTCCGTCGCAAAAACTTTCGGGGGCGGAGCGGAGCCCCGCCCTGGGTTATGTAAGCGACCGCGCTGGGCCGTTTCTCTTTCTTTTCCGGACCCTGCAGTGGCGCCTAAAGTCTGAGAGAGGGAAGTCGCCTCTGTGCTCGTGAGTGCATGGGGTATAAGGCAAGTGCTGAGGGAGAAAACGTAGTTGATGGGGTAGAGCAGACGGGGTTGGAGGTGGGGTGGAGGGGGAGGGCTTTGGACAGAAGACCTGGGAGGCTTGGTGGGGGAGGGGCGCCCAGGCCTGGGCACTAAGAAACAAGTCCCCTGGAGCTCAAGACCATCTCGGCCTCCCCTAGCCCAAGAGAGGACTGGCTTCATGACTCCCTGAAACCATTTCTAAATGCCTTAGAACAAACCTTGCATATTCATTATTGTTATTGAACTATTAAAAGTCTTTTTTGGGGGCGAGCTGAATCAGATCCTTTGCTGGAGCTGGCACACGGAGGAAGTCCTGGAGGGAGGGTAGACACCGTGGAGGTAAGGGCTTGGGACCTGTGTCAGGTGAGACCAAGTCGTGAGAGGTACTGCACCACGAAGTGGTTTAAACTATCAGTGTTTGACAGGATATATTGGCGGGTAAACCTAAGAGAAAA

**Assembled sequence**

Insert 1

ACTGGGGTGGATGCAGTGGGCCCCACTCTGTGGTGCAGTACCTCTCACGACTTGGTCTCACGTTGTGGGGCTTAGCTTCATATTTTCAAACTGAAATATTCTCTTCCTTAACCTCCACATAAATCCAAGTTTATAATTTTTATTATTTTAAAATTTTATTTATTTTTCTGTTTTGGGGACAGGGTCTCCTCCTGTCACTCAGGCTGGAGTGCAATGGCACAATCATAGCTCACTGCAGCCTGGAACTCCTGGGCTTAAGCAATCTTCCTGCCTTCGATTCCCAAAGAGCTGGGATTATAGTCATGAACCACTGCAATCCACCCAAATCCAAGTTTACACTAAAAGATAAAATTCCAACATTGTAGGGGATTGGTCAGGTGGTGGGAATAATTATAAAGATAAAGTTATAGGAAATAGACACAAACCTTCTTGGAAGGTGGAAAGTTTTGCAAAAGCCTCAGGATAGGGTTATAGCTGAAAGCAGCCTAATCCCCTTACCTTGAGTTAATAGCTTCGAGTAAGTACAAAGACATGTAAGAGAGTTTATCTAAAGAGCATGTTTACCTTTGATCATTTGTAGGACTGCTCTCTCTGGGGGACTGCGACCAGATTAATTACCCACAGGTGTGTTGACTCAAAGCCTTTGTCATTAAATCTGTGCTGAATAAAGGCCCACAGGGCCAGATAGTCAGGGCACGCAGCTGCCACAACCCTTTCTGTGAGTGGCCTGGCCCTCTGGTGCACTCTTTCACTGAATATCGGTGTCTGAGTACATTATTCATCCATCGTGCAGCCTGGGTCTGCCGGTCAGACCCTGGCACAACATTTAAGAGGAAATGAAAGTCACAAAGTTATCCCAGTCTCTGGAGTCACTGTCAAAACTTTGGTGAGGAATCTTCCAGGTTTTCCCCTACTTCAAATATATATTAATATTATGTAAGTGATATTAGTGGCATTT

Inserts 2, 3 and 4

GCGGTGGCTCACGCCTGTAATCCCAGCACTTTGAGAGGCCAAGGCAGGCAGATCACGAGGTCAGGAGATCGAGACCATCCTGGCTAACACAGTGAAACCCCGTCTCTACTAAAAATACAAAAAATTAGTCGGGCGTGGTGGCAGGAGCCTGTAGTCCCAGCTACTCCAGAGGCTGAGGCAGCAGAATGCCCTGAACCCGGGAGGCGGAGCTTGCAGTGAGCCAAGATCGCGCCACTGCACTCCAGCCTGGGCGACAGAGCAAGACTCCGTCTCAAAAAAAAAAAACAAAAAACAAAAAAACTCTCCTTTACTTTTTCTCTCCCCTTTTCTTCCTATCTCTTCCCTCATTTCTTCAACACGTCCCCCCATCCTTCCCTCTTTTCTCCATTCTCTGCATTTGATCCCCGGTATATTCCAGCCTCCAGGCCAACAAACTTCTCCGCGTCCGCCGGGAGCAGGTCAGGGAAGGGACGCGAGGCGGCGCTGTCACCGCATTCTGAGCGCCGCAGCTCCCTGGGCCCCTTGTATCATTTCAGTGAAGGTCACTCCAGTCTTTCATGGAGGCCAAACTAAGGGTGTAAATTAGGATCCTCACTGAAGTGGCGGGACCCTAAGAGGCTTTTTCCTGGCCCCTTAGTTGTGGGTTTTCCTGCGGGCGGCGCAGCCGGTTTCCATCAGAACCGCCCAGAGGCGGACGCTGCCTTCCTGGGGTGACGGAGCAGCAGGAAGCGTTTTCGGATCCTGGAATACGTGGGCGGCCCGTGGGAGGGGCTGAGGCGCAGTTTCCTACTCACCCGGATCCGAATCCTCCGCGGTGCTGTTTCAAGAGAGCCGGATTCCAGATCGCGCTTCAGCCCGGACTCGGATTTCCTGCCCTGCGGGTCTGCATTTTCATAACGGGCAGGTGTGAGTGCCCTGCAGCTGGAGACCAGAAGCCTGAAGGCAGCTCGGCCCTCCCCAGCCCACAGCGCCGTTATTCCGTTTCTATATCAGTAAACACATTTCATTTTCCGTAGACCAGGGCGGGGTGACGGGTGATCCCAGTCCTCGCAGTGAATTCCGGGCAGCAAAATTCAAAACACATGCGGCCAAGGCCGGGCACGGTGGTTCACGCCTGTAATCCCAGCACTTTGGGAGGTCGAGGCGGGCGATCACCTGAGGTCGGGAGCTCGAGACCAACCTGACCAACATGGGGAAATCCCGTCTCTACTAAAAATATAAAATTAGACGGGCTTGGTGGTGAATGCCTGTAATCCCAGCTAGTCGGGAGGCTGAGGCAGGAGAATCGCTTAAACCTTGGAGGCGGAGGTTGCGGTGAGCCGAGATCGCGCCATTGCACTTCAGCCTGGGCAACAAGAGGGAAAACTCCGTCGCAAAAACTTTCGGGGGCGGAGCGGAGCCCCGCCCTGGGTTATGTAAGCGACCGCGCTGGGCCGTTTCTCTTTCTTTTCCGGACCCTGCAGTGGCGCCTAAAGTCTGAGAGAGGGAAGTCGCCTCTGTGCTCGTGAGTGCATGGGGTATAAGGCAAGTGCTGAGGGAGAAAACGTAGTTGATGGGGTAGAGCAGACGGGGTTGGAGGTGGGGTGGAGGGGGAGGGCTTTGGACAGAAGACCTGGGAGGCTTGGTGGGGGAGGGGCGCCCAGGCCTGGGCACTAAGAAACAAGTCCCCTGGAGCTCAAGACCATCTCGGCCTCCCCTAGCCCAAGAGAGGACTGGCTTCATGACTCCCTGAAACCATTTCTAAATGCCTTAGAACAAACCTTGCATATTCATTATTGTTATTGAACTATTAAAAGTCTTTTTTGGGGGCGAGCTGAATCAGATCCTTTGCTGGAGCTGGCACACGGAGGAAGTCCTGGAGGGAGGGTAGACACCGTGGAGGTAAGGGCTTGGGACCTGTGTCAGGTGAGACCAAGTCGTGAGAGGTACTGCACCACGAAGTGGTTTAAACTATCAGTGTTTGACAGGATATATTGGCGGGTAAACCTAAGAGAAAA

## Colony 3

Forward CO9566: TGGTGTAAACAAATTGACGC

NNNNNNNNNNNGGACGTTTTTATGTACTGGGGTGGATGCAGTGGGCCCCACTCTGTGGTGCAGTACCTCTCACGACTTGGTCTCACGTTGTGGGGCTTAGCTTCATATTTTCAAACTGAAATATTCTCTTCCTTAACCTCCACATAAATCCAAGTTTATAATTTTTATTATTTTAAAATTTTATTTATTTTTCTGTTTTGGGGACAGGGTCTCCTCCTGTCACTCAGGCTGGAGTGCAATGGCACAATCATAGCTCACTGCAGCCTGGAACTCCTGGGCTTAAGCAATCTTCCTGCCTTCGATTCCCAAAGAGCTGGGATTATAGTCATGAACCACTGCAATCCACCCAAATCCAAGTTTACACTAAAAGATAAAATTCCAACATTGTAGGGGATTGGTCAGGTGGTGGGAATAATTATAAAGATAAAGTTATAGGAAATAGACACAAACCTTCTTGGAAGGTGGAAAGTTTTGCAAAAGCCTCAGGATAGGGTTATAGCTGAAAGCAGCCTAATCCCCTTACCTTGAGTTAATAGCTTCGAGTAAGTACAAAGACATGTAAGAGAGTTTATCTAAAGAGCATGTTTACCTTTGATCATTTGTAGGACTGCTCTCTCTGGGGGACTGCGACCAGATTAATTACCCACAGGTGTGTTGACTCAAAGCCTTTGTCATTAAATCTGTGCTGAATAAAGGCCCACAGGGCCAGATAGTCAGGGCACGCAGCTGCCACAACCCTTTCTGTGAGTGGCCTGGCCCTCTGGTGCACTCTTTCACTGAATATCGGTGTCTGAGTACATTATTCATCCATCGTGCAGCCTGGGTCTGCCGGTCAGACCCTGGCACAACATTTAAGAGGAAATGAAAGTCACAAAGTTATCCCAGTCTCTGGAGTCACTGTCAAAACTTTGGTGAGGAATCTTCCAGGTTTTCCCCTACTTCAAATATATATTAATATTATGTAAGTGATATTAGTGGCATTTTCGCCCA

Forward CO9574: AGAAATAATGAAACTACGTC

NNNNNNNCNNNNNGGNGCGGNGGCTCACGCCTGTAATCCCAGCACTTTGAGAGGCCAAGGCAGGCAGATCACGAGGTCAGGAGATCGAGACCATCCTGGCTAACACAGTGAAACCCCGTCTCTACTAAAAATACAAAAAATTAGTCGGGCGTGGTGGCAGGAGCCTGTAGTCCCAGCTACTCCAGAGGCTGAGGCAGCAGAATGCCCTGAACCCGGGAGGCGGAGCTTGCAGTGAGCCAAGATCGCGCCACTGCACTCCAGCCTGGGCGACAGAGCAAGACTCCGTCTCAAAAAAAAAAAACAAAAAACAAAAAAACTCTCCTTTACTTTTTCTCTCCCCTTTTCTTCCTATCTCTTCCCTCATTTCTTCAACACGTCCCCCCATCCTTCCCTCTTTTCTCCATTCTCTGCATTTGATCCCCGGTATATTCCAGCCTCCAGGCCAACAAACTTCTCCGCGTCCGCCGGGAGCAGGTCAGGGAAGGGACGCGAGGCGGCGCTGTCACCGCATTCTGAGCGCCGCAGCTCCCTGGGCCCCTTGTATCATTTCAGTGAAGGTCACTCCAGTCTTTCATGGAGGCCAAACTAAGGGTGTAAATTAGGATCCTCACTGAAGTGGCGGGACCCTAAGAGGCTTTTTCCTGGCCCCTTAGTTGTGGGTTTTCCTGCGGGCGGCGCAGCCGGTTTCCATCAGAACCGCCCAGAGGCGGACGCTGCCTTCCTGGGGTGACGGAGCAGCAGGAAGCGTTTTCGGATCCTGGAATACGTGGGCGGCCCGTGGGAGGGGCTGAGGCGCAGTTTCCTACTCACCCGGATCCGAATCCTCCGCGGTGCTGTTTCAAGAGAGCCGGATTCCAGATCGCGCTCCAGCCCGGACTCGGAATTCCTGCCCTGCGGGTCTGCATTTTCATAACGGGCAGGTGTGAGTGCCCTGCAGCTGGAGACCAGAAGCCTGAAGGCAGCTCGGCCCTCCCCANNCCACAGCGCCGTTATTCCGTTTCTATATCAGTAAACACATTTCATTTTCCGTAGACCAGGNCGGGGTGACGGGTGATCCNANTCCTCGCAGTGAATTCCGGTCNNCATANTTCANANNACATGCGGNCACGCCNGTGCTNGNNGNTCCNNNCTGNNATCCNNNNCTTNGGAAGNNN

Reverse CO9567: ACGCCCTTTTAAATATCCG

NNNNNNNNNNNCTTAGGTTTACCCGCCNATATATCCTGTCAAACACTGATAGTTTAAACCACTTCGTGGTGCAGTACCTCTCACGACTTGGTCTCACCTGACACAGGTCCCAAGCCCTTACCTCCACGGTGTCTACCCTCCCTCCAGGACTTCCTCCGTGTGCCAGCTCCAGCAAAGGATCTGATTCAGCTCGCCCCCAAAAAAGACTTTTAATAGTTCAATAACAATAATGAATATGCAAGGTTTGTTCTAAGGCATTTAGAAATGGTTTCAGGGAGTCATGAAGCCAGTCCTCTCTTGGGCTAGGGGAGGCCGAGATGGTCTTGAGCTCCAGGGGACTTGTTTCTTAGTGCCCAGGCCTGGGCGCCCCTCCCCCACCAAGCCTCCCAGGTCTTCTGTCCAAAGCCCTCCCCCTCCACCCCACCTCCAACCCCGTCTGCTCTACCCCATCAACTACGTTTTCTCCCTCAGCACTTGCCTTATACCCCATGCACTCACGAGCACAGAGGCGACTTCCCTCTCTCAGACTTTAGGCGCCACTGCAGGGTCCGGAAAAGAAAGAGAAACGGCCCAGCGCGGTCGCTTACATAACCCAGGGCGGGGCTCCGCTCCGCCCCCGAAAGTTTTTGCGACGGAGTTTTCCCTCTTGTTGCCCAGGCTGAAGTGCAATGGCGCGATCTCGGCTCACCGCAACCTCCGCCTCCAAGGTTTAAGCGATTCTCCTGCCTCAGCCTCCCGACTAGCTGGGATTACAGGCATTCACCACCAAGCCCGTCTAATTTTATATTTTTAGTAGAGACGGGATTTCCCCATGTTGGTCAGGTTGGTCTCGAGCTCCCGACCTCAGGTGATCGCCCGCCTCGACCTCCCAAAGTGCTGGGATTACAGGCGTGAACCACCGTGCCCGGCCTTGGCCGCATGTGTTTTGAATTTTGCTGCCCGGAATTCACTGCGAGGACTGGGATCACCCGTCACCCCGCCCTGGTCTACGGAAAATGAAATGTGTTTACTGATATAGAAACGGAATAACGGCGCTGTGGGCTGGGGAGGGCCGAGCTGCCTTCAGGCTTCTGGTCTCCAGCTGCAGGGCACTCACACCTGCCCGTTATGAAAATGCAGACCCGCAGGGCAGGAATTCCAAGTCCGGCCTGGAGCGCGATCTGGAATCCGGNTCTCTTGAAANAGCACCGCGGNAGGATTCGGATCCGGGTGANAAGGAAACTGNNCCTCAGGCCCNNCCCACGGGCCGCCCCNGT

Reverse Complement Reverse CO9567: ACGCCCTTTTAAATATCCG

CCGGATTCCAGATCGCGCTCCAGGCCGGACTTGGAATTCCTGCCCTGCGGGTCTGCATTTTCATAACGGGCAGGTGTGAGTGCCCTGCAGCTGGAGACCAGAAGCCTGAAGGCAGCTCGGCCCTCCCCAGCCCACAGCGCCGTTATTCCGTTTCTATATCAGTAAACACATTTCATTTTCCGTAGACCAGGGCGGGGTGACGGGTGATCCCAGTCCTCGCAGTGAATTCCGGGCAGCAAAATTCAAAACACATGCGGCCAAGGCCGGGCACGGTGGTTCACGCCTGTAATCCCAGCACTTTGGGAGGTCGAGGCGGGCGATCACCTGAGGTCGGGAGCTCGAGACCAACCTGACCAACATGGGGAAATCCCGTCTCTACTAAAAATATAAAATTAGACGGGCTTGGTGGTGAATGCCTGTAATCCCAGCTAGTCGGGAGGCTGAGGCAGGAGAATCGCTTAAACCTTGGAGGCGGAGGTTGCGGTGAGCCGAGATCGCGCCATTGCACTTCAGCCTGGGCAACAAGAGGGAAAACTCCGTCGCAAAAACTTTCGGGGGCGGAGCGGAGCCCCGCCCTGGGTTATGTAAGCGACCGCGCTGGGCCGTTTCTCTTTCTTTTCCGGACCCTGCAGTGGCGCCTAAAGTCTGAGAGAGGGAAGTCGCCTCTGTGCTCGTGAGTGCATGGGGTATAAGGCAAGTGCTGAGGGAGAAAACGTAGTTGATGGGGTAGAGCAGACGGGGTTGGAGGTGGGGTGGAGGGGGAGGGCTTTGGACAGAAGACCTGGGAGGCTTGGTGGGGGAGGGGCGCCCAGGCCTGGGCACTAAGAAACAAGTCCCCTGGAGCTCAAGACCATCTCGGCCTCCCCTAGCCCAAGAGAGGACTGGCTTCATGACTCCCTGAAACCATTTCTAAATGCCTTAGAACAAACCTTGCATATTCATTATTGTTATTGAACTATTAAAAGTCTTTTTTGGGGGCGAGCTGAATCAGATCCTTTGCTGGAGCTGGCACACGGAGGAAGTCCTGGAGGGAGGGTAGACACCGTGGAGGTAAGGGCTTGGGACCTGTGTCAGGTGAGACCAAGTCGTGAGAGGTACTGCACCACGAAGTGGTTTAAACTATCAGTGTTTGACAGGATATATNGGCGGGTAAACCTAAG

**Assembled sequence**

Insert 1

GGACGTTTTTATGTACTGGGGTGGATGCAGTGGGCCCCACTCTGTGGTGCAGTACCTCTCACGACTTGGTCTCACGTTGTGGGGCTTAGCTTCATATTTTCAAACTGAAATATTCTCTTCCTTAACCTCCACATAAATCCAAGTTTATAATTTTTATTATTTTAAAATTTTATTTATTTTTCTGTTTTGGGGACAGGGTCTCCTCCTGTCACTCAGGCTGGAGTGCAATGGCACAATCATAGCTCACTGCAGCCTGGAACTCCTGGGCTTAAGCAATCTTCCTGCCTTCGATTCCCAAAGAGCTGGGATTATAGTCATGAACCACTGCAATCCACCCAAATCCAAGTTTACACTAAAAGATAAAATTCCAACATTGTAGGGGATTGGTCAGGTGGTGGGAATAATTATAAAGATAAAGTTATAGGAAATAGACACAAACCTTCTTGGAAGGTGGAAAGTTTTGCAAAAGCCTCAGGATAGGGTTATAGCTGAAAGCAGCCTAATCCCCTTACCTTGAGTTAATAGCTTCGAGTAAGTACAAAGACATGTAAGAGAGTTTATCTAAAGAGCATGTTTACCTTTGATCATTTGTAGGACTGCTCTCTCTGGGGGACTGCGACCAGATTAATTACCCACAGGTGTGTTGACTCAAAGCCTTTGTCATTAAATCTGTGCTGAATAAAGGCCCACAGGGCCAGATAGTCAGGGCACGCAGCTGCCACAACCCTTTCTGTGAGTGGCCTGGCCCTCTGGTGCACTCTTTCACTGAATATCGGTGTCTGAGTACATTATTCATCCATCGTGCAGCCTGGGTCTGCCGGTCAGACCCTGGCACAACATTTAAGAGGAAATGAAAGTCACAAAGTTATCCCAGTCTCTGGAGTCACTGTCAAAACTTTGGTGAGGAATCTTCCAGGTTTTCCCCTACTTCAAATATATATTAATATTATGTAAGTGATATTAGTGGCATTTTCGCCCA

Inserts 2, 3 and 4

GGCTCACGCCTGTAATCCCAGCACTTTGAGAGGCCAAGGCAGGCAGATCACGAGGTCAGGAGATCGAGACCATCCTGGCTAACACAGTGAAACCCCGTCTCTACTAAAAATACAAAAAATTAGTCGGGCGTGGTGGCAGGAGCCTGTAGTCCCAGCTACTCCAGAGGCTGAGGCAGCAGAATGCCCTGAACCCGGGAGGCGGAGCTTGCAGTGAGCCAAGATCGCGCCACTGCACTCCAGCCTGGGCGACAGAGCAAGACTCCGTCTCAAAAAAAAAAAACAAAAAACAAAAAAACTCTCCTTTACTTTTTCTCTCCCCTTTTCTTCCTATCTCTTCCCTCATTTCTTCAACACGTCCCCCCATCCTTCCCTCTTTTCTCCATTCTCTGCATTTGATCCCCGGTATATTCCAGCCTCCAGGCCAACAAACTTCTCCGCGTCCGCCGGGAGCAGGTCAGGGAAGGGACGCGAGGCGGCGCTGTCACCGCATTCTGAGCGCCGCAGCTCCCTGGGCCCCTTGTATCATTTCAGTGAAGGTCACTCCAGTCTTTCATGGAGGCCAAACTAAGGGTGTAAATTAGGATCCTCACTGAAGTGGCGGGACCCTAAGAGGCTTTTTCCTGGCCCCTTAGTTGTGGGTTTTCCTGCGGGCGGCGCAGCCGGTTTCCATCAGAACCGCCCAGAGGCGGACGCTGCCTTCCTGGGGTGACGGAGCAGCAGGAAGCGTTTTCGGATCCTGGAATACGTGGGCGGCCCGTGGGAGGGGCTGAGGCGCAGTTTCCTACTCACCCGGATCCGAATCCTCCGCGGTGCTGTTTCAAGAGAGCCGGATTCCAGATCGCGCTCCAGGCCGGACTTGGAATTCCTGCCCTGCGGGTCTGCATTTTCATAACGGGCAGGTGTGAGTGCCCTGCAGCTGGAGACCAGAAGCCTGAAGGCAGCTCGGCCCTCCCCAGCCCACAGCGCCGTTATTCCGTTTCTATATCAGTAAACACATTTCATTTTCCGTAGACCAGGGCGGGGTGACGGGTGATCCCAGTCCTCGCAGTGAATTCCGGGCAGCAAAATTCAAAACACATGCGGCCAAGGCCGGGCACGGTGGTTCACGCCTGTAATCCCAGCACTTTGGGAGGTCGAGGCGGGCGATCACCTGAGGTCGGGAGCTCGAGACCAACCTGACCAACATGGGGAAATCCCGTCTCTACTAAAAATATAAAATTAGACGGGCTTGGTGGTGAATGCCTGTAATCCCAGCTAGTCGGGAGGCTGAGGCAGGAGAATCGCTTAAACCTTGGAGGCGGAGGTTGCGGTGAGCCGAGATCGCGCCATTGCACTTCAGCCTGGGCAACAAGAGGGAAAACTCCGTCGCAAAAACTTTCGGGGGCGGAGCGGAGCCCCGCCCTGGGTTATGTAAGCGACCGCGCTGGGCCGTTTCTCTTTCTTTTCCGGACCCTGCAGTGGCGCCTAAAGTCTGAGAGAGGGAAGTCGCCTCTGTGCTCGTGAGTGCATGGGGTATAAGGCAAGTGCTGAGGGAGAAAACGTAGTTGATGGGGTAGAGCAGACGGGGTTGGAGGTGGGGTGGAGGGGGAGGGCTTTGGACAGAAGACCTGGGAGGCTTGGTGGGGGAGGGGCGCCCAGGCCTGGGCACTAAGAAACAAGTCCCCTGGAGCTCAAGACCATCTCGGCCTCCCCTAGCCCAAGAGAGGACTGGCTTCATGACTCCCTGAAACCATTTCTAAATGCCTTAGAACAAACCTTGCATATTCATTATTGTTATTGAACTATTAAAAGTCTTTTTTGGGGGCGAGCTGAATCAGATCCTTTGCTGGAGCTGGCACACGGAGGAAGTCCTGGAGGGAGGGTAGACACCGTGGAGGTAAGGGCTTGGGACCTGTGTCAGGTGAGACCAAGTCGTGAGAGGTACTGCACCACGAAGTGGTTTAAACTATCAGTGTTTGACAGGATATAT

## Colony 4

Forward CO9566: TGGTGTAAACAAATTGACGC

NNNNNNNNNNTNNGGACGTTTTTATGTACTGGGGTGGATGCAGTGGGCCCCACTCTGTGGTGCAGTACCTCTCACGACTTGGTCTCACGTTGTGGGGCTTAGCTTCATATTTTCAAACTGAAATATTCTCTTCCTTAACCTCCACATAAATCCAAGTTTATAATTTTTATTATTTTAAAATTTTATTTATTTTTCTGTTTTGGGGACAGGGTCTCCTCCTGTCACTCAGGCTGGAGTGCAATGGCACAATCATAGCTCACTGCAGCCTGGAACTCCTGGGCTTAAGCAATCTTCCTGCCTTCGATTCCCAAAGAGCTGGGATTATAGTCATGAACCACTGCAATCCACCCAAATCCAAGTTTACACTAAAAGATAAAATTCCAACATTGTAGGGGATTGGTCAGGTGGTGGGAATAATTATAAAGATAAAGTTATAGGAAATAGACACAAACCTTCTTGGAAGGTGGAAAGTTTTGCAAAAGCCTCAGGATAGGGTTATAGCTGAAAGCAGCCTAATCCCCTTACCTTGAGTTAATAGCTTCGAGTAAGTACAAAGACATGTAAGAGAGTTTATCTAAAGAGCATGTTTACCTTTGATCATTTGTAGGACTGCTCTCTCTGGGGGACTGCGACCAGATTAATTACCCACAGGTGTGTTGACTCAAAGCCTTTGTCATTAAATCTGTGCTGAATAAAGGCCCACAGGGCCAGATAGTCAGGGCACGCAGCTGCCACAACCCTTTCTGTGAGTGGCCTGGCCCTCTGGTGCACTCTTTCACTGAATATCGGTGTCTGAGTACATTATTCATCCATCGTGCAGCCTGGGTCTGCCGGTCAGACCCTGGCACAACATTTAAGAGGAAATGAAAGTCACAAAGTTATCCCAGTCTCTGGAGTCACTGTCAAAACTTTGGTGAGGAATCTTCCAGGTTTTCCCCTACTTCAAATATATATTAATATTATGTAAGTGATATTAGTGGCATTTTCGCC

Forward CO9574: AGAAATAATGAAACTACGTC

NNNNNNNCNNNNNGGNGCGGTGGCTCACGCCTGTAATCCCAGCACTTTGAGAGGCCAAGGCAGGCAGATCACGAGGTCAGGAGATCGAGACCATCCTGGCTAACACAGTGAAACCCCGTCTCTACTAAAAATACAAAAAATTAGTCGGGCGTGGTGGCAGGAGCCTGTAGTCCCAGCTACTCCAGAGGCTGAGGCAGCAGAATGCCCTGAACCCGGGAGGCGGAGCTTGCAGTGAGCCAAGATCGCGCCACTGCACTCCAGCCTGGGCGACAGAGCAAGACTCCGTCTCAAAAAAAAAAAACAAAAAACAAAAAAACTCTCCTTTACTTTTTCTCTCCCCTTTTCTTCCTATCTCTTCCCTCATTTCTTCAACACGTCCCCCCATCCTTCCCTCTTTTCTCCATTCTCTGCATTTGATCCCCGGTATATTCCAGCCTCCAGGCCAACAAACTTCTCCGCGTCCGCCGGGAGCAGGTCAGGGAAGGGACGCGAGGCGGCGCTGTCACCGCATTCTGAGCGCCGCAGCTCCCTGGGCCCCTTGTATCATTTCAGTGAAGGTCACTCCAGTCTTTCATGGAGGCCAAACTAAGGGTGTAAATTAGGATCCTCACTGAAGTGGCGGGACCCTAAGAGGCTTTTTCCTGGCCCCTTAGTTGTGGGTTTTCCTGCGGGCGGCGCAGCCGGTTTCCATCAGAACCGCCCAGAGGCGGACGCTGCCTTCCTGGGGTGACGGAGCAGCAGGAAGCGTTTTCGGATCCTGGAATACGTGCGCGGCCCGTGGGAGGGGCTGAGGCGCAGTTTCCTACTCACCCGGATCCGAATCCTCCGCGGTGCTGTTTCAAGAGAGCCGGATTCCAGATCGCGCTCCAGCCCGGACTCGGAATTCCTGCCCTGCGGGTCTGCATTTTCATAACGGGCAGANNTGAGTGCNCTGAAGCTGTAGACCNNAAGCCTGAATGCAGCTCGGCNCTCCCCAGNNCACAGCGNNGTTATNTCNNTTNNANNTNANNACANACANNTCNNTTTTCTGTANACCANGGGCGGNNGACGGNTG

Reverse CO9567: ACGCCCTTTTAAATATCCG

NNNNNNNTTNTCTTAGGTTTACCCGCCAATATATCCTGTCAAACACTGATAGTTTAAACCACTTCGTGGTGCAGTACCTCTCACGACTTGGTCTCACCTGACACAGGTCCCAAGCCCTTACCTCCACGGTGTCTACCCTCCCTCCAGGACTTCCTCCGTGTGCCAGCTCCAGCAAAGGATCTGATTCAGCTCGCCCCCAAAAAAGACTTTTAATAGTTCAATAACAATAATGAATATGCAAGGTTTGTTCTAAGGCATTTAGAAATGGTTTCAGGGAGTCATGAAGCCAGTCCTCTCTTGGGCTAGGGGAGGCCGAGATGGTCTTGAGCTCCAGGGGACTTGTTTCTTAGTGCCCAGGCCTGGGCGCCCCTCCCCCACCAAGCCTCCCAGGTCTTCTGTCCAAAGCCCTCCCCCTCCACCCCACCTCCAACCCCGTCTGCTCTACCCCATCAACTACGTTTTCTCCCTCAGCACTTGCCTTATACCCCATGCACTCACGAGCACAGAGGCGACTTCCCTCTCTCAGACTTTAGGCGCCACTGCAGGGTCCGGAAAAGAAAGAGAAACGGCCCAGCGCGGTCGCTTACATAACCCAGGGCGGGGCTCCGCTCCGCCCCCGAAAGTTTTTGCGACGGAGTTTTCCCTCTTGTTGCCCAGGCTGAAGTGCAATGGCGCGATCTCGGCTCACCGCAACCTCCGCCTCCAAGGTTTAAGCGATTCTCCTGCCTCAGCCTCCCGACTAGCTGGGATTACAGGCATTCACCACCAAGCCCGTCTAATTTTATATTTTTAGTAGAGACGGGATTTCCCCATGTTGGTCAGGTTGGTCTCGAGCTCCCGACCTCAGGTGATCGCCCGCCTCGACCTCCCAAAGTGCTGGGATTACAGGCGTGAACCACCGTGCCCGGCCTTGGCCGCATGTGTTTTGAATTTTGCTGCCCGGAATTCACTGCGAGGACTGGGATCACCCGTCACCCCGCCCTGGTCTACGGAAAATGAAATGTGTTTACTGATATAGAAACGGAATAACGGCGCTGTGGGCTGGGGAGGGCCGAGCTGCCTTCAGGCTTCTGGTCTCCAGCTGCAGGGCACTCACACCTGCCCGTTATGAAAATGCAGACCCGCAGGGCAGGAATTCCGAGTCCGGGCTGGAACGCGATCTGGAATCGGCTCTCTTGAAACANNNCCGCGGAGAATCGGAACCGGNTNNNAAGAAACTGCGCCTCAACCCTTCCNNGGGCCGNCCANGNNTTCCAGGATCCGAANNNCTTCCTGNTGCTCCNNNNCCCCANNNANGNNNNNNNCGCNNNTGGNTGTTTNNGATGAANANNGAT

Reverse Complement Reverse CO9567: ACGCCCTTTTAAATATCCG

TGTTTCAAGAGAGCCGATTCCAGATCGCGTTCCAGCCCGGACTCGGAATTCCTGCCCTGCGGGTCTGCATTTTCATAACGGGCAGGTGTGAGTGCCCTGCAGCTGGAGACCAGAAGCCTGAAGGCAGCTCGGCCCTCCCCAGCCCACAGCGCCGTTATTCCGTTTCTATATCAGTAAACACATTTCATTTTCCGTAGACCAGGGCGGGGTGACGGGTGATCCCAGTCCTCGCAGTGAATTCCGGGCAGCAAAATTCAAAACACATGCGGCCAAGGCCGGGCACGGTGGTTCACGCCTGTAATCCCAGCACTTTGGGAGGTCGAGGCGGGCGATCACCTGAGGTCGGGAGCTCGAGACCAACCTGACCAACATGGGGAAATCCCGTCTCTACTAAAAATATAAAATTAGACGGGCTTGGTGGTGAATGCCTGTAATCCCAGCTAGTCGGGAGGCTGAGGCAGGAGAATCGCTTAAACCTTGGAGGCGGAGGTTGCGGTGAGCCGAGATCGCGCCATTGCACTTCAGCCTGGGCAACAAGAGGGAAAACTCCGTCGCAAAAACTTTCGGGGGCGGAGCGGAGCCCCGCCCTGGGTTATGTAAGCGACCGCGCTGGGCCGTTTCTCTTTCTTTTCCGGACCCTGCAGTGGCGCCTAAAGTCTGAGAGAGGGAAGTCGCCTCTGTGCTCGTGAGTGCATGGGGTATAAGGCAAGTGCTGAGGGAGAAAACGTAGTTGATGGGGTAGAGCAGACGGGGTTGGAGGTGGGGTGGAGGGGGAGGGCTTTGGACAGAAGACCTGGGAGGCTTGGTGGGGGAGGGGCGCCCAGGCCTGGGCACTAAGAAACAAGTCCCCTGGAGCTCAAGACCATCTCGGCCTCCCCTAGCCCAAGAGAGGACTGGCTTCATGACTCCCTGAAACCATTTCTAAATGCCTTAGAACAAACCTTGCATATTCATTATTGTTATTGAACTATTAAAAGTCTTTTTTGGGGGCGAGCTGAATCAGATCCTTTGCTGGAGCTGGCACACGGAGGAAGTCCTGGAGGGAGGGTAGACACCGTGGAGGTAAGGGCTTGGGACCTGTGTCAGGTGAGACCAAGTCGTGAGAGGTACTGCACCACGAAGTGGTTTAAACTATCAGTGTTTGACAGGATATATTGGCGGGTAAACCTAAGA

**Assembled sequence**

Insert 1

GGACGTTTTTATGTACTGGGGTGGATGCAGTGGGCCCCACTCTGTGGTGCAGTACCTCTCACGACTTGGTCTCACGTTGTGGGGCTTAGCTTCATATTTTCAAACTGAAATATTCTCTTCCTTAACCTCCACATAAATCCAAGTTTATAATTTTTATTATTTTAAAATTTTATTTATTTTTCTGTTTTGGGGACAGGGTCTCCTCCTGTCACTCAGGCTGGAGTGCAATGGCACAATCATAGCTCACTGCAGCCTGGAACTCCTGGGCTTAAGCAATCTTCCTGCCTTCGATTCCCAAAGAGCTGGGATTATAGTCATGAACCACTGCAATCCACCCAAATCCAAGTTTACACTAAAAGATAAAATTCCAACATTGTAGGGGATTGGTCAGGTGGTGGGAATAATTATAAAGATAAAGTTATAGGAAATAGACACAAACCTTCTTGGAAGGTGGAAAGTTTTGCAAAAGCCTCAGGATAGGGTTATAGCTGAAAGCAGCCTAATCCCCTTACCTTGAGTTAATAGCTTCGAGTAAGTACAAAGACATGTAAGAGAGTTTATCTAAAGAGCATGTTTACCTTTGATCATTTGTAGGACTGCTCTCTCTGGGGGACTGCGACCAGATTAATTACCCACAGGTGTGTTGACTCAAAGCCTTTGTCATTAAATCTGTGCTGAATAAAGGCCCACAGGGCCAGATAGTCAGGGCACGCAGCTGCCACAACCCTTTCTGTGAGTGGCCTGGCCCTCTGGTGCACTCTTTCACTGAATATCGGTGTCTGAGTACATTATTCATCCATCGTGCAGCCTGGGTCTGCCGGTCAGACCCTGGCACAACATTTAAGAGGAAATGAAAGTCACAAAGTTATCCCAGTCTCTGGAGTCACTGTCAAAACTTTGGTGAGGAATCTTCCAGGTTTTCCCCTACTTCAAATATATATTAATATTATGTAAGTGATATTAGTGGCATTTTCGCC

Inserts 2 and 3

GCGGTGGCTCACGCCTGTAATCCCAGCACTTTGAGAGGCCAAGGCAGGCAGATCACGAGGTCAGGAGATCGAGACCATCCTGGCTAACACAGTGAAACCCCGTCTCTACTAAAAATACAAAAAATTAGTCGGGCGTGGTGGCAGGAGCCTGTAGTCCCAGCTACTCCAGAGGCTGAGGCAGCAGAATGCCCTGAACCCGGGAGGCGGAGCTTGCAGTGAGCCAAGATCGCGCCACTGCACTCCAGCCTGGGCGACAGAGCAAGACTCCGTCTCAAAAAAAAAAAACAAAAAACAAAAAAACTCTCCTTTACTTTTTCTCTCCCCTTTTCTTCCTATCTCTTCCCTCATTTCTTCAACACGTCCCCCCATCCTTCCCTCTTTTCTCCATTCTCTGCATTTGATCCCCGGTATATTCCAGCCTCCAGGCCAACAAACTTCTCCGCGTCCGCCGGGAGCAGGTCAGGGAAGGGACGCGAGGCGGCGCTGTCACCGCATTCTGAGCGCCGCAGCTCCCTGGGCCCCTTGTATCATTTCAGTGAAGGTCACTCCAGTCTTTCATGGAGGCCAAACTAAGGGTGTAAATTAGGATCCTCACTGAAGTGGCGGGACCCTAAGAGGCTTTTTCCTGGCCCCTTAGTTGTGGGTTTTCCTGCGGGCGGCGCAGCCGGTTTCCATCAGAACCGCCCAGAGGCGGACGCTGCCTTCCTGGGGTGACGGAGCAGCAGGAAGCGTTTTCGGATCCTGGAATACGTGCGCGGCCCGTGGGAGGGGCTGAGGCGCAGTTTCCTACTCACCCGGATCCGAATCCTCCGCGGTGCTGTTTCAAGAGAGCCGGATTCCAGATCGCGCTCCAGCCCGGACTCGGAATTCCTGCCCTGCGGGTCTGCATTTTCATAACGGGCAGA

Inserts 3 and 4

TGTTTCAAGAGAGCCGATTCCAGATCGCGTTCCAGCCCGGACTCGGAATTCCTGCCCTGCGGGTCTGCATTTTCATAACGGGCAGGTGTGAGTGCCCTGCAGCTGGAGACCAGAAGCCTGAAGGCAGCTCGGCCCTCCCCAGCCCACAGCGCCGTTATTCCGTTTCTATATCAGTAAACACATTTCATTTTCCGTAGACCAGGGCGGGGTGACGGGTGATCCCAGTCCTCGCAGTGAATTCCGGGCAGCAAAATTCAAAACACATGCGGCCAAGGCCGGGCACGGTGGTTCACGCCTGTAATCCCAGCACTTTGGGAGGTCGAGGCGGGCGATCACCTGAGGTCGGGAGCTCGAGACCAACCTGACCAACATGGGGAAATCCCGTCTCTACTAAAAATATAAAATTAGACGGGCTTGGTGGTGAATGCCTGTAATCCCAGCTAGTCGGGAGGCTGAGGCAGGAGAATCGCTTAAACCTTGGAGGCGGAGGTTGCGGTGAGCCGAGATCGCGCCATTGCACTTCAGCCTGGGCAACAAGAGGGAAAACTCCGTCGCAAAAACTTTCGGGGGCGGAGCGGAGCCCCGCCCTGGGTTATGTAAGCGACCGCGCTGGGCCGTTTCTCTTTCTTTTCCGGACCCTGCAGTGGCGCCTAAAGTCTGAGAGAGGGAAGTCGCCTCTGTGCTCGTGAGTGCATGGGGTATAAGGCAAGTGCTGAGGGAGAAAACGTAGTTGATGGGGTAGAGCAGACGGGGTTGGAGGTGGGGTGGAGGGGGAGGGCTTTGGACAGAAGACCTGGGAGGCTTGGTGGGGGAGGGGCGCCCAGGCCTGGGCACTAAGAAACAAGTCCCCTGGAGCTCAAGACCATCTCGGCCTCCCCTAGCCCAAGAGAGGACTGGCTTCATGACTCCCTGAAACCATTTCTAAATGCCTTAGAACAAACCTTGCATATTCATTATTGTTATTGAACTATTAAAAGTCTTTTTTGGGGGCGAGCTGAATCAGATCCTTTGCTGGAGCTGGCACACGGAGGAAGTCCTGGAGGGAGGGTAGACACCGTGGAGGTAAGGGCTTGGGACCTGTGTCAGGTGAGACCAAGTCGTGAGAGGTACTGCACCACGAAGTGGTTTAAACTATCAGTGTTTGACAGGATATATTGGCGGGTAAACCTAAGA

## Colony 5

Forward CO9566: TGGTGTAAACAAATTGACGC

NNNNNNNNNNNNNGGACGTTTTTATGTACTGGGGTGGATGCAGTGGGCCCCACTCTGTGGTGCAGTACCTCTCACGACTTGGTCTCACGTTGTGGGGCTTAGCTTCATATTTTCAAACTGAAATATTCTCTTCCTTAACCTCCACATAAATCCAAGTTTATAATTTTTATTATTTTAAAATTTTATTTATTTTTCTGTTTTGGGGACAGGGTCTCCTCCTGTCACTCAGGCTGGAGTGCAATGGCACAATCATAGCTCACTGCAGCCTGGAACTCCTGGGCTTAAGCAATCTTCCTGCCTTCGATTCCCAAAGAGCTGGGATTATAGTCATGAACCACTGCAATCCACCCAAATCCAAGTTTACACTAAAAGATAAAATTCCAACATTGTAGGGGATTGGTCAGGTGGTGGGAATAATTATAAAGATAAAGTTATAGGAAATAGACACAAACCTTCTTGGAAGGTGGAAAGTTTTGCAAAAGCCTCAGGATAGGGTTATAGCTGAAAGCAGCCTAATCCCCTTACCTTGAGTTAATAGCTTCGAGTAAGTACAAAGACATGTAAGAGAGTTTATCTAAAGAGCATGTTTACCTTTGATCATTTGTAGGACTGCTCTCTCTGGGGGACTGCGACCAGATTAATTACCCACAGGTGTGTTGACTCAAAGCCTTTGTCATTAAATCTGTGCTGAATAAAGGCCCACAGGGCCAGATAGTCAGGGCACGCAGCTGCCACAACCCTTTCTGTGAGTGGCCTGGCCCTCTGGTGCACTCTTTCACTGAATATCGGTGTCTGAGTACATTATTCATCCATCGTGCAGCCTGGGTCTGCCGGTCAGACCCTGGCACAACATTTAAGAGGAAATGAAAGTCACAAAGTTATCCCAGTCTCTGGAGTCACTGTCAAAACTTTGGTGAGGAATCTTCCAGGTTTTCCCCTACTTCAAATATATATTAATATTATGTAAGTGATATTAGTGGCATTTTTCGCCCAGGCTGGAATGCAGTGGCATGATCTCGGCTCACTCTAACCTCTACCTCCCAGATTCAAGCGATTCTCCTGCCTCAGCCTCCCAAACAGCTGGAACTACAGGCACCCACCACCACGCCCGGCTAATTTTTGCATTTTCNNAGAGACAGGGTTTCACCATGTTGGCCAGGCTGATCTTGAACTTCTGACCTCAGGTTATCTGCCTGCCATAGNNNCCAAAGTCTGGGATTACAGGCATGANCNNTGGGCCCAGCTCCCTAACCTTTTAAAANGGTTAAAAGNTGCTGGCNNATCTTNNNTAGCAATNCTTAANTTGCNNTTACNNTTTTAATNNNAACNAGAATTTNNNCGGGAACNGTNNTTNGGAANNAATAANTCTCTTNACTTCCTGGGNNNNN

Forward CO9574: AGAAATAATGAAACTACGTC

NNNNNNNNNNNNNGGNGCGGNGGCTCACGCCTGTAATCCCAGCACTTTGAGAGGCCAAGGCAGGCAGATCACGAGGTCAGGAGATCGAGACCATCCTGGCTAACACAGTGAAACCCCGTCTCTACTAAAAATACAAAAAATTAGTCGGGCGTGGTGGCAGGAGCCTGTAGTCCCAGCTACTCCAGAGGCTGAGGCAGCAGAATGCCCTGAACCCGGGAGGCGGAGCTTGCAGTGAGCCAAGATCGCGCCACTGCACTCCAGCCTGGGCGACAGAGCAAGACTCCGTCTCAAAAAAAAAAAACAAAAAACAAAAAAACTCTCCTTTACTTTTTCTCTCCCCTTTTCTTCCTATCTCTTCCCTCATTTCTTCAACACGTCCCCCCATCCTTCCCTCTTTTCTCCATTCTCTGCATTTGATCCCCGGTATATTCCAGCCTCCAGGCCAACAAACTTCTCCGCGTCCGCCGGGAGCAGGTCAGGGAAGGGACGCGAGGCGGCGCTGTCACCGCATTCTGAGCGCCGCAGCTCCCTGGGCCCCTTGTATCATTTCAGTGAAGGTCACTCCAGTCTTTCATGGAGGCCAAACTAAGGGTGTAAATTAGGATCCTCACTGAAGTGGCGGGACCCTAAGAGGCTTTTTCCTGGCCCCTTAGTTGTGGGTTTTCCTGCGGGCGGCGCAGCCGGTTTCCATCAGAACCGCCCAGAGGCGGACGCTGCCTTCCTGGGGTGACGGAGCAGCAGGAAGCGTTTTCGGATCCTGGAATACGTGGGCGGCCCGTGGNAGGGGCTGAGGCGCAGTTTCCTACTCACCCGGATCCGAATCCTCCGCGGNGCTGTTTCAAGAGAGCCGGATTCCAGATCGCGCTCCAGCCCGGACTCGGAATTCCTGCCCTGCGGGTCTGCATTTTCATAACGGNCAGGTGTGAGTGCCCTGCAGCTGGAGACCAGAAGCCTGAAGGCAGCTCGGACCTCCCAGNCACAGCGCCGTTATTNCGNTTCTATATCNANANACNNTTTCATTTCCTANAACAGTNCGNGATGACGGNNGAATNCAAGTCACACAAGNAAATACAGANNANAATTATTAAT

Reverse CO9567: ACGCCCTTTTAAATATCCG

NNNNNNTNNNNNNCTTANNNNTACCCGCCAATATATCCTGTCAAACACTGATAGTTTAAACCACTTCGTGGTGCAGTACCTCTCACGACTTGGTCTCACCTGACACAGGTCCCAAGCCCTTACCTCCACGGTGTCTACCCTCCCTCCAGGACTTCCTCCGTGTGCCAGCTCCAGCAAAGGATCTGATTCAGCTCGCCCCCAAAAAAGACTTTTAATAGTTCAATAACAATAATGAATATGCAAGGTTTGTTCTAAGGCATTTAGAAATGGTTTCAGGGAGTCATGAAGCCAGTCCTCTCTTGGGCTAGGGGAGGCCGAGATGGTCTTGAGCTCCAGGGGACTTGTTTCTTAGTGCCCAGGCCTGGGCGCCCCTCCCCCACCAAGCCTCCCAGGTCTTCTGTCCAAAGCCCTCCCCCTCCACCCCACCTCCAACCCCGTCTGCTCTACCCCATCAACTACGTTTTCTCCCTCAGCACTTGCCTTATACCCCATGCACTCACGAGCACAGAGGCGACTTCCCTCTCTCAGACTTTAGGCGCCACTGCAGGGTCCGGAAAAGAAAGAGAAACGGCCCAGCGCGGTCGCTTACATAACCCAGGGCGGGGCTCCGCTCCGCCCCCGAAAGTTTTTGCGACGGAGTTTTCCCTCTTGTTGCCCAGGCTGAAGTGCAATGGCGCGATCTCGGCTCACCGCAACCTCCGCCTCCAAGGTTTAAGCGATTCTCCTGCCTCAGCCTCCCGACTAGCTGGGATTACAGGCATTCACCACCAAGCCCGTCTAATTTTATATTTTTAGTAGAGACGGGATTTCCCCATGTTGGTCAGGTTGGTCTCGAGCTCCCGACCTCAGGTGATCGCCCGCCTCGACCTCCCAAAGTGCTGGGATTACAGGCGTGAACCACCGTGCCCGGCCTTGGCCGCATGTGTTTTGAATTTTGCTGCCCGGAATTCACTGCGAGGACTGGGATCACCCGTCACCCCGCCCTGGTCTACGGAAAATGAAATGTGTTTACTGATATAGAAACGGAATAACGGCGCTGTGGGCTGGGGAGGGCCGAGCTGCCTTCAGGCTTCTGGTCTCCAGCTGCAGGGCACTCACACCTGCCCGTTATGAAAATGCAGACCCGCAGGGCAGGAATTCCGAGTCCGGGCTGGAGCGCGATCTGGAATCCGGCTCTCTTGAAACAGCACCGCGGAGGATTCGGATCCGGGTGAGTAGGAAACTGCGCCTCAGCCCTTCCCACGGGNCCGCCCACGAATTCCAGGATCCGAAAACGCTTCCTGCTGNNCCGNNCCCCCAGGAAGGCANNGTCCGCCNNNGGNCGGTTCTGATGGAAANCGGCNGNNCCGCCNGCAGGAAAACCCNAACTAAGGGGCCAGGAAAAAGCCTCTTAAGGGCCCGCCANTTCAGGNAGGATCCTAATTNACCCCNTTNNTTTGNNNCCNCNNAAANNNNNGNNNNGGNNNNNNNNNAANNNNAAAAGGNGNCNCCNNGGNNTTNGNNGGGNNNTNAAAANNNGGGNNNNNNNGNNCNNNCNNNNNCCGNCNNNNNNNNNNNNNNNNNNNNNCNNNNGNNNANNNGNNNAAANAANNNNTTNNNNNNNNNNCNGNNNGNNNTNNNNNNNNNNCCNNNGGGTNNNNNNATNNNNGNNANNNNNNNAAAAANANNANNNNNNTNNNNGNGGNNGNNNNNNNNNNNNNNNNAAANNNGNGNNAAANNAANNNNNNNNNCNAANNNNNNNNNNNNNAAAAAAAANNNNNNNNNNNNNNNTNTTTTNNTNNNTTNTNNCTTNTTNTNNT

Reverse Complement Reverse CO9567: ACGCCCTTTTAAATATCCG

CCCGTGGGAAGGGCTGAGGCGCAGTTTCCTACTCACCCGGATCCGAATCCTCCGCGGTGCTGTTTCAAGAGAGCCGGATTCCAGATCGCGCTCCAGCCCGGACTCGGAATTCCTGCCCTGCGGGTCTGCATTTTCATAACGGGCAGGTGTGAGTGCCCTGCAGCTGGAGACCAGAAGCCTGAAGGCAGCTCGGCCCTCCCCAGCCCACAGCGCCGTTATTCCGTTTCTATATCAGTAAACACATTTCATTTTCCGTAGACCAGGGCGGGGTGACGGGTGATCCCAGTCCTCGCAGTGAATTCCGGGCAGCAAAATTCAAAACACATGCGGCCAAGGCCGGGCACGGTGGTTCACGCCTGTAATCCCAGCACTTTGGGAGGTCGAGGCGGGCGATCACCTGAGGTCGGGAGCTCGAGACCAACCTGACCAACATGGGGAAATCCCGTCTCTACTAAAAATATAAAATTAGACGGGCTTGGTGGTGAATGCCTGTAATCCCAGCTAGTCGGGAGGCTGAGGCAGGAGAATCGCTTAAACCTTGGAGGCGGAGGTTGCGGTGAGCCGAGATCGCGCCATTGCACTTCAGCCTGGGCAACAAGAGGGAAAACTCCGTCGCAAAAACTTTCGGGGGCGGAGCGGAGCCCCGCCCTGGGTTATGTAAGCGACCGCGCTGGGCCGTTTCTCTTTCTTTTCCGGACCCTGCAGTGGCGCCTAAAGTCTGAGAGAGGGAAGTCGCCTCTGTGCTCGTGAGTGCATGGGGTATAAGGCAAGTGCTGAGGGAGAAAACGTAGTTGATGGGGTAGAGCAGACGGGGTTGGAGGTGGGGTGGAGGGGGAGGGCTTTGGACAGAAGACCTGGGAGGCTTGGTGGGGGAGGGGCGCCCAGGCCTGGGCACTAAGAAACAAGTCCCCTGGAGCTCAAGACCATCTCGGCCTCCCCTAGCCCAAGAGAGGACTGGCTTCATGACTCCCTGAAACCATTTCTAAATGCCTTAGAACAAACCTTGCATATTCATTATTGTTATTGAACTATTAAAAGTCTTTTTTGGGGGCGAGCTGAATCAGATCCTTTGCTGGAGCTGGCACACGGAGGAAGTCCTGGAGGGAGGGTAGACACCGTGGAGGTAAGGGCTTGGGACCTGTGTCAGGTGAGACCAAGTCGTGAGAGGTACTGCACCACGAAGTGGTTTAAACTATCAGTGTTTGACAGGATATATTGGCGGGTA

**Assembled sequence**

Inserts 1 and 2

GGACGTTTTTATGTACTGGGGTGGATGCAGTGGGCCCCACTCTGTGGTGCAGTACCTCTCACGACTTGGTCTCACGTTGTGGGGCTTAGCTTCATATTTTCAAACTGAAATATTCTCTTCCTTAACCTCCACATAAATCCAAGTTTATAATTTTTATTATTTTAAAATTTTATTTATTTTTCTGTTTTGGGGACAGGGTCTCCTCCTGTCACTCAGGCTGGAGTGCAATGGCACAATCATAGCTCACTGCAGCCTGGAACTCCTGGGCTTAAGCAATCTTCCTGCCTTCGATTCCCAAAGAGCTGGGATTATAGTCATGAACCACTGCAATCCACCCAAATCCAAGTTTACACTAAAAGATAAAATTCCAACATTGTAGGGGATTGGTCAGGTGGTGGGAATAATTATAAAGATAAAGTTATAGGAAATAGACACAAACCTTCTTGGAAGGTGGAAAGTTTTGCAAAAGCCTCAGGATAGGGTTATAGCTGAAAGCAGCCTAATCCCCTTACCTTGAGTTAATAGCTTCGAGTAAGTACAAAGACATGTAAGAGAGTTTATCTAAAGAGCATGTTTACCTTTGATCATTTGTAGGACTGCTCTCTCTGGGGGACTGCGACCAGATTAATTACCCACAGGTGTGTTGACTCAAAGCCTTTGTCATTAAATCTGTGCTGAATAAAGGCCCACAGGGCCAGATAGTCAGGGCACGCAGCTGCCACAACCCTTTCTGTGAGTGGCCTGGCCCTCTGGTGCACTCTTTCACTGAATATCGGTGTCTGAGTACATTATTCATCCATCGTGCAGCCTGGGTCTGCCGGTCAGACCCTGGCACAACATTTAAGAGGAAATGAAAGTCACAAAGTTATCCCAGTCTCTGGAGTCACTGTCAAAACTTTGGTGAGGAATCTTCCAGGTTTTCCCCTACTTCAAATATATATTAATATTATGTAAGTGATATTAGTGGCATTTTTCGCCCAGGCTGGAATGCAGTGGCATGATCTCGGCTCACTCTAACCTCTACCTCCCAGATTCAAGCGATTCTCCTGCCTCAGCCTCCCAAACAGCTGGAACTACAGGCACCCACCACCACGCCCGGCTAATTTTTGCATTTTC

Inserts 2, 3 and 4

GGCTCACGCCTGTAATCCCAGCACTTTGAGAGGCCAAGGCAGGCAGATCACGAGGTCAGGAGATCGAGACCATCCTGGCTAACACAGTGAAACCCCGTCTCTACTAAAAATACAAAAAATTAGTCGGGCGTGGTGGCAGGAGCCTGTAGTCCCAGCTACTCCAGAGGCTGAGGCAGCAGAATGCCCTGAACCCGGGAGGCGGAGCTTGCAGTGAGCCAAGATCGCGCCACTGCACTCCAGCCTGGGCGACAGAGCAAGACTCCGTCTCAAAAAAAAAAAACAAAAAACAAAAAAACTCTCCTTTACTTTTTCTCTCCCCTTTTCTTCCTATCTCTTCCCTCATTTCTTCAACACGTCCCCCCATCCTTCCCTCTTTTCTCCATTCTCTGCATTTGATCCCCGGTATATTCCAGCCTCCAGGCCAACAAACTTCTCCGCGTCCGCCGGGAGCAGGTCAGGGAAGGGACGCGAGGCGGCGCTGTCACCGCATTCTGAGCGCCGCAGCTCCCTGGGCCCCTTGTATCATTTCAGTGAAGGTCACTCCAGTCTTTCATGGAGGCCAAACTAAGGGTGTAAATTAGGATCCTCACTGAAGTGGCGGGACCCTAAGAGGCTTTTTCCTGGCCCCTTAGTTGTGGGTTTTCCTGCGGGCGGCGCAGCCGGTTTCCATCAGAACCGCCCAGAGGCGGACGCTGCCTTCCTGGGGTGACGGAGCAGCAGGAAGCGTTTTCGGATCCTGGAATACGTGGGCGGCCCGTGGNAGGGGCTGAGGCGCAGTTTCCTACTCACCCGGATCCGAATCCTCCGCGGNGCTGTTTCAAGAGAGCCGGATTCCAGATCGCGCTCCAGCCCGGACTCGGAATTCCTGCCCTGCGGGTCTGCATTTTCATAACGGNCAGGTGTGAGTGCCCTGCAGCTGGAGACCAGAAGCCTGAAGGCAGCTCGGACCTCCCAGNCACAGCGCCGTTATTCCGTTTCTATATCAGTAAACACATTTCATTTTCCGTAGACCAGGGCGGGGTGACGGGTGATCCCAGTCCTCGCAGTGAATTCCGGGCAGCAAAATTCAAAACACATGCGGCCAAGGCCGGGCACGGTGGTTCACGCCTGTAATCCCAGCACTTTGGGAGGTCGAGGCGGGCGATCACCTGAGGTCGGGAGCTCGAGACCAACCTGACCAACATGGGGAAATCCCGTCTCTACTAAAAATATAAAATTAGACGGGCTTGGTGGTGAATGCCTGTAATCCCAGCTAGTCGGGAGGCTGAGGCAGGAGAATCGCTTAAACCTTGGAGGCGGAGGTTGCGGTGAGCCGAGATCGCGCCATTGCACTTCAGCCTGGGCAACAAGAGGGAAAACTCCGTCGCAAAAACTTTCGGGGGCGGAGCGGAGCCCCGCCCTGGGTTATGTAAGCGACCGCGCTGGGCCGTTTCTCTTTCTTTTCCGGACCCTGCAGTGGCGCCTAAAGTCTGAGAGAGGGAAGTCGCCTCTGTGCTCGTGAGTGCATGGGGTATAAGGCAAGTGCTGAGGGAGAAAACGTAGTTGATGGGGTAGAGCAGACGGGGTTGGAGGTGGGGTGGAGGGGGAGGGCTTTGGACAGAAGACCTGGGAGGCTTGGTGGGGGAGGGGCGCCCAGGCCTGGGCACTAAGAAACAAGTCCCCTGGAGCTCAAGACCATCTCGGCCTCCCCTAGCCCAAGAGAGGACTGGCTTCATGACTCCCTGAAACCATTTCTAAATGCCTTAGAACAAACCTTGCATATTCATTATTGTTATTGAACTATTAAAAGTCTTTTTTGGGGGCGAGCTGAATCAGATCCTTTGCTGGAGCTGGCACACGGAGGAAGTCCTGGAGGGAGGGTAGACACCGTGGAGGTAAGGGCTTGGGACCTGTGTCAGGTGAGACCAAGTCGTGAGAGGTACTGCACCACGAAGTGGTTTAAACTATCAGTGTTTGACAGGATATATTGGCGGGTA
