# Supplementary material for: UniClo: scarless hierarchical DNA assembly without sequence constraint
Source: Nucleic Acids Res. 2025 Jun 23;53(12):gkaf548. doi: 10.1093/nar/gkaf548 (PMC12205989; doi:10.1093/nar/gkaf548)
Supplement: gkaf548_Supplemental_Files [file gkaf548_supplemental_files.zip › Supplementary revised.pdf]

Supplementary Information

UniClo: Scarless hierarchical DNA assembly without sequence constraint

Carol N. Flores-Fernández[1], Da Lin[1†], Katherine Robins[1‡], Chris A. O'Callaghan[1]*

[1]Centre for Human Genetics, Nuffield Department of Medicine, University of Oxford, Roosevelt Drive, Oxford OX3 7BN, UK

[†]Current address: Triple Helix Biotechnology Ltd, Moneta Building (B280), Babraham Research Campus, Babraham, CB22 3AT, UK

[‡]Current address: Complete Regulatory, 19-20 King Edward Street, Macclesfield, SK10 1AQ, UK

*Corresponding author

E-mail: chris.ocallaghan@ndm.ox.ac.uk

Table of contents

Supplementary Methods 2
Calculation of transformation efficiency 2
Recombinant methylases production and purification 2
List of Supplementary Tables and Figures 3
References 38

Supplementary Methods

Calculation of transformation efficiency

The transformation efficiency (cfu/µg) of the assemblies was calculated using Equation S1.

$$Transformation\ efficiency\ (\frac{cfu}{\mu g}) = \frac{Number\ of\ transformants}{\mu g\ of\ DNA}\ x\ \frac{final\ volume\ at\ recovery\ (mL)}{volume\ plated\ (mL)} \qquad \text{Equation S1}$$

Recombinant methylases production and purification

The plasmids POC1471, POC1464, POC1466 and POC1468 encoding the recombinant methylases are available from Addgene, and the Addgene plasmid identification codes are provided in Table S2. Full details of the production and application of these enzymes have been published previously (1). For methylases production, these plasmids were transformed into the expression host *E. coli* BL21(DE3)pLysS (Merck KGaA, Novagen). The cells harbouring the recombinant plasmids were grown in 10 mL of LB broth containing either 0.1 g/l ampicillin or 0.05 g/l kanamycin with 0.05 g/l chloramphenicol and incubated at 37 °C overnight. The overnight starter cultures were used to inoculate 150 ml of the same medium without chloramphenicol. Cultivation was carried out in 1 l baffled flasks at 37 °C and 230 rpm in a shaker incubator (New Brunswick™ Innova® 44/44R Incubator Shakers, Eppendorf, Stevenage UK) until the absorbance at 600 nm reached 0.45. At this point, the cell expression was induced by adding 0.5 mM isopropyl β-D-1-thiogalactopyranoside (IPTG) and incubating at 20 °C and 230 rpm for up to 24 h. The cells were harvested by centrifugation (4,000×g at 4 °C for 20 min) and stored at -20 °C until purification. For methylase purification, the cell pellets from 100 ml cultures were re-suspended in 2.5 ml of 50 mM sodium phosphate buffer containing 300 mM NaCl, 10% glycerol and 10 mM imidazole, pH 7.5. The resuspended cells were disrupted by sonication (Ultrasonic processors, Vibra-Cell™ VCX 500) with 20 cycles of 10 s ON and 50 s OFF at 35% amplitude. Subsequently, the cell suspension was centrifuged (17,200×g at 4 °C for 30 min) and the clarified cell lysates were recovered. The methylases were purified from the clarified lysate by gravity-flow affinity chromatography using a His-Tag Ni-affinity resin (Ni-NTA Agarose, ThermoFisher, Loughborough UK). Buffers containing 10, 50, and 400 mM imidazole were used as binding/equilibration, washing, and elution buffers respectively. These buffers were prepared in 50 mM sodium phosphate buffer containing 300 mM NaCl and 10% glycerol, pH 7.5. The purified methylases were desalted using PD-10 Desalting Columns (Cytiva), protein concentration was determined using Pierce™ BCA Protein Assay Kit (ThermoFisher), and they were analysed by SDS-PAGE (1). Finally, the desalted purified methylases were stored at -80 °C until the DNA assembly assays.

List of Supplementary Tables and Figures

Supplementary Tables

Table S1	Chemicals, plasmids and services.
Table S2	Details of plasmids used for DNA assembly and plasmids encoding the recombinant methylases.
Table S3	Recombinant methylases.
Table S4	Reaction conditions used for the site-selective DNA methylation-protection of different targets and controls.
Table S5	Assembly vectors, donor plasmids and resulting assembled plasmids.
Table S6	Transformation efficiency and success rate data of the assemblies from this study.
Table S7	Scarless hierarchical assembly of DNA fragments containing internal BsaI sites using UniClo.
Table S8	DNA assembly techniques and their features in comparison with UniClo.

Supplementary Figures

Figure S1	Schematic overview of MetClo.
Figure S2	MetClo assembly vector design.
Figure S3	Assembly vector POC1355 designed to be methylated by the M.Osp807II switch methylase.
Figure S4	Donor plasmids POC1343, POC1344, POC1345 and POC1346.
Figure S5	Sequences of fragments in donor plasmids POC1343, POC1344, POC1345 and POC1346.
Figure S6	Assembled plasmid POC1358.
Figure S7	Agarose gel electrophoresis of POC1358.
Figure S8	Design of plasmid POC1423 to test methylation protection.
Figure S9	Polyacrylamide gel electrophoresis of sgRNA.
Figure S10	Agarose gel electrophoresis (1%) showing the site-selective methylation-protection of POC1423 and related controls.
Figure S11	Donor plasmids POC1426, POC1427, POC1428 and POC1429.
Figure S12	Methylation protection of POC1426, POC1427, POC1428 and POC1429.
Figure S13	Design of assembly vector POC1430 to test DNA assembly using methylation-protection.
Figure S14	Sequences of fragments in donor plasmids POC1426, POC1427, POC1428 and POC1429.
Figure S15	Assembled plasmid POC1431.
Figure S16	Restriction sites in the assembled plasmid POC1431.
Figure S17	Agarose gel electrophoresis of POC1431 assembled after methylation using M2.Eco31I.
Figure S18	Agarose gel electrophoresis of POC1431 assembled after methylation using M2.Eco31I_2.
Figure S19	Agarose gel electrophoresis of POC1431 assembled after methylation using M2.BsaI.
Figure S20	VL, VM and VR assembly vectors for scarless DNA assembly.
Figure S21	Scarless hierarchical assembly scheme for 11 fragments using UniClo.
Figure S22	Assembled plasmids from the first round of scarless hierarchical assembly using UniClo.
Figure S23	Agarose gel electrophoresis of the assembled plasmids from the first round of the hierarchical assembly using UniClo.
Figure S24	Assembled plasmid POC1550 from the second round of the hierarchical assembly using UniClo.
Figure S25	Agarose gel electrophoresis of the assembled plasmid POC1550 from the second round of the hierarchical assembly using UniClo.

Table S1. Chemicals, plasmids and services.

Chemicals/plasmids/services	Manufacturer/ Supplier	Code	Notes
Q5® High-Fidelity 2X Master Mix	NEB	M0492S	-20 °C
Monarch® Spin Plasmid Miniprep Kit	NEB	T1010S	RT
Quick-Load(R) Purple 1 kb Plus DNA Ladder	NEB	N0552S	-20 °C
NEB® 10-beta Competent *E. coli*	NEB	C3019I	-20 °C
NEB® 10-beta Electrocompetent *E. coli*	NEB	C3020K	-20 °C
EnGen® Spy dCas9	NEB	M0652T	-20 °C
SAM	NEB	B9003S	-20 °C
T4 DNA Ligase	NEB	M0202S	-20 °C
BsaI-HF®v2	NEB	R3733S	-20 °C
BamHI	NEB	R3189S	-20 °C
DraIII-HF	NEB	R3510S	-20 °C
NotI-HF	NEB	R3189S	-20 °C
QIAquick PCR Purification Kit	Qiagen	28104	RT
QIAprep® Spin Miniprep Kit	Qiagen	27106	RT
UltraPure™ DNase/RNase-Free Distilled Water	ThermoFisher	10977035	RT
GeneArtTM Precision gRNA Synthesis Kit	ThermoFisher	A29377	-20 °C
Kanamycin Sulfate	ThermoFisher	11815024	RT
IPTG	ThermoFisher	R0392	4 °C
X-Gal	ThermoFisher	R0404	-20 °C
Genomic DNA Clean & ConcentratorTM Kit-25	Cambridge Bioscience	D4064	RT
Tris-HCl	Merck KGaA	93363	RT
EDTA	Merck KGaA	E9884	RT
DTT	Merck KGaA	D9779	RT
Ampicillin sodium crystalline	Merck KGaA	A9518-25G	4 °C
MCE 0.05U WH PL 25MM 100PK	Merck KGaA	VMWP02500	RT
Chloramphenicol	Merck KGaA	C0378	RT
Primers	Merck KGaA	-	Sequences in the manuscript
Plasmids for the assembly	Addgene	-	IDs in Table S2
Methylases plasmids	Addgene	-	IDs in Table S2
DNA synthesis	GenScript	-	https://www.genscript.com
DNA sequencing	Source BioScience	-	https://sourcebioscience.com

SAM, S-adenosylmethionine; IPTG, isopropyl β-D-1-thiogalactopyranoside; X-Gal, 5-bromo-4-chloro-3-indolyl-beta-D-galacto-pyranoside; EDTA, ethylenediaminetetraacetic acid; DTT, dithiothreitol; NEB, New England Biolabs; and RT, room temperature.

Table S2. Details of plasmids used for DNA assembly and plasmids encoding the recombinant methylases.

Laboratory code[a]	Addgene code[b]	Description	Selection	Purpose
**Plasmids used for DNA assembly**				
POC1343	221568	Donor plasmid containing Fragment 1 for assembly of ~1 kb DNA	Kanamycin	DNA assembly using recombinant switch methylases.
POC1344	221569	Donor plasmid containing Fragment 2 for assembly of ~1 kb DNA	Kanamycin	
POC1345	221570	Donor plasmid containing Fragment 3 for assembly of ~1 kb DNA	Kanamycin	
POC1346	221571	Donor plasmid containing Fragment 4 for assembly of ~1 kb DNA	Kanamycin	
POC1355	221572	Assembly vector for assembly of ~1 kb DNA	Ampicillin	
POC1358	236103	Assembled plasmid containing ~1 kb DNA	Ampicillin	
POC1423	221559	Plasmid containing a methylation-protectable and an always-methylatable BsaI site	Ampicillin	*In vitro* methylation-protection.
POC1426	221573	Donor plasmid containing Fragment 1 for assembly of ~3.6 kb DNA	Kanamycin	DNA assembly of fragments with internal type IIS restriction sites using the recombinant methylases and methylation-protection.
POC1427	221574	Donor plasmid containing Fragment 2 for assembly of ~3.6 kb DNA	Kanamycin	
POC1428	221575	Donor plasmid containing Fragment 3 for assembly of ~3.6 kb DNA	Kanamycin	
POC1429	221576	Donor plasmid containing Fragment 4 for assembly of ~3.6 kb DNA	Kanamycin	
POC1430	221577	Assembly vector for assembly of ~ 3.6 kb DNA	Ampicillin	
POC1431	221578	Assembled plasmid containing ~3.6 kb DNA	Ampicillin	
POC1518	221579	VL: Assembly vector for assembly of left-end fragments in 10.8 kb assembled fragment	Kanamycin	Hierarchical DNA assembly of fragments with internal type IIS restriction sites using the assembly vectors for scarless type IIS enzyme-based assembly, the recombinant methylases and methylation-protection.
POC1519	221580	VM: Assembly vector for assembly of 'middle' fragments in 10.8 kb assembled fragment	Kanamycin	
POC1520	221581	VR: Assembly vector for assembly of right-end fragments in 10.8 kb assembled fragment	Kanamycin	
POC1525	226496	VM: Assembly vector for assembly of the final 10.8 kb assembled fragment	Chloramphenicol	
POC1535	226476	VL_1_1: Donor plasmid containing Fragment 1_1 for assembly of 10.8 kb DNA	Chloramphenicol	
POC1536	226477	VM_1_2: Donor plasmid containing Fragment 1_2 for assembly of 10.8 kb DNA	Chloramphenicol	
POC1537	226478	VM 1_3: Donor plasmid containing Fragment 1_3 for assembly of 10.8 kb DNA	Chloramphenicol	
POC1538	226479	VR 1_4: Donor plasmid containing Fragment 1_4 for assembly of 10.8 kb DNA	Chloramphenicol	
POC1539	226480	VL 1_5: Donor plasmid containing Fragment 1_5 for assembly of 10.8 kb DNA	Chloramphenicol	
POC1540	226481	VM 1_6: Donor plasmid containing Fragment 1_6 for assembly of 10.8 kb DNA	Chloramphenicol	
POC1541	226482	VR 1_7: Donor plasmid containing Fragment 1_7 for assembly of 10.8 kb DNA	Chloramphenicol	
POC1542	226483	VL 1_8: Donor plasmid containing Fragment 1_8 for assembly of 10.8 kb DNA	Chloramphenicol	
POC1543	226484	VR 1_9: Donor plasmid containing Fragment 1_9 for assembly of 10.8 kb DNA	Chloramphenicol	
POC1544	226485	VL 1_10: Donor plasmid containing Fragment 1_10 for assembly of 10.8 kb DNA	Chloramphenicol	
POC1545	226486	VR 1_11: Donor plasmid containing Fragment 1_11 for assembly of 10.8 kb DNA	Chloramphenicol	

POC1546	226487	VL_2_1: Assembled plasmid from the first round of assembly containing 1_1-1_4	Kanamycin	
POC1547	226489	VM_2_2: Assembled plasmid from the first round of assembly containing 1_5-1_7	Kanamycin	
POC1548	226490	VM_2_3: Assembled plasmid from the first round of assembly containing 1_8-1_9	Kanamycin	
POC1549	226491	VR_2_4: Assembled plasmid from the first round of assembly containing 1_10-1_11	Kanamycin	
POC1550	226497	VM_3_1: Assembled plasmid from the second round of assembly containing 1_1-1_14	Chloramphenicol	
POC1553	238470	VL: Assembly vector for assembly of left-end fragments	Chloramphenicol	Further rounds of a hierarchical scarless DNA assembly using methylation-protection.
POC1554	238471	VR: Assembly vector for assembly of right-end fragments	Chloramphenicol	

The key VL, VM and VR assembly vector plasmids are, respectively, POC1518, POC1519 and POC1520 with kanamycin selection and POC1553, POC1525 and POC1554 with chloramphenicol selection.

Plasmids encoding recombinant methylases

POC1471	213759	Switch methylase M.Osp807II	Ampicillin	Methylation of methylation-switchable sites in assembly vectors.
POC1464	213736	Non-switchable methylase M2.Eco31I	Kanamycin	Methylation of always-methylatable (internal sites) of fragments in donor plasmids.
POC1466	213754	Non-switchable methylase M2.Eco31I_2	Kanamycin	
POC1468	213756	Non-switchable methylase M2.BsaI	Kanamycin	

[b]All the plasmids described in this table are available from Addgene using their corresponding Addgene code.

Table S3. Recombinant methylases.

Methylase	Type	Plasmid Code	Methylase recognition sequence[b] and methylated base[c]	Overlap with BsaI site	Methylated product[d]
M.Osp807II	Switch	POC1471	5'… G**A**CNNNGTC … 3'<br>3'… CTGNNNC**A**G … 5'	Partial	5'… G**A**CNNGGTCTC(N)$_1$▼ … 3'<br>3'… CTGNNCC**A**GAG(N)$_5$▲ … 5'
M2.Eco31I<br>M2.Eco31I_2[a]	Non-switchable<br>Non-switchable	POC1464<br>POC1466	5'… GGT**C**TC … 3'<br>3'… CCAGAG… 5'	Complete	5'… GGT**C**TC(N)$_1$▼… 3'<br>3'… CCAGAG(N)$_5$▲ … 5'
M2.BsaI	Non-switchable	POC1468	5'… GGTCTC … 3'<br>3'… CCAGAG … 5'<br>5-methylcytosine top strand (base undetermined)	Complete	5'… GGTCTC(N)$_1$▼… 3'<br>3'… CCAGAG(N)$_5$▲ … 5'<br>5-methylcytosine top strand (base undetermined)

[a]M2.Eco31I_2 is a truncated version of M2.Eco31I with the second ATG used as the start codon, [b]the methylase recognition sequence is in red, [c]the methylated base is in bold and underlined, [d]the methylated product shows the methylase recognition sequence and the methylated base as well as the BsaI endonuclease recognition sequence (GGTCTC, green) and its restriction site is marked with ▼▲.

Table S4. Reaction conditions used for the site-selective DNA methylation-protection of different targets and controls.

Components	Target DNA					Controls[h]			
	[a]POC1423	[b]POC1426	[c]POC1427	[d]POC1428	[e]POC1429	POC1423			
NF water (µl)	12.6	11.3	10.5	13.4	13.2	13.1	16.5	17.0	15.8
10X NEB Buffer r3.1 (µl)	3.0	3.0	3.0	3.0	3.0	3.0	3.0	3.0	3.0
sgRNA[f] (µl)	3.9	3.9	3.9	3.9	3.9	3.9	-	-	3.9
dCas9 (µl)	0.5	0.5	0.5	0.5	0.5	-	0.5	-	0.5
Partial volume 1 (µl)	20.0	18.7	17.9	20.8	20.6	20.0	20.0	20.0	23.2
Incubate at 25 °C x 10 min									
Target DNA (µl)	5.8	5.3	6.1	6.8	7.0	5.8	5.8	5.8	5.8
Incubate at 37 °C x 15 min									
3200 µM SAM (µl)	1.0	1.0	1.0	1.0	1.0	1.0	1.0	1.0	1.0
Non-switchable methylase[g]	3.2	5.0	5.0	1.4	1.4	3.2	3.2	3.2	-
Partial volume 2 (µl)	10.0	11.3	12.1	9.2	9.4	10.0	10.0	10.0	6.8
Final volume (µl)	30.0	30.0	30.0	30.0	30.0	30.0	30.0	30.0	30.0
Incubate at 37 °C x 15 min									
Incubate at 80 °C x 20 min									
Purify using the QIAquick PCR Purification Kit									

NF, nuclease-free; plasmid DNA concentration (ng/µl): [a]431, [b]470.6, [c]409.1, [d]365.2 and [e]354.7. Each plasmid is around 5 kb, and the DNA amount required for 811 fmol is around 2500 ng. [f]sgRNA concentration (ng/µl): 80 ng/µl. The sgRNA is 100 nt, and the amount required for 8110 fmol is around 313 ng. [g]The recombinant non-switchable methylases used were M2.Eco31I_2 for POC1423, M2.Eco31I for POC1426 and POC1427, and M2.BsaI for POC1428 and POC1429. The concentrations of the methylases M2.Eco31I_2, M2.Eco31I and M2.BsaI were 0.15, 0.10, and 0.36 µg/µl respectively; and 500 ng of each methylase were used in the reactions. In these reactions, the target DNA and methylases concentrations vary; therefore, the volume of water can be adjusted to achieve a final volume of 30 µl. [h]Controls were tested using POC1423 without the addition of dCas9, sgRNA, or both dCas9 and sgRNA.

Table S5. Assembly vectors, donor plasmids and resulting assembled plasmids.

Assembly vector	Donor plasmids				Assembled plasmid	Size of assembled fragment
POC1355	POC1343	POC1344	POC1345	POC1346	POC1358	1.0 kb
POC1430	POC1426	POC1427	POC1428	POC1429	POC1431	3.6 kb
POC1518 (VL)	POC1535 (VL_1_1)	POC1536 (VM_1_2)	POC1537 (VM_1_3)	POC1538 (VR_1_4)	POC1546 (VL_2_1)	3.5 kb
POC1519 (VM)	POC1539 (VL_1_5)	POC1540 (VM_1_6)	POC1541 (VR_1_7)		POC1547 (VM_2_2)	3.5 kb
POC1519 (VM)	POC1542 (VL_1_8)	POC1543 (VR_1_9)			POC1548 (VM_2_3)	1.5 kb
POC1520 (VR)	POC1544 (VL_1_10)	POC1545 (VR_1_11)			POC1549 (VR_2_4)	2.3 kb
POC1525 (VM)	POC1546 (VL_2_1)	POC1547 (VM_2_2)	POC1548 (VM_2_3)	POC1549 (VR_2_4)	POC1550 (VM_3_1)	10.8 kb

Table S6. Transformation efficiency and success rate data of the assemblies from this study.

Assembled plasmid	Size of assembled fragment	Number of transformants (cfu)	µg of DNA	Final volume at recovery (mL)	Volume plated (mL)	Transformation efficiency[a] (cfu/µg)	Success rate[b] (%)
POC1358	1.0 kb	200	2.5	0.9	0.05	1.4×10^3	100 (10/10)
POC1431	3.6 kb	341	1.0	1.0	0.10	3.4×10^3	100 (5/5)
POC1546 (VL_2_1)	3.5 kb	149	0.7	1.0	0.10	2.1×10^3	100 (5/5)
POC1547 (VM_2_2)	3.5 kb	40	0.6	1.0	0.10	0.7×10^3	100 (5/5)
POC1548 (VM_2_3)	1.5 kb	231	0.5	1.0	0.10	4.6×10^3	100 (5/5)
POC1549 (VR_2_4)	2.3 kb	90	0.5	1.0	0.10	1.8×10^3	100 (5/5)
POC1550 (VM_3_1)	10.8 kb	120	0.8	1.0	0.10	1.5×10^3	83 (5/6)

[a]Calculations were carried out using Equation S1. [b]The numbers in parentheses indicate the number of correct white colonies / the total number of white colonies analysed. The assembled fragment from the correct colonies was confirmed by sequencing.

Table S7. Scarless hierarchical assembly of DNA fragments containing internal BsaI sites using UniClo.

Details of a type IIS enzyme-based assembly of DNA fragments containing multiple internal BsaI sites using UniClo. Recombinant M.Osp807II switch methylase and M2.BsaI non-switchable methylase were used with methylation-protection.

First round													
							Second Round						
**Fragment**		**Donor plasmid (Chl^R)**			**Assembly vector (Kan^R)**		**Assembled plasmid (New donor plasmid) (Kan^R)**		**Assembly vector (Chl^R)**		**Final assembled plasmid (Chl^R)**	
	**Size (bp)**	**Type**	**Code**	**Size (bp)**	**Type**	**Size (bp)**	**Code**	**Size of assembled fragment (kb)**	**Type**	**Size (bp)**	**Code**	**Size of assembled fragment (kb)**
1_1	776	VL	POC1535 (VL_1_1)	2600	POC1518 (VL)	8023	POC1546 (VL_2_1)	3.5	VM	7994	POC1550 (VM_3_1)	10.8
1_2	957	VM	POC1536 (VM_1_2)	2777								
1_3	890	VM	POC1537 (VM_1_3)	2710								
1_4	861	VR	POC1538 (VR_1_4)	2687								
1_5	1095	VL	POC1539 (VL_1_5)	2919	POC1519 (VM)	8019	POC1547 (VM_2_2)	3.5				
1_6	1218	VM	POC1540 (VM_1_6)	3038								
1_7	1266	VR	POC1541 (VR_1_7)	3092								
1_8	711	VL	POC1542 (VL_1_8)	2535	POC1519 (VM)	8019	POC1548 (VM_2_3)	1.5				
1_9	785	VR	POC1543 (VR_1_9)	2611								
1_10	1138	VL	POC1544 (VL_1_10)	2962	POC1520 (VR)	8025	POC1549 (VR_2_4)	2.3				
1_11	1118	VR	POC1545 (VR_1_11)	2940								

VL, Vector Left; VM, Vector Middle; and VR, Vector Right. Chl^R, chloramphenicol resistance; and Kan^R, kanamycin resistance. This scheme was used to assemble 11 DNA fragments to generate a final assembled fragment of 10.8 kb.

Table S8. DNA assembly techniques and their features in comparison with UniClo.

DNA assembly technique	Assembly vectors	Fragments to be assembled	Joining adaptors	Restriction enzymes	Scars in the final assembled fragment	Use of methylases	Assembly of fragments with internal restriction sites	Methylation reaction	Reported assembled fragment size	Notes	Ref.
PSA	Two sets of multiple vectors with dual antibiotic markers	Synthesised linear DNA	Multiple	Three type IIS	Yes, due to the joining adaptors	Yes, to methylate fragments	Yes, using a methylase and oligos for site-selective protection of methylation	*In vitro*	91 kb	Assembly of pairs of fragments only	(2)
MASTER	Multiple	PCR-amplified short linear DNA with methylation in flanking sites	Multiple	One modification-dependent endonuclease	Yes, in the flanking sites	No, but it uses DNA methylation	Yes, using a modification-dependent endonuclease that cuts only methylated DNA	*In vitro*	29 kb	The modification-dependent endonuclease only cuts the methylated flanking sites	(3)
2ab assembly	-	In multiple lefty and righty donor plasmids with dual antibiotic markers	Multiple	Three type IIP	Yes, due to the type IIP restriction sites	Yes, to site-specific methylation of plasmids	No	*In vivo*	528 plasmids encoding bi-cistronic operons	The 'lefty' and 'righty' donor plasmid parts form the assembled plasmid	(4)
MoClo	Multiple	In multiple donor plasmids	Multiple	Three type IIS	Yes, due to the joining adaptors	No	No, domestication is carried out	-	33 kb	Requires multiple assembly vectors	(5)
SSEA	One with multiple type IIP restriction sites	PCR-amplified linear DNA	Multiple	Multiple type IIP depending on the sequence	Yes, small scars on the flanking sites	No	No	-	12 kb	The final assembled fragment can not be released from the assembled plasmid	(6)
TNT	Multiple	In multiple donor plasmids	Multiple signatures	Two type IIS	Yes, due to the signatures	Yes, to site-selective protection of digestion in assembly vectors	Yes, using oligos for site-selective protection of restriction	*In vivo*	12 kb	Requires multiple assembly vectors and signatures and leaves unwanted scars	(7)

MetClo	Multiple	In multiple donor plasmids	Multiple compatible	One type IIS	Yes, small scars on the flanking sites	Yes, to methylate assembly vectors	No	*In vivo*	218 kb	One-pot assembly reaction	(8)
PS-Brick	Three with IIP and IIS restriction sites	PCR-amplified linear DNA	Two	One type IIP and two type IIS	Yes, small scars due to IIP restriction sites	No	No	-	Propanol pathway formed by *L. lactis kivD* and *S. cerevisiae ADH2*	Assemblies in metabolic engineering	(9)
Start-Stop	Multiple	PCR-amplified or synthesised linear DNA or in donor plasmids	Multiple and conserved sequence motifs	Restricted to one specific type IIS	Yes, scarless only for CDSs in expression units	No	No	-	Carotenoid pathways	Assemblies in metabolic engineering	(10)
GGA-DAD	None or one	PCR-amplified linear DNA	Multiple compatible	One type IIS for each step	Yes, small scars on the flanking sites	No	No, domestication is carried out	-	40 kb	One-pot assembly reaction	(11)
GG-type BioBrick	Multiple	Multiple	Multiple	Multiple type IIP	Yes, due to type IIP restriction sites	Yes, to site-specific methylation in acceptor and donor plasmids	No	*In vivo*	-	Several undesired products	(12)
PlasmidMaker	-	PCR-amplified linear DNA	Multiple	One argonaute protein	No, due to homology sharing between fragments	No	Yes, argonaute protein cuts only at specific sites required for the assembly	-	18 kb	Requires major infrastructure, automation equipment and software. Complex custom setup.	(13)
UniClo	Two sets of three scarless vectors	Synthesised DNA in donor plasmids	Two universals	One type IIS	No, fully scarless	Yes, to methylate assembly vectors and donor plasmids	Yes, using methylases and CRISPR/dCas9 for site-selective methylation-protection	*In vitro*	10.8 kb	Assembly of any DNA sequence using one guide RNA	This study

PSA, Pairwise Selection Assembly; MASTER, Methylation-Assisted Tailorable Ends Rational; MoClo, Modular Cloning; SSEA, Seamless Stack Enzymatic Assembly; TNT, Three Nucleotides; MetClo, Methylase-assisted Cloning; GGA-DAD, Golden Gate with Data-optimized Assembly Design; and GG, Golden Gate

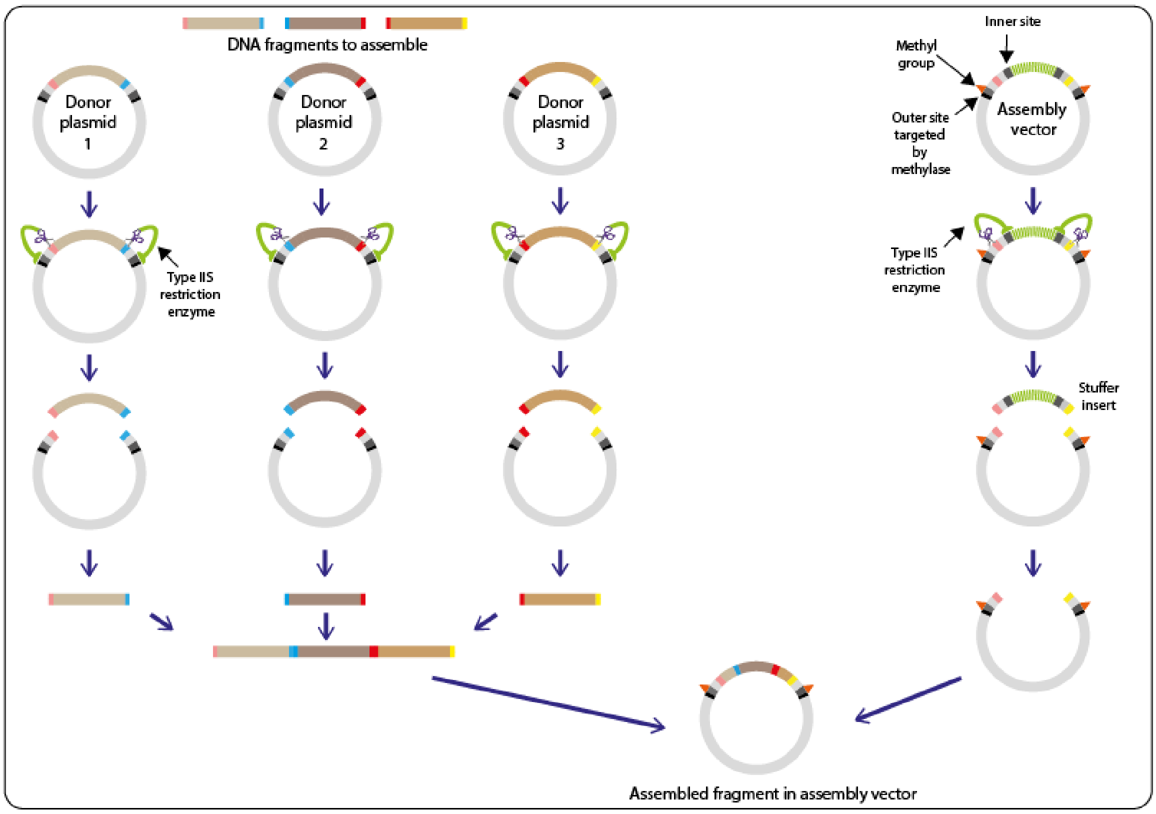

Figure S1. Schematic overview of MetClo. MetClo requires only a single type IIS restriction enzyme. The assembly vector has two outer sites for this restriction enzyme sites which direct cutting inwards and two inner sites which direct cutting outwards from a stuffer insert which contains a lacZ marker. Prior to the assembly a switch methylase is used to methylate the outer sites which renders them inactive as recognition sites for the type IIS restriction enzyme. During the reaction the inner sites which are not methylated are cut outwards by the type IIS restriction enzyme and the stuffer insert is released. The DNA fragments to be assembled are cloned into donor plasmids which have unmethylated recognition site for the type IIS restriction enzyme flanking the inserted fragments. During the assembly reaction the type IIS enzyme cuts inwards from these sites releasing the fragments. The fragments and the cut assembly vector have cohesive overhangs (shown as pink, blue, red and yellow) that dictate the correct order and orientation of assembly.

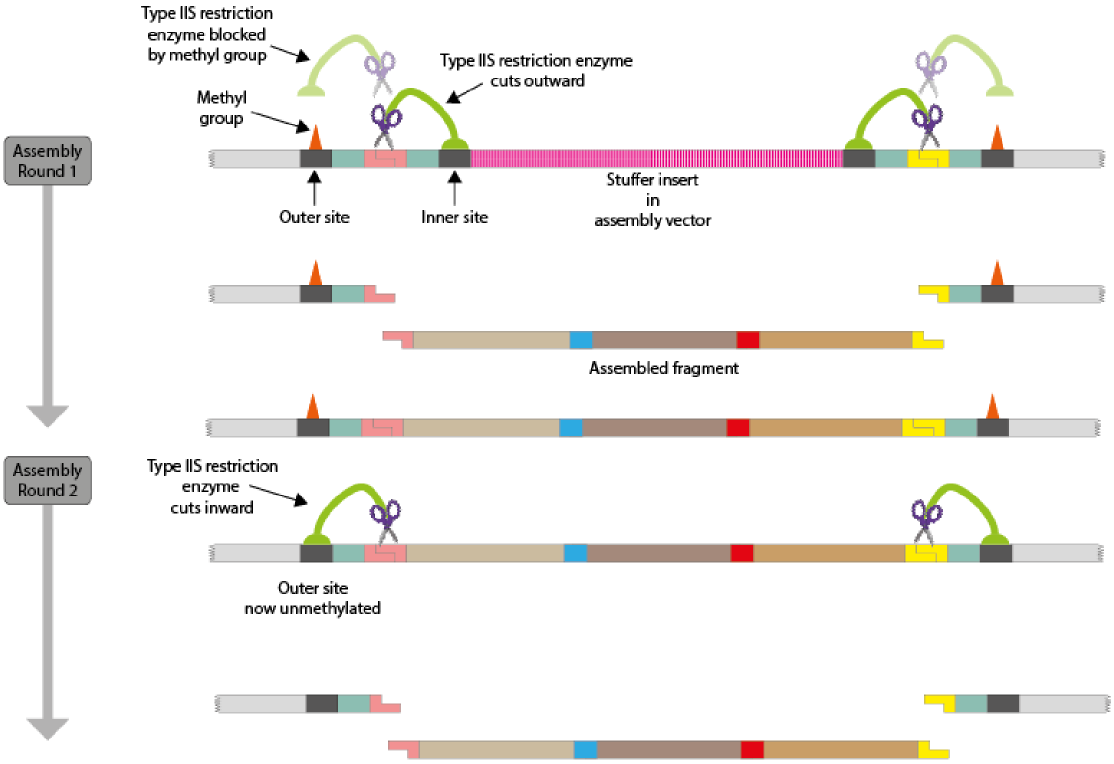

Figure S2. MetClo assembly vector design. A schematic view is shown of the design of the type IIS restriction enzyme sites around the insert region in a MetClo assembly vector. The outer sites are designed to be targets for methylation by a switch methylase and prior to the first round of the assembly these sites are methylated which inhibits the activity of the type IIS restriction enzyme at these sites. These outer sites are termed 'methylation-switchable'. During the reaction the inner sites which are not methylated are cut outwards by the type IIS restriction enzyme and the stuffer insert is released. During the assembly reaction, cohesive overhangs (pink and yellow) on the assembled fragment interact with compatible overhangs on the assembly vector. The plasmid is then propagated in a strain which does not express the switch methylase and this leaves the outer sites unmethylated. In the next round of the assembly, the type IIS restriction enzyme cuts inwards from these outer sites to release the assembled fragment for assembly with other fragments in that round.

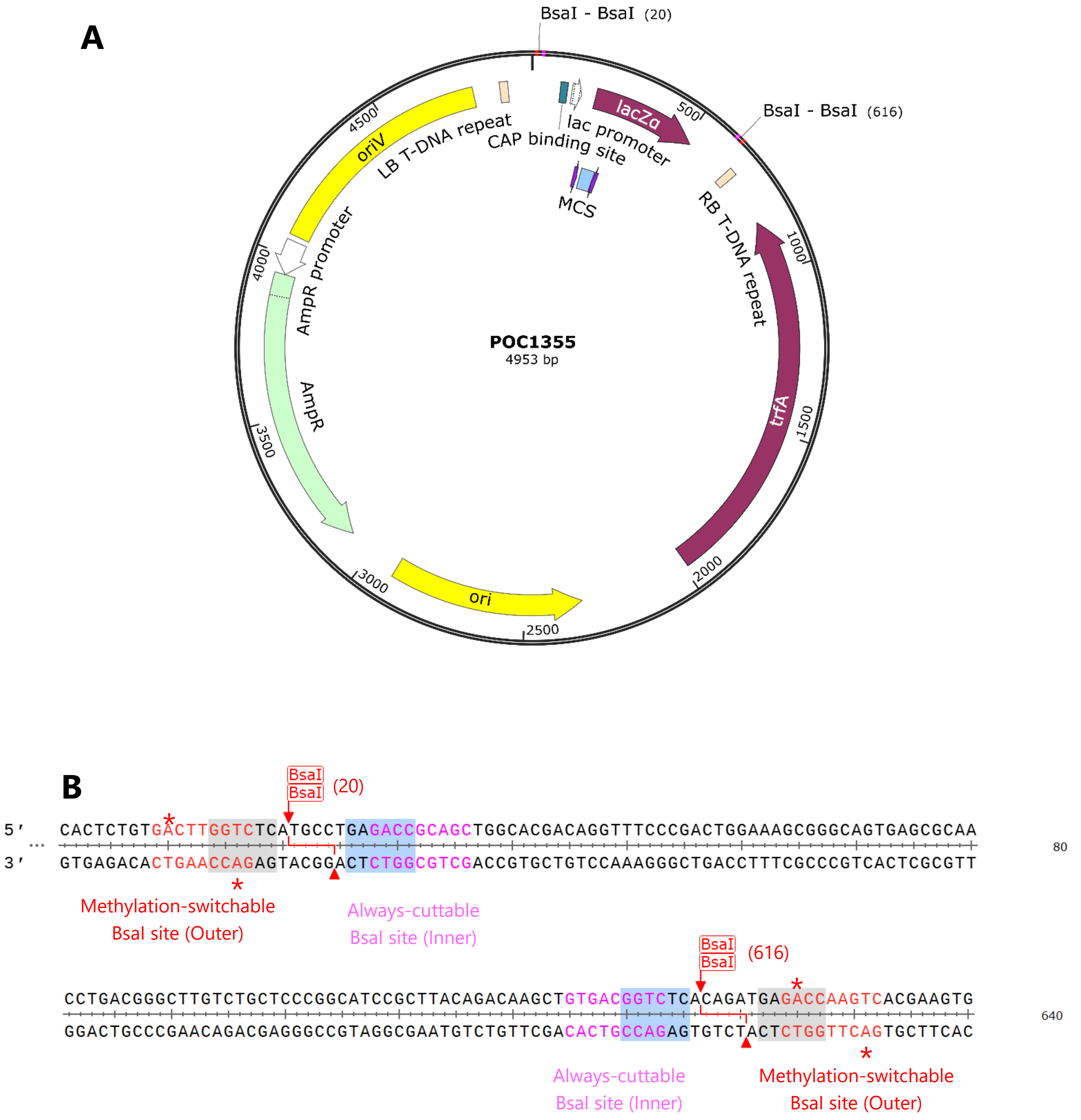

Figure S3. Assembly vector POC1355 designed to be methylated by the M.Osp807II switch methylase. (**A**) The vector contains two pairs of BsaI sites. Each pair is formed by an outer and an inner site. The outer sites are oriented to direct cutting inwards and the inner sites are oriented to direct cutting outwards. (**B**) The outer BsaI sites (GGTCTC) are partially overlapped by recognition sites for the methylase (in red with the methylated base marked with an asterisk) allowing methylation, and so forming **methylation-switchable** restriction sites. The inner BsaI sites are designed such that the adjacent sequence is not a recognition site for the methylase (magenta) and so they are not methylated and so are **always-cuttable** restriction sites.

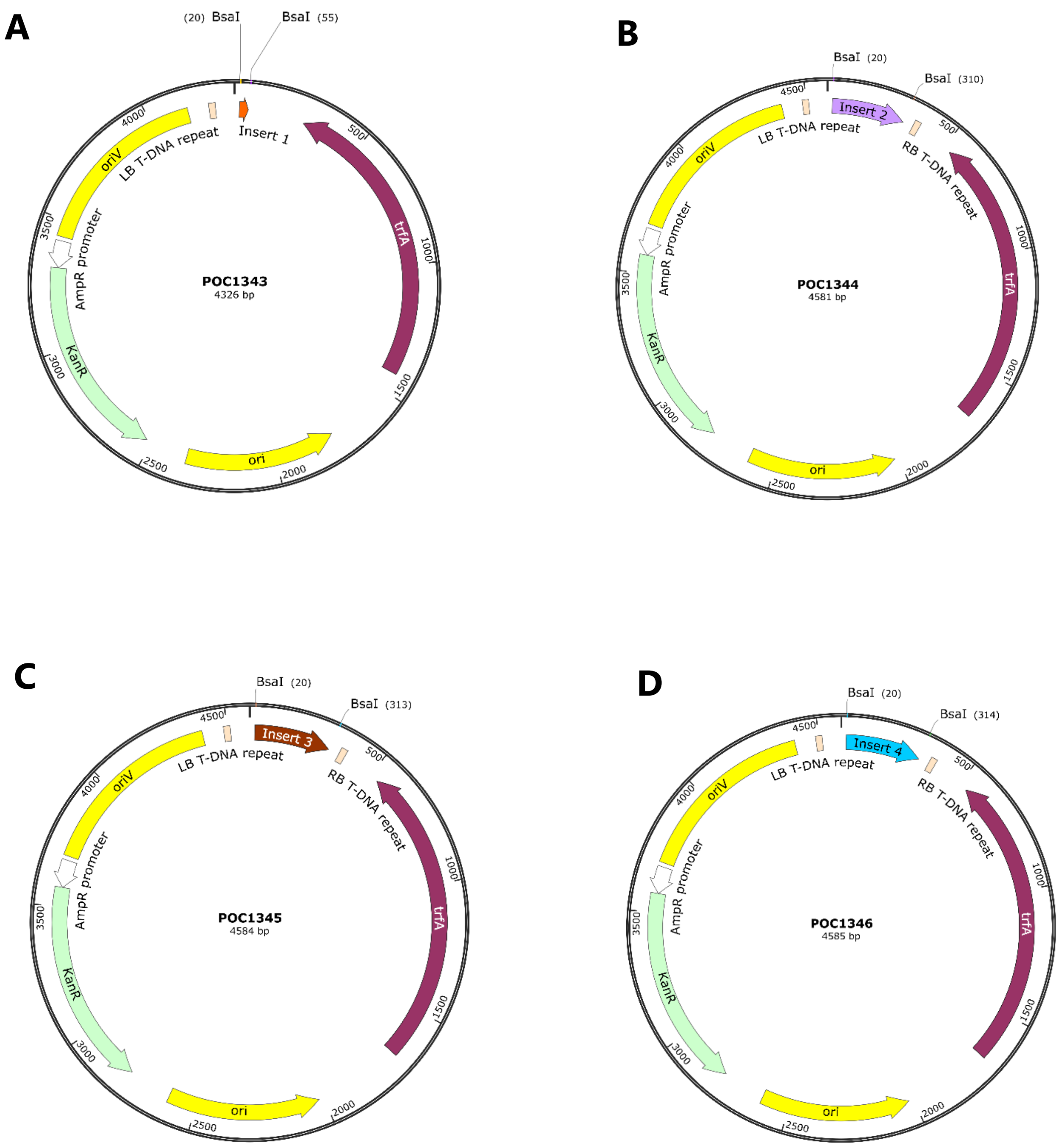

Figure S4. Donor plasmids POC1343, POC1344, POC1345 and POC1346. The donor plasmids (**A**) POC1343, (**B**) POC1344, (**C**) POC1345 and (**D**) POC1346 contain fragments: 1 (orange), 2 (purple), 3 (brown) and 4 (light blue), respectively. The fragments are flanked by unmethylated outer BsaI sites.

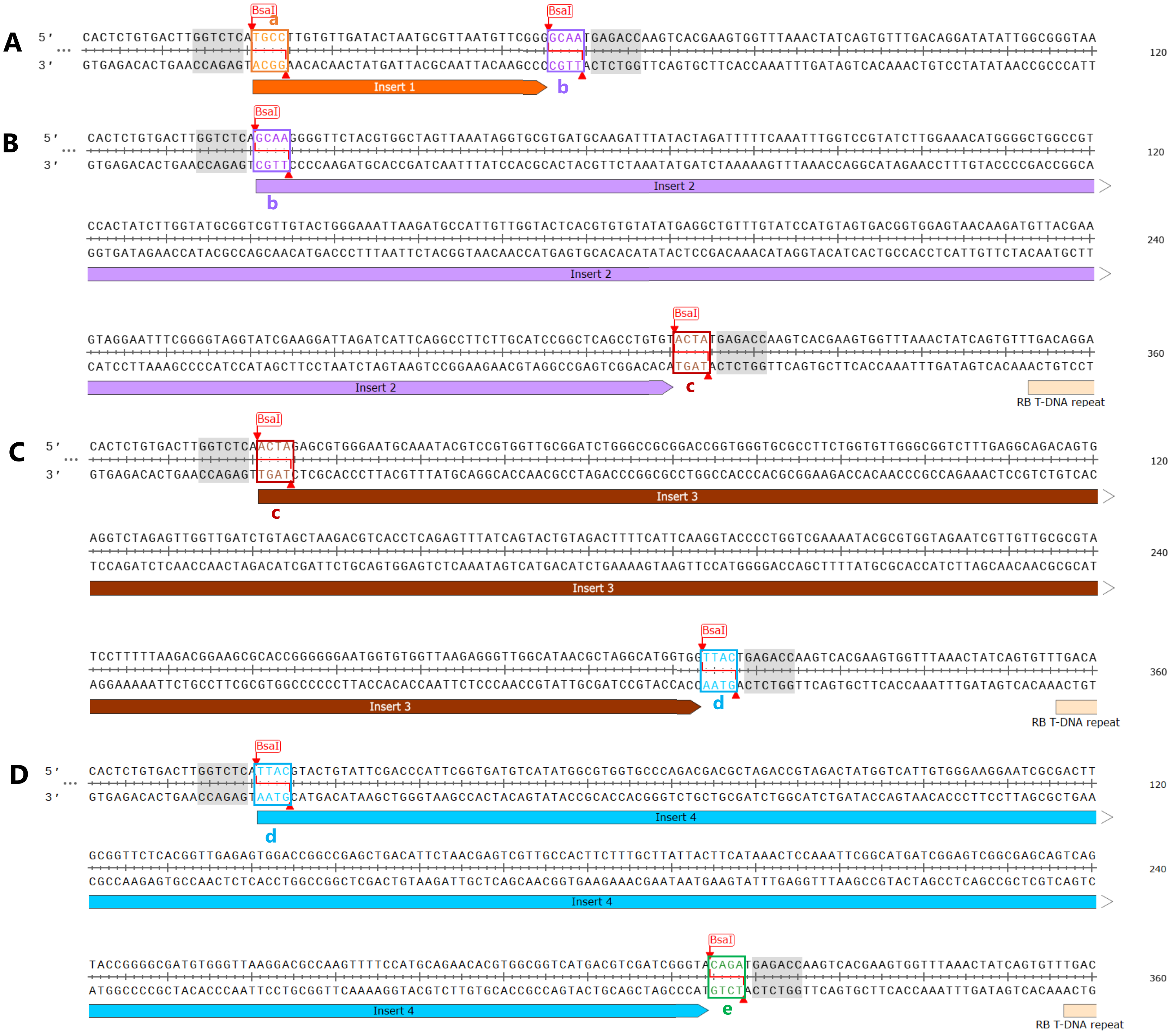

Figure S5. Sequences of fragments in donor plasmids POC1343, POC1344, POC1345 and POC1346. Fragment sequences. showing the fragments (**A**) 1 (orange), (**B**) 2 (purple), (**C**) 3 (brown) and (**D**) 4 (light blue) in POC1343, POC1344, POC1345 and POC1346, respectively, flanked by unmethylated outer *Bsa*I sites. The cohesive overhangs used during the assembly are labelled 'a', 'b', 'c', 'd' and 'e'. The BsaI sites are shaded in grey and red arrows indicate where the strands are cut.

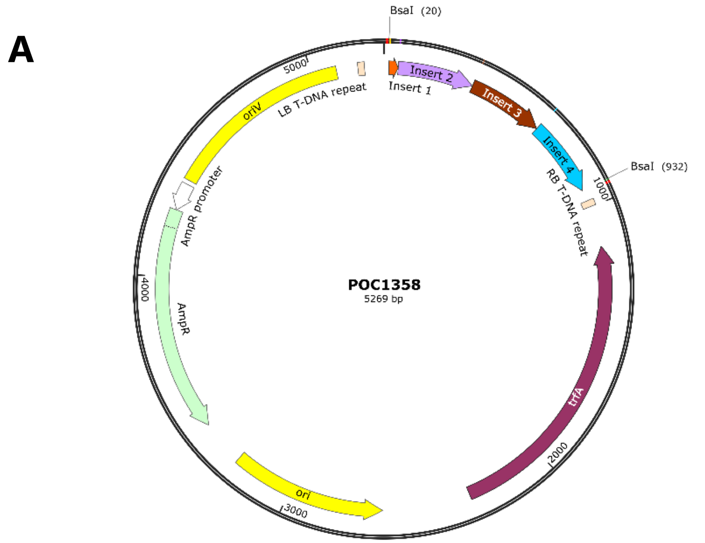

Figure S6. Assembled plasmid POC1358. (**A**) Assembled plasmid POC1358 with the assembled fragment flanked by BsaI sites. (**B**) Sequence showing the fragments correctly assembled due to the consecutive compatible overhangs within the fragment ('b', 'c', 'd'). The overhangs 'a' and 'e' determine the interaction with the assembly vector and so the orientation of the fragment in the final assembled plasmid. The flanking BsaI sites are the 'outer' BsaI sites of the assembly vector POC1355 and their methylation is removed by plasmid replication in an *E. coli* strain lacking the methylase. As these outer sites are are now unmethylated and so cuttable, they can be cut to release the fragment for a subsequent round of a multi-round assembly. The recognition sequence of the M.Osp807II BsaI-associated switch methylase is in red with the previously methylated base marked with an asterisk.

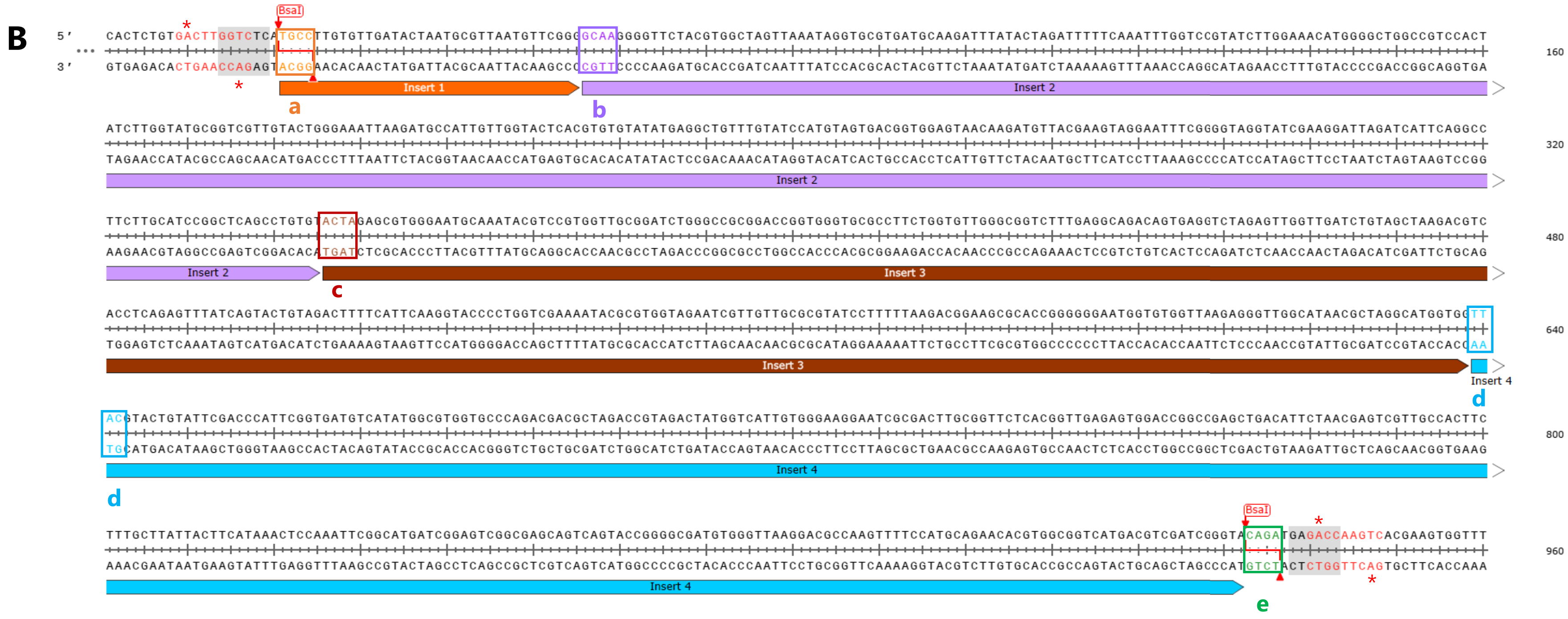

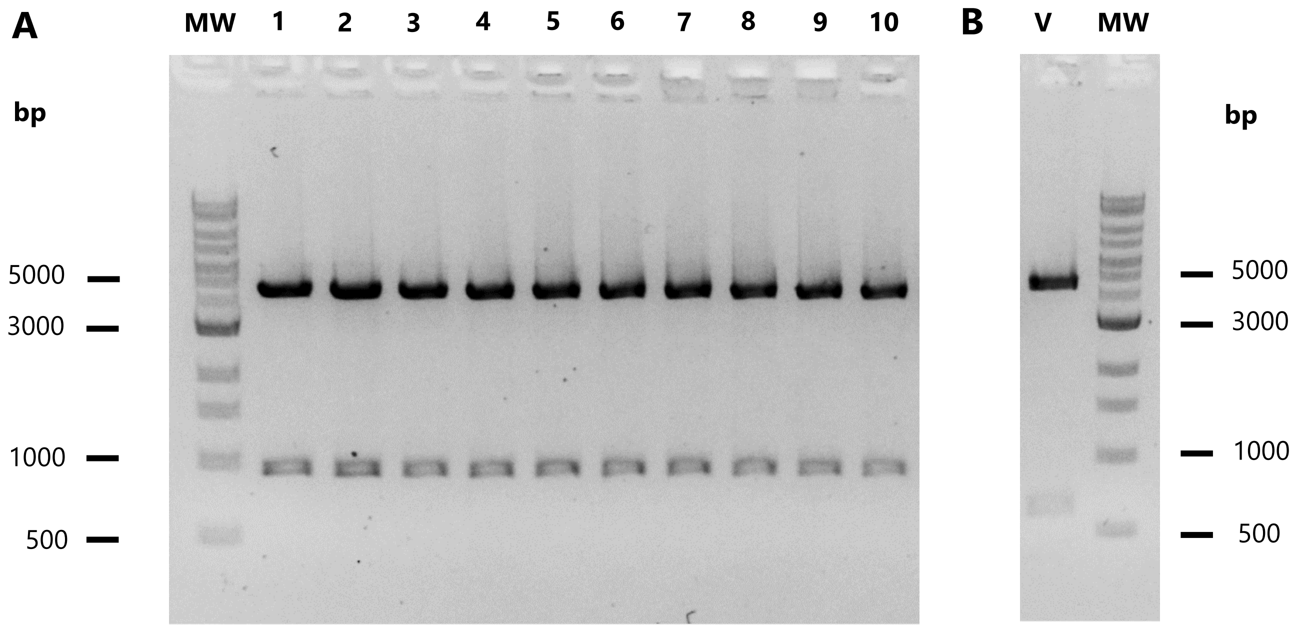

Figure S7. Agarose gel electrophoresis of POC1358. Agarose gel electrophoresis (1%) showing the restriction by BsaI of (**A**) the assembled plasmid POC1358 and (**B**) assembly vector POC1355. Lanes: 1 to 10, POC1358 extracted from ten white colonies; and lane V, POC1355. MW, molecular weight marker (Quick-Load® 1 kb Extend DNA Ladder, New England Biolabs, N3239S, 0.5 to 48.5 kb). The restriction of POC1358 results in a band of 912 bp corresponding to the assembled fragment, and a band of 4357 bp corresponding to the vector backbone. The restriction of the POC1355 results in bands of 596 and 4357 bp.

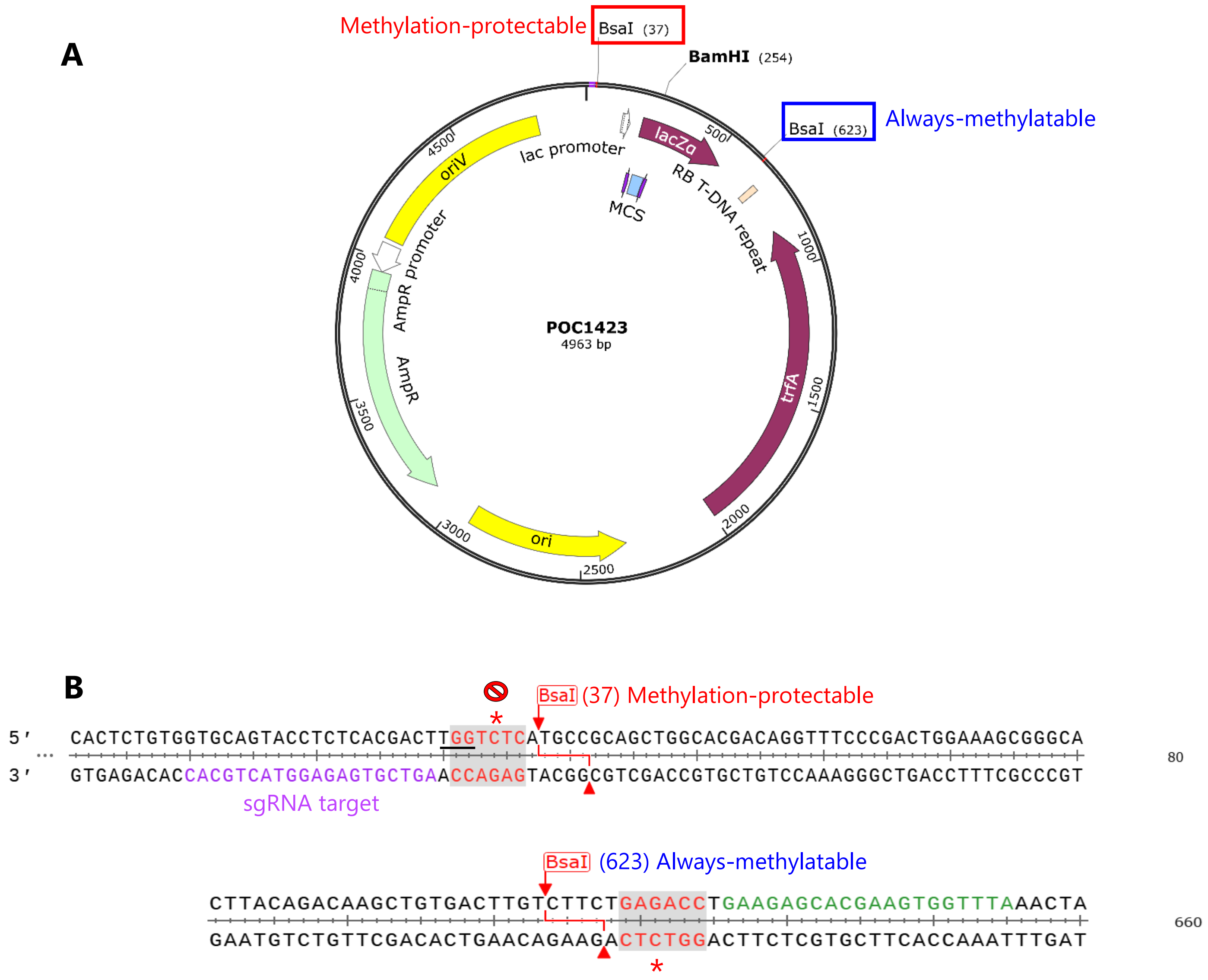

Figure S8. Design of plasmid POC1423 to test methylation protection. The plasmid POC1423 was designed to test targeted methylation-protection from the M2.Eco31I_2 non-switchable methylase using the methylation-protection dCas9/sgRNA molecule (MP-dCas9/sgRNA). (**A**) The plasmid contains a **methylation-protectable** (red box) and an **always-methylatable** BsaI site (blue box) in opposite orientations. There is a BamHI site between the two BsaI sites. (**B**) The BsaI recognition sequence (GGTCTC) fully overlaps and exactly matches that of the non-switchable methylase (GGTCTC, bases in red with the methylated base asterisked). In the methylation-protectable BsaI site, the upstream sequence was engineered to include an sgRNA target (purple) adjacent to a PAM sequence (underlined on the opposite strand). The MP-dCas9/sgRNA binds to the sgRNA target and PAM sequence, thus protecting the BsaI site from methylation (🚫) and allowing subsequent cutting by BsaI. At the always-methylatable site, the upstream sequence (green) does not include the sgRNA target, so methylation always occurs blocking subsequent cutting by BsaI.

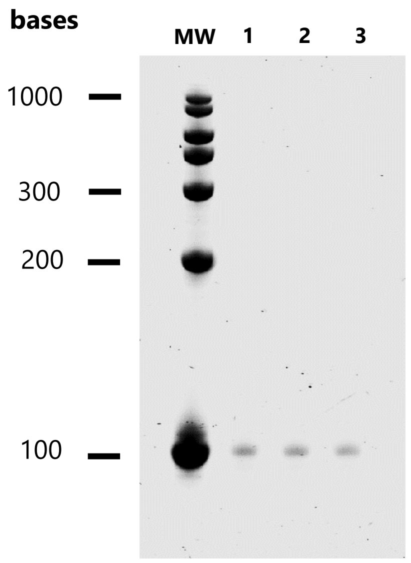

Figure S9. Polyacrylamide gel electrophoresis of sgRNA. 6% Novex™ TBE-Urea Gel (ThermoFisher) showing the purified *in vitro* transcribed sgRNA. Lanes: 1-3, sgRNA (100 nt). MW, molecular weight marker (RNA Century™-Plus Markers, ThermoFisher, AM7145, 100 to 1000 bases).

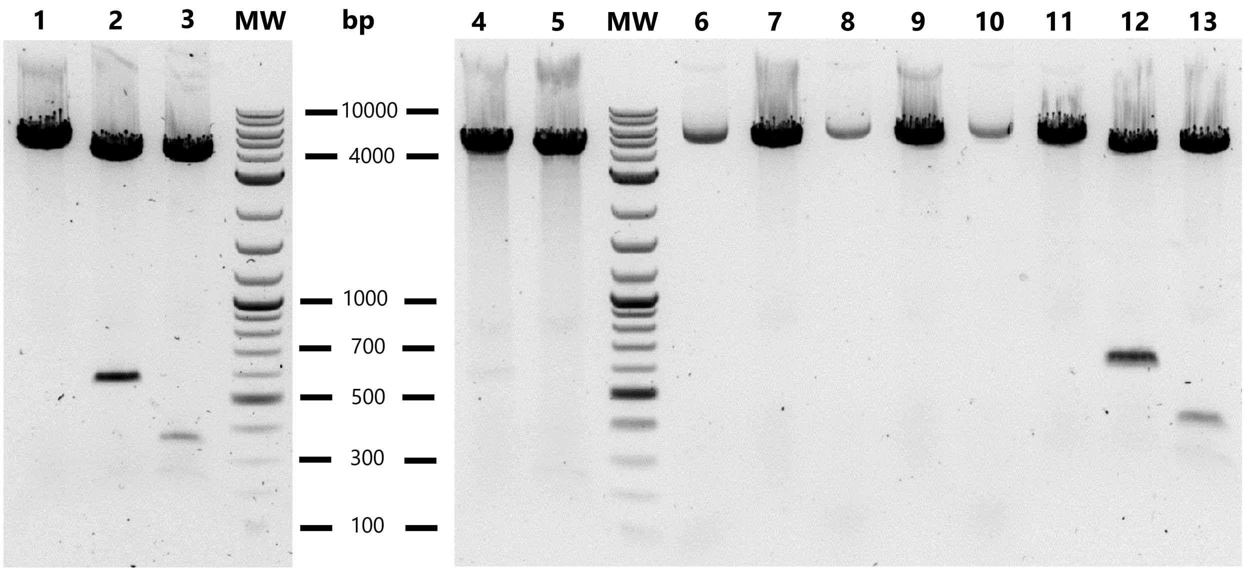

1	2	3	MW	Lane number	4	5	MW	6	7	8	9	10	11	12	13
+		+		BamHI		+			+		+		+		+
-	+	+		BsaI	+	+		+	+	+	+	+	+	+	+
-	-	-		Methylase (M2.Eco31I_2)	+	+		+	+	+	+	+	+	-	-
-	-	-		sgRNA	+	+		+	+	-	-	-	-	+	+
-	-	-		dCas9	+	+		-	-	+	+	-	-	+	+
-	-	-		dCas9+ sgRNA = MP-dCas9/sgRNA	+	+		-	-	-	-	-	-	+	+

Figure S10. Agarose gel electrophoresis (1%) showing the site-selective methylation-protection of POC1423 and related controls. Lanes: 1, digestion by BamHI (4963 bp); 2, digestion by BsaI (586 and 4377 bp); 3, digestion by both BsaI and BamHI (217, 369 and 4377 bp); 4, after methylation-protection (MP-dCas9/sgRNA and methylase) and digested by BsaI (4963 bp); 5, after methylation-protection and digested by both BsaI and BamHI (217 and 4746 bp); 6, without dCas9 but with methylase and digested by BsaI (circular plasmid); 7, without dCas9 but with methylase and digested by both BsaI and BamHI (4963 bp); 8, without sgRNA but with methylase and digested by BsaI (circular plasmid); 9, without sgRNA but with methylase and digested by both BsaI and BamHI (4963 bp); 10, without MP-dCas9/sgRNA but with methylase and digested by BsaI (circular plasmid); 11, without MP-dCas9/sgRNA but with methylase and digested by both BsaI and BamHI (4963 bp); 12, protected by MP-dCas9/sgRNA but without methylase and digested by BsaI (586 and 4377 bp); and 13, protected by MP-dCas9/sgRNA but without methylase and digested by both BsaI and BamHI (217, 369 and 4377 bp). The non-switchable methylase M2.Eco31I_2 was used. MW, molecular weight marker (Quick-Load® Purple 1 kb Plus DNA Ladder, New England Biolabs, N0550S, 0.1 to 10 kb).

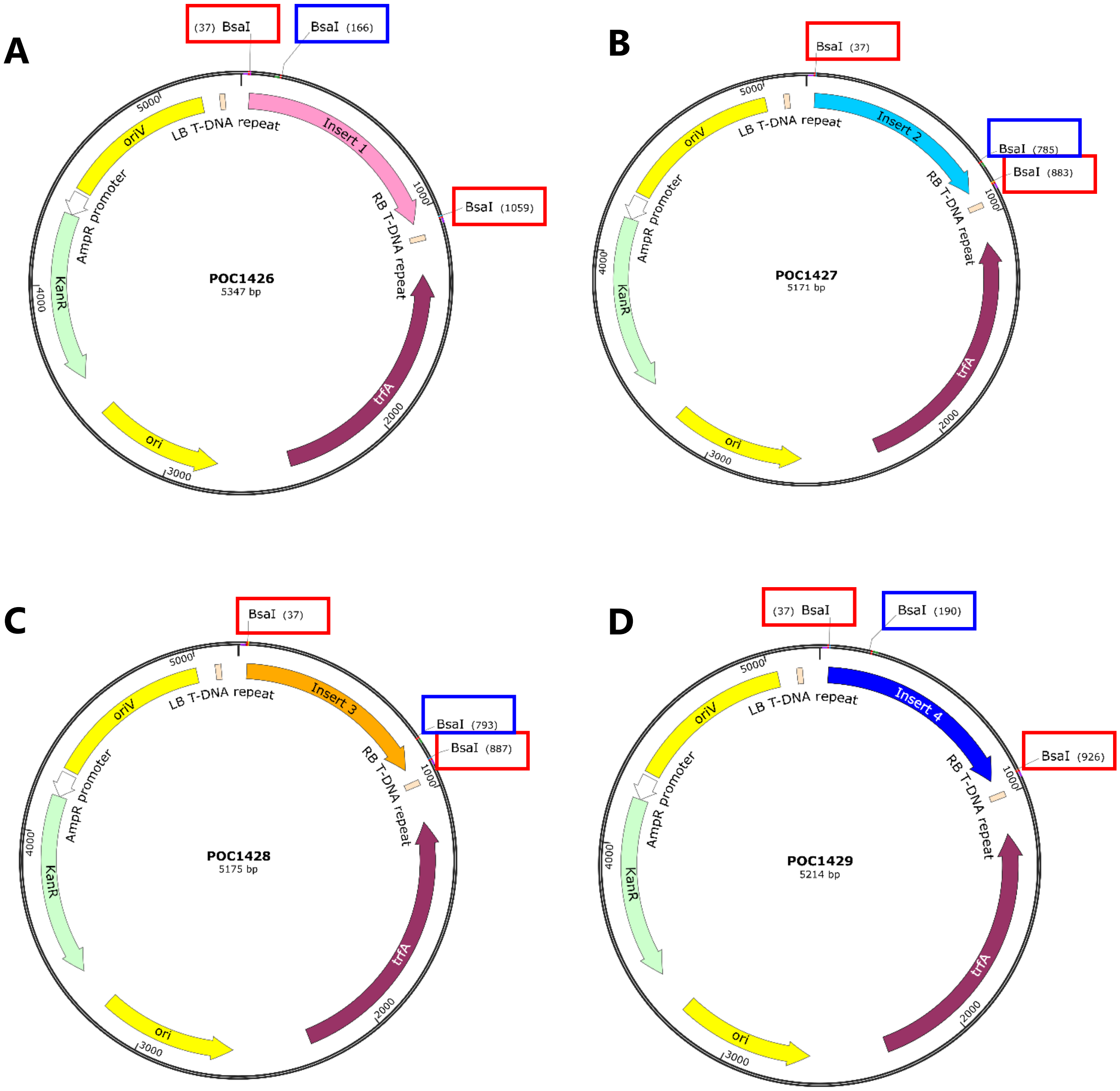

Figure S11. Donor plasmids POC1426, POC1427, POC1428 and POC1429. Donor plasmids (**A**) POC1426, (**B**) POC1427, (**C**) POC1428 and (**D**) POC1429 containing the fragments: 1 (pink), 2 (light blue), 3 (orange) and 4 (blue); respectively. These plasmids were designed to test the methylation-protection of fragments with internal BsaI sites methylation by the MP-dCas9/sgRNA and non-switchable methylases. Each fragment is flanked by 'outer' BsaI sites (red boxes) that are both **methylation-switchable and methylation-protectable** and each fragment contains an internal **always-methylatable** BsaI site (blue boxes).

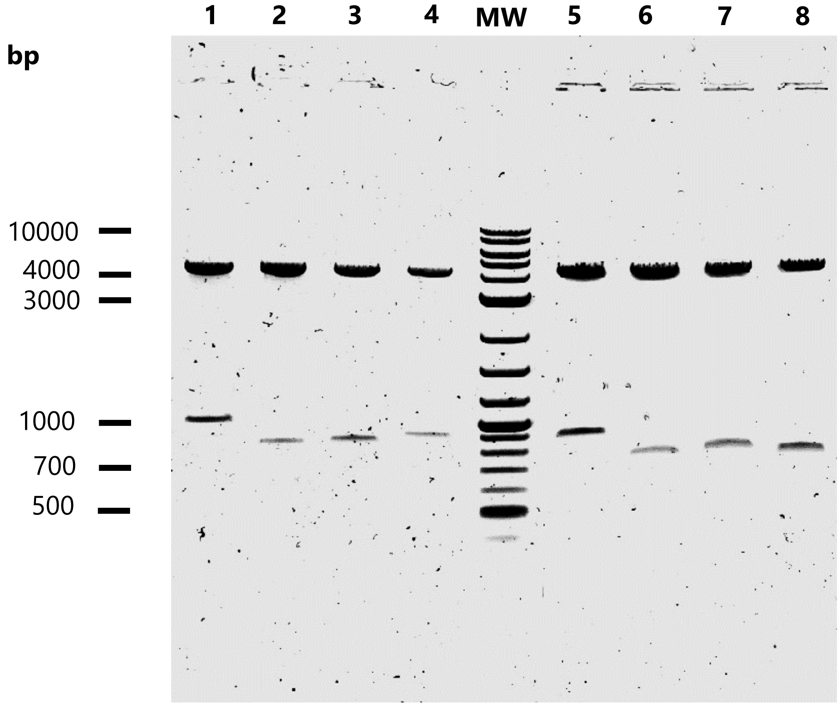

Figure S12. Methylation protection of POC1426, POC1427, POC1428 and POC1429. Agarose gel electrophoresis (1%) showing the site-selective methylation-protection of the donor plasmids POC1426, POC1427, POC1428 and POC1429 which each contain fragments that include one internal BsaI site (always-methylatable) and are flanked by outer BsaI sites (methylation-protectable). Lanes: 1, POC1426 after methylation-protection (MP-dCas9/sgRNA and methylase) and digested by BsaI (1022 and 4325 bp); 2, POC1427 after methylation-protection and digested by BsaI (846 and 4325 bp); 3, POC1428 after methylation-protection and digested by BsaI (850 and 4325 bp); 4, POC1429 after methylation-protection and digested by BsaI (889 and 4325 bp); 5, POC1426 digested by BsaI (129, 893 and 4325 bp); 6, POC1427 digested by BsaI (98, 748 and 4325 bp); 7, POC1428 digested by BsaI (94, 756 and 4325 bp); and 8, POC1429 digested by BsaI (153, 736 and 4325 bp). The non-switchable methylases M2.Eco31I was used for methylation of plasmids POC1426 and POC1427. The methylase M2.BsaI was used for methylation of plasmids POC1428 and POC1429. MW, molecular weight marker (Quick-Load® Purple 1 kb Plus DNA Ladder, New England Biolabs, N0550S, 0.1 to 10 kb). Bands of around 150 and below are not visible on the gel.

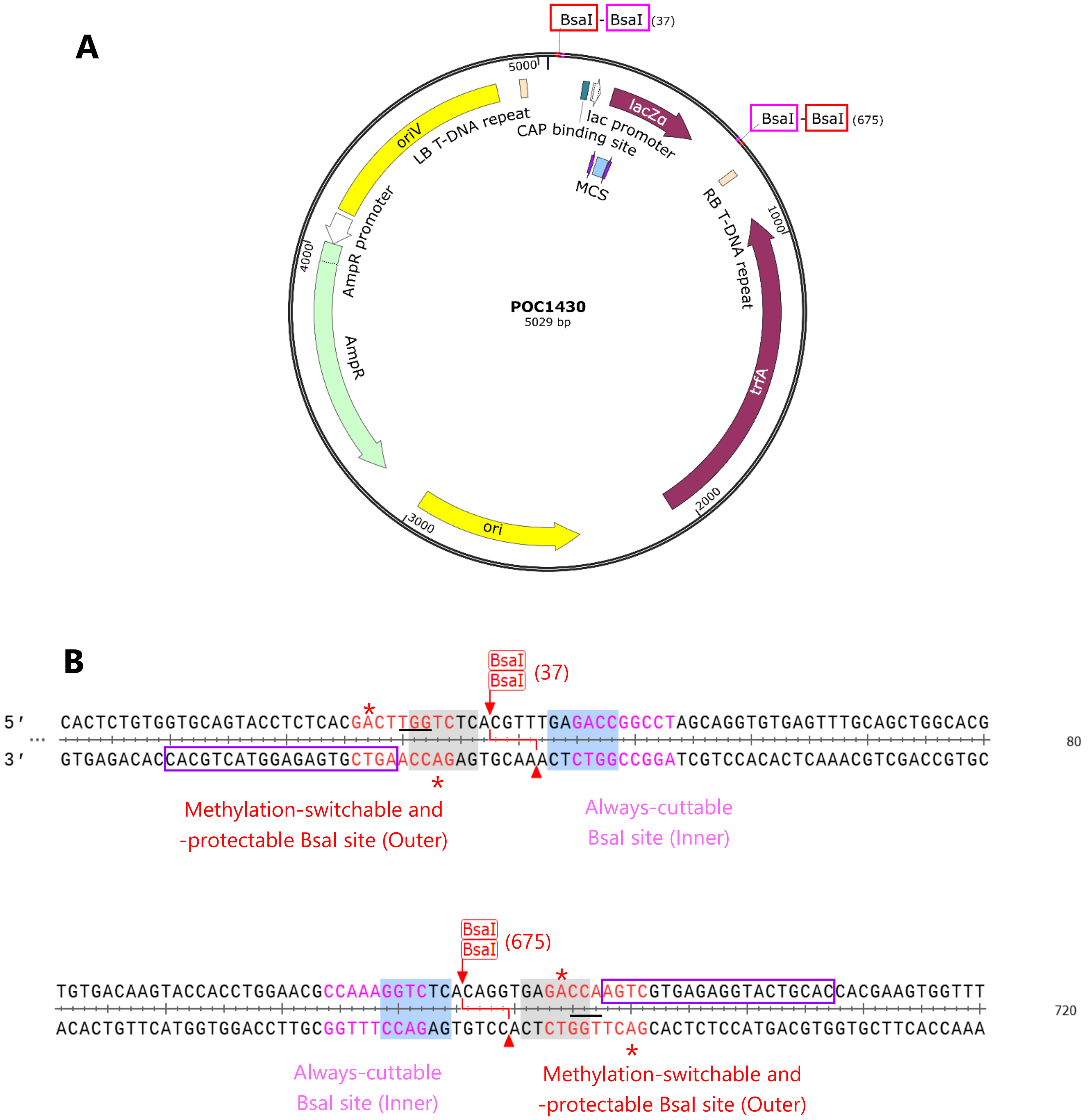

Figure S13. Design of assembly vector POC1430 to test DNA assembly using methylation-protection. Assembly vector POC1430 was designed to be methylated by the M.Osp807II switch methylase to test DNA assembly using methylation-protection approach. (**A**) The vector contains two pairs of *Bsa*I sites with each pair formed by an outer (red box) and an inner site (magenta box). The outer sites are oriented to direct cutting inwards and the inner sites are oriented to direct cutting outwards. (**B**) The 'outer' *Bsa*I sites (GGTCTC) partially overlap with the methylase recognition sequences (bases in red with the methylated base asterisked), so forming **methylation-switchable restriction sites**. These outer sites are also **methylation-protectable** because they have an upstream sequence that includes the sgRNA target (purple box) and the PAM sequence (underlined). At the inner BsaI sites, the upstream sequence (magenta) does not include either a recognition site for the methylase nor an sgRNA target and so these BsaI sites are **always-cuttable restriction sites**.

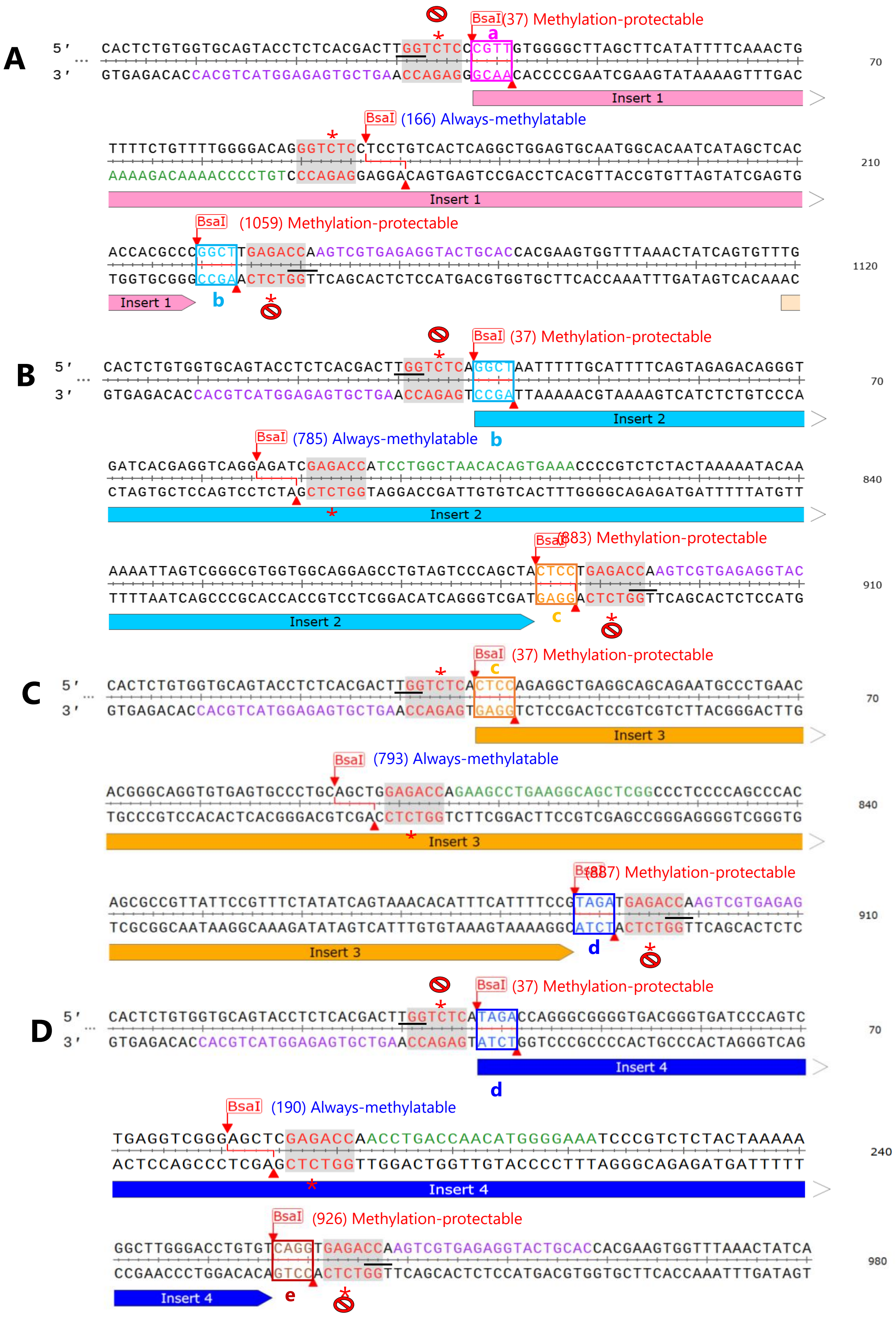
A
5′ ... CACTCTGTGGTGCAGTACCTCTCACGACTTGGTCTCCCGTTGTGGGGCTTAGCTTCATATTTTCAAACTG
3′ GTGAGACACCACGTCATGGAGAGTGCTGAACCAGAGGGCAACACCCCGAATCGAAGTATAAAAGTTTGAC
BsaI (37) Methylation-protectable
a
70
Insert 1
BsaI (166) Always-methylatable
TTTTCTGTTTTGGGGACAGGGTCTCCTCCTGTCACTCAGGCTGGAGTGCAATGGCACAATCATAGCTCAC
AAAAGACAAAACCCCTGTCCCAGAGGAGGACAGTGAGTCCGACCTCACGTTACCGTGTTAGTATCGAGTG
210
Insert 1
BsaI (1059) Methylation-protectable
ACCACGCCCGGCTTGAGACCAAGTCGTGAGAGGTACTGCACCACGAAGTGGTTTAAACTATCAGTGTTTG
TGGTGCGGGCCGAACTCTGGTTCAGCACTCTCCATGACGTGGTGCTTCACCAAATTTGATAGTCACAAAC
1120
Insert 1
b
B
BsaI (37) Methylation-protectable
5′ ... CACTCTGTGGTGCAGTACCTCTCACGACTTGGTCTCAGGCTAATTTTTGCATTTTCAGTAGAGACAGGGT
3′ GTGAGACACCACGTCATGGAGAGTGCTGAACCAGAGTCCGATTAAAAACGTAAAAGTCATCTCTGTCCCA
70
Insert 2
BsaI (785) Always-methylatable
b
GATCACGAGGTCAGGAGATCGAGACCATCCTGGCTAACACAGTGAAACCCCGTCTCTACTAAAAATACAA
CTAGTGCTCCAGTCCTCTAGCTCTGGTAGGACCGATTGTGTCACTTTGGGGCAGAGATGATTTTTATGTT
840
Insert 2
BsaI (883) Methylation-protectable
AAAATTAGTCGGGCGTGGTGGCAGGAGCCTGTAGTCCCAGCTACTCCTGAGACCAAGTCGTGAGAGGTAC
TTTTAATCAGCCCGCACCACCGTCCTCGGACATCAGGGTCGATGAGGACTCTGGTTCAGCACTCTCCATG
910
Insert 2
c
C
BsaI (37) Methylation-protectable
c
5′ ... CACTCTGTGGTGCAGTACCTCTCACGACTTGGTCTCACTCCAGAGGCTGAGGCAGCAGAATGCCCTGAAC
3′ GTGAGACACCACGTCATGGAGAGTGCTGAACCAGAGTGAGGTCTCCGACTCCGTCGTCTTACGGGACTTG
70
Insert 3
BsaI (793) Always-methylatable
ACGGGCAGGTGTGAGTGCCCTGCAGCTGGAGACCAGAAGCCTGAAGGCAGCTCGGCCCTCCCCAGCCCAC
TGCCCGTCCACACTCACGGGACGTCGACCTCTGGTCTTCGGACTTCCGTCGAGCCGGGAGGGGTCGGGTG
840
Insert 3
BsaI (887) Methylation-protectable
AGCGCCGTTATTCCGTTTCTATATCAGTAAACACATTTCATTTTCCGTAGATGAGACCAAGTCGTGAGAG
TCGCGGCAATAAGGCAAAGATATAGTCATTTGTGTAAAGTAAAAGGCATCTACTCTGGTTCAGCACTCTC
910
Insert 3
d
D
BsaI (37) Methylation-protectable
5′ ... CACTCTGTGGTGCAGTACCTCTCACGACTTGGTCTCATAGACCAGGGCGGGGTGACGGGTGATCCCAGTC
3′ GTGAGACACCACGTCATGGAGAGTGCTGAACCAGAGTATCTGGTCCCGCCCCACTGCCCACTAGGGTCAG
70
Insert 4
d
BsaI (190) Always-methylatable
TGAGGTCGGGAGCTCGAGACCAACCTGACCAACATGGGAAATCCCGTCTCTACTAAAAA
ACTCCAGCCCTCGAGCTCTGGTTGGACTGGTTGTACCCTTTAGGGCAGAGATGATTTTT
240
Insert 4
BsaI (926) Methylation-protectable
GGCTTGGGACCTGTGTCAGGTGAGACCAAGTCGTGAGAGGTACTGCACCACGAAGTGGTTTAAACTATCA
CCGAACCCTGGACACAGTCCACTCTGGTTCAGCACTCTCCATGACGTGGTGCTTCACCAAATTTGATAGT
980
Insert 4
e

Figure S14. Sequences of fragments in donor plasmids POC1426, POC1427, POC1428 and POC1429. Sequences showing the fragments (**A**) 1 (pink), (**B**) 2 (light blue), (**C**) 3 (orange), and (**D**) 4 (blue) in plasmids POC1426, POC1427, POC1428 and POC1429 respectively. Each fragment is flanked by outer BsaI sites that are both **methylation-switchable and methylation-protectable** and each fragment contains an internal **always-methylatable** BsaI site. The BsaI recognition sequence (GGTCTC) fully overlaps and exactly matches that of the non-switchable methylases M2.Eco31I and M2.BsaI (GGTCTC, bases in red). The base methylated by M2.Eco31I is asterisked. In the methylation-protectable outer BsaI site, the upstream sequence was engineered to include an sgRNA target (purple) adjacent to a PAM sequence (underlined on the opposite strand). At the always-methylatable internal site, the upstream sequence (green) does not include the sgRNA target, so methylation always occurs blocking subsequent cutting by BsaI. The cohesive overhangs used during the assembly are labelled 'a', 'b', 'c', 'd' and 'e'. Bases protected from methylation are marked '🚫'.

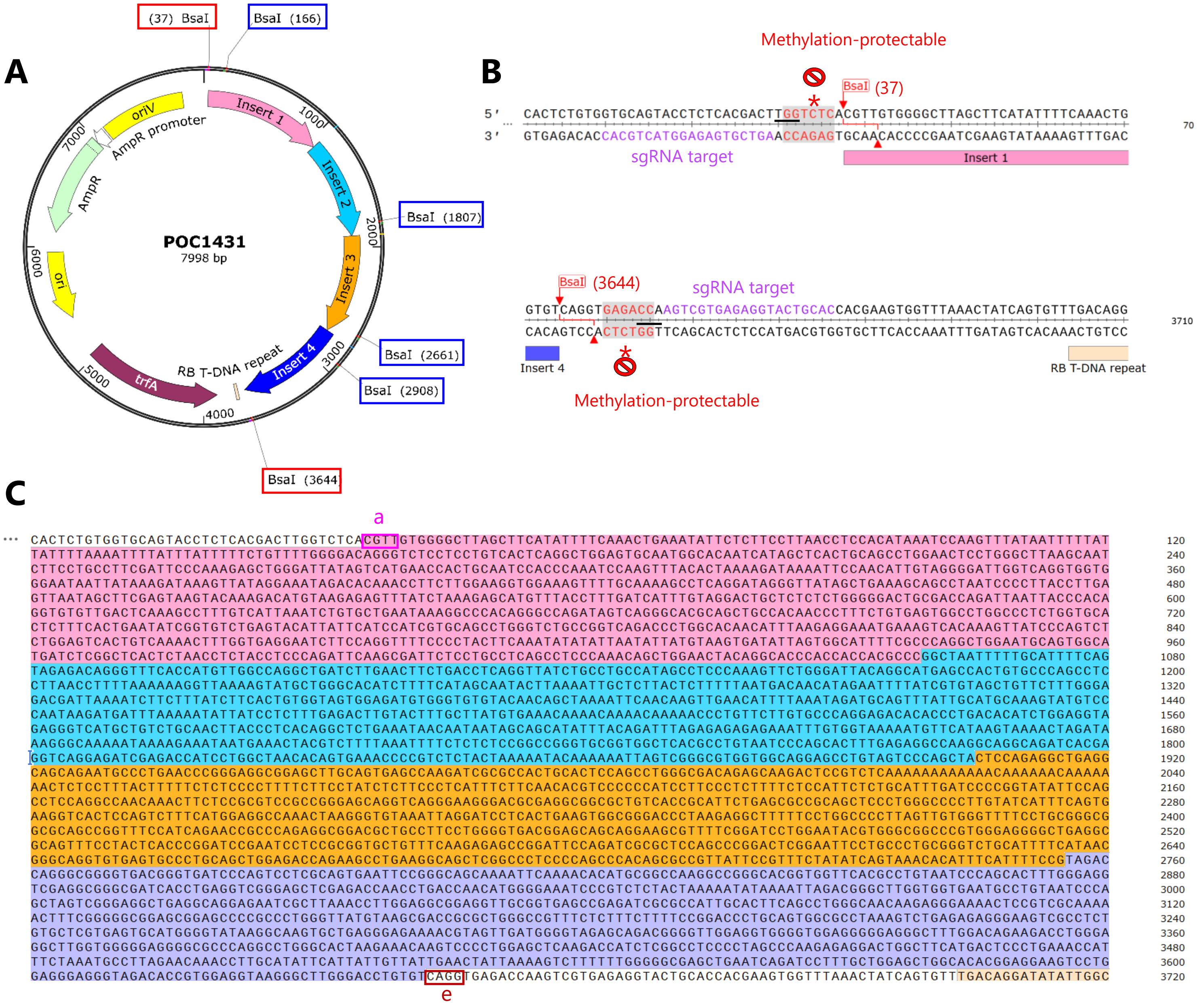

Figure S15. Assembled plasmid POC1431. (**A**) Assembled plasmid POC1431 with the four fragments assembled using the methylation-protection approach. The assembled fragment is flanked by 'outer' BsaI sites (red boxes) and contains four internal BsaI sites which are always-methylatable (blue boxes). (**B**) The 'outer' flanking BsaI sites are methylation-protectable because their upstream sequences includes the sgRNA target (purple) and the PAM sequence (underlined). Thus, this assembled plasmid can be used as a donor in a next round of assembly. The recognition sequence of the M2.Eco31I and M2.BsaI non-switchable methylases fully overlap and exactly match the BsaI recognition site (GGTCTC, bases shown in red). The base methylated by M2.Eco31I is asterisked. (**C**) Sequence showing the fragments correctly assembled. The terminal overhangs 'a' (pink box: CGTT) and 'e' (brown box: CAGG) of the fragments are compatible with those of the original assembly vector POC1430. The methylation is removed transforming the assembled plasmid into a *E. coli* lacking the methylases.

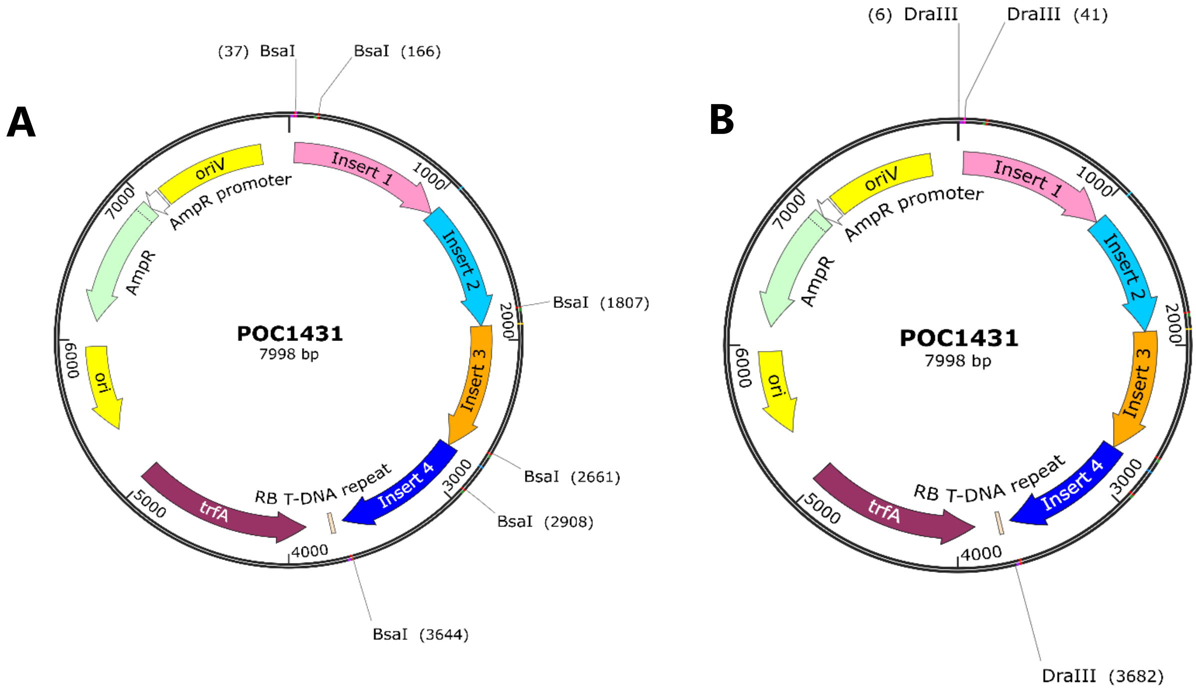

Figure S16. Restriction sites in the assembled plasmid POC1431. (**A**) The assembled plasmid POC1431 has six BsaI sites and digestion with BsaI should yield six bands of 129, 247, 736, 854, 1641, and 4391 bp. (**B**) POC1431 has three DraIII sites and digestion with BsaI should yield three bands of 35, 3641, and 4322 bp.

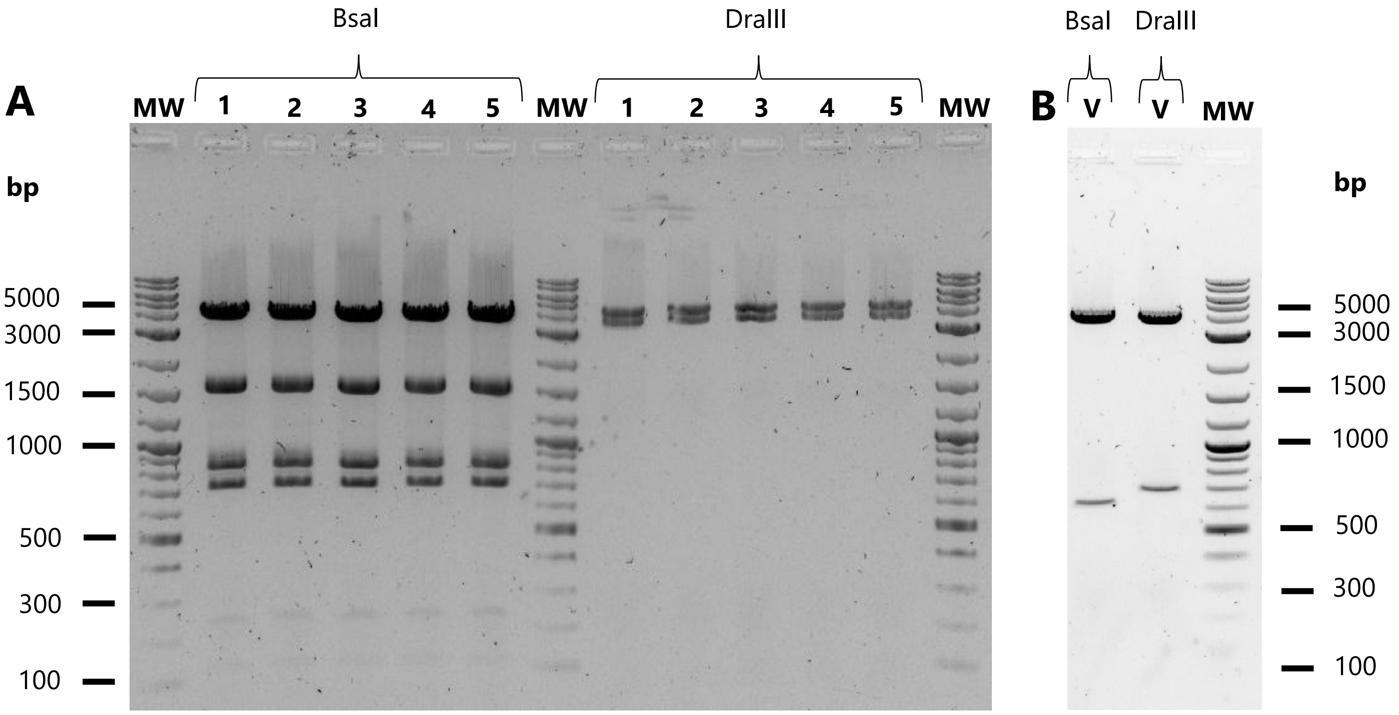

Figure S17. Agarose gel electrophoresis of POC1431 assembled after methylation using M2.Eco31I. (**A**) Agarose gel electrophoresis (1%) showing the restriction by BsaI and DraIII of the assembled plasmid POC1431 and (**B**) assembly vector POC1430. Lanes: 1 to 5, POC1431 extracted from five white colonies; and V, POC1430. MW, molecular weight marker (Quick-Load® Purple 1 kb Plus DNA Ladder, New England Biolabs, N0550S, 0.1 to 10 kb). The restriction of POC1431 by BsaI will result in six bands of 129, 247, 736, 854, 1641, and 4391 bp; and by DraIII will result in three bands of 35, 3641, and 4322 bp. The restriction of POC1430 by BsaI will result in two bands of 638 and 4391 bp, and by DraIII in two bands of 707 and 4322. Bands less than 100 bp are not observed in the gel. The assembly vector was methylated by the M.Osp807II switch methylase and the fragments by the M2.Eco31I non-switchable methylase.

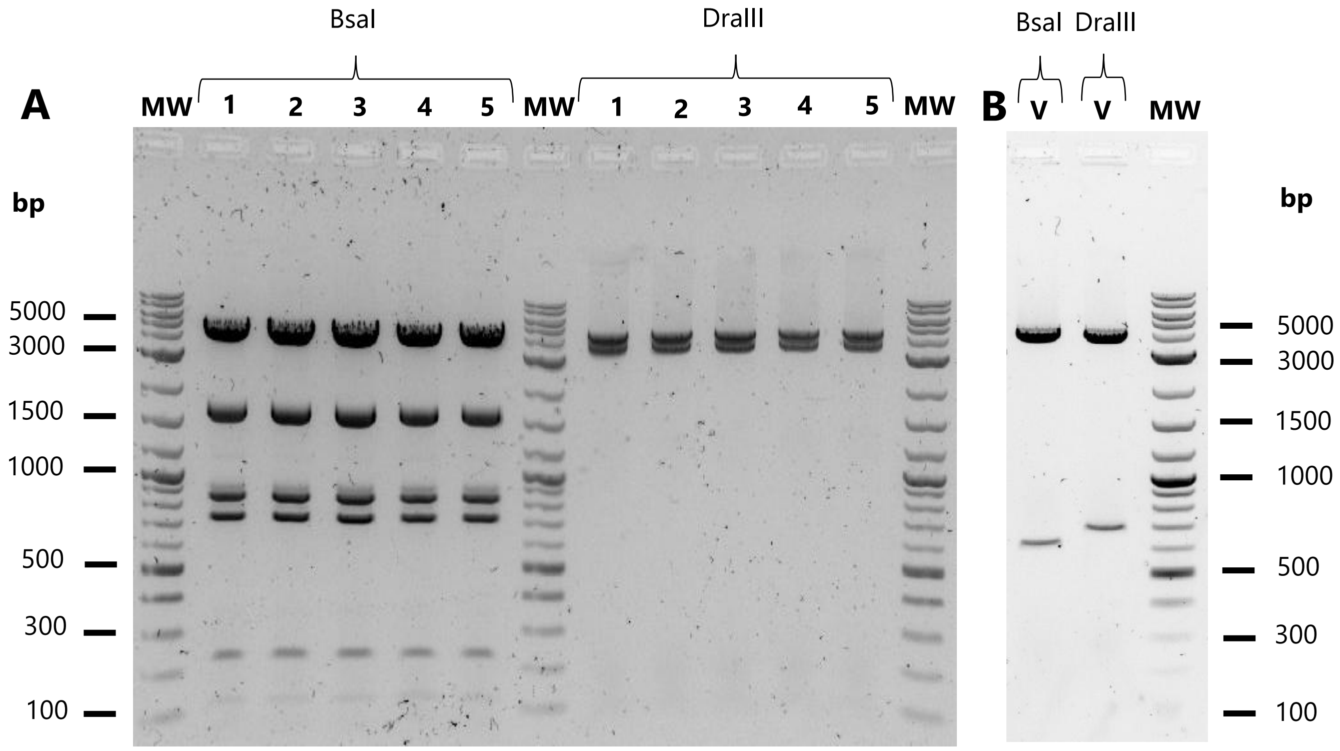

Figure S18. Agarose gel electrophoresis of POC1431 assembled after methylation using M2.Eco31I_2. (**A**) Agarose gel electrophoresis (1%) showing the restriction by BsaI and DraIII of the assembled plasmid POC1431 and (**B**) assembly vector POC1430. Lanes: 1 to 5, POC1431 extracted from five white colonies; and V, POC1430. MW, molecular weight marker (Quick-Load® Purple 1 kb Plus DNA Ladder, New England Biolabs, N0550S, 0.1 to 10 kb). The restriction of POC1431 by BsaI will result in six bands of 129, 247, 736, 854, 1641, and 4391 bp; and by DraIII will result in three bands of 35, 3641, and 4322 bp. The restriction of POC1430 by BsaI will result in two bands of 638 and 4391 bp, and by DraIII in two bands of 707 and 4322. Bands less than 100 bp are not observed in the gel. The assembly vector was methylated by the M.Osp807II switch methylase and the fragments by the M2.Eco31I_2 non-switchable methylase. M2.Eco31I_2 is a truncated form of M2.Eco31I and both isoforms share the same recognition site and cutting characteristics.

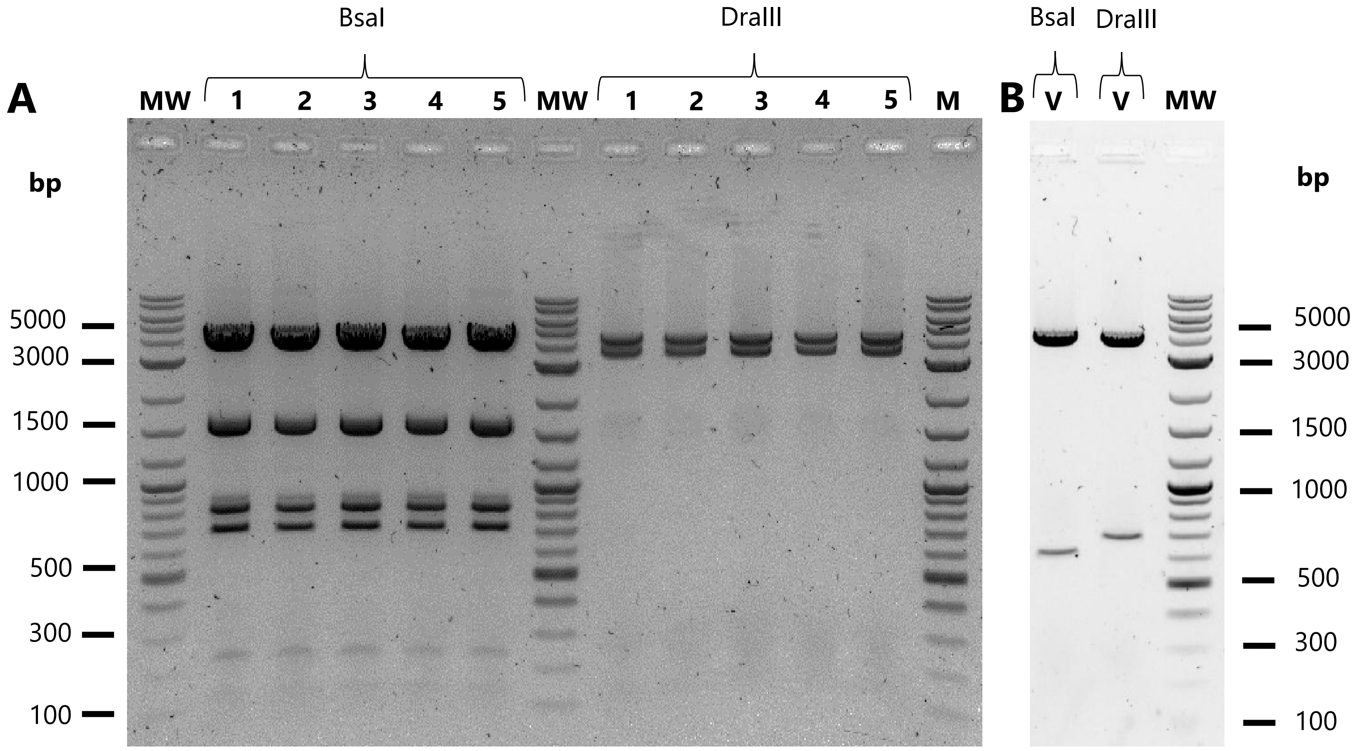

Figure S19. Agarose gel electrophoresis of POC1431 assembled after methylation using M2.BsaI. (**A**) Agarose gel electrophoresis (1%) showing the restriction by BsaI and DraIII of the assembled plasmid POC1431 and (**B**) assembly vector POC1430. Lanes: 1 to 5, POC1431 extracted from five white colonies; and V, POC1430. MW, molecular weight marker (Quick-Load® Purple 1 kb Plus DNA Ladder, New England Biolabs, N0550S, 0.1 to 10 kb). The restriction of POC1431 by BsaI will result in six bands of 129, 247, 736, 854, 1641, and 4391 bp; and by DraIII will result in three bands of 35, 3641, and 4322 bp. The restriction of POC1430 by BsaI will result in two bands of 638 and 4391 bp, and by DraIII in two bands of 707 and 4322. Bands less than 100 bp are not observed in the gel. The assembly vector was methylated by the M.Osp807II switch methylase and the fragments by the M2.BsaI non-switchable methylase.

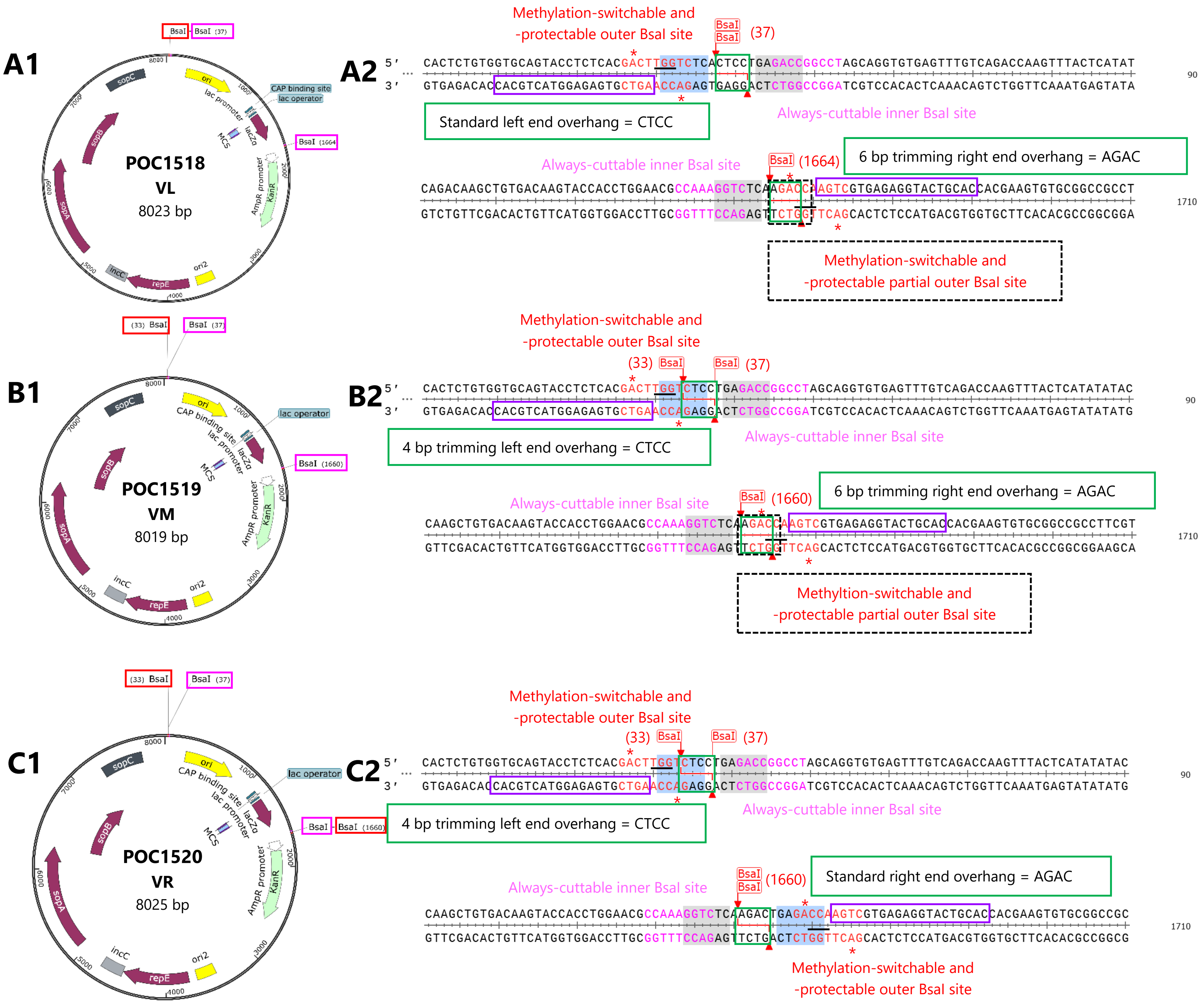

Figure S20. VL, VM and VR assembly vectors for scarless DNA assembly. Assembly vectors for scarless type IIS enzyme-based assembly using the methylation-protection approach, and the M.Osp807II switch methylase. (**A**) Vector Left, VL (POC1518). (**B**) Vector Middle, VM; and (POC1519). (**C**) Vector Right, VR (POC1520). **(A1), (B1) and (C1)** Vector maps showing the BsaI sites (methylation-switchable and protectable in red boxes, and always-cuttable in magenta boxes). **(A2), (B2) and (C2)** Sequence details of the assembly vectors. All the vectors have the left overhangs (CTCC) and the same right overhang (AGAC) shown in green boxes. The recognition sequence of the methylase is in red with the methylated base asterisked, the sgRNA target is in a purple box, the PAM sequence is underlined, and the altered recognition sequence of the methylase is in magenta. The dashed boxes indicate the partial outer BsaI sites (GGTCT) that will be methylated. These partial sites will be reconstituted (GGTCTC) after the assembly.

A. First round of assembly

[Fragment 1-1] (u, b) + [Fragment 1-2] (b, c) + [Fragment 1-3] (c, d) + [Fragment 1-4] (d, v) —VL (u, v)→ [Fragment 2_1] (u, e)

[Fragment 1-5] (u, f) + [Fragment 1-6] (f, g) + [Fragment 1-7] (g, v) —VM (u, v)→ [Fragment 2_2] (e, h)

[Fragment 1-8] (u, i) + [Fragment 1-9] (i, v) —VM (u, v)→ [Fragment 2_3] (h, j)

[Fragment 1-10] (u, k) + [Fragment 1-11] (k, v) —VR (u, v)→ [Fragment 2_4] (j, v)

B. Second round of assembly

[Fragment 2_1] (u, e) + [Fragment 2_2] (e, h) + [Fragment 2_3] (h, j) + [Fragment 2_4] (j, v) —VM (u, v)→ [Fragment 3_1] (a, l)

Figure S21. Scarless hierarchical assembly scheme for 11 fragments using UniClo. (**A**) The fragments in donor plasmids are assembled in the first round in four groups in assembly vectors VL, VM, and VR. The letters in green or blue symbolise the left and right overhangs used in that round of the assembly. The green overhangs (u and v) represent overhangs generated from the vector adaptor sequence. The blue overhangs (b-j) represent overhangs generated from within the desired sequence for assembly (**B**) The new donor plasmids containing the assembled fragments from the first round donate fragments for the second round to produce the final assembled fragment in the VM assembly vector which can be cut out using BsaI to generate fragment 3_1 with overhangs 'a' and 'l'.

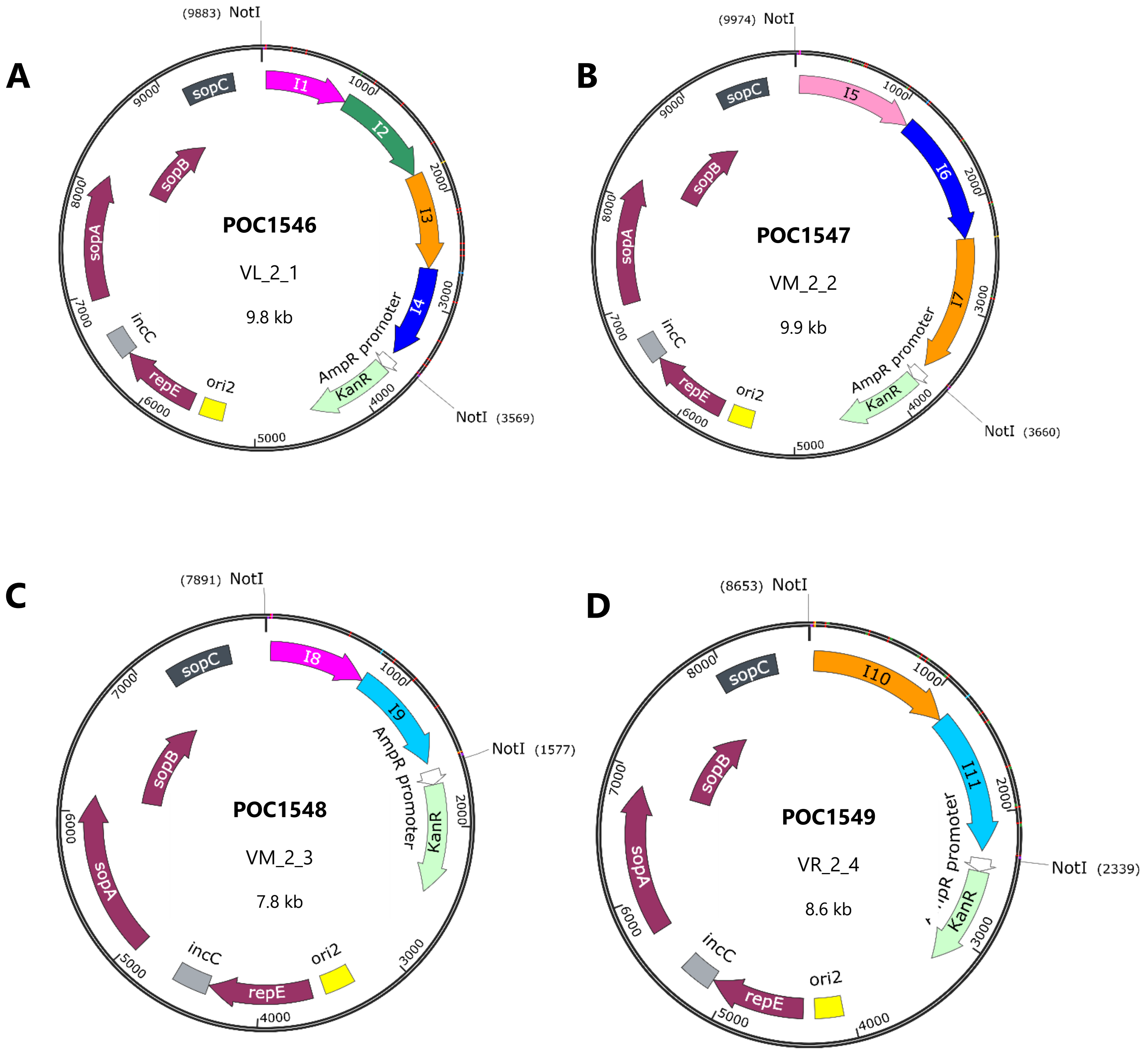

Figure S22. Assembled plasmids from the first round of scarless hierarchical assembly using UniClo. Assembled plasmids from the first round of the scarless hierarchical assembly using UniClo showing the two NotI restriction sites used to confirm correct assembly. (**A**) POC1546, (**B**) POC1547, (**C**) POC1548 and (**D**) POC1549 containing the assembled fragments 2_1, 2_2, 2_3 and 2_4, respectively.

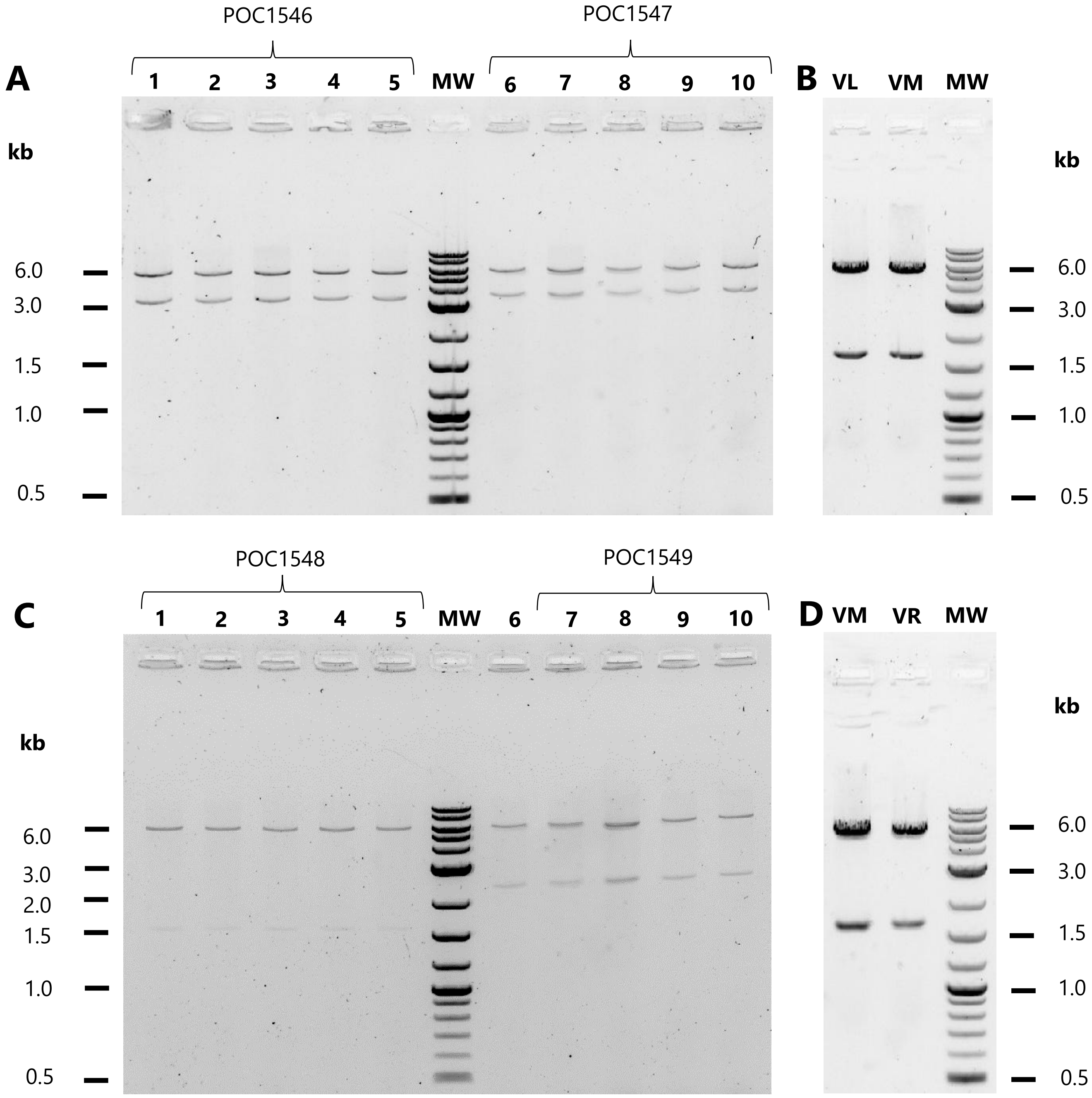

Figure S23. Agarose gel electrophoresis of the assembled plasmids from the first round of the hierarchical assembly using UniClo. Agarose gel electrophoresis (1%) showing restriction by NotI of the assembled plasmids POC1546, POC1547, POC1549 and POC1549 from the first round of assembly; and the assembly vectors VL (POC1518), VM (POC1519) and VR (POC1520) as controls. (**A**) Lanes: 1 to 5, POC1546; and lanes 6 to 10, POC1547 containing the assembled fragments 2_1 and 2_2, respectively. (**B**) Lanes: VL, assembly vector VL; and VM, assembly vector VM. (**C**) Lanes: 1 to 5, POC1548; and lanes 6 to 10, POC1549 containing the assembled fragments 2_3 and 2_4, respectively. (**D**) Lanes: VM, assembly vector VM; and VR, assembly vector VR. MW, molecular weight marker (Quick-Load® Purple 1 kb Plus DNA Ladder, New England Biolabs, N0550S, 0.1 to 10 kb). The assembled plasmids were extracted from five white colonies. For all the assembled plasmids, the restriction digestion analysis showed a band of 6.3 kb corresponding to the plasmid backbone and bands of 3.5, 3.5, 1.5, and 2.3 kb corresponding to the assembled fragments 2_1, 2_2, 2_3 and 2_4; respectively. For all the assembly vectors, the restriction digestion showed two bands of around 1.7 and 6.3 kb. The assembly vector was methylated by the M.Osp807II switch methylase and the fragments by the M2.BsaI non-switchable methylase.

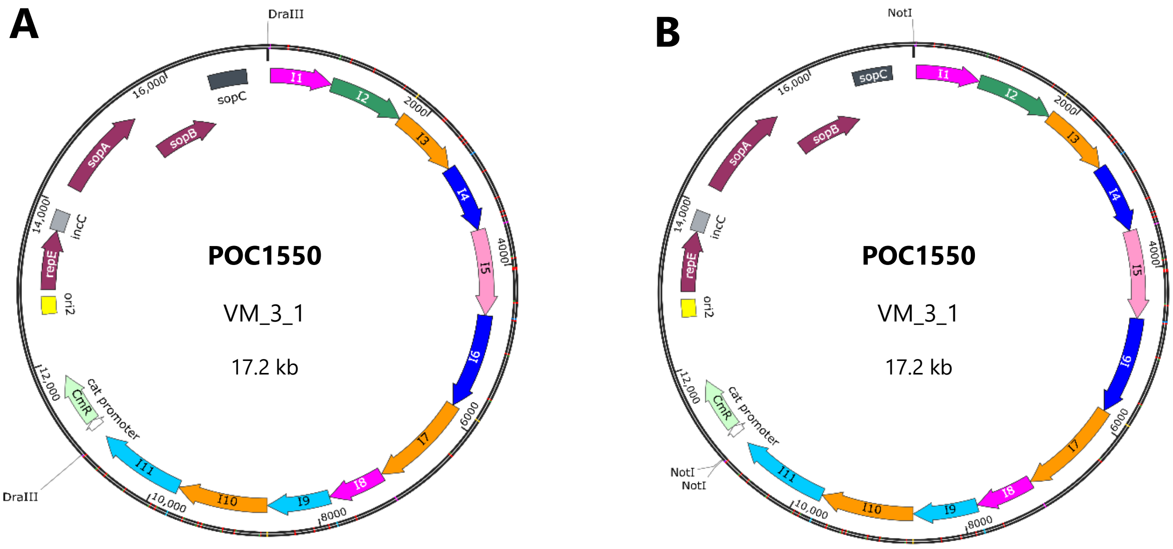

Figure S24. Assembled plasmid POC1550 from the second round of the hierarchical assembly using UniClo. Assembled plasmid POC1550 from the second round of the scarless hierarchical assembly using UniClo showing (**A**) the two DraIII and (**B**) the two NotI restriction sites used to confirm the correctly assembled fragment 3_1.

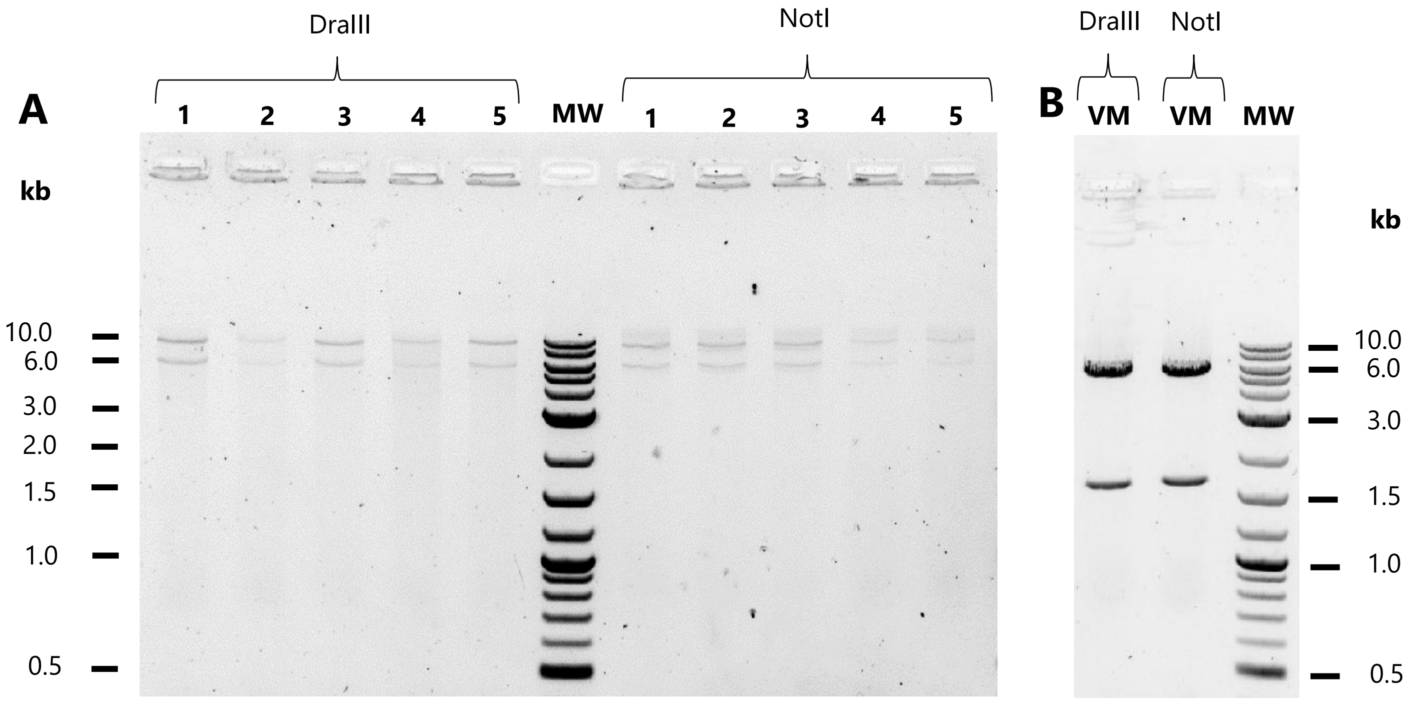

Figure S25. Agarose gel electrophoresis of the assembled plasmid POC1550 from the second round of the hierarchical assembly using UniClo. Agarose gel electrophoresis (1%) showing the restriction by DraIII and NotI of the assembled plasmid POC1550 from the second round of assembly, and the assembly vector VM (POC1519) as control. For the assembly, the assembly vector was methylated by the M.Osp807II switch methylase and the fragments by the M2.BsaI non-switchable methylase. (**A**) Lanes: 1 to 5, POC1550 extracted from five white colonies. The restriction digestion analysis of POC1550 with DraIII and NotI showed a band of 6.3 kb corresponding to the plasmid backbone and a band of 10.8 kb corresponding to the assembled fragment 3_1. (**B**) VM, assembly vector VM (POC1519). The restriction digestion of the assembly vector VM (POC1519) with DraIII and NotI showed two bands of around 1.7 and 6.3 kb. MW, molecular weight marker (Quick-Load® Purple 1 kb Plus DNA Ladder, New England Biolabs, N0550S, 0.1 to 10 kb).

References

1. Flores-Fernández,C.N., Lin,D., Robins,K. and O'Callaghan,C.A. (2024) DNA methylases for site-selective inhibition of type IIS restriction enzyme activity. *Appl. Microbiol. Biotechnol.*, **108**, 174.

2. Blake,W.J., Chapman,B.A., Zindal,A., Lee,M.E., Lippow,S.M. and Baynes,B.M. (2010) Pairwise selection assembly for sequence-independent construction of long-length DNA. *Nucleic Acids Res.*, **38**, 2594–2602.

3. Chen,W.-H., Qin,Z.-J., Wang,J. and Zhao,G.-P. (2013) The MASTER (methylation-assisted tailorable ends rational) ligation method for seamless DNA assembly. *Nucleic Acids Res.*, **41**, e93–e93.

4. Leguia,M., Brophy,J.A.N., Densmore,D., Asante,A. and Anderson,J.C. (2013) 2ab assembly: a methodology for automatable, high-throughput assembly of standard biological parts. *J. Biol. Eng.*, **7**, 2.

5. Weber,E., Engler,C., Gruetzner,R., Werner,S. and Marillonnet,S. (2011) A modular cloning system for standardized assembly of multigene constructs. *PLoS One*, **6**.

6. Yu,D., Tan,Y., Sun,Z., Sun,X., Sheng,X., Zhou,T., Liu,L., Mo,Y., Jiang,B., Ouyang,N., *et al.* (2017) In Vitro Seamless Stack Enzymatic Assembly of DNA Molecules Based on a Strategy Involving Splicing of Restriction Sites. *Sci. Rep.*, **7**, 1–10.

7. De Paoli,H.C., Tuskan,G.A. and Yang,X. (2016) An innovative platform for quick and flexible joining of assorted DNA fragments. *Sci. Rep.*, **6**, 19278.

8. Lin,D. and O'Callaghan,C.A. (2018) MetClo: methylase-assisted hierarchical DNA assembly using a single type IIS restriction enzyme. *Nucleic Acids Res.*, **46**.

9. Liu,S., Xiao,H., Zhang,F., Lu,Z., Zhang,Y., Deng,A., Li,Z., Yang,C. and Wen,T. (2019) A seamless and iterative DNA assembly method named PS-Brick and its assisted metabolic engineering for threonine and 1-propanol production. *Biotechnol. Biofuels*, **12**, 1–20.

10. Taylor,G.M., Mordaka,P.M. and Heap,J.T. (2019) Start-Stop Assembly: a functionally scarless DNA assembly system optimized for metabolic engineering. *Nucleic Acids Res.*, **47**, e17–e17.

11. Pryor,J.M., Potapov,V., Bilotti,K., Pokhrel,N. and Lohman,G.J.S. (2022) Rapid 40 kb Genome Construction from 52 Parts through Data-optimized Assembly Design. *ACS Synth. Biol.*, **11**, 2036–2042.

12. Matsumura,I. (2022) Golden Gate Assembly of BioBrick-Compliant Parts Using Type II Restriction Endonucleases. *Biotechniques*, **72**, 185–193.

13. Enghiad,B., Xue,P., Singh,N., Boob,A.G., Shi,C., Petrov,V.A., Liu,R., Peri,S.S., Lane,S.T., Gaither,E.D., *et al.* (2022) PlasmidMaker is a versatile, automated, and high throughput end-to-end platform for plasmid construction. *Nat. Commun.*, **13**, 2697.
